# Adherence to Dihydroartemisinin-Piperaquine Treatment among Patients with Uncomplicated Malaria in Northern Ghana

**DOI:** 10.1155/2019/5198010

**Published:** 2019-04-01

**Authors:** Abraham Rexford Oduro, Samuel Chatio, Paula Beeri, Thomas Anyorigiya, Rita Baiden, Philip Adongo, Patricia Akweongo

**Affiliations:** ^1^Navrongo Health Research Centre, Navrongo, Ghana; ^2^University of Health and Allied Sciences, Ho, Ghana; ^3^School of Public Health, University of Ghana, Accra, Ghana

## Abstract

Treatment adherence has been described as the process whereby patients take medications, follow diet, and effect other lifestyle changes that relate to agreed recommendations from healthcare providers. The determinants of such treatment adherence include patient, the health condition, therapy type, socioeconomic conditions, and the healthcare system. The study examined adherence in malaria patients treated with dihydroartemisinin-piperaquine in routine clinical care in northern Ghana. The study was conducted in Navrongo Health Research Centre in the Kassena-Nankana district of northern Ghana. Patients confirmed with uncomplicated malaria were prescribed dihydroartemisinin-piperaquine in blister packs to be taken daily for three days. Follow-up visits were made on days 3 and 28 after diagnosis to collect data on adherence, drug safety and therapeutic effectiveness. During follow-up visits, in-depth interviews were conducted and the blister packs directly observed for the number of tablets remaining. The in-depth interviews documented day-by-day account of doses taken, number of tablets taken during each dose, time of each dose, reasons for any leftover or missed dose, and whether or not there was vomiting. Treatment adherence was classified as definitely nonadherent, incomplete adherence, and completely adherent. A total of 405 patients were screened; 299 were positive by rapid diagnostic testing and 216 by microscopy. The average age was 12 years and females represented 54.0%. All participants completed day 3 follow-up but 12.7% had leftover pills. Treatment adherence was 50.9% (95% CI 44.1, 57.8), 36.1% (95% CI 29.7, 42.9), and 13.0% (95% CI 8.8, 18.2) for completely adherent, incomplete adherence, and definitely nonadherent, respectively. All completely adherent patients were free of parasitemia on day 28 of follow-up. A total of 49 adverse events related to malaria symptoms were documented. Effort to improve adherence should be individualized as it is dependent on a number of factors such as the patients' temperament, the disease, support at home, and complexity of treatment.

## 1. Introduction

Treatment adherence refers to the process whereby patients take medications, follow diet, and effect other lifestyle changes that relate to agreed recommendations from healthcare providers [1–4]. The determinants of medication adherence may be related to the patient, therapy, health condition, and socioeconomic or the healthcare system characteristics [1–4]. For patient factors, these often relate to lack of understanding of their disease condition, lack of involvement in the treatment decision-making process, and suboptimal medical literacy, all of which influence treatment adherence. A patient's beliefs and attitudes concerning treatment effectiveness, their previous experiences with treatment, and fear of adverse events among others can affect adherence to treatment. Improved patients level of understanding and knowledge of their diagnosis and treatment can therefore empower them to be adherent to therapy [5–9].

The most important therapy-related factors associated with treatment adherence are, however, adverse effects. The potential adverse effects associated with a specific drug can cloud the ability of a patient to adhere to a prescribed therapy whether the patient has experienced it personally previously or is from another source [3, 5]. Other therapy-related factors are those associated with packaging and complexity of treatment schedules. Adverse effects are also the main contributors to nonadherence in asymptomatic disease conditions. In addition, concomitant conditions and comorbidities influence treatment adherence [3, 5].

Socioeconomic factors such as possession of health insurance, out-of-pocket expenses, and support from relations also influence treatment adherence. An increase in cost sharing makes patients less likely to adhere to a prescribed therapy, with the possibility of either delaying or never initiating it. This is because if a patient does not have the means, it is unlikely that he or she will seek the appropriate care necessary [5, 7]. In addition, healthcare systems can create barriers to treatment adherence. For instance, a cordial communication between clients and health service providers influences adherence to treatments as a misinterpretation of a healthcare provider in terms of the diagnosis and drug regimen can undermine adherence. Other health systems factors affecting adherence include poor availability of information technology for easy accessibility of information and poor patient-physician communication. The amount of time a clinician spends with patients may be insufficient to educate patients on adherence to treatment [5, 10]. The general interventions that improve adherence to prescribed medications are more successful for short-term than for long-term treatments and chronic illnesses [4]. Poor adherence to treatment regimens has long been recognized as a substantial barrier to achieving better outcomes for patients. Studies have shown that at any given time as many as half of all patients do not adhere faithfully to their prescription-medication regimens causing substantial hospital admissions and cost. Poor treatment adherence is well widespread and recognized and it remains difficult to determine which patients will or will not adhere to their medication as directed and good adherence reduces adverse events, severe disease, and overall mortality [3, 4, 11].

Prompt diagnosis, treatment, and adherence to dosage regimen of antimalarials provide individual benefits of curing infection and preventing progression to severe disease, and community-level benefits by reducing the infectious reservoir, avert reinfection, and the emergence and spread of drug resistance [12]. Although several studies have evaluated the efficacy, tolerance, and adherence to antimalarial drugs [12–15], there is limited information on patients' adherence to dihydroartemisinin-piperaquine in routine clinical care in Ghana despite its widespread use. The study examined the proportion of patients with uncomplicated malaria treated with dihydroartemisinin-piperaquine who adhered to the recommended age-specific course of treatment.

## 2. Methods

### 2.1. Study Settings

The study was conducted in Navrongo Health Research Centre located in Kassena-Nankana District (KND) of northeastern Ghana. The KND is on latitude 10°30′ and 11°00′N and longitude 1°00′ and 1°30′W and cover surface area of 1,674 square kilometers. [15]. The Navrongo Health and Demographic Surveillance System monitors health and demographic dynamics of the study area [16]. Ecologically, the vegetation of the area is Sahelian savanna characterized by a short rainy season and a prolonged dry season from October to March. Malaria transmission in the area has been characterized as hyperendemic with distinct seasonal patterns of peak transmission during the major rains and low rates of infection during the dry season [17–19]. The health system of the study area follows that of the Ghana health system with one referral hospital located in the district capital, eight health centres for secondary curative and preventive health care, and about thirty community-based health planning and services clinics located in various communities to provide primary healthcare services. At the time of the study, there were three private clinics, two pharmacy shops, and over fifty drug and chemical shops in the study area.

### 2.2. Study Design and Population

The study area has five demarcated zones (North, South, East, West, and Central) based on the Navrongo Health and Demographic Surveillance System. Two of the zones (Central and South) were selected to represent the urban and rural areas, respectively, for this study. All the healthcare workers at the selected health facilities in the selected zones were retrained on current malaria treatment guidelines including dihydroartemisinin-piperaquine use for uncomplicated malaria. Project staff had training on study procedures and how to administer the questionnaire after it had been pretested. All patients who reported to the health facilities in the selected zones and who reported fever or history of fever in the previous 24 hours and who had positive malaria parasitemia were eligible for recruitment. A rapid diagnostic test (RDT) was used to screen for malaria parasites. Blood smears were also prepared for microscopic confirmation of the malaria before enrolment into the study. The two tests were done concurrently on the same samples. This is because persistence of the HRP2 antigen means that patients continue to test positive with HRP2-based RDTs long after the patient has been treated and the parasites had been eliminated. The two tests therefore helped to distinguish between lingering positivity and de novo infection in this high-transmission area where patients frequently experience repeated malaria infections and treatments. Adult patients, parents, or caregivers who accompanied the patient to the health facility responded to the interview questions where necessary. Recruitment of cases started in October 2014 and ended in November 2015.

### 2.3. Participant Selection and Data Gathering

All patients had to satisfy the study selection criteria. Inclusion criteria were a positive malaria test by microscopy, a diagnosis of uncomplicated malaria, written informed consent, age ≥6 months and weight ≥5 kg, capability of taking an oral medication, resident in the study area, and being available for follow-up. Exclusion criteria were unwillingness to provide informed consent, known pregnancy, lactating mothers, complicated malaria or severe disease, and nonresident in the study area or intention to move out within one month. Written informed consent was obtained from all 18-year-old or older patients prior to blood smear preparation. Parental consent was obtained for all patients below 18 years of age. In addition, patients aged 12–17 years gave assent to be eligible for enrolment into the study. A structured questionnaire was developed and administered in the form of an interview at enrolment and during follow up visits. At enrolment, baseline data including sociodemographic characteristics, place of residence, and the characteristic of the current medications prescribed were collected.

### 2.4. Study Drug

Dihydroartemisinin-piperaquine brand (Duo-Cotecxin™, manufactured by Zhejiang Holley Nanhu Pharmaceutical Company Limited, China; Lot Number 131121; date of manufacture 12/11/2013 and date of expiry 11/2015), which was in the market at the time and was being prescribed, was used in the study. Treatment doses of dihydroartemisinin-piperaquine (Duo-Cotecxin™) were as follows: 20/160 mg (dihydroartemisinin-piperaquine) children dose was given as one tablet per day for those 5 kg to <10 kg and two tablets per day for those 10 kg to <20 kg and for the adults the dosage was 40/320 mg (dihydroartemisinin-piperaquine) administered as two tablets per day for those between 20 kg and <40 and 3 tablets per day for those ≥ 40 kg. All the treatment doses were given once daily for three days. The non-artemisinin agent piperaquine is an oral active bisquinoline that is structurally similar to chloroquine. Both piperaquine and chloroquine have similar targets through the inhibition of the heme-digestion pathway in the food vacuole of the parasite [20].

### 2.5. Treatment and Follow-Up

All patients prescribed with dihydroartemisinin-piperaquine (DP) were supplied with blister packs of the drug for the three-day treatment course for their current illness episodes and were taught how to take their medications at home. Participants were asked to repeat a dose anytime vomiting occurred within one hour of taking the scheduled medication. Follow-up of all enrolled patients was made to their homes 3 days after the health facility visit. During follow-up visits, consent was sought prior to in-depth interviews with patients or parents/guardians at home using a structured questionnaire. The DP blister packs were directly observed and the number of tablets remaining was counted. The in-depth interview included a day-by-day account of the number of doses taken, number of tablets taken during each dose, time of each dose, reasons for any leftover or missed dose, and whether or not there was vomiting. When study participants or caregivers were not available on the day of visit, attempts were made again to trace them within the next two days in order to minimize recall bias. Patients were considered lost to follow-up if still not found on rescheduled visits. Additional follow-up visits were made 28 days after initial diagnosis to collect further data on drug safety and to monitor therapeutic effectiveness of study drug. During the visit, if a patient was found to be still sick, s/he was immediately referred to the health facility for evaluation and further treatment.

### 2.6. Classification of Adherence

By combining the responses to the oral interviews and the physical tablet count from the blister pack, patient adherence to treatment was classified into three categories: definitely nonadherent, incomplete adherence, and completely adherent. A patient who did not take the tablets at all or as recommended by the time of follow-up with physical count of the pills showing a greater than expected number of pills was classified as definitely nonadherent. Any patient (s) who reported that they did not take all the doses as recommended at the time of follow-up or any case where a physical count of the pills showed a greater than or less than the expected number of pills was classified as incomplete adherence. Complete adherence was a case where a patient reported taking all doses as recommended at the time of follow-up visit and a physical count of the pills showed the expected number of pills was taken and no pill left in the pack.

### 2.7. Ethics Approval and Consent to Participate

Ethical clearance was obtained from the Ghana Health Service Ethics Committee and Navrongo Institutional Review Board.

## 3. Results

### 3.1. Background Characteristics

A total of 405 participants were screened for inclusion into the study. Of this number, 299 (73.8%) tested positive by rapid malaria diagnostic testing (RDT). Of the 299 positive RDT patients, 216 (72.2%) were positive by microscopy, 36.6% for those <5 years, 47.5% for the 5-17 years old, and 16.2% for those ≥ 18 years. All the microscopically confirmed malaria patients were enrolled into the study. Overall female patients represented 54.0% (117/216) of the enrolled participants. The average age of the enrolled patients was 12 years; 11.6 years for males and 12.9 years for females, respectively. About 37% (79/216) of the participants were below 5 years of age, 47% (102/216) were 5 to 17 years old, and 16% (35/216) were 18 years old or older. The total geometric mean parasite density at enrolment was 6,908 (95% CI 4914, 9711) parasites per microlitre of blood. The highest parasite burden, 10,053 (95% CI 5828, 17340) parasites per microlitre of blood, was in children aged below 5 years, 9294 (5744, 15040) was in 5-17 years, and 1058 (552, 2869) was in 18 years old and older.  Table 1 presents additional characteristics of the patients and details of the study drug administration.

All (100%) the 216 participants recruited were met 3 days after initial visit to the health facility. The study completion rate at day 28 was therefore 99.1% (214/216). Two of the participants in the <5years old and 5 to 17 years old group were not met on day 28 visit and were therefore declared lost to follow-up. Total microscopy positive for malaria parasites on day 28 was 2.8% (6/214): 2.6% (2/78), 4.0% (4/101), and 0/0% (0/35) for the <5 years old, 5 to 17 years old, and those eighteen years and above, respectively. In all, participants with pills after day 3 were 13.0% (28/216). The total number of participants that took study drugs as instructed was 123 (56.9%) and those that did not take the study drugs as instructed were 93 (43.1%). The total number of participants that took the study drugs as instructed and repeated dose after vomiting was 110 (50.9%) and those that took the drugs as instructed but did not repeat dose after vomiting were 13 (6%).

Overall, 43.1% of the participants did not take their medication as instructed and 28 participants (13%) had leftover pills three days after first visit to the health facility. Among the 123 (56.9%) participants who reported that they had taken their medication as instructed by the healthcare provider, 13 (10.6%) reported vomiting within an hour of their doses but did not repeat the dose.  Table 1 presents the demographic characteristics of study participants and treatment administration outcomes among the enrolled patients.

### 3.2. Level of Patient Adherence to Treatment

**Figure 1:**
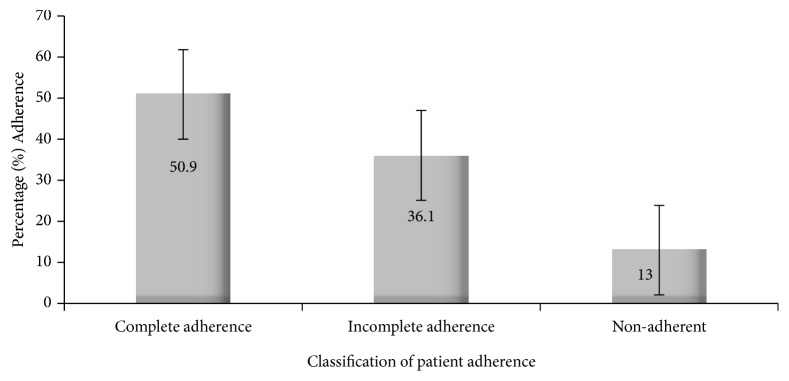
Classification of patient adherence to dihydroartemisinin-piperaquine treatment for uncomplicated malaria. Complete adherence, incomplete adherence, and nonadherence of enrolled patients to dihydroartemisinin-piperaquine.

Figure 1 presents the level of patient adherence to treatment in the study. Patient adherence to treatment was classified as complete in 50.9% (95% CI 44.1, 57.8) of the patients and increased with age from a low of 43.0% (95% CI 31.9, 54.7) in patients aged below 5 years and 53.9% (95% CI 43.8, 63.8) in patients aged 5-17 years to 60.0% (95% CI 42.1, 76.1) in patients aged 18 years or older (Table 2). Complete adherence was higher (52.1% (95% CI 42.7, 61.5)) in female patients than in male patients (49.5% (95% CI 39.3, 59.7)), although the difference was not significant (p=0.7). Incomplete adherence was found in 36.1% (95% CI 29.7, 42.9) of the patients with 13.0% (95% CI 8.8, 18.2) of them being classified as definitely nonadherent. Patients who were completely adherent to treatment were free of parasitemia on day 28 of follow-up. Of the 6 patients who were parasitemic on day 28, five were classified as incomplete adherents and one as definitely nonadherent. There was no demonstrable relationship between parasite density and adherence to treatment (Table 2).

### 3.3. Reasons for Nonadherence

A total of 28 patients were classified as definitely nonadherent for not taking all the doses. Four of the 28 patients (14.3%) indicated that they felt well and so decided to keep the medication for future use. Similarly, 28.6% (8/28) felt well and so did not know it was necessary to continue the medication. Most of them, 85.7% (24/28), skipped their doses but had plans to continue taking the medication in subsequent days after the visit. Side effects experienced by 10.7% (3/28) were also mentioned as a reason for incompletion of the medication. Other less frequent reasons, forgetfulness, care-giver or participant not at home, and participant refusing to take medication, accounted for 17.9% (5/28). For the participants categorized as definitely nonadherent, they were asked what they would do with the remaining drugs. While 90% (25/28) reported they would continue with the medication, 10.7% (3/28) reported they would discard the remaining drugs. Only one participant reported that the drugs would be saved and used again when there is another illness episode.

### 3.4. Adverse Events Reported

A total of 49 adverse events were associated with DP treatment as reported by the study participants. The most commonly reported events were weakness, 32.6% (16/49), dizziness, 18.4% (9/49), abdominal pain, 12.2% (6/49), itching, 10.2% (5/49), headache, 8.2% (4/49), and loss of appetite, 4.1% (2/49). There were other adverse events reported, which summed up to 14.3% (7/49). By age, most, 36.7% (18/49), of the adverse events were reported by patients aged 5-17 years followed by 32.6% (16/49) in those below 5 years of age.  Table 3 presents further details of the distribution of reported adverse events.

### 3.5. Reasons Advanced for Preference of DP in Another Malaria Episode

Most, 97.2% (210/216), of the patients reported that they would prefer the same treatment again if they experienced another episode of malaria. Among the reasons for this preference, 49.0% (103/210) of the patients perceived DP to be effective; 43.3% (91/210) of the patients reported that they experienced no side effects and so would like to have the same treatment if they had another malaria episode. Good taste, 5.2% (11/210), and the fewer number of tablets, 2.4% (5/210), were the other reasons given by patients for the choice of DP for their next malaria episode. Of the 6 patients who preferred a different treatment other than DP for their next malaria episode, 3 perceived DP as ineffective and the remaining 3 complained of the side effects experienced for their decisions.

## 4. Discussion

Adherence to malaria treatment is one of the cornerstones of the disease control as prompt treatment cures infection, delay drug resistance and reduces infectious reservoir and transmission [21]. Detecting adherence to treatment includes both direct and indirect methods like self-reporting, interviews, therapeutic outcome, and pill count, among others. The direct measures involving biological markers, tracer compounds, biological assays, and monitors are more reliable but are not used in routine practice [22].

In this study, we did not use self-reported approach as the most feasible method for assessing patient adherence to medication because it is prone to recall bias and social desirability bias [22, 23]. Despite the public health efforts at improving adherence to malaria treatment, it remains suboptimal in all methods, age groups, gender, and type of drugs [23, 24]. One of the common reasons given by patients in this study for failure of compliance in treatment was the fact that they felt well and so decided to keep the medication for future use. Others skipped their doses but had plans to continue taking the medication in subsequent days and some did not know it was necessary to complete the medication. Others include adverse effect, forgetfulness and care-giver not at home, and participant refusing to take medication, all consistent with findings from other self-reported studies [25, 26].

From the study results, complete patient adherence to treatment was about 51% (95% CI 44.1, 57.8) of the patients and this increased with age and was higher in female patients than in male patients, although the difference was not significant. The proportion of complete adherence in the study was lower because higher adherence to treatment is often associated with acute and life-threatening diseases like malaria except that most of those findings have come from self-reporting studies [26]. This study has verified the blister packs used by the participants directly to determine the number of tablets of the study drugs used and this could have accounted for the low adherence compared to the self-reported studies. Other factors that have been documented to be associated with higher adherence to treatment include trust in the physician, being married, older, employed, not smoking, or drinking associated with higher compliance [27].

Overall, about two-fifths of the participants did not take their medication as instructed and about one in ten had leftover pills three days after first visit to the health facility. Even among the those participants who reported that they had taken their medication as instructed by the healthcare provider, about one in ten reported vomiting but did not repeat the dose. This resulted in about 36% of the patients being classified as incomplete adherence and 13% being classified as definitely nonadherent. Patients who were completely adherent to treatment were free of parasitemia at the end of the follow-up, while for patients who were parasitemic, majority of them were classified as nonadherent. However, there was no demonstrable relationship between parasite density and adherence to treatment.

Treatment nonadherence is the most common cause of treatment failure. Failure of medical treatment may alternate treatment or cause a change in dose schedule and this may lead to the removal of effective drugs or subject patient to dangers of adverse effect of the alternative drugs [28, 29].

Methods to improve treatment adherence include different packaging and new dosage forms. Others are health education, behavioral oriented programmes, tailoring medication taking to daily habits, and treatment contracts. The most significant finding for treatment adherence is the information given to the patient by the doctor as opposed to other healthcare providers, which increased the need required by the patient to take the medication [30, 31].

### 4.1. Study Limitations

Self-report is the most common and feasible method for assessing patient adherence to medication, but it can be prone to recall bias and social desirability bias. As such, this study had to verify the blister packs used by the participants directly to determine the number of tablets of the study drugs used by participants and this could have accounted for the low adherence compared to the self-reported studies. This could have also led to breach of patients confidentiality.

## 5. Conclusions

Healthcare professionals should be aware of the importance of noncompliance to antimalarials because of the dangers associated with drug resistance and severe and complicated malaria. Patient education is a cornerstone in achieving compliance to malaria treatment and a good patient-clinician relationship is equally vital. Failure of malaria treatment due to noncompliance remains high and so prescribers must take time to improve adherence to malaria treatment.

## Figures and Tables

**Table 1:** Characteristics of study participants and treatment dose outcomes.

Characteristics of study participants	Number (percentage) of participants				
	Male	Female	<5 years	5-17 years	>18 years
Number enrolled	99 (45.8)	117(54.2)	79(36.6)	102(47.2)	35(16.2)
Completed day 3 follow-ups	99(100)	117(100)	79(100)	102(100)	35(100)
Completed day 28 follow-ups	99(100)	115(98.3)	78(98.7)	101(99.0)	35(100)
Lost to follow-up by day 28	1(1.0)	1(0.9)	0(0)	1(1.0)	1(2.9)
Did not take drugs as instructed	47(47.5)	46(39.3)	36(45.6)	44(43.1)	13(37.1)
Took drugs as instructed	52(52.5)	71(60.7)	43(54.4)	58(56.9)	22 62.9)
Did not repeat dose after vomiting	5(5.1)	8(6.8)	10(12.7)	3(2.9)	0 (0)
Participants with pills after day 3	16(16.2)	12(10.2)	10(12.7)	15(14.7)	3(8.6)

**Table 2:** Classification of patient adherence by patient attributes.

Patient attributes	Total number	Completely adherent	Incomplete adherence	Definitely nonadherent
Total	216	50.9(44.1,57.8)	36.1(29.7, 42.9)	13.0(8.8,18.2)
< 5years	79	43.0(31.9,54.7)	44.3(33.1,55.9)	12.7(6.2,22.0)
5-17 years	102	53.9(43.8,63.8)	31.4(22.5,41.3)	14.7(8.5,23.1)
≥18 years	35	60.0(42.1,76.10	31.4(16.9,49.3)	8.6(1.8,23.1)
Males	99	49.5(39.3,59.7)	34.3(25.1,44.6)	16.2(9.5,24.9)
Females	117	52.1(42.7,61.5)	37.6(22.8,47.0)	10.3(5.4,17.2)
<1000 ul	53	60.4(46.0,73.5)	30.2(18.3,44.3)	9.4(3.1,20.7)
1000-9999 ul	27	55.6(35.3,74.5)	37.0(19.4,57.6)	7.4(0.9,24.3)
10000-19999 ul	29	65.5(45.7,82.1)	27.6(12.7,47.2)	6.9(0.8,22.8)
>20000 ul	107	41.1(31.7,51.0)	41.1(31.7,51.0)	17.8(11.0,26.3)
Positive on day 28	6	0(0,0)	83.3(35.9,99.6)	16.7(0.4,64.1)

**Table 3:** Frequencies of adverse events following the DP treatment.

Description	Age category			
	<5 years, n	5-17 years, n	≥18 years, n	Total, n (%)
Headache	2	0	2	4 (8.2)
Weakness	3	7	6	16 (32.6)
Dizziness	3	4	2	9 (18.4)
Abdominal Pain	1	3	2	6 (12.2)
Itching	2	1	2	5 (10.2)
Loss of appetite	0	1	1	2 (4.1)
Others	5	2	0	7 (14.3)
Total	16	18	15	49(100)

## Data Availability

The dataset used to support the findings of this study are provided as supplementary material.

## Supplementary Materials

Supplementary Materials Dataset used to produce the results of this manuscript.
