# Supplementary material for: Adherence to Dihydroartemisinin-Piperaquine Treatment among Patients with Uncomplicated Malaria in Northern Ghana
Source: J Trop Med. 2019 Apr 1;2019:5198010. doi: 10.1155/2019/5198010 (PMC6463560; doi:10.1155/2019/5198010)
Supplement: Supplementary Materials — Dataset used to produce the results of this manuscript. [file 5198010.f1.pdf]

idno	q1	q2	q10	q11	q12	q12a	q17a	q18	q19	q19a	q20	q21	q21b
1	20-Oct-14	23-Oct-14	F	24-Mar-05	888		NA	C	8	NA	23-Oct-14	7	34
2	20-Oct-14	23-Oct-14	F	15-Jun-04		Y	NA	C	8	NA	23-Oct-14	9	4
3	20-Oct-14	23-Oct-14	F	10-Dec-71		Y	NA	C	8	NA	23-Oct-14	6	40
4	20-Oct-14	23-Oct-14	F	13-May-72		Y	NA	C	8	NA	23-Oct-14		
5	20-Oct-14	23-Oct-14	F	4-Jan-09		Y	NA	C	8	NA	23-Oct-14	10	5
6	21-Oct-14	24-Oct-14	F	5-Nov-07		Y	NA	C	8	NA	24-Oct-14	8	0
7	21-Oct-14	24-Oct-14	M	25-Feb-03		Y	NA	C	8	NA	24-Oct-14	8	18
8	21-Oct-14	24-Oct-14	M	14-May-05	9	Y	NA	C	8	NA	24-Oct-14	9	51
9	21-Oct-14	24-Oct-14	F	7-Sep-06	8	Y	NA	C	8	NA	24-Oct-14	7	34
10	21-Oct-14	24-Oct-14	M	7-Aug-04		Y	NA	C	8	NA	24-Oct-14	6	52
11	28-Oct-14	31-Oct-14	F	15-Jun-71		Y	NA	C	8	NA	31-Oct-14	7	23
12	28-Oct-14	31-Oct-14	F	8-Dec-11	2	Y	NA	C	8	NA	31-Oct-14	9	8
13	28-Oct-14	31-Oct-14	M	14-Feb-01	13	Y	NA	C	8	NA	31-Oct-14	8	20
14	3-Nov-14	6-Nov-14	M	11-Mar-12		Y	NA	C	8	NA	6-Nov-14	8	12
15	3-Nov-14	6-Nov-14	M	27-Apr-09		Y	NA	C	8	NA	6-Nov-14	6	50
16	3-Nov-14	6-Nov-14	F	15-Jun-88		Y	NA	C	8	NA	6-Nov-14	7	48
17	3-Nov-14	6-Nov-14	F	25-Jul-00		Y	NA	C	8	NA	6-Nov-14	8	43
18	4-Nov-14	7-Nov-14	M	1-Mar-89	25	Y	NA	C	8	NA	7-Nov-14	7	22
19	4-Nov-14	7-Nov-14	F	15-Jul-66		Y	NA	C	8	NA	7-Nov-14	7	41
20	4-Nov-14	7-Nov-14	F	13-Jul-03	11	Y	NA	C	8	NA	7-Nov-14	7	56
21	4-Nov-14	7-Nov-14	M	18-May-10		Y	NA	C	8	NA	7-Nov-14	8	11
22	4-Nov-14	7-Nov-14	F	24-Jul-10	4	Y	NA	C	8	NA	7-Nov-14	6	41
23	4-Nov-14	7-Nov-14	M	27-Jul-06	8	Y	NA	C	8	NA	7-Nov-14	8	43
24	4-Nov-14	7-Nov-14	F	6-Oct-52			NA	C	8	NA	7-Nov-14	8	59
25	10-Nov-14	13-Nov-14	F	22-Jun-01		Y	NA	C	8	NA	13-Nov-14	7	5
26	10-Nov-14	13-Nov-14	F	26-Apr-06		Y	NA	C	8	NA	13-Nov-14	8	5
27	10-Nov-14	13-Nov-14	F	2-Nov-10		Y	NA	C	8	NA	13-Nov-14	8	40
28	10-Nov-14	13-Nov-14	F	7-Apr-01		Y	NA	C	8	NA	13-Nov-14		
29	10-Nov-14	13-Nov-14	F	5-Mar-02	12	Y	NA	C	8	NA	13-Nov-14	6	47
30	11-Nov-14	14-Nov-14	M	31-Aug-12	2	Y	NA	C	8	NA	14-Nov-14	9	5
31	11-Nov-14	14-Nov-14	F	10-Jun-02	12	Y	NA	C	8	NA	14-Nov-14	7	53
32	11-Nov-14	14-Nov-14	F	10-Nov-05		Y	NA	C	8	NA	14-Nov-14	7	11
33	11-Nov-14	14-Nov-14	F	3-Jan-11		Y	NA	C	8	NA	14-Nov-14	8	27
34	17-Nov-14	20-Nov-14	F	11-May-74		Y	NA	C	8	NA	20-Nov-14	7	17
35	17-Nov-14	20-Nov-14	F	15-Sep-83	31	Y	NA	C	8	NA	20-Nov-14	7	53
36	17-Nov-14	18-Nov-14	F	5-Apr-84	30	Y	NA	C	8	NA	20-Nov-14	9	12
37	17-Nov-14	20-Nov-14	F	5-May-00	14	Y	NA	C	8	NA	20-Nov-14	8	25
38	24-Nov-14	27-Nov-14	F	19-Aug-10	4	Y	NA	C	8	NA	27-Nov-14	7	44
39	24-Nov-14	27-Nov-14	F	26-Jun-02	12	Y	NA	C	8	NA	27-Nov-14	7	18
40	28-Nov-14	1-Dec-14	F	11-Sep-08		Y	NA	C	8	NA	1-Dec-14	7	7
41	2-Dec-14	5-Dec-14	F	5-Mar-07		Y	NA	C	8	NA	5-Dec-14	7	15
42	8-Dec-14	11-Dec-14	M	19-Mar-10		Y	NA	C	8	NA	11-Dec-14	8	11
43	9-Dec-14	12-Dec-14	F	23-Jul-09		Y	NA	C	8	NA	12-Dec-14	8	45
44	15-Dec-14	18-Dec-14	M	1-Dec-12		Y	NA	C	8	NA	18-Dec-14	7	10
45	16-Dec-14	19-Dec-14	F	16-Jul-13		Y	NA	C	8	NA	19-Dec-14	11	28
46	17-Dec-14	20-Dec-14	F	2-Apr-10		Y	NA	C	8	NA	20-Dec-14	8	39

47	18-Dec-14	21-Dec-14	M	30-Jun-11		Y	NA	C	8	NA	21-Dec-14	6	37
48	18-Dec-14	21-Dec-14	F	20-May-03		Y	NA	C	8	NA	21-Dec-14	7	5
49	22-Dec-14	25-Dec-14	M	15-Jun-90		Y	NA	C	8	NA	25-Dec-14	7	35
50	22-Dec-14	25-Dec-14	M	20-Mar-12		Y	NA	C	8	NA	25-Dec-14	8	0
51	22-Dec-14	25-Dec-14	M	2-Oct-07		Y	NA	C	8	NA	25-Dec-14	6	58
52	22-Dec-14	25-Dec-14	F	13-Nov-99		Y	NA	C	8	NA	25-Dec-14	8	58
53	22-Dec-14	25-Dec-14	F	8-Jun-13		Y	NA	C	8	NA	25-Dec-14	7	50
54	22-Dec-14	25-Dec-14	M	9-May-08		Y	NA	C	8	NA	25-Dec-14	8	4
55	23-Dec-14	26-Dec-14	F	8-Jun-97		Y	NA	C	8	NA	26-Dec-14	10	3
56	23-Dec-14	26-Dec-14	F	12-Jan-83		Y	NA	C	8	NA	26-Dec-14	7	44
57	23-Dec-14	26-Dec-14	M	4-May-04		Y	NA	C	8	NA	26-Dec-14	8	5
58	29-Dec-14	1-Jan-15	F	10-Nov-04		Y	NA	C	8	NA	1-Jan-15	7	54
59	29-Dec-14	1-Jan-15	M	5-Aug-08		Y	NA	C	8	NA	1-Jan-15	8	45
60	30-Dec-14	2-Jan-15	F	18-Dec-04	10	Y	NA	C	8	NA	2-Jan-15	8	15
61	30-Dec-14	2-Jan-15	F	15-Jun-65		Y	NA	C	8	NA	2-Jan-15	9	10
62	30-Dec-14	2-Jan-15	F	15-Jun-03		Y	NA	C	8	NA	2-Jan-15	9	15
63	5-Jan-15	8-Jan-15	M	25-Dec-12		Y	NA	C	8	NA	8-Jan-15	9	35
64	5-Jan-15	8-Jan-15	F	17-Feb-10		Y	NA	C	8	NA	8-Jan-15	9	6
65	5-Jan-15	8-Jan-15	M	29-Apr-12		Y	NA	C	8	NA	8-Jan-15	8	6
66	6-Jan-15	9-Jan-15	F	18-Sep-06		Y	NA	C	8	NA	9-Jan-15	8	15
67	6-Jan-15	9-Jan-15	M	11-Dec-13		Y	NA	C	8	NA	9-Jan-15	9	15
68	6-Jan-15	9-Jan-15	F	6-Jul-07		Y	NA	C	8	NA	9-Jan-15	8	46
69	12-Jan-15	15-Jan-15	F	23-Jul-07	7	Y	NA	C	8	NA	15-Jan-15	9	10
70	12-Jan-15	15-Jan-15	F	26-Jan-48	66	Y	NA	C	8	NA	15-Jan-15	9	0
71	12-Jan-15	15-Jan-15	F	10-Apr-99	15	Y	NA	C	8	NA	15-Jan-15	9	13
72	20-Jan-15	23-Jan-15	F	15-Jun-49	65	Y	NA	C	8	NA	20-Jan-15	8	55
73	26-Jan-15	29-Jan-15	F	29-Sep-04	10	Y	NA	C	8	NA	26-Jan-15	8	25
74	26-Jan-15	29-Jan-15	M	10-Apr-73	41	Y	NA	C	8	NA	29-Jan-15	6	50
75	26-Jan-15	29-Jan-15	F	17-Nov-09	5	Y	NA	C	8	NA	29-Jan-15	7	17
76	2-Feb-15	5-Feb-15	F	19-Aug-10	4		NA	C	8	NA	5-Feb-15	10	45
77	2-Feb-15	5-Feb-15	F	13-Jan-11	3	Y	NA	C	8	NA	5-Feb-15	8	30
78	20-Jan-15	23-Jan-15	F	18-Jun-04	10	Y	NA	C	8	NA	23-Jan-15	7	15
79	16-Dec-14	19-Dec-14	M	20-Jul-70		Y	NA	C	8	NA	19-Dec-14	11	0
80	14-Sep-15	17-Sep-15	M	15-Apr-82			NA	C	8	NA	17-Sep-15	6	30
81	14-Sep-15	17-Sep-15	M	13-Jul-91			NA	C	8	NA	17-Sep-15	6	10
82	14-Sep-15	17-Sep-15	F	9-Nov-06			NA	C	8	NA	17-Sep-15	6	53
83	14-Sep-15	17-Sep-15	F	19-Jan-07			NA	C	8	NA	17-Sep-15	8	27
84	8-Sep-15	11-Sep-15	M	26-Mar-09			NA	C	8	NA	11-Sep-15	7	43
85	8-Sep-15	11-Sep-15	F	24-Mar-03			NA	C	8	NA	11-Sep-15	6	33
86	8-Sep-15	11-Sep-15	M	28-Jan-14			NA	C	8	NA	11-Sep-15	8	5
87	8-Sep-15	11-Sep-15	F	6-Mar-09			NA	C	8	NA	11-Sep-15	7	19
88	8-Sep-15	11-Sep-15	F	15-Aug-98			NA	C	8	NA	11-Sep-15	7	1
89	8-Sep-15	11-Sep-15	F	20-Feb-14			NA	C	8	NA	11-Sep-15	8	40
90	8-Sep-15	11-Sep-15	M	20-Feb-14	1	Y	NA	C	8	NA	11-Sep-15	8	54
91	8-Sep-15	11-Sep-15	F	21-Oct-07			NA	C	8	NA	11-Sep-15	6	40
92	8-Sep-15	11-Sep-15	F	9-Oct-12			NA	C	8	NA	11-Sep-15	7	32
93	8-Sep-15	11-Sep-15	F	15-Jun-92			NA	C	8	NA	11-Sep-15	7	7

94	7-Sep-15	10-Sep-15	F	15-Jun-55			NA	C	8	NA	10-Sep-15	7	50
95	7-Sep-15	10-Sep-15	F	23-Mar-12			NA	C	8	NA	10-Sep-15	7	34
96	7-Sep-15	10-Sep-15	F	16-Nov-66			NA	C	8	NA	10-Sep-15	9	15
97	7-Sep-15	10-Sep-15	F	15-Apr-36			NA	C	8	NA	10-Sep-15	10	30
98	3-Nov-15	6-Nov-15	F	11-Jan-10			NA	C	8	NA	6-Nov-15	9	21
99	3-Nov-15	6-Nov-15	M	23-Dec-05			NA	C	8	NA	6-Nov-15	7	5
100	3-Nov-15	6-Nov-15	F	12-Feb-07			NA	C	8	NA	6-Nov-15	7	48
101	3-Nov-15	6-Nov-15	M	7-Oct-08			NA	C	8	NA	6-Nov-15	8	7
102	3-Nov-15	6-Nov-15	M	25-Jun-13			NA	C	8	NA	6-Nov-15	6	36
103	3-Nov-15	6-Nov-15	M	15-Mar-13			NA	C	8	NA	6-Nov-15	7	30
104	3-Nov-15	6-Nov-15	F	26-Jan-10			NA	C	8	NA	6-Nov-15	7	18
105	3-Nov-15	6-Nov-15	F	30-Sep-11			NA	C	8	NA	6-Nov-15	6	5
106	3-Nov-15	6-Nov-15	M	4-Oct-09			NA	C	8	NA	6-Nov-15	10	5
107	3-Nov-15	6-Nov-15	F	8-Jan-02			NA	C	8	NA	6-Nov-15	8	55
108	3-Nov-15	6-Nov-15	F	15-Jun-80			NA	C	8	NA	6-Nov-15	8	20
109	3-Nov-15	6-Nov-15	F	26-Apr-09			NA	C	8	NA	6-Nov-15	7	10
110	3-Nov-15	6-Nov-15	M	15-Jun-96			NA	C	8	NA	6-Nov-15	6	31
111	2-Nov-15	5-Nov-15	F	15-Jun-95		Y	NA	C	8	NA	5-Nov-15	11	5
112	2-Nov-15	5-Nov-15	F	26-Jul-14			NA	C	8	NA	5-Nov-15	8	38
113	2-Nov-15	5-Nov-15	F	22-Jul-09			NA	C	8	NA	5-Nov-15	9	20
114	2-Nov-15	5-Nov-15	F	15-Jul-10			NA	C	8	NA	5-Nov-15	7	42
115	2-Nov-15	5-Nov-15	F	11-Jan-11			NA	C	8	NA	5-Nov-15	8	38
116	2-Nov-15	5-Nov-15	F	18-Feb-09			NA	C	8	NA	5-Nov-15	7	22
117	2-Nov-15	5-Nov-15	M	14-Feb-12			NA	C	8	NA	5-Nov-15	8	8
118	2-Nov-15	5-Nov-15	M	24-Mar-12			NA	C	8	NA	5-Nov-15	8	30
119	2-Nov-15	5-Nov-15	M	23-Sep-12			NA	C	8	NA	5-Nov-15	7	8
120	2-Nov-15	5-Nov-15	F	16-Mar-06			NA	C	8	NA	5-Nov-15	6	20
121	2-Nov-15	5-Nov-15	F	1-May-87			NA	C	8	NA	5-Nov-15	7	10
122	2-Nov-15	5-Nov-15	F	10-Dec-35			NA	C	8	NA	5-Nov-15	6	50
123	27-Oct-15	30-Oct-15	M	19-Mar-14			NA	C	8	NA	30-Oct-15	8	30
124	27-Oct-15	30-Oct-15	F	19-Mar-13			NA	C	8	NA	30-Oct-15	7	44
125	7-Sep-15	10-Sep-15	M	21-Aug-08			NA	C	8	NA	10-Sep-15	8	43
126	7-Sep-15	10-Sep-15	F	12-Jul-08			NA	C	8	NA	10-Sep-15	7	2
127	7-Sep-15	10-Sep-15	F	8-Feb-10			NA	C	8	NA	10-Sep-15	8	23
128	7-Sep-15	10-Sep-15	M	11-Jul-11			NA	C	8	NA	10-Sep-15	6	45
129	7-Sep-15	10-Sep-15	M	15-Dec-11			NA	C	8	NA	10-Sep-15	7	45
130	7-Sep-15	10-Sep-15	F	15-Jan-13			NA	C	8	NA	10-Sep-15	8	5
131	7-Sep-15	10-Sep-15	M	11-Aug-11			NA	C	8	NA	10-Sep-15	7	11
132	1-Sep-15	4-Sep-15	F	11-Mar-64			NA	C	8	NA	4-Sep-15	6	30
133	1-Sep-15	4-Sep-15	M	15-Jun-65			NA	C	8	NA	4-Sep-15	8	30
134	1-Sep-15	4-Sep-15	M	24-Oct-10			NA	C	8	NA	4-Sep-15	6	55
135	1-Sep-15	4-Sep-15	M	9-Aug-57	58	Y	NA	C	8	NA	4-Sep-15	9	30
136	1-Sep-15	4-Sep-15	M	19-Mar-79			NA	C	8	NA	4-Sep-15	7	26
137	1-Sep-15	4-Sep-15	M	2-Mar-11			NA	C	8	NA	4-Sep-15	6	51
138	1-Sep-15	4-Sep-15	F	6-May-11			NA	C	8	NA	4-Sep-15	8	0
139	1-Sep-15	4-Sep-15	M	11-Jul-41			NA	C	8	NA	4-Sep-15	6	28
140	31-Aug-15	3-Sep-15	F	1-Jul-98			NA	C	8	NA	3-Sep-15	8	0

141	31-Aug-15	3-Sep-15	M	6-May-01			NA	C	8	NA	3-Sep-15	7	33
142	31-Aug-15	3-Sep-15	M	26-Mar-10	5	Y	NA	C	8	NA	3-Sep-15	7	1
143	31-Aug-15	3-Sep-15	F	1-Apr-12			NA	C	8	NA	3-Sep-15	6	27
144	31-Aug-15	3-Sep-15	F	4-Feb-74	41	Y	NA	C	8	NA	3-Sep-13	8	20
145	1-Sep-15	4-Sep-15	M	4-Mar-14			NA	C	8	NA	4-Sep-15	7	41
146	20-Oct-15	23-Oct-15	F	21-Jun-85			NA	C	8	NA	23-Oct-15	6	5
147	19-Oct-15	22-Oct-15	F	19-Jul-13			NA	C	8	NA	19-Oct-15	8	10
148	19-Oct-15	22-Oct-15	F	4-Jul-12			NA	C	8	NA	22-Oct-15	7	5
149	19-Oct-15	22-Oct-15	F	13-Aug-11			NA	C	8	NA	22-Oct-15	8	53
150	19-Oct-15	22-Oct-15	M	1-Dec-12			NA	C	8	NA	22-Oct-15	8	13
151	19-Oct-15	22-Oct-15	M	3-Sep-13			NA	C	8	NA	22-Oct-15	8	33
152	19-Oct-15	22-Oct-15	F	16-Mar-07			NA	C	8	NA	22-Oct-15	6	40
153	19-Oct-15	22-Oct-15	M	18-Mar-12			NA	C	8	NA	22-Oct-15	6	27
154	19-Oct-15	22-Oct-15	F	3-Dec-13			NA	C	8	NA	22-Oct-15	7	35
155	19-Oct-15	22-Oct-15	M	7-Oct-12			NA	C	8	NA	22-Oct-15	2	27
156	19-Oct-15	22-Oct-15	M	12-Jan-11			NA	C	8	NA	22-Oct-15	7	5
157	19-Oct-15	22-Oct-15	F	27-Jun-12			NA	C	8	NA	22-Oct-15	6	32
158	13-Oct-15	16-Oct-15	F	25-Aug-08			NA	C	8	NA	16-Oct-15	10	10
159	13-Oct-15	16-Oct-15	M	6-Oct-04			NA	C	8	NA	16-Oct-15	6	20
160	13-Oct-15	16-Oct-15	M	18-Nov-04			NA	C	8	NA	16-Oct-15	1	10
161	13-Oct-15	16-Oct-15	M	19-Dec-09			NA	C	8	NA	10-Nov-15	1	25
162	13-Oct-15	16-Oct-15	M	6-Nov-06			NA	C	8	NA	16-Oct-15	7	5
163	13-Oct-15	16-Oct-15	F	17-Apr-06			NA	C	8	NA	16-Oct-15	7	25
164	13-Oct-15	16-Oct-15	M	16-Sep-13			NA	C	8	NA	16-Oct-15	8	7
165	13-Oct-15	16-Oct-15	M	17-May-08			NA	C	8	NA	16-Oct-15	6	45
166	13-Oct-15	16-Oct-15	F	14-Oct-07			NA	C	8	NA	16-Oct-15	8	28
167	12-Oct-15	15-Oct-15	F	3-Apr-13			NA	C	8	NA	15-Oct-15	7	44
168	12-Oct-15	15-Oct-15	M	2-Oct-07			NA	C	8	NA	15-Oct-15	6	45
169	12-Oct-15	15-Oct-15	M	29-Apr-09			NA	C	8	NA	15-Oct-15	7	10
170	12-Oct-15	15-Oct-15	F	11-Oct-11			NA	C	8	NA	15-Oct-15	8	9
171	12-Oct-15	15-Oct-15	F	12-Dec-13			NA	C	8	NA	15-Oct-15	7	9
172	12-Oct-15	15-Oct-15	F	11-Sep-09			NA	C	8	NA	15-Oct-15	7	29
173	12-Oct-15	15-Oct-15	F	6-Feb-06			NA	C	8	NA	12-Oct-15	7	33
174	12-Oct-15	15-Oct-15	F	14-Jul-12			NA	C	8	NA		6	53
175	12-Oct-15	15-Oct-15	M	28-Dec-10			NA	C	8	NA	15-Oct-15	6	30
176	28-Sep-15	1-Oct-15	F	15-Apr-62			NA	C	8	NA	1-Oct-15	6	20
177	22-Sep-15	25-Sep-15	F	13-Feb-12			NA	C	8	NA	25-Sep-15	7	33
178	22-Sep-15	25-Sep-15	F	5-Nov-13			NA	C	8	NA	25-Sep-15	6	36
179	22-Sep-15	25-Sep-15	F	3-Sep-11			NA	C	8	NA	25-Sep-15	6	20
180	22-Sep-15	25-Sep-15	F	12-Oct-14		M	NA	C	8	NA	25-Sep-15	6	38
181	22-Sep-15	25-Sep-15	F	14-May-15			NA	C	8	NA	25-Sep-15	7	5
182	22-Sep-15	25-Sep-15	M	22-Feb-14			NA	C	8	NA	25-Sep-15	8	16
183	22-Sep-15	25-Sep-15	F	6-Jun-59			NA	C	8	NA	25-Sep-15	8	10
184	22-Sep-15	25-Sep-15	M	29-May-09			NA	C	8	NA	25-Sep-15	7	30
185	22-Sep-15	25-Sep-15	F	2-Jan-05			NA	C	8	NA	25-Sep-15	7	12
186	15-Sep-15	18-Sep-15	F	14-Apr-13			NA	C	8	NA	18-Sep-15	8	47
187	15-Sep-15	18-Sep-15	M	11-Jun-06			NA	C	8	NA	18-Sep-15	7	43

188	15-Sep-15	18-Sep-15	M	31-Oct-10			NA	C	8	NA	18-Sep-15	7	30
189	15-Sep-15	18-Sep-15	M	29-Aug-11			NA	C	8	NA	18-Sep-15	8	22
190	15-Sep-15	18-Sep-15	F	8-Jun-03			NA	C	8	NA	18-Sep-15	8	10
191	15-Sep-15	18-Sep-15	F	31-Dec-05			NA	C	8	NA	18-Sep-15	10	1
192	15-Sep-15	18-Sep-15	M	22-Sep-08			NA	C	8	NA	18-Sep-15	7	35
193	15-Sep-15	18-Sep-15	M	5-Nov-11			NA	C	8	NA	18-Sep-15	7	1
194	15-Sep-15	18-Sep-15	F	4-Sep-14			NA	C	8	NA	18-Sep-15	9	28
195	15-Sep-15	18-Sep-15	F	10-Oct-06			NA	C	8	NA	18-Sep-15	9	15
196	15-Sep-15	18-Sep-15	F	4-Jul-09			NA	C	8	NA	18-Sep-15	7	40
197	14-Sep-15	17-Sep-15	M	15-Jul-09			NA	C	8	NA	17-Sep-15	6	43
198	14-Sep-15	17-Sep-15	F	29-Nov-11			NA	C	8	NA	17-Sep-15	11	38
199	14-Sep-15	17-Sep-15	F	16-Apr-12			NA	C	8	NA	17-Sep-15	7	30
200	14-Sep-15	17-Sep-15	M	1-Nov-08			NA	C	8	NA	17-Sep-15	7	8
201	14-Sep-15	17-Sep-15	F	12-Jan-10			NA	C	8	NA	17-Sep-15	7	30
202	22-Sep-15	25-Sep-15	F	11-Jan-81	34	Y	NA	C	8	NA	25-Sep-15	6	20
203	31-Aug-15	3-Sep-15	M	10-Jul-07			NA	C	8	NA	3-Sep-15	8	35
204	31-Aug-15	3-Sep-15	M	27-Jun-12			NA	C	8	NA	3-Sep-15	9	30
205	31-Aug-15	3-Sep-15	M	17-Nov-11			NA	C	8	NA	3-Sep-15	8	46
206	31-Aug-15	3-Sep-15	F	21-Dec-13			NA	C	8	NA	3-Sep-15	7	57
207	31-Aug-15	3-Aug-15	M	15-Jul-63			NA	C	8	NA	3-Sep-15	8	11
208	25-Aug-15	28-Aug-15	F	28-Dec-36			NA	C	8	NA	28-Aug-15	8	30
209	25-Aug-15	28-Aug-15	M	8-Aug-07			NA	C	8	NA	28-Aug-15	7	3
210	25-Aug-15	28-Aug-15	F	9-Apr-14			NA	C	8	NA	28-Aug-15	9	3
211	25-Aug-15	28-Aug-15	M	2-May-09			NA	C	8	NA	28-Aug-15	7	18
212	25-Aug-15	28-Aug-15	F	1-Nov-37		Y	NA	C	8	NA	28-Aug-15	8	10
213	25-Aug-15	28-Aug-15	F	20-Mar-07	8	Y	NA	C	8	NA	28-Aug-15	7	38
214	25-Aug-15	28-Aug-15	M	10-Aug-03			NA	C	8	NA	28-Aug-15	6	50
215	25-Aug-15	28-Aug-15	M	10-Jan-12			NA	C	8	NA	28-Aug-15	10	37
216	25-Aug-15	28-Aug-15	M	7-Jan-53			NA	C	8	NA	28-Aug-15	7	40
217	25-Aug-15	28-Aug-15	F	15-Jun-74			NA	C	8	NA	28-Aug-15	9	35
218	24-Aug-15	27-Aug-15	F	15-Jun-98			NA	C	8	NA	27-Aug-15	8	16
219	24-Aug-15	27-Aug-15	M	18-Jun-10			NA	C	8	NA	27-Aug-15	9	28
220	24-Aug-15	27-Aug-15	M	19-Mar-95			NA	C	8	NA	28-Aug-15	8	11
221	24-Aug-15	27-Aug-15	M	15-Jul-61			NA	C	8	NA	27-Aug-15	7	24
222	24-Aug-15	27-Aug-15	F	5-Jun-80			NA	C	8	NA	27-Aug-15	8	58
223	24-Aug-15	27-Aug-15	F	16-Jul-00			NA	C	8	NA	27-Aug-15	6	50
224	24-Aug-15	27-Aug-15	M	17-Oct-00			NA	C	8	NA	27-Aug-15	8	22
225	24-Aug-15	27-Aug-15	M	15-May-13			NA	C	8	NA	27-Aug-15	9	50
226	24-Aug-15	27-Aug-15	F	7-Nov-10			NA	C	8	NA	27-Aug-15	7	55
227	24-Aug-15	27-Aug-15	M	15-May-06			NA	C	8	NA	27-Aug-15	7	5
228	24-Aug-15	27-Aug-15	F	10-Jun-09			NA	C	8	NA	27-Aug-15	8	34
229	24-Aug-15	27-Aug-15	M	18-Oct-08			NA	C	8	NA	27-Aug-15	7	46
230	24-Aug-15	27-Aug-15	M	12-Feb-08			NA	C	8	NA	27-Aug-15	7	24
231	18-Aug-15	21-Aug-15	F	12-Nov-98			NA	C	8	NA	21-Aug-15	8	40
232	18-Aug-15	21-Aug-15	M	15-Aug-96			NA	C	8	NA	21-Aug-15	9	10
233	17-Aug-15	20-Aug-15	M	13-Jun-98			NA	C	8	NA	20-Aug-15	7	0
234	17-Aug-15	20-Aug-15	F	9-Dec-06			NA	C	8	NA	20-Aug-15	8	20

235	17-Aug-15	20-Aug-15	F	1-Jan-06	NA	C	8	NA	20-Aug-15	7	35
236	17-Aug-15	20-Aug-15	M	21-Jun-13	NA	C	8	NA	20-Aug-15	7	57
237	17-Aug-15	20-Aug-15	F	2-Jun-11	NA	C	8	NA	20-Aug-15	7	42
238	17-Aug-15	20-Aug-15	M	6-Nov-08	NA	C	8	NA	20-Aug-15	6	48
239	6-Oct-15	9-Oct-15	F	30-Dec-10	NA	C	8	NA	9-Oct-15	7	24
240	6-Oct-15	9-Oct-15	M	12-Jul-04	NA	C	8	NA	9-Oct-15	8	28
241	6-Oct-15	9-Oct-15	F	22-Jan-07	NA	C	8	NA	9-Oct-15	6	38
242	6-Oct-15	9-Oct-15	M	31-Aug-04	NA	C	8	NA	9-Oct-15	7	41
243	6-Oct-15	9-Oct-15	M	21-Aug-06	NA	C	8	NA	9-Oct-15	7	58
244	5-Oct-15	8-Oct-15	M	17-Apr-54	NA	C	8	NA	2-Nov-15	12	30
245	6-Oct-15	9-Oct-15	M	8-Jul-10	NA	C	8	NA	9-Oct-15	6	16
246	5-Oct-15	8-Oct-15	M	7-Nov-11	NA	C	8	NA	8-Oct-15	6	51
247	5-Oct-15	8-Oct-15	F	2-Apr-07	NA	C	8	NA	8-Oct-15	7	10
248	5-Oct-15	8-Oct-15	F	11-Apr-07	NA	C	8	NA	8-Oct-15	7	53
249	5-Oct-15	8-Oct-15	M	30-Sep-09	NA	C	8	NA	8-Oct-15	8	11
250	5-Oct-15	8-Oct-15	M	11-Jun-12	NA	C	8	NA	8-Oct-15	8	45
251	5-Oct-15	8-Oct-15	F	3-Jul-11	NA	C	8	NA	8-Oct-15	7	15
252	29-Sep-15	2-Oct-15	M	25-Oct-13	NA	C	8	NA	2-Oct-15	7	51
253	29-Sep-15	2-Oct-15	M	3-Jan-13	NA	C	8	NA	2-Oct-15	6	1
254	29-Sep-15	2-Oct-15	F	24-Mar-05	NA	C	8	NA	2-Oct-15	6	54
255	29-Sep-15	2-Oct-15	M	1-Dec-08	NA	C	8	NA	2-Oct-15	7	9
256	29-Sep-15	2-Oct-15	F	10-Aug-10	NA	C	8	NA	2-Oct-15	7	27
257	29-Sep-15	2-Oct-15	F	15-Jun-58	NA	C	8	NA	2-Oct-15	7	10
258	29-Sep-15	2-Oct-15	M	29-Jan-02	NA	C	8	NA	2-Oct-15	7	49
259	29-Sep-15	2-Oct-15	F	13-Jun-02	NA	C	8	NA	2-Oct-15	6	30
260	29-Sep-15	2-Oct-15	M	14-Jan-11	NA	C	8	NA	2-Oct-14	6	41
261	28-Sep-15	1-Oct-15	M	15-Feb-12	NA	C	8	NA	1-Oct-15	7	18
262	28-Sep-15	1-Oct-15	M	16-Feb-14	NA	C	8	NA	1-Oct-15	6	56
263	28-Sep-15	1-Oct-15	F	5-Sep-13	NA	C	8	NA	1-Oct-15	7	32
264	28-Sep-15	1-Oct-15	M	15-Oct-13	NA	C	8	NA	1-Oct-15	8	14
265	28-Sep-15	1-Oct-15	F	15-Dec-11	NA	C	8	NA	1-Oct-15	6	34
266	28-Sep-15	1-Oct-15	F	16-Mar-11	NA	C	8	NA	1-Oct-15	8	5
267	28-Sep-15	1-Oct-15	M	8-Nov-05	NA	C	8	NA	1-Oct-15	7	30
268	28-Sep-15	1-Sep-15	M	7-Jun-13	NA	C	8	NA	1-Oct-15	6	43
269	28-Sep-15	1-Oct-15	M	21-Jun-11	NA	C	8	NA	1-Oct-15	8	37
270	27-Oct-15	30-Oct-15	F	20-Aug-07	NA	C	8	NA	30-Oct-15	6	40
271	27-Oct-15	30-Oct-15	M	21-Sep-08	NA	C	8	NA	30-Oct-15	8	33
272	27-Oct-15	30-Oct-15	M	17-Apr-09	NA	C	8	NA	30-Oct-15	6	28
273	27-Oct-15	30-Oct-15	F	12-Jun-08	NA	C	8	NA	30-Oct-15	8	9
274	27-Oct-15	30-Oct-15	M	29-Jun-13	NA	C	8	NA	30-Oct-15	8	54
275	27-Oct-15	30-Oct-15	M	30-Nov-07	NA	C	8	NA	30-Oct-15	7	0
276	27-Oct-15	30-Oct-15	M	10-Jun-11	NA	C	8	NA	30-Oct-15	6	15
277	27-Oct-15	30-Oct-15	F	1-Jul-08	NA	C	8	NA	30-Oct-15	12	13
278	27-Oct-15	30-Oct-15	M	31-Jul-11	NA	C	8	NA	30-Oct-15	7	10
279	26-Oct-15	29-Oct-15	F	26-Dec-11	NA	C	8	NA	29-Oct-15	1	30
280	26-Oct-15	29-Oct-15	M	8-Apr-10	NA	C	8	NA	29-Oct-15	7	53
281	26-Oct-15	29-Oct-15	F	19-May-13	NA	C	8	NA	29-Oct-15	7	2

282	26-Oct-15	29-Oct-15	M	13-Nov-13	NA	C	8	NA	29-Oct-15	7	20
283	26-Oct-15	29-Oct-15	M	7-Dec-13	NA	C	8	NA	29-Oct-15	6	30
284	26-Oct-15	29-Oct-15	M	27-Jul-10	NA	C	8	NA	29-Oct-15	9	20
285	26-Oct-15	29-Oct-15	F	29-Oct-09	NA	C	8	NA	29-Oct-15	7	9
286	26-Oct-15	29-Oct-15	F	11-Jan-12	NA	C	8	NA	29-Oct-15	8	50
287	26-Oct-15	29-Oct-15	M	25-May-12	NA	C	8	NA	29-Oct-15	8	0
288	26-Oct-15	29-Oct-15	F	3-Aug-10	NA	C	8	NA	29-Oct-15	7	25
289	26-Oct-15	29-Oct-15	F	31-Oct-13	NA	C	8	NA	29-Oct-15	6	48
290	26-Oct-15	29-Oct-15	F	14-Jun-10	NA	C	8	NA	29-Oct-15	6	21
291	26-Oct-15	29-Oct-15	M	1-Mar-12	NA	C	8	NA	29-Oct-15	8	19
292	20-Oct-15	23-Oct-15	F	18-Apr-12	NA	C	8	NA	23-Oct-15	9	11
293	20-Oct-15	23-Oct-15	F	3-Dec-08	NA	C	8	NA	23-Oct-15	6	41
294	20-Oct-15	23-Oct-15	M	5-Jun-02	NA	C	8	NA	23-Oct-15		
295	20-Oct-15	23-Oct-15	M	2-Jan-14	NA	C	8	NA	23-Oct-15	6	6
296	20-Oct-15	23-Oct-15	F	7-Apr-10	NA	C	8	NA	23-Oct-15	7	32
297	20-Oct-15	23-Oct-15	M	9-Jul-07	NA	C	8	NA	23-Oct-15	8	19
298	20-Oct-15	23-Oct-15	F	22-May-04	NA	C	8	NA	23-Oct-15	10	10
299	20-Oct-15	23-Oct-15	F	17-May-03	NA	C	8	NA	23-Oct-15	7	10

q22	q23	q24
N	NA	DUO-COTECXIN
N	NA	DUO-COTECXIN
N	NA	DUO-COTECXIN
N	NA	DUO-COTECXIN
N	NA	DUO-COTECXIN
N	NA	DUO-COTECXIN
N	NA	DUO-COTECXIN
N	NA	DUO-COTECXIN
N	NA	DUO-COTECXIN
N	NA	DUO-COTECXIN
N	NA	DUO-COTECXIN
N	NA	DUO-COTECXIN
N	NA	DUO-COTECXIN
N	NA	DUO-COTECXIN
N	NA	DUO-COTECXIN
N	NA	DUO-COTECXIN
N	NA	DUO-COTECXIN
N	NA	DUO-COTECXIN
N	NA	DUO-COTECXIN
N	NA	DUO-COTECXIN
N	NA	DUO-COTECXIN
N	NA	DUO-COTECXIN
N	NA	DUO-COTECXIN
N	NA	DUO-COTECXIN
N	NA	DUO-COTECXIN
N	NA	DUO-COTECXIN
N	NA	DUO-COTECXIN
N	NA	DUO-COTECXIN
N	NA	DUO-COTECXIN
N	NA	DUO-COTECXIN
N	NA	DUO-COTECXIN
N	NA	DUO-COTECXIN
N	NA	DUO-COTECXIN
N	NA	DUO-COTECXIN
N	NA	DUO-COTECXIN
N	NA	DUO-COTECXIN
N	NA	DUO-CTECXIN
N	NA	DUO-COTECXIN
		DUO-COECXIN
N	NA	DUO-COTECXIN
N	NA	DUO-COTECXIN
N	NA	DUO-COTECXIN
N	NA	DUO-COTECXIN DIHYDRTEMISININ PIPERAQUINE
N	NA	DUO-COTECXIN
N	NA	DUO-COTECXIN
N	NA	DUO-COTECXIN

N	NA	DUO-COTECXIN
N	NA	DUO-COTECXIN
N	NA	DUO-COTECXIN
N	NA	DUO-COTECXIN
N	NA	DUO-COTECXIN
N	NA	DUO-COTECXIN
N	NA	DUO-COTECXIN
N	NA	DUO-COTECXIN
	DUO-CTECCXIN	
N	NA	DUO-COTECXIN
N	NA	DUO-COTECXIN
N	NA	DUO-COTECXIN
Y	DUO-COTECXIN	DUO-COTECXIN
N	NA	DUO-COTECXIN
N	NA	DUO-COTECXIN
N	NA	DUO-COTECXIN
N	NA	DUO-COTECXIN
N	NA	DUO-COTECXIN
N	NA	DUO-COTECXIN
N	NA	DUO-COTECXIN
N	NA	DUO-COTECXIN
N	NA	DUO-COTECXIN
N	NA	DUO-COTECXIN
N	NA	DUO-COTECXIN
N	NA	DUO-COTECXIN
N	NA	DUO-CTECXIN
N	NA	DUO-COTECXIN
N	NA	DUO-COTECXIN
N	NA	DUO-COTECXIN
N	NA	DUO-COTECXIN
N	NA	DUO-COTECXIN
N	NA	DUO-COTECXIN
N	NA	DUO-COTECXIN
N	NA	DUO-COTECXIN
Y	DUO-COTECXIN	DUO-COTECXIN
N	NA	DUO-COTECXIN
N	NA	DUO-COTECXIN
N	NA	DUO-COTECXIN
N	NA	DUO-COTECXIN
N	NA	DUO-COTECXIN
N	NA	DUO-COTECXIN
N	NA	DUO-COTECXIN
N	NA	DUO-COTECXIN
N	NA	DUO-COTECXIN
N	NA	DUO-COTECXIN
N	NA	DUO-COTECXIN
N	NA	DUO-COTECXIN

N	NA	DUO-COTECXIN
N	NA	DUO-COTECXIN
N	NA	DUO-COTECXIN
N	NA	DUO-COTECXIN
N	NA	DUO-COTECXIN
N	NA	DUO-COTECXIN
N	NA	DUO-COTECXIN
N	NA	DUO-COTECXIN
N	NA	DUO-COTECXIN
N	NA	DUO-COTECXIN
N	NA	DUO-COTECXIN
N	NA	DUO-COTECXIN
N	NA	DUO-COTECXIN
N	NA	DUO-COTECXIN
N	NA	DUO-COTECXIN
N	NA	DUO-COTECXIN
N	NA	DUO-COTECXIN
N	NA	DUO-COTECXIN
N	NA	DUO-COTECXIN
N	NA	DUO-COTECXIN
N	NA	DUO-COTECXIN
N	NA	DUO-COTECXIN
N	NA	DUO-COTECXIN
N	NA	DUO-COTECXIN
N	NA	DUO-COTECXIN
N	NA	DUO-COTECXIN
N	NA	DUO-COTECXIN
N	NA	DUO-COTECXIN
N	NA	DUO-COTECXIN
N	NA	DUO-COTECXIN
N	NA	DUO-COTECXIN
N	NA	DUO-COTECXIN
N	NA	DUO-COTECXIN
N	NA	DUO-COTECXIN
N	NA	DUO-COTECXIN
N	NA	DUO-COTECXIN
N	NA	DUO-COTECXIN
N	NA	DUO-COTECXIN
N	NA	DUO-COTECXIN
N	NA	DUO-COTECXIN
N	NA	DUO-COTECXIN
N	NA	DUO-COTECXIN
N	NA	DUO-COTECXIN
N	NA	DUO-COTECXIN
N	NA	DUO-COTECXIN
N	NA	DUO-COTECXIN
N	NA	DUO-COTECXIN

N	NA	DUO-COTECXIN
N	NA	DUO-COTECXIN
N	NA	DUO-COTECXIN
N	NA	DUO-COTECXIN
N	NA	DUO-COTECXIN
N	NA	DUO-COTECXIN
N	NA	DUO-COTECXIN
N	NA	DUO-COTECXIN
N	NA	DUO-COTECXIN
N	NA	DUO-COTECXIN
N	NA	DUO-COTECXIN
N	NA	DUO-COTECXIN
N	NA	DUO-COTECXIN
N	NA	DUO-COTECXIN
N	NA	DUO-COTECXIN
N	NA	DUO-COTECXIN
N	NA	DUO-COTECXIN
N	NA	DUO-COTECXIN
N	NA	DUO-COTECXIN
N	NA	DUO-COTECXIN
N	NA	DUO-COTECXIN
N	NA	DUO-COTECXIN
N	NA	DUO-COTECXIN
N	NA	DUO-COTECXIN
N	NA	DUO-COTECXIN
N	NA	DUO-COTECXIN
N	NA	DUO-COTECXIN
N	NA	DUO-COTECXIN
N	NA	DUO-COTECXIN
N	NA	DUO-COTECXIN
N	NA	DUO-COTECXIN
N	NA	DUO-COTECXIN
N	NA	DUO-COTECXIN
N	NA	DUO-COTECXIN
N	NA	DUO-COTECXIN
N	NA	DUO-COTECXIN
N	NA	DUO-COTECXIN
N	NA	DUO-COTECXIN
N	NA	DUO-COTECXIN
N	NA	DUO-COTECXIN
N	NA	DUO-COTECXIN
N	NA	DUO-COTECXIN
N	NA	DUO-COTECXIN
N	NA	DUO-COTECXIN
N	NA	DUO-COTECXIN
N	NA	DUO-COTECXIN
N	NA	DUO-COTECXIN

N	NA	DUO-COTECXIN
N	NA	DUO-COTECXIN
N	NA	DUO-COTECXIN
N	NA	DUO-COTECXIN
N	NA	DUO-COTECXIN
N	NA	DUO-COTECXIN
N	NA	DUO-COTECXIN
N	NA	DUO-COTECXIN
N	NA	DUO-COTECXIN
N	NA	DUO-COTECXIN
N	NA	DUO-COTECXIN
N	NA	DUO-COTECXIN
N	NA	DUO-COTECXIN
N	NA	DUO-COTECXIN
N	NA	DUO-COTECXIN
N	NA	DUO-COTECXIN
N	NA	DUO-COTECXIN
N	NA	DUO-COTECXIN
N	NA	DUO-COTECXIN
N	NA	DUO-COTECXIN
N	NA	DUO-COTECXIN
Y	DUO-COTECXIN	DUO-COTECXIN
N	NA	DUO-COTECXIN
N	NA	DUO-COTECXIN
N	NA	DUO-COTECXIN
N	NA	DUO-COTECXIN
N	NA	DUO-COTECXIN
N	NA	DUO-COTECXIN
N	NA	DUO-COTECXIN
N	NA	DUO-COTECXIN
N	NA	DUO-COTECXIN
N	NA	DUO-COTECXIN
N	NA	DUO-COTECXIN
N	NA	DUO-COTECXIN
N	NA	DUO-COTECXIN
N	NA	DUO-COTECXIN
N	NA	DUO-COTECXIN
N	NA	DUO-COTECXIN
N	NA	DUO-COTECXIN
N	NA	DUO-COTECXIN
N	NA	DUO-COTECXIN
N	NA	DUO-COTECXIN
N	NA	DUO-COTECXIN
N	NA	DUO-COTECXIN
N	NA	DUO-COTECXIN
N	NA	DUO-COTECXIN
N	NA	DUO-COTECXIN

N	NA	DUO-COTECXIN
N	NA	DUO-COTECXIN
N	NA	DUO-COTECXIN
N	NA	DUO-COTECXIN
N	NA	DUO-COTECXIN
N	NA	DUO-COTECXIN
N	NA	DUO-COTECXIN
N	NA	DUO-COTECXIN
N	NA	DUO-COTECXIN
N	NA	DUO-COTECXIN
N	NA	DUO-COTECXIN
N	NA	DUO-COTECXIN
N	NA	DUO-COTECXIN
N	NA	DUO-COTECXIN
N	NA	DUO-COTECXIN
N	NA	DUO-COTECXIN
N	NA	DUO-COTECXIN
N	NA	DUO-COTECXIN
N	NA	DUO-COTECXIN
N	NA	DUO-COTECXIN
N	NA	DUO-COTECXIN
N	NA	DUO-COTECXIN
N	NA	DUO-COTECXIN
N	NA	DUO-COTECXIN
N	NA	DUO-COTECXIN
N	NA	DUO-COTECXIN
N	NA	DUO-COTECXIN
N	NA	DUO-COTECXIN
N	NA	DUO-COTECXIN
N	NA	DUO-COTECXIN
N	NA	DUO-COTECXIN
N	NA	DUO-COTECXIN
N	NA	DUO-COTECXIN
N	NA	DUO-COTECXIN
N	NA	DUO-COTECXIN
N	NA	DUO-COTECXIN
N	NA	DUO-COTECXIN
N	NA	DUO-COTECXIN
N	NA	DUO-COTECXIN
N	NA	DUO-COTECXIN
N	NA	DUO-COTECXIN
N	NA	DUO-COTECXIN
N	NA	DUO-COTECXIN
N	NA	DUO-COTECXIN
N	NA	DUO-COTECXIN
N	NA	DUO-COTECXIN
		DUO-COTECXIN

N	NA	DUO-COTECXIN
N	NA	DUO-COTECXIN
N	NA	DUO-COTECXIN
N	NA	DUO-COTECXIN
N	NA	DUO-COTECXIN
N	NA	DUO-COTECXIN
N	NA	DUO-COTECXIN
N	NA	DUO-COTECXIN
N	NA	DUO-COTECXIN
N	NA	DUO-COTECXIN
N	NA	DUO-COTECXIN
N	NA	DUO-COTECXIN
N	NA	DUO-COTECXIN
N	NA	DUO-COTECXIN
N	NA	DUO-COTECXIN
N	NA	DUO-COTECXIN
N	NA	DUO-COTECXIN
N	NA	DUO-COTECXIN

q24a	q25f	q25f1	q25f2	q25f3	q26s	q26s1	q26s2	q26s3	q27n	q27n1
DIHYDROARTEMISININ PIPERAQUINE	N	8	88	88	88	88	8	8	2	88
DIHYDROARTEMISININ PIPERAQUINE	N	8	88	88	88	88	8	8	2	88
DIHYDROARTEMISIN PIPERAQUINE	N	8	88	88	88	88	8	8	3	88
DIHYDROARTEMISININ PIPERAQUINE	N	8	88	88	88	88	8	8	3	88
DIHYDROARTEMISININ PIPERAQUINE	N	8	88	88	88	88	8	8	2	88
DIHYDROARTEMISININ PIPERAQUINE	N	8	88	88	88	88	8	8	2	88
DIHDROARTEMISININ PIPERAQUINE	N	8	88	88	88	88	8	8	1	88
DIHYDROARTEMISININ PIPERAQUINE	N	8	88	88	88	88	8	8	1	88
DIHDROARTEMISININ PIPERAQUINE	N	8	88	88	88	88	8	8	2	88
DIHYDROARTEMISININ PIPERAQUINE	N	8	88	88	88	88	8	8	2	88
DIHYDROARTEMISININ PIPERAQUINE	N	8	88	88	88	88	8	8	3	88
DIHYDRARTEMISININ/PIPERAQUINE	N	8	88	88	88	88	8	8	2	88
DIHYDROARTEMISININ/PIPERAQUINE	N	8	88	88	88	88	8	8	1	88
DIHYDRARTEMISININ PIPERAQUINE	N	8	88	88	88	88	8	8	1	88
DIHYDRARTEMISININ/PIPERAQUINE	Y	8	88	88	88	88	8	8	2	88
DIHYDROARTEMISININ/PIPERAQUINE	N	8	88	88	88	88	8	8	3	88
DIHYDROARTEMISININ/PIPERAQUINE	N	8	88	88	88	88	8	8	3	88
DIHYDROARTEMISININ PIPERAQUINE	N	8	88	88	88	88	8	8	3	88
DIHYDROARTEMISININ/PIPERAQUINE	Y	8	88	88	88	88	Y	8	1	88
DIHYDROARTEMISININ/PIPERAQUINE	N	8	88	88	88	88	8	8	2	88
DIHYDROARTEMISININ/PIPERAQUINE	N	8	88	88	88	88	N	8	2	88
DIHYDROARTEMISININ/PIPERAQUINE	N	8	88	88	88	88	N	8	1	88
DIHYDROABTEMISININ/PIPERAQUINE	N	8	88	88	88	88	N	8	2	88
DIHYDROARTEMISININ/PIPERAQUINE	N	8	88	88	88	88	8	8	3	88
DIHYDROARTEMISININ PIPERAQUINE	N	8	88	88	88	88	8	8	3	88
DIHYDROARTEMISININ PIPERAQUINE	N	8	88	88	88	88	8	8	2	88
DIHYDROARTEMISININ PIPERAQUINE	N	8	88	88	88	88	8	8	1	88
DIHYDROABTEMISININ/PIPERAQUINE	N	8	88	88	88	88	8	8	2	88
DIHYDROARTEMISININ PIPERAQUINE	N	8	88	88	88	88	8	8	0	88
DIHYDROARTEMISININ PIPERAQUINE	N	8	88	88	88	88	8	8	1	88
DIHYDROARTEMISININ PIPERAQUINE	N	8	88	88	3	88	N	8	3	88
(DIHYDROARTEMISININ PIPERAQUINE)	N	8	88	88	88	88	8	8	2	88
DIHYDROARTEMISININ PIPERAQUINE	N	8	88	88	88	88	N	8	1	88
(DIHYDROARTEMISININ PIPERAQUINE)	N	8	88	88	88	88	Y	8	1	88
DIHYDROARTEMISININ PIPERAQUINE	N	8	88	88	88	88	Y	8	1	88
DIHYDROARTEMISININ PIPERAQUINE	N	8	88	88	88	88	Y	8	1	88
(DIHYDROABTEMISININ PIPERAQUINE)	N	8	88	88	88	88	N	8	1	88
DIHYDROARTEMISININ PIPERAQUINE	N	8	88	88	88	88	N	8	1	88
DIHYDROABTEMISININ PIPERAQUINE	N	8	88	88	88	88	Y	8	1	88
(DIHYDROARTEMISININ PIPERAQUINE)	N	8	88	88	88	88	Y	8	1	88
DIHYDROARTEMISININ PIPERAQUINE	N	8	88	88	88	88	8	8	1	88
DIHYDROARTEMISININ PIPERAQUINE	N	8	88	88	88	88	Y	8	1	88
	N	8	88	88	88	88	Y	8	1	88
DIHYDROARTEMISININ PIPERAQUINE	N	8	88	88	88	88	N	8	2	88
DIHYDROARTEMISININ PIPERAQUINE	N	8	88	88	88	88	8	8	1	88
DIHYDROARTEMISININ PIPERAQUINE	N	8	88	88	88	88	8	8	1	88

DIHYDROARTEMISININ PIPERAQUINE	N	8	88	88	88	88		8	8	2	88
DIHYDROARTEMISININ PIPERAQUINE	N	8	88	88	88	88	N		8	1	88
DIHYDROARTEMISININ PIPERAQUINE	N	8	88	88	88	88		8	8	3	88
DIHYDROARTEMISININ PIPERAQUINE	N	8	88	88	88	88	N		8	88	88
DIHYDROARTEMISININ PIPERAQUINE	N	8	88	88	88	88	N		8	2	88
DIHYDROARTEMISININ PIPERAQUINE	N	8	88	88	88	88		8	8	3	88
DIHYDROARTEMISININ PIPERAQUINE	N	8	88	88	88	88		8	8	2	88
DIHYDROARTEMISININ PIPERAQUINE	N	8	88	88	88	88		8	8	2	88
	N	8	88	88	88	88		8	8	3	88
DIHYDROARTEMISININ PIPERAQUINE	N	8	88	88	88	88		8	8	3	88
DIHYDROARTEMISININ PIPERAQUINE	N	8	88	88	88	88		8	8	2	88
DIHYDROARTEMISININ PIPERAQUINE	N	8	88	88	88	88		8	8	2	88
DIHYDROARTEMISININ PIPERAQUINE	N	8	88	88	88	88		8	8	2	88
DIHYDROARTEMISININ PIPERAQUINE	N	8	88	88	88	88	N		8	2	88
DIHYDROARTEMISININ PIPERAQUINE	N	8	88	88	88	88		8	8	3	88
DIHYDROARTEMISININ PIPERAQUINE	N	8	88	88	88	88	N		8	2	88
DIHYDROARTEMISININ PIPERAQUINE	N	8	88	88	88	88		8	8	2	88
DIHYDROARTEMISININ PIPERAQUINE	N	8	88	88	88	88		8	8	2	88
DIHYDROARTEMISININ PIPERAQUINE	N	8	88	88	88	88		8	8	2	88
DIHYDROARTEMISININ PIPERAQUINE	N	8	88	88	88	88		8	8	1	88
DIHYDROARTEMISININ PIPERAQUINE	N	8	88	88	88	88	N		8	1	88
DIHYDROARTEMISININ PIPERAQUINE	N	8	88	88	88	88		8	8	2	88
DIHYDROARTEMISININ PIPERAQUINE	N	8	88	88	88	88		8	8	2	88
DIHYDROARTEMISININ PIPERAQUINE	N	8	88	88	88	88	N		8	3	88
DIHYDROARTEMISIN PIPERAQUINE	N	8	88	88	88	88		8	8	2	88
DIHYDROARTEMISIN PIPERAQUINE	N	8	88	88	88	88	N		8	3	88
DIHYDROARTEMISIN PIPERAQUINE	N	8	88	88	88	88		8	8	2	88
DIHYDROARTEMISIN PIPERAQUINE	N	8	88	88	88	88		8	8	3	88
DIHYDROARTEMISIN PIPERAQUINE	N	8	88	88	88	88		8	8	2	88
DIHYDROARTEMISIN PIPERAQUINE	N	8	88	88	88	88		8	8	2	88
DIHYDROARTEMISIN PIPERAQUINE	N	8	88	88	88	88		8	8	2	88
DIHYDROARTEMISIN PIPERAQUINE	N	8	88	88	88	88		8	8	2	88
DIHYDROARTEMISIN PIPERAQUINE	N	8	88	88	88	88		8	8	3	88
DIHYDROARTEMISININ PIPERAQUINE	N	8	88	88	88	88	N		8	3	88
DIHYDROARTEMISININ PIPERAQUINE	N	8	88	88	88	88	N		8	3	88
DIHYDROARTEMISININ PIPERAQUINE	N	8	88	88	88	88	N		8	2	88
DIHYDROARTEMISININ PIPERAQUINE	N	8	88	88	88	88	N		8	2	88
DIHYDROARTEMISININ PIPERAQUINE	N	8	88	88	88	88	N		8	2	88
DIHYDROARTEMISININ PIPERAQUINE	N	8	88	88	88	88	N		8	2	88
DIHYDROARTEMISININ PIPERAQUINE	N	8	88	88	88	88	N		8	2	88
DIHYDROARTEMISININ PIPERAQUINE	N	8	88	88	88	88	Y		8	1	88
DIHYDROARTEMISININ PIPERAQUINE	N	8	88	88	88	88	N		8	3	88
DIHYDROARTEMISININ PIPERAQUINE	N	8	88	88	88	88	N		8	1	88
DIHYDROARTEMISININ PIPERAQUINE	N	8	88	88	88	88	N		8	1	88
DIHYDROARTEMISININ PIPERAQUINE	N	8	88	88	88	88	N		8	2	88
DIHYDROARTEMISININ PIPERAQUINE	N	8	88	88	88	88		8	8	2	88
DIHYDROARTEMISININ PIPERAQUINE	N	8	88	88	88	88	N		8	3	88

DIHYDROARTEMISININ PIPERAQUINE	N	8	88	88	88	88	N	8	3	88
DIHYDROARTEMISININ PIPERAQUINE	N	8	88	88	88	88	N	8	2	88
DIHYDROARTEMISININ PIPERAQUINE	N	8	88	88	88	88	N	8	3	88
DIHYDROARTEMISININ PIPERAQUINE	N	8	88	88	88	88	N	8	88	88
DIHYDROARTEMISININ PIPERAQUINE	N	8	88	88	88	88	N	8	2	88
DIHYDROARTEMISININ PIPERAQUINE	N	8	88	88	88	88	N	8	2	88
DIHYDROARTEMISININ PIPERAQUINE	N	8	88	88	88	88	N	8	2	88
DIHYDROARTEMISININ PIPERAQUINE	N	8	88	88	88	88	N	8	2	88
DIHYDROARTEMISININ PIPERAQUINE	N	8	88	88	88	88	N	8	2	88
DIHYDROARTEMISININ PIPERAQUINE	N	8	88	88	88	88	N	8	1	88
DIHYDROARTEMISININ PIPERAQUINE	N	8	88	88	88	88	N	8	2	88
DIHYDROARTEMISININ PIPERAQUINE	N	8	88	88	88	88	N	8	2	88
DIHYDROARTEMISININ PIPERAQUINE	N	8	88	88	88	88	N	8	2	88
DIHYDROARTEMISININ PIPERAQUINE	N	8	88	88	88	88	N	8	88	88
DIHYDROARTEMISININ PIPERAQUINE	N	8	88	88	88	88	N	8	3	88
DIHYDROARTEMISININ PIPERAQUINE	N	8	88	88	88	88	N	8	2	88
DIHYDROARTEMISININ PIPERAQUINE	N	8	88	88	88	88	N	8	3	88
DIHYDROARTEMISININ PIPERAQUINE	N	8	88	88	88	88	N	8	3	88
DIHYDROARTEMISININ PIPERAQUINE	N	8	88	88	88	88	N	8	2	88
DIHYDROARTEMISININ PIPERAQUINE	N	8	88	88	88	88	N	8	2	88
DIHYDROARTEMISININ PIPERAQUINE	N	8	88	88	88	88	N	8	2	88
DIHYDROARTEMISININ PIPERAQUINE	8	8	88	88	88	88	N	8	2	88
DIHYDROARTEMISININ PIPERAQUINE	N	8	88	88	88	88	N	8	2	88
DIHYDROARTEMISININ PIPERAQUINE	N	8	88	88	88	88	N	8	2	88
DIHYDROARTEMISININ PIPERAQUINE	N	8	88	88	88	88	N	8	2	88
DIHYDROARTEMISININ PIPERAQUINE	N	8	88	88	88	88	N	8	2	88
DIHYDROARTEMISININ PIPERAQUINE	N	8	88	88	88	88	N	8	2	88
DIHYDROARTEMISININ PIPERAQUINE	N	8	88	88	88	88	N	8	3	88
DIHYDROARTEMISININ PIPERAQUINE	N	8	88	88	88	88	N	8	3	88
DIHYDROARTEMISININ PIPERAQUINE	N	8	88	88	88	88	N	8	1	88
DIHYDROARTEMISININ PIPERAQUINE	N	8	88	88	88	88	8	8	1	88
DIHYDROARTEMISININ PIPERAQUINE	N	8	88	88	88	88	N	8	2	88
DIHYDROARTEMISININ PIPERAQUINE	N	8	88	88	88	88	N	8	2	88
DIHYDROARTEMISININ PIPERAQUINE	N	8	88	88	88	88	N	8	1	88
DIHYDROARTEMISININ PIPERAQUINE	N	8	88	88	88	88	N	8	1	88
DIHYDROARTEMISININ PIPERAQUINE	N	8	88	88	88	88	N	8	2	88
DIHYDROARTEMISININ PIPERAQUINE	N	8	88	88	88	88	N	8	2	88
DIHYDROARTEMISININ PIPERAQUINE	N	8	88	88	88	88	N	8	2	88
DIHYDROARTEMISININ PIPERAQUINE	N	8	88	88	88	88	N	8	3	88
DIHYDROARTEMISININ PIPERAQUINE	N	8	88	88	88	88	N	8	3	88
DIHYDROARTEMISININ PIPERAQUINE	N	8	88	88	88	88	N	8	2	88
DIHYDROARTEMISININ PIPERAQUINE	N	8	88	88	88	88	N	8	3	88
DIHYDROARTEMISININ PIPERAQUINE	N	8	88	88	88	88	N	8	3	88
DIHYDROARTEMISININ PIPERAQUINE	N	8	88	88	88	88	N	8	2	88
DIHYDROARTEMISININ PIPERAQUINE	N	8	88	88	88	88	N	8	2	88
DIHYDROARTEMISININ PIPERAQUINE	N	8	88	88	88	88	N	8	3	88
DIHYDROARTEMISININ PIPERAQUINE	N	8	88	88	88	88	N	8	3	88

DIHYDROARTEMISININ PIPERAQUINE	N	8	88	88	88	88	N		8	3	88
DIHYDROARTEMISININ PIPERAQUINE	N	8	88	88	88	88	N		8	1	88
DIHYDROARTEMISININ PIPERAQUINE	N	8	88	88	88	88	N		8	1	88
DIHYDROARTEMISININ PIPERAQUINE	N	8	88	88	88	88	N		8	88	88
DIHYDROARTEMISININ PIPERAQUINE	N	8	88	88	88	88	N		8	1	88
DIHYDROARTEMISININ PIPERAQUINE	N	8	88	88	88	88	N		8	3	88
DIHYDROARTEMISININ PIPERAQUINE	N	8	88	88	88	88	N		8	2	88
DIHYDROARTEMISININ PIPERAQUINE	N	8	88	88	88	88	N		8	2	88
DIHYDROARTEMISININ PIPERAQUINE	N	8	88	88	88	88	N		8	2	88
DIHYDROARTEMISININ PIPERAQUINE	N	8	88	88	88	88	N		8	2	88
DIHYDROARTEMISININ PIPERAQUINE	N	8	88	88	88	88	N		8	1	88
DIHYDROARTEMISININ PIPERAQUINE	N	8	88	88	88	88	N		8	2	88
DIHYDROARTEMISININ PIPERAQUINE	N	8	88	88	88	88	N		8	2	88
DIHYDROARTEMISININ PIPERAQUINE	N	8	88	88	88	88	N		8	2	88
DIHYDROARTEMISININ PIPERAQUINE	N	8	88	88	88	88	N		8	2	88
DIHYDROARTEMISININ PIPERAQUINE	N	8	88	88	88	88	N		8	2	88
DIHYDROARTEMISININ PIPERAQUINE	N	8	88	88	88	88	N		8	2	88
DIHYDROARTEMISININ PIPERAQUINE	N	8	88	88	88	88	N		8	2	88
DIHYDROARTEMISININ PIPERAQUINE	N	8	88	88	88	88	N		8	2	88
DIHYDROARTEMISININ PIPERAQUINE	N	8	88	88	88	88	N		8	2	88
DIHYDROARTEMISININ PIPERAQUINE	N	8	88	88	88	88	N		8	2	88
DIHYDROARTEMISININ PIPERAQUINE	N	8	88	88	88	88	N		8	88	88
DIHYDROARTEMISININ PIPERAQUINE	N	8	88	88	88	88	N		8	2	88
DIHYDROARTEMISININ PIPERAQUINE	N	8	88	88	88	88	N		8	2	88
DIHYDROARTEMISININ PIPERAQUINE	N	8	88	88	88	88	N		8	2	88
DIHYDROARTEMISININ PIPERAQUINE	N	8	88	88	88	88	N		8	2	88
DIHYDROARTEMISININ PIPERAQUINE	N	8	88	88	88	88	N		8	1	88
DIHYDROARTEMISININ PIPERAQUINE	N	8	88	88	88	88	N		8	1	88
DIHYDROARTEMISININ PIPERAQUINE	N	8	88	88	88	88	N		8	2	88
DIHYDROARTEMISININ PIPERAQUINE	N	8	88	88	88	88		8	8	1	88
DIHYDROARTEMISININ PIPERAQUINE	N	8	88	88	88	88	N		8	1	88
DIHYDROARTEMISININ PIPERAQUINE	N	8	88	88	88	88	N		8	2	88
DIHYDROARTEMISININ PIPERAQUINE	N	8	88	88	88	88	N		8	2	88
DIHYDROARTEMISININ PIPERAQUINE	N	8	88	88	88	88	N		8	2	88
DIHYDROARTEMISININ PIPERAQUINE	N	8	88	88	88	88	N		8	2	88
DIHYDROARTEMISININ PIPERAQUINE	N	8	88	88	88	88	N		8	3	88
DIHYDROARTEMISININ PIPERAQUINE	N	8	88	88	88	88	N		8	2	88
DIHYDROARTEMISININ PIPERAQUINE	N	8	88	88	88	88	N		8	1	88
DIHYDROARTEMISININ PIPERAQUINE	N	8	88	88	88	88	N		8	2	88
DIHYDROARTEMISININ PIPERAQUINE	N	8	88	88	88	88	N		8	1	88
DIHYDROARTEMISININ PIPERAQUINE	N	8	88	88	88	88	N		8	2	88
DIHYDROARTEMISININ PIPERAQUINE	N	8	88	88	88	88	N		8	1	88
DIHYDROARTEMISININ PIPERAQUINE	N	8	88	88	88	88	N		8	3	88
DIHYDROARTEMISININ PIPERAQUINE	N	8	88	88	88	88	N		8	2	88
DIHYDROARTEMISININ PIPERAQUINE	N	8	88	88	88	88	N		8	2	88
DIHYDROARTEMISININ PIPERAQUINE	N	8	88	88	88	88	N		8	2	88
DIHYDROARTEMISININ PIPERAQUINE	N	8	88	88	88	88	N		8	2	88

DIHYDROARTEMISININ PIPERAQUINE	N	8	88	88	88	88	N		8	2	88
DIHYDROARTEMISININ PIPERAQUINE	N	8	88	88	88	88	N		8	2	88
DIHYDROARTEMISININ PIPERAQUINE	N	8	88	88	88	88	N		8	3	88
DIHYDROARTEMISININ PIPERAQUINE	N	8	88	88	88	88	N		8	2	88
DIHYDROARTEMISININ PIPERAQUINE	N	8	88	88	88	88	N		8	2	88
DIHYDROARTEMISININ PIPERAQUINE	N	8	88	88	88	88	N		8	2	88
DIHYDROARTEMISININ PIPERAQUINE	N	8	88	88	88	88	N		8	1	88
DIHYDROARTEMISININ PIPERAQUINE	N	8	88	88	88	88	N		8	2	88
DIHYDROARTEMISININ PIPERAQUINE	N	8	88	88	88	88	N		8	2	88
DIHYDROARTEMISININ PIPERAQUINE	N	8	88	88	88	88	N		8	2	88
DIHYDROARTEMISININ PIPERAQUINE	N	8	88	88	88	88	N		8	2	88
DIHYDROARTEMISININ PIPERAQUINE	N	8	88	88	88	88	N		8	2	88
DIHYDROARTEMISININ PIPERAQUINE	N	8	88	88	88	88	N		8	1	88
DIHYDROARTEMISININ PIPERAQUINE	N	8	88	88	88	88	N		8	2	88
DIHYDROARTEMISININ PIPERAQUINE	N	8	88	88	88	88	N		8	3	88
DIHYDROARTEMISININ PIPERAQUINE	Y	8	88	88	88	88	N		8	2	88
DIHYDROARTEMISININ PIPERAQUINE	Y	8	88	88	88	88	N		8	2	88
DIHYDROARTEMISININ PIPERAQUINE	Y	8	88	88	88	88	N		8	2	88
DIHYDROARTEMISININ PIPERAQUINE	Y	8	88	88	88	88	N		8	1	88
DIHYDROARTEMISININ PIPERAQUINE	Y	8	88	88	88	88	N		8	3	88
DIHYDROARTEMISININ PIPERAQUINE	N	8	88	88	88	88	N		8	2	88
DIHYDROARTEMISININ PIPERAQUINE	N	8	88	88	88	88	N		8	2	88
DIHYDROARTEMISININ PIPERAQUINE	N	8	88	88	88	88	N		8	1	88
DIHYDROARTEMISININ PIPERAQUINE	N	8	88	88	88	88	N		8	2	88
DIHYDROARTEMISININ PIPERAQUINE	N	8	88	88	88	88	N		8	3	88
DIHYDROARTEMISININ PIPERAQUINE	N	8	88	88	88	88	N		8	2	88
DIHYDROARTEMISININ PIPERAQUINE	N	8	88	88	88	88	N		8	2	88
DIHYDROARTEMISININ PIPERAQUINE	Y	8	88	88	88	88	N		8	2	88
DIHYDROARTEMISININ PIPERAQUINE	N	8	88	88	88	88	N		8	3	88
DIHYDROARTEMISININ PIPERAQUINE	N	8	88	88	88	88	N		8	3	88
DIHYDROARTEMISININ PIPERAQUINE	N	8	88	88	88	88	N		8	3	88
DIHYDROARTEMISININ PIPERAQUINE	N	8	88	88	88	88	N		8	2	88
DIHYDROARTEMISININ PIPERAQUINE	N	8	88	88	88	88	N		8	3	88
DIHYDROARTEMISININ PIPERAQUINE	N	8	88	88	88	88	N		8	3	88
DIHYDROARTEMISININ PIPERAQUINE	N	8	88	88	88	88	N		8	3	88
DIHYDROARTEMISININ PIPERAQUINE	N	8	88	88	88	88	N		8	3	88
DIHYDROARTEMISININ PIPERAQUINE	N	8	88	88	88	88	N		8	3	88
DIHYDROARTEMISININ PIPERAQUINE	N	8	88	88	88	88		8	8	88	88
DIHYDROARTEMISININ PIPERAQUINE	Y	8	88	88	88	88	N		8	2	88
DIHYDROARTEMISININ PIPERAQUINE	N	8	88	88	88	88	N		8	2	88
DIHYDROARTEMISININ PIPERAQUINE	N	8	88	88	88	88	N		8	2	88
DIHYDROARTEMISININ PIPERAQUINE	N	8	88	88	88	88	N		8	2	88
DIHYDROARTEMISININ PIPERAQUINE	N	8	88	88	88	88	N		8	2	88
DIHYDROARTEMISININ PIPERAQUINE	N	8	88	88	88	88	Y		8	1	88
DIHYDROARTEMISININ PIPERAQUINE	N	8	88	88	88	88	N		8	3	88
DIHYDROARTEMISININ PIPERAQUINE	N	8	88	88	88	88	N		8	3	88
DIHYDROARTEMISININ PIPERAQUINE	N	8	88	88	88	88	N		8	2	88

DIHYDROARTEMISININ PIPERAQUINE	N	8	88	88	88	88	N	8	2	88
DIHYDROARTEMISININ PIPERAQUINE	N	8	88	88	88	88	N	8	1	88
DIHYDROARTEMISININ PIPERAQUINE	N	8	88	88	88	88	N	8	1	88
DIHYDROARTEMISININ PIPERAQUINE	N	8	88	88	88	88	N	8	2	88
DIHYDROARTEMISININ PIPERAQUINE	N	8	88	88	88	88	N	8	2	88
DIHYDROARTEMISININ PIPERAQUINE	N	8	88	88	88	88	N	8	2	88
DIHYDROARTEMISININ PIPERAQUINE	N	8	88	88	88	88	8	8	88	88
DIHYDROARTEMISININ PIPERAQUINE	N	8	88	88	88	88	N	8	2	88
DIHYDROARTEMISININ PIPERAQUINE	N	8	88	88	88	88	N	8	88	88
DIHYDROARTEMISININ PIPERAQUINE	N	8	88	88	88	88	N	8	3	88
DIHYDROARTEMISININ PIPERAQUINE	N	8	88	88	88	88	N	8	2	88
DIHYDROARTEMISININ PIPERAQUINE	N	8	88	88	88	88	N	8	1	88
DIHYDROARTEMISININ PIPERAQUINE	N	8	88	88	88	88	N	8	2	88
DIHYDROARTEMISININ PIPERAQUINE	N	8	88	88	88	88	N	8	2	88
DIHYDROARTEMISININ PIPERAQUINE	N	8	88	88	88	88	N	8	2	88
DIHYDROARTEMISININ PIPERAQUINE	N	8	88	88	88	88	N	8	2	88
DIHYDROARTEMISININ PIPERAQUINE	N	8	88	88	88	88	N	8	2	88
DIHYDROARTEMISININ PIPERAQUINE	N	8	88	88	88	88	N	8	2	88
DIHYDROARTEMISININ PIPERAQUINE	N	8	88	88	88	88	N	8	2	88
DIHYDROARTEMISININ PIPERAQUINE	N	8	88	88	88	88	N	8	2	88
DIHYDROARTEMISININ PIPERAQUINE	N	8	88	88	88	88	N	8	2	88
DIHYDROARTEMISININ PIPERAQUINE	N	8	88	88	88	88	N	8	2	88
DIHYDROARTEMISININ PIPERAQUINE	N	8	88	88	88	88	N	8	3	88
DIHYDROARTEMISININ PIPERAQUINE	N	8	88	88	88	88	N	8	2	88
DIHYDROARTEMISININ PIPERAQUINE	N	8	88	88	88	88	N	8	2	88
DIHYDROARTEMISININ PIPERAQUINE	N	8	88	88	88	88	N	8	2	88
DIHYDROARTEMISININ PIPERAQUINE	N	8	88	88	88	88	N	8	2	88
DIHYDROARTEMISININ PIPERAQUINE	N	8	88	88	88	88	N	8	1	88
DIHYDROARTEMISININ PIPERAQUINE	N	8	88	88	88	88	N	8	88	88
DIHYDROARTEMISININ PIPERAQUINE	N	8	88	88	88	88	N	8	2	88
DIHYDROARTEMISININ PIPERAQUINE	8	8	88	88	88	88	N	8	2	88
DIHYDROARTEMISININ PIPERAQUINE	N	8	88	88	88	88	N	8	2	88
DIHYDROARTEMISININ PIPERAQUINE	N	8	88	88	88	88	N	8	88	88
DIHYDROARTEMISININ PIPERAQUINE	N	8	88	88	88	88	N	8	2	88
DIHYDROARTEMISININ PIPERAQUINE	Y	8	88	88	88	88	Y	8	1	88
DIHYDROARTEMISININ PIPERAQUINE	N	8	88	88	88	88	N	8	1	88
DIHYDROARTEMISININ PIPERAQUINE	N	8	88	88	88	88	8	8	88	88
DIHYDROARTEMISININ PIPERAQUINE	N	8	88	88	88	88	N	8	2	88
DIHYDROARTEMISININ PIPERAQUINE	N	8	88	88	88	88	N	8	2	88
DIHYDROARTEMISININ PIPERAQUINE	N	8	88	88	88	88	N	8	2	88
DIHYDROARTEMISININ PIPERAQUINE	N	8	88	88	88	88	N	8	1	88
DIHYDROARTEMISININ PIPERAQUINE	N	8	88	88	88	88	N	8	1	88
DIHYDROARTEMISININ PIPERAQUINE	N	8	88	88	88	88	N	8	1	88
DIHYDROARTEMISININ PIPERAQUINE	N	8	88	88	88	88	N	8	2	88
DIHYDROARTEMISININ PIPERAQUINE	N	8	88	88	88	88	N	8	2	88
DIHYDROARTEMISININ PIPERAQUINE	N	8	88	88	88	88	N	8	2	88
DIHYDROARTEMISININ PIPERAQUINE	N	8	88	88	88	88	N	8	2	88

DIHYDROARTEMISININ PIPERAQUINE	N	8	88	88	88	88	N	8	1	88
DIHYDROARTEMISININ PIPERAQUINE	N	8	88	88	88	88	N	8	2	88
DIHYDROARTEMISININ PIPERAQUINE	N	8	88	88	88	88	N	8	2	88
DIHYDROARTEMISININ PIPERAQUINE	N	8	88	88	88	88	N	8	2	88
DIHYDROARTEMISININ PIPERAQUINE	N	8	88	88	88	88	N	8	2	88
DIHYDROARTEMISININ PIPERAQUINE	N	8	88	88	88	88	N	8	2	88
DIHYDROARTEMISININ PIPERAQUINE	N	8	88	88	88	88	N	8	2	88
DIHYDROARTEMISININ PIPERAQUINE	N	8	88	88	88	88	N	8	2	88
DIHYDROARTEMISININ PIPERAQUINE	N	8	88	88	88	88	N	8	2	88
DIHYDROARTEMISININ PIPERAQUINE	N	8	88	88	88	88	N	8	2	88
DIHYDROARTEMISININ PIPERAQUINE	Y	8	88	88	88	88	N	8	1	88
DIHYDROARTEMISININ PIPERAQUINE	N	8	88	88	88	88	N	8	2	88
DIHYDROARTEMISININ PIPERAQUINE	N	8	88	88	88	88	N	8	2	88
DIHYDROARTEMISININ PIPERAQUINE	N	8	88	88	88	88	N	8	1	88
DIHYDROARTEMISININ PIPERAQUINE	N	8	88	88	88	88	N	8	2	88
DIHYDROARTEMISININ PIPERAQUINE	N	8	88	88	88	88	N	8	2	88
DIHYDROARTEMISININ PIPERAQUINE	N	8	88	88	88	88	N	8	2	88
DIHYDROARTEMISININ PIPERAQUINE	N	8	88	88	88	88	N	8	2	88

q27n2	q27n3	q28t	q28t1	q28t2	q28t3	q29t	q29t1	q29t2	q29t3	q30t	q30t1	q30t2	q30t3	q31v	q31v1
88	88	Ev	88	88	88	Ye	88	88	88	No	88	88	88	No	88
88	88	Af	88	88	88	Ye	88	88	88	No	88	88	88	No	88
88	88	Ev	88	88	88	Ye	88	88	88	No	88	88	88	No	88
88	88	Af	88	88	88	Ye	88	88	88	No	88	88	88	No	88
88	88	Af	88	88	88	Ye	88	88	88	No	88	88	88	Ye	88
88	88	Ev	88	88	88	Ye	88	88	88	No	88	88	88	No	88
1	88	Ev	88	Ev	88	Ye	88	Ye	88	No	88	No	88	No	88
88	88	Ev	88	88	88	Ye	88	88	88	No	88	88	88	No	88
88	88	Af	88	88	88	Ye	88	88	88	No	88	88	88	Ye	88
88	88	Ev	88	88	88	Ye	88	88	88	No	88	88	88	No	88
88	88	Ev	88	88	88	Ye	88	88	88	No	88	88	88	No	88
88	88	Ev	88	88	88	Ye	88	88	88	No	88	88	88	No	88
88	88	Ev	88	88	88	Ye	88	88	88	No	88	88	88	No	88
88	88	Ev	88	88	88	Ye	88	88	88	No	88	88	88	No	88
88	88	Af	88	88	88	Ye	88	88	88	No	88	88	88	No	88
88	88	Af	88	88	88	Ye	88	88	88	Ye	88	88	88	No	88
88	88	Ev	88	88	88	Ye	88	88	88	No	88	88	88	No	88
88	88	Ev	88	88	88	Ye	88	88	88	No	88	88	88	No	88
1	88	Af	88	Ev	88	Ye	88	Ye	88	No	88	No	88	No	88
88	88	Ev	88	88	88	Ye	88	88	88	No	88	88	88	No	88
88	88	Ev	88	88	88	Ye	88	88	88	No	88	88	88	No	88
88	88	Ev	88	88	88	Ye	88	88	88	No	88	88	88	No	88
88	88	Ev	88	88	88	Ye	88	88	88	No	88	88	88	No	88
88	88	Ev	88	88	88	Ye	88	88	88	No	88	88	88	No	88
88	88	Ev	88	88	88	Ye	88	88	88	No	88	88	88	No	88
88	88	Ev	88	88	88	Ye	88	88	88	No	88	88	88	No	88
88	88	Af	88	88	88	Ye	88	88	88	No	88	88	88	No	88
88	88	Ev	88	88	88	Ye	88	88	88	No	88	88	88	No	88
88	88	88	88	88	88	88	88	88	88	88	88	88	88	88	88
88	88	Af	88	88	88	Ye	88	88	88	No	88	88	88	No	88
88	88	Ev	88	88	88	Ye	88	88	88	Do	88	88	88	No	88
88	88	Af	88	88	88	Ye	88	88	88	No	88	88	88	No	88
88	88	Ev	88	88	88	Ye	88	88	88	No	88	88	88	Ye	88
1	88	Mo	88	Ev	88	Ye	88	Ye	88	No	88	No	88	No	88
1	88	Mo	88	Ev	88	Ye	88	Ye	88	No	88	No	88	No	88
1	88	Af	88	Ev	88	Ye	88	Ye	88	No	88	No	88	No	88
88	88	Ev	88	88	88	Ye	88	88	88	No	88	88	88	No	88
88	88	Ev	88	88	88	Ye	88	88	88	No	88	88	88	Ye	88
1	88	Af	88	Ev	88	Ye	88	Ye	88	No	88	Ye	88	No	88
1	88	Mo	88	Ev	88	Ye	88	Ye	88	No	88	No	88	No	88
88	88	Ev	88	88	88	Ye	88	88	88	No	88	88	88	No	88
1	88	Mo	88	Ev	88	Ye	88	Ye	88	No	88	No	88	No	88
1	88	Mo	88	Ev	88	Ye	88	Ye	88	No	88	No	88	No	88
88	88	Ev	88	88	88	Ye	88	88	88	No	88	88	88	No	88
88	88	Ev	88	88	88	Ye	88	88	88	Ye	88	88	88	Ye	88
88	88	Ev	88	88	88	Ye	88	88	88	No	88	88	88	No	88

88	88	Af	88	88	88	Ye	88	88	88	No	88	88	88	No	88
88	88	Mo	88	88	88	Ye	88	88	88	Ye	88	88	88	No	88
88	88	Mo	88	88	88	Ye	88	88	88	No	88	88	88	No	88
88	88	88	88	88	88	88	88	88	88	88	88	88	88	88	88
88	88	Ev	88	88	88	Ye	88	88	88	No	88	88	88	No	88
88	88	Ev	88	88	88	Ye	88	88	88	No	88	88	88	No	88
88	88	Ev	88	88	88	Ye	88	88	88	No	88	88	88	No	88
88	88	Ev	88	88	88	Ye	88	88	88	No	88	88	88	No	88
88	88	Ev	88	88	88	Ye	88	88	88	No	88	88	88	Ye	88
88	88	Ev	88	88	88	Ye	88	88	88	No	88	88	88	No	88
88	88	Ev	88	88	88	Ye	88	88	88	No	88	88	88	No	88
88	88	Mo	88	88	88	Ye	88	88	88	No	88	88	88	No	88
88	88	Ev	88	88	88	Ye	88	88	88	No	88	88	88	No	88
88	88	Mo	88	88	88	Ye	88	88	88	No	88	88	88	No	88
88	88	Ev	88	88	88	Ye	88	88	88	No	88	88	88	No	88
88	88	Mo	88	88	88	Ye	88	88	88	No	88	88	88	No	88
88	88	Ev	88	88	88	Ye	88	88	88	Ye	88	88	88	Ye	88
88	88	Ev	88	88	88	Ye	88	88	88	No	88	88	88	No	88
88	88	Ev	88	88	88	Ye	88	88	88	Ye	88	88	88	No	88
1	88	Mo	88 Ev		88	Ye	88 Ye		88	No	88 No		88	No	88
88	88	Ev	88	88	88	Ye	88	88	88	Ye	88	88	88	Ye	88
88	88	Ev	88	88	88	Ye	88	88	88	No	88	88	88	Ye	88
88	88	Ev	88	88	88	Ye	88	88	88	No	88	88	88	No	88
88	88	Ev	88	88	88	Ye	88	88	88	No	88	88	88	No	88
88	88	Ev	88	88	88	Ye	88	88	88	No	88	88	88	No	88
88	88	Mo	88	88	88	Ye	88	88	88	No	88	88	88	No	88
88	88	Mo	88	88	88	Ye	88	88	88	No	88	88	88	No	88
88	88	Ev	88	88	88	Ye	88	88	88	No	88	88	88	No	88
88	88	Ev	88	88	88	Ye	88	88	88	No	88	88	88	Ye	88
88	88	Mo	88	88	88	Ye	88	88	88	Ye	88	88	88	No	88
88	88	Ev	88	88	88	Ye	88	88	88	No	88	88	88	No	88
88	88	Ev	88	88	88	Ye	88	88	88	No	88	88	88	No	88
88	88	Ev	88	88	88	Ye	88	88	88	No	88	88	88	No	88
88	88	Af	88	88	88	Ye	88	88	88	No	88	88	88	No	88
88	88	Af	88	88	88	Ye	88	88	88	No	88	88	88	No	88
88	88	Af	88	88	88	Ye	88	88	88	No	88	88	88	No	88
88	88	Mo	88	88	88	Ye	88	88	88	No	88	88	88	No	88
88	88	Ev	88	88	88	Ye	88	88	88	No	88	88	88	Ye	88
88	88	Ev	88	88	88	Ye	88	88	88	No	88	88	88	No	88
88	88	Mo	88	88	88	Ye	88	88	88	Ye	88	88	88	No	88
1	88	Mo	88 Ev		88	Ye	88 Ye		88	No	88 No		88	No	88
88	88	Mo	88	88	88	Ye	88	88	88	No	88	88	88	No	88
88	88	Ev	88	88	88	Ye	88	88	88	Ye	88	88	88	Ye	88
88	88	Ev	88	88	88	Ye	88	88	88	Ye	88	88	88	Ye	88
88	88	Af	88	88	88	Ye	88	88	88	No	88	88	88	No	88
88	88	Af	88	88	88	Ye	88	88	88	No	88	88	88	No	88
88	88	Af	88	88	88	Ye	88	88	88	No	88	88	88	No	88

88	88 Af	88	88	88 Ye	88	88	88 No	88	88	88 No	88
88	88 Mo	88	88	88 Ye	88	88	88 No	88	88	88 No	88
88	88 Ev	88	88	88 Ye	88	88	88 No	88	88	88 Ye	88
88	88 88	88	88	88 88	88	88	88 88	88	88	88 88	88
88	88 Af	88	88	88 Ye	88	88	88 No	88	88	88 No	88
88	88 Ev	88	88	88 Ye	88	88	88 No	88	88	88 No	88
88	88 Ev	88	88	88 Ye	88	88	88 No	88	88	88 No	88
88	88 Ev	88	88	88 Ye	88	88	88 No	88	88	88 No	88
88	88 Af	88	88	88 Ye	88	88	88 No	88	88	88 No	88
88	88 Ev	88	88	88 Ye	88	88	88 No	88	88	88 No	88
88	88 Ev	88	88	88 Ye	88	88	88 No	88	88	88 No	88
88	88 Af	88	88	88 Ye	88	88	88 No	88	88	88 No	88
88	88 Af	88	88	88 Ye	88	88	88 No	88	88	88 No	88
88	88 88	88	88	88 88	88	88	88 88	88	88	88 88	88
88	88 Mo	88	88	88 Ye	88	88	88 No	88	88	88 No	88
88	88 Mo	88	88	88 Ye	88	88	88 No	88	88	88 No	88
88	88 Af	88	88	88 Ye	88	88	88 No	88	88	88 No	88
88	88 Af	88	88	88 Ye	88	88	88 No	88	88	88 No	88
88	88 Af	88	88	88 Ye	88	88	88 Ye	88	88	88 No	88
88	88 Mo	88	88	88 Ye	88	88	88 No	88	88	88 No	88
88	88 Ev	88	88	88 Ye	88	88	88 No	88	88	88 Ye	88
88	88 Ev	88	88	88 Ye	88	88	88 No	88	88	88 No	88
88	88 Ev	88	88	88 Ye	88	88	88 No	88	88	88 No	88
88	88 Ev	88	88	88 Ye	88	88	88 No	88	88	88 Ye	88
88	88 Ev	88	88	88 Ye	88	88	88 No	88	88	88 No	88
88	88 Ev	88	88	88 Ye	88	88	88 No	88	88	88 Ye	88
88	88 Af	88	88	88 Ye	88	88	88 No	88	88	88 No	88
88	88 Mo	88	88	88 Ye	88	88	88 No	88	88	88 No	88
88	88 Mo	88	88	88 Ye	88	88	88 No	88	88	88 No	88
88	88 Af	88	88	88 Ye	88	88	88 No	88	88	88 No	88
88	88 Ev	88	88	88 Ye	88	88	88 No	88	88	88 No	88
88	88 Ev	88	88	88 Ye	88	88	88 No	88	88	88 Ye	88
88	88 Af	88	88	88 Ye	88	88	88 No	88	88	88 Ye	88
88	88 Af	88	88	88 Ye	88	88	88 No	88	88	88 No	88
88	88 Ev	88	88	88 Ye	88	88	88 No	88	88	88 No	88
88	88 Af	88	88	88 Ye	88	88	88 No	88	88	88 No	88
88	88 Mo	88	88	88 Ye	88	88	88 No	88	88	88 No	88
88	88 Mo	88	88	88 Ye	88	88	88 No	88	88	88 No	88
88	88 Af	88	88	88 Ye	88	88	88 No	88	88	88 No	88
88	88 Af	88	88	88 Ye	88	88	88 No	88	88	88 No	88
88	88 Af	88	88	88 Ye	88	88	88 No	88	88	88 No	88
88	88 Ev	88	88	88 Ye	88	88	88 No	88	88	88 No	88
88	88 Af	88	88	88 Ye	88	88	88 No	88	88	88 No	88
88	88 Ev	88	88	88 Ye	88	88	88 No	88	88	88 Ye	88
88	88 Af	88	88	88 Ye	88	88	88 No	88	88	88 No	88
88	88 Af	88	88	88 Ye	88	88	88 No	88	88	88 No	88
88	88 Ev	88	88	88 Ye	88	88	88 No	88	88	88 No	88

88	88 Af	88	88	88 Ye	88	88	88 No	88	88	88 No	88
88	88 Ev	88	88	88 Ye	88	88	88 No	88	88	88 No	88
88	88 Af	88	88	88 Ye	88	88	88 No	88	88	88 No	88
88	88 88	88	88	88 88	88	88	88 88	88	88	88 88	88
88	88 Af	88	88	88 Ye	88	88	88 Ye	88	88	88 No	88
88	88 Mo	88	88	88 Ye	88	88	88 No	88	88	88 No	88
88	88 Mo	88	88	88 Ye	88	88	88 No	88	88	88 No	88
88	88 Af	88	88	88 Ye	88	88	88 No	88	88	88 No	88
88	88 Ev	88	88	88 Ye	88	88	88 No	88	88	88 No	88
88	88 Ev	88	88	88 Ye	88	88	88 No	88	88	88 No	88
88	88 Ev	88	88	88 Ye	88	88	88 No	88	88	88 Ye	88
88	88 Ev	88	88	88 Ye	88	88	88 No	88	88	88 No	88
88	88 Ev	88	88	88 Ye	88	88	88 No	88	88	88 No	88
88	88 Mo	88	88	88 Ye	88	88	88 No	88	88	88 Ye	88
88	88 Ev	88	88	88 Ye	88	88	88 Ye	88	88	88 No	88
88	88 Ev	88	88	88 Ye	88	88	88 No	88	88	88 No	88
88	88 Mo	88	88	88 Ye	88	88	88 No	88	88	88 No	88
88	88 Mo	88	88	88 Ye	88	88	88 No	88	88	88 No	88
88	88 Ev	88	88	88 Ye	88	88	88 No	88	88	88 No	88
88	88 Ev	88	88	88 Ye	88	88	88 No	88	88	88 No	88
88	88 Ev	88	88	88 Ye	88	88	88 No	88	88	88 No	88
88	88 88	88	88	88 88	88	88	88 88	88	88	88 88	88
88	88 Ev	88	88	88 Ye	88	88	88 No	88	88	88 Ye	88
88	88 Ev	88	88	88 Ye	88	88	88 No	88	88	88 Ye	88
88	88 Af	88	88	88 Ye	88	88	88 No	88	88	88 No	88
88	88 Ev	88	88	88 Ye	88	88	88 No	88	88	88 No	88
88	88 Af	88	88	88 Ye	88	88	88 No	88	88	88 No	88
88	88 Af	88	88	88 Ye	88	88	88 No	88	88	88 No	88
88	88 Af	88	88	88 Ye	88	88	88 No	88	88	88 No	88
88	88 Ev	88	88	88 Ye	88	88	88 No	88	88	88 No	88
88	88 Af	88	88	88 Ye	88	88	88 Ye	88	88	88 Ye	88
88	88 Ev	88	88	88 Ye	88	88	88 No	88	88	88 No	88
88	88 Mo	88	88	88 Ye	88	88	88 No	88	88	88 No	88
88	88 Mo	88	88	88 Ye	88	88	88 No	88	88	88 No	88
88	88 Mo	88	88	88 Ye	88	88	88 No	88	88	88 No	88
88	88 Ev	88	88	88 Ye	88	88	88 No	88	88	88 No	88
88	88 Ev	88	88	88 Ye	88	88	88 No	88	88	88 No	88
88	88 Af	88	88	88 Ye	88	88	88 No	88	88	88 No	88
88	88 Af	88	88	88 Ye	88	88	88 No	88	88	88 No	88
88	88 Ev	88	88	88 Ye	88	88	88 Ye	88	88	88 No	88
88	88 Mo	88	88	88 Ye	88	88	88 No	88	88	88 No	88
88	88 Af	88	88	88 Ye	88	88	88 Ye	88	88	88 No	88
88	88 Af	88	88	88 Ye	88	88	88 No	88	88	88 No	88
88	88 Af	88	88	88 Ye	88	88	88 No	88	88	88 Ye	88
88	88 Mo	88	88	88 Ye	88	88	88 No	88	88	88 No	88
88	88 Af	88	88	88 Ye	88	88	88 Ye	88	88	88 No	88
88	88 Ev	88	88	88 Ye	88	88	88 No	88	88	88 No	88

88	88	Af	88		88	88	Ye	88		88	88	No	88		88	88	No	88
88	88	Af	88		88	88	Ye	88		88	88	No	88		88	88	Ye	88
88	88	Ev	88		88	88	Ye	88		88	88	No	88		88	88	No	88
88	88	Ev	88		88	88	Ye	88		88	88	No	88		88	88	No	88
88	88	Ev	88		88	88	Ye	88		88	88	No	88		88	88	No	88
88	88	Ev	88		88	88	Ye	88		88	88	88	88		88	88	No	88
88	88	Mo	88		88	88	Ye	88		88	88	Ye	88		88	88	No	88
88	88	Mo	88		88	88	Ye	88		88	88	No	88		88	88	No	88
88	88	Af	88		88	88	Ye	88		88	88	No	88		88	88	No	88
88	88	Af	88		88	88	Ye	88		88	88	No	88		88	88	No	88
88	88	Af	88		88	88	Ye	88		88	88	No	88		88	88	No	88
88	88	Af	88		88	88	Ye	88		88	88	No	88		88	88	Ye	88
88	88	Af	88		88	88	Ye	88		88	88	No	88		88	88	No	88
88	88	Af	88		88	88	Ye	88		88	88	No	88		88	88	No	88
88	88	Ev	88		88	88	Ye	88		88	88	No	88		88	88	No	88
88	88	Mo	88		88	88	Ye	88		88	88	No	88		88	88	No	88
88	88	Af	88		88	88	Ye	88		88	88	No	88		88	88	Ye	88
88	88	Af	88		88	88	Ye	88		88	88	No	88		88	88	No	88
88	88	Mo	88		88	88	Ye	88		88	88	Ye	88		88	88	No	88
88	88	Mo	88		88	88	Ye	88		88	88	No	88		88	88	No	88
88	88	Ev	88		88	88	No	88		88	88	No	88		88	88	No	88
88	88	Ev	88		88	88	Ye	88		88	88	No	88		88	88	No	88
88	88	Mo	88		88	88	Ye	88		88	88	Ye	88		88	88	No	88
88	88	Af	88		88	88	Ye	88		88	88	No	88		88	88	No	88
88	88	Af	88		88	88	Ye	88		88	88	No	88		88	88	No	88
88	88	Af	88		88	88	Ye	88		88	88	No	88		88	88	No	88
88	88	Af	88		88	88	Ye	88		88	88	No	88		88	88	No	88
88	88	Mo	88		88	88	Ye	88		88	88	No	88		88	88	No	88
88	88	Af	88		88	88	Ye	88		88	88	No	88		88	88	No	88
88	88	Mo	88		88	88	Ye	88		88	88	No	88		88	88	No	88
88	88	Ev	88		88	88	Ye	88		88	88	No	88		88	88	No	88
88	88	Ev	88		88	88	Ye	88		88	88	No	88		88	88	No	88
88	88	Mo	88		88	88	Ye	88		88	88	No	88		88	88	No	88
88	88	Af	88		88	88	Ye	88		88	88	No	88		88	88	No	88
88	88	Af	88		88	88	Ye	88		88	88	No	88		88	88	No	88
88	88	Ev	88		88	88	Ye	88		88	88	No	88		88	88	No	88
88	88	Ev	88		88	88	Ye	88		88	88	No	88		88	88	No	88
88	88	88	88		88	88	88	88		88	88	88	88		88	88	88	88
88	88	Mo	88		88	88	Ye	88		88	88	No	88		88	88	No	88
88	88	Af	88		88	88	Ye	88		88	88	No	88		88	88	No	88
88	88	Af	88		88	88	Ye	88		88	88	No	88		88	88	No	88
88	88	Af	88		88	88	No	88		88	88	No	88		88	88	No	88
88	88	Af	88		88	88	Ye	88		88	88	No	88		88	88	Ye	88
1	88	Af	88	Ev		88	Ye	88	Ye		88	No	88	No		88	No	88
88	88	Ev	88		88	88	Ye	88		88	88	No	88		88	88	No	88
88	88	Ev	88		88	88	Ye	88		88	88	No	88		88	88	No	88
88	88	Ev	88		88	88	Ye	88		88	88	No	88		88	88	No	88

88	88	Ev	88	88	88	Ye	88	88	88	No	88	88	88	No	88
88	88	Mo	88	88	88	Ye	88	88	88	Ye	88	88	88	No	88
88	88	Af	88	88	88	Ye	88	88	88	No	88	88	88	No	88
88	88	Ev	88	88	88	Ye	88	88	88	No	88	88	88	Ye	88
88	88	Af	88	88	88	Ye	88	88	88	No	88	88	88	No	88
88	88	Ev	88	88	88	Ye	88	88	88	No	88	88	88	No	88
88	88	88	88	88	88	88	88	88	88	88	88	88	88	88	88
88	88	Af	88	88	88	Ye	88	88	88	No	88	88	88	No	88
88	88	88	88	88	88	88	88	88	88	88	88	88	88	88	88
88	88	Ev	88	88	88	Ye	88	88	88	No	88	88	88	No	88
88	88	Ev	88	88	88	Ye	88	88	88	No	88	88	88	Ye	88
88	88	Ev	88	88	88	Ye	88	88	88	No	88	88	88	No	88
88	88	Ev	88	88	88	Ye	88	88	88	No	88	88	88	No	88
88	88	Ev	88	88	88	Ye	88	88	88	No	88	88	88	No	88
88	88	Ev	88	88	88	Ye	88	88	88	No	88	88	88	No	88
88	88	Ev	88	88	88	Ye	88	88	88	No	88	88	88	No	88
88	88	Ev	88	88	88	Ye	88	88	88	No	88	88	88	No	88
88	88	Ev	88	88	88	Ye	88	88	88	No	88	88	88	No	88
88	88	Ev	88	88	88	Ye	88	88	88	No	88	88	88	Ye	88
88	88	Ev	88	88	88	Ye	88	88	88	No	88	88	88	No	88
88	88	Af	88	88	88	Ye	88	88	88	No	88	88	88	No	88
88	88	Af	88	88	88	Ye	88	88	88	No	88	88	88	No	88
88	88	Af	88	88	88	Ye	88	88	88	No	88	88	88	No	88
88	88	Af	88	88	88	Ye	88	88	88	No	88	88	88	Ye	88
88	88	Ev	88	88	88	Ye	88	88	88	No	88	88	88	No	88
88	88	Af	88	88	88	No	88	88	88	No	88	88	88	No	88
88	88	Ev	88	88	88	Ye	88	88	88	No	88	88	88	No	88
88	88	Ev	88	88	88	Ye	88	88	88	Ye	88	88	88	No	88
88	88	88	88	88	88	88	88	88	88	88	88	88	88	88	88
88	88	Mo	88	88	88	Ye	88	88	88	Ye	88	88	88	Ye	88
88	88	Ev	88	88	88	Ye	88	88	88	No	88	88	88	No	88
88	88	Mo	88	88	88	Ye	88	88	88	No	88	88	88	No	88
88	88	88	88	88	88	88	88	88	88	88	88	88	88	88	88
88	88	Mo	88	88	88	Ye	88	88	88	No	88	88	88	No	88
1	88	Mo	88	Ev	88	Ye	88	Ye	88	No	88	No	88	No	88
88	88	Ev	88	88	88	Ye	88	88	88	No	88	88	88	Ye	88
88	88	88	88	88	88	88	88	88	88	88	88	88	88	88	88
88	88	Ev	88	88	88	Ye	88	88	88	No	88	88	88	No	88
88	88	Ev	88	88	88	Ye	88	88	88	No	88	88	88	No	88
88	88	Ev	88	88	88	Ye	88	88	88	No	88	88	88	No	88
88	88	Ev	88	88	88	Ye	88	88	88	No	88	88	88	Ye	88
88	88	Ev	88	88	88	Ye	88	88	88	No	88	88	88	No	88
88	88	Af	88	88	88	Ye	88	88	88	No	88	88	88	No	88
88	88	Af	88	88	88	Ye	88	88	88	No	88	88	88	No	88
88	88	Af	88	88	88	Ye	88	88	88	No	88	88	88	No	88
88	88	Af	88	88	88	Ye	88	88	88	No	88	88	88	No	88
88	88	Af	88	88	88	Ye	88	88	88	No	88	88	88	No	88

88	88	Af	88	88	88	Ye	88	88	88	Ye	88	88	88	No	88
88	88	Mo	88	88	88	Ye	88	88	88	No	88	88	88	No	88
88	88	Ev	88	88	88	Ye	88	88	88	No	88	88	88	No	88
88	88	Af	88	88	88	Ye	88	88	88	No	88	88	88	No	88
88	88	Af	88	88	88	Ye	88	88	88	No	88	88	88	No	88
88	88	Af	88	88	88	Ye	88	88	88	No	88	88	88	No	88
88	88	Af	88	88	88	Ye	88	88	88	No	88	88	88	No	88
88	88	Ev	88	88	88	Ye	88	88	88	Ye	88	88	88	No	88
88	88	Af	88	88	88	Ye	88	88	88	No	88	88	88	No	88
88	88	Af	88	88	88	Ye	88	88	88	No	88	88	88	No	88
1	88	Af	88	Ev	88	Ye	88	Ye	88	No	88	No	88	Ye	88
88	88	Ev	88	88	88	Ye	88	88	88	No	88	88	88	No	88
88	88	Ev	88	88	88	Ye	88	88	88	No	88	88	88	Ye	88
88	88	Ev	88	88	88	Ye	88	88	88	No	88	88	88	Ye	88
88	88	Ev	88	88	88	Ye	88	88	88	No	88	88	88	No	88
88	88	Ev	88	88	88	Ye	88	88	88	No	88	88	88	No	88
88	88	Mo	88	88	88	Ye	88	88	88	No	88	88	88	No	88
88	88	Ev	88	88	88	Ye	88	88	88	No	88	88	88	No	88

q31v2	q31v3	q32i	q32i1	q32i2	q32i3	q33r	q33r1	q33r2	q33r3	q33anti	q33anti1	q33anti2	q33anti3
88	88	88	88	88	88	88	88	88	88	NA	NA	NA	NA
88	88	88	88	88	88	88	88	88	88	NA	NA	NA	NA
88	88	88	88	88	88	88	88	88	88	NA	NA	NA	NA
88	88	88	88	88	88	88	88	88	88	NA	NA	NA	NA
88	88	No	88	88	88	88	88	88	88	NA	NA	NA	NA
88	88	88	88	88	88	88	88	88	88	NA	NA	NA	NA
88	88	88	88	88	88	88	88	88	88	NA	NA	NA	NA
88	88	88	88	88	88	88	88	88	88	NA	NA	NA	NA
88	88	No	88	88	88	88	88	88	88	NA	NA	NA	NA
88	88	88	88	88	88	88	88	88	88	NA	NA	NA	NA
88	88	88	88	88	88	88	88	88	88	NA	NA	NA	NA
88	88	88	88	88	88	88	88	88	88	NA	NA	NA	NA
88	88	88	88	88	88	88	88	88	88	NA	NA	NA	NA
88	88	88	88	88	88	88	88	88	88	NA	NA	NA	NA
88	88	88	88	88	88	88	88	88	88	NA	NA	NA	NA
88	88	88	88	88	88	88	88	88	88	NA	NA	NA	NA
88	88	88	88	88	88	88	88	88	88	NA	NA	NA	NA
88	88	88	88	88	88	88	88	88	88	NA	NA	NA	NA
88	88	88	88	88	88	88	88	88	88	NA	NA	NA	NA
88	88	88	88	88	88	88	88	88	88	NA	NA	NA	NA
88	88	88	88	88	88	88	88	88	88	NA	NA	NA	NA
88	88	88	88	88	88	88	88	88	88	NA	NA	NA	NA
88	88	88	88	88	88	88	88	88	88	NA	NA	NA	NA
88	88	88	88	88	88	88	88	88	88	NA	NA	NA	NA
88	88	88	88	88	88	88	88	88	88	NA	NA	NA	NA
88	88	88	88	88	88	88	88	88	88	NA	NA	NA	NA
88	88	88	88	88	88	88	88	88	88	NA	NA	NA	NA
88	88	88	88	88	88	88	88	88	88	NA	NA	NA	NA
88	88	88	88	88	88	ot	88	88	88	NA	NA	NA	NA
88	88	88	88	88	88	88	88	88	88	NA	NA	NA	NA
88	88	88	88	88	88	88	88	88	88	NA	NA	NA	NA
88	88	88	88	88	88	88	88	88	88	NA	NA	NA	NA
88	88	No	88	88	88	88	88	88	88	NA	NA	NA	NA
88	88	88	88	88	88	88	88	88	88	NA	NA	NA	NA
88	88	88	88	88	88	88	88	88	88	NA	NA	NA	NA
88	88	88	88	88	88	88	88	88	88	NA	NA	NA	NA
88	88	88	88	88	88	88	88	88	88	NA	NA	NA	NA
88	88	No	88	88	88	88	88	88	88	NA	NA	NA	NA
88	88	88	88	88	88	88	88	Fo	88	NA	NA	NA	NA
88	88	88	88	88	88	88	88	88	88	NA	NA	NA	NA
88	88	88	88	88	88	88	88	88	88	NA	NA	NA	NA
88	88	88	88	88	88	88	88	88	88	NA	NA	NA	NA
88	88	88	88	88	88	88	88	88	88	NA	NA	NA	NA
88	88	88	88	88	88	88	88	88	88	NA	NA	NA	NA
88	88	No	88	88	88	88	88	88	88	NA	NA	NA	NA
88	88	88	88	88	88	88	88	88	88	NA	NA	NA	NA

88	88	88	88	88	88	88	88	88	88	NA	NA	NA	NA
88	88	88	88	88	88	88	88	88	88	NA	NA	NA	NA
88	88	88	88	88	88	88	88	88	88	NA	NA	NA	NA
88	88	88	88	88	88	Do	88	88	88	NA	NA	NA	NA
88	88	88	88	88	88	88	88	88	88	NA	NA	NA	NA
88	88	88	88	88	88	88	88	88	88	NA	NA	NA	NA
88	88	88	88	88	88	88	88	88	88	NA	NA	NA	NA
88	88	88	88	88	88	88	88	88	88	NA	NA	NA	NA
88	88	No	88	88	88	88	88	88	88	NA	NA	NA	NA
88	88	88	88	88	88	88	88	88	88	NA	NA	NA	NA
88	88	88	88	88	88	88	88	88	88	NA	NA	NA	NA
88	88	88	88	88	88	88	88	88	88	NA	NA	NA	NA
88	88	88	88	88	88	88	88	88	88	NA	NA	NA	NA
88	88	88	88	88	88	88	88	88	88	NA	NA	NA	NA
88	88	88	88	88	88	88	88	88	88	NA	NA	NA	NA
88	88	88	88	88	88	88	88	88	88	NA	NA	NA	NA
88	88	Ye	88	88	88	88	88	88	88	NA	NA	NA	NA
88	88	88	88	88	88	88	88	88	88	NA	NA	NA	NA
88	88	88	88	88	88	88	88	88	88	NA	NA	NA	NA
88	88	88	88	88	88	88	88	88	88	NA	NA	NA	NA
88	88	No	88	88	88	88	88	88	88	NA	NA	NA	NA
88	88	No	88	88	88	88	88	88	88	NA	NA	NA	NA
88	88	88	88	88	88	88	88	88	88	NA	NA	NA	NA
88	88	88	88	88	88	88	88	88	88	NA	NA	NA	NA
88	88	88	88	88	88	88	88	88	88	NA	NA	NA	NA
88	88	88	88	88	88	88	88	88	88	NA	NA	NA	NA
88	88	88	88	88	88	88	88	88	88	NA	NA	NA	NA
88	88	88	88	88	88	88	88	88	88	NA	NA	NA	NA
88	88	Ye	88	88	88	88	88	88	88	NA	NA	NA	NA
88	88	88	88	88	88	88	88	88	88	NA	NA	NA	NA
88	88	88	88	88	88	88	88	88	88	NA	NA	NA	NA
88	88	88	88	88	88	88	88	88	88	NA	NA	NA	NA
88	88	88	88	88	88	88	88	88	88	NA	NA	NA	NA
88	88	88	88	88	88	88	88	88	88	NA	NA	NA	NA
88	88	88	88	88	88	88	88	88	88	NA	NA	NA	NA
88	88	88	88	88	88	88	88	88	88	NA	NA	NA	NA
88	88	88	88	88	88	88	88	88	88	NA	NA	NA	NA
88	88	No	88	88	88	88	88	88	88	NA	NA	NA	NA
88	88	88	88	88	88	88	88	88	88	NA	NA	NA	NA
88	88	88	88	88	88	88	88	88	88	NA	NA	NA	NA
88	88	88	88	88	88	88	88	88	88	NA	NA	NA	NA
88	88	88	88	88	88	88	88	88	88	NA	NA	NA	NA
88	88	Ye	88	88	88	88	88	88	88	NA	NA	NA	NA
88	88	Ye	88	88	88	88	88	88	88	NA	NA	NA	NA
88	88	88	88	88	88	88	88	88	88	NA	NA	NA	NA
88	88	88	88	88	88	88	88	88	88	NA	NA	NA	NA
88	88	88	88	88	88	88	88	88	88	NA	NA	NA	NA

88	88	88	88	88	88	88	88	88	88	NA	NA	NA	NA
88	88	88	88	88	88	88	88	88	88	NA	NA	NA	NA
88	88	No	88	88	88	88	88	88	88	NA	NA	NA	NA
88	88	88	88	88	88	Si	88	88	88	NA	NA	NA	NA
88	88	88	88	88	88	88	88	88	88	NA	NA	NA	NA
88	88	88	88	88	88	88	88	88	88	NA	NA	NA	NA
88	88	88	88	88	88	88	88	88	88	NA	NA	NA	NA
88	88	88	88	88	88	88	88	88	88	NA	NA	NA	NA
88	88	88	88	88	88	88	88	88	88	NA	NA	NA	NA
88	88	88	88	88	88	88	88	88	88	NA	NA	NA	NA
88	88	88	88	88	88	88	88	88	88	NA	NA	NA	NA
88	88	88	88	88	88	88	88	88	88	NA	NA	NA	NA
88	88	88	88	88	88	88	88	88	88	NA	NA	NA	NA
88	88	88	88	88	88	Do	88	88	88	NA	NA	NA	NA
88	88	88	88	88	88	88	88	88	88	NA	NA	NA	NA
88	88	88	88	88	88	88	88	88	88	NA	NA	NA	NA
88	88	88	88	88	88	88	88	88	88	NA	NA	NA	NA
88	88	88	88	88	88	88	88	88	88	NA	NA	NA	NA
88	88	88	88	88	88	88	88	88	88	NA	NA	NA	NA
88	88	88	88	88	88	88	88	88	88	NA	NA	NA	NA
88	88	No	88	88	88	88	88	88	88	NA	NA	NA	NA
88	88	88	88	88	88	88	88	88	88	NA	NA	NA	NA
88	88	88	88	88	88	88	88	88	88	NA	NA	NA	NA
88	88	No	88	88	88	88	88	88	88	NA	NA	NA	NA
88	88	88	88	88	88	88	88	88	88	NA	NA	NA	NA
88	88	No	88	88	88	88	88	88	88	NA	NA	NA	NA
88	88	88	88	88	88	88	88	88	88	NA	NA	NA	NA
88	88	88	88	88	88	88	88	88	88	NA	NA	NA	NA
88	88	88	88	88	88	88	88	88	88	NA	NA	NA	NA
88	88	88	88	88	88	88	88	88	88	NA	NA	NA	NA
88	88	88	88	88	88	88	88	88	88	NA	NA	NA	NA
88	88	No	88	88	88	88	88	88	88	NA	NA	NA	NA
88	88	No	88	88	88	88	88	88	88	NA	NA	NA	NA
88	88	88	88	88	88	88	88	88	88	NA	NA	NA	NA
88	88	88	88	88	88	88	88	88	88	NA	NA	NA	NA
88	88	88	88	88	88	88	88	88	88	NA	NA	NA	NA
88	88	88	88	88	88	88	88	88	88	NA	NA	NA	NA
88	88	88	88	88	88	88	88	88	88	NA	NA	NA	NA
88	88	88	88	88	88	88	88	88	88	NA	NA	NA	NA
88	88	88	88	88	88	88	88	88	88	NA	NA	NA	NA
88	88	88	88	88	88	88	88	88	88	NA	NA	NA	NA
88	88	88	88	88	88	88	88	88	88	NA	NA	NA	NA
88	88	88	88	88	88	88	88	88	88	NA	NA	NA	NA
88	88	Ye	88	88	88	88	88	88	88	NA	NA	NA	NA
88	88	88	88	88	88	88	88	88	88	NA	NA	NA	NA
88	88	88	88	88	88	88	88	88	88	NA	NA	NA	NA
88	88	88	88	88	88	88	88	88	88	NA	NA	NA	NA

88	88	88	88	88	88	88	88	88	88	NA	NA	NA	NA
88	88	88	88	88	88	88	88	88	88	NA	NA	NA	NA
88	88	88	88	88	88	88	88	88	88	NA	NA	NA	NA
88	88	88	88	88	88	88	88	88	88	NA	NA	NA	NA
88	88	88	88	88	88	88	88	88	88	NA	NA	NA	NA
88	88	88	88	88	88	88	88	88	88	NA	NA	NA	NA
88	88	88	88	88	88	88	88	88	88	NA	NA	NA	NA
88	88	88	88	88	88	88	88	88	88	NA	NA	NA	NA
88	88	88	88	88	88	88	88	88	88	NA	NA	NA	NA
88	88	88	88	88	88	88	88	88	88	NA	NA	NA	NA
88	88	Ye	88	88	88	88	88	88	88	NA	NA	NA	NA
88	88	88	88	88	88	88	88	88	88	NA	NA	NA	NA
88	88	88	88	88	88	88	88	88	88	NA	NA	NA	NA
88	88	No	88	88	88	88	88	88	88	NA	NA	NA	NA
88	88	88	88	88	88	88	88	88	88	NA	NA	NA	NA
88	88	88	88	88	88	88	88	88	88	NA	NA	NA	NA
88	88	88	88	88	88	88	88	88	88	NA	NA	NA	NA
88	88	88	88	88	88	88	88	88	88	NA	NA	NA	NA
88	88	88	88	88	88	88	88	88	88	NA	NA	NA	NA
88	88	88	88	88	88	88	88	88	88	NA	NA	NA	NA
88	88	88	88	88	88	88	88	88	88	NA	NA	NA	NA
88	88	88	88	88	88	88	88	88	88	NA	NA	NA	NA
88	88	No	88	88	88	88	88	88	88	NA	NA	NA	NA
88	88	No	88	88	88	88	88	88	88	NA	NA	NA	NA
88	88	88	88	88	88	88	88	88	88	NA	NA	NA	NA
88	88	88	88	88	88	88	88	88	88	NA	NA	NA	NA
88	88	88	88	88	88	88	88	88	88	NA	NA	NA	NA
88	88	88	88	88	88	88	88	88	88	NA	NA	NA	NA
88	88	88	88	88	88	88	88	88	88	NA	NA	NA	NA
88	88	88	88	88	88	88	88	88	88	NA	NA	NA	NA
88	88	No	88	88	88	88	88	88	88	NA	NA	NA	NA
88	88	88	88	88	88	88	88	88	88	NA	NA	NA	NA
88	88	88	88	88	88	88	88	88	88	NA	NA	NA	NA
88	88	88	88	88	88	88	88	88	88	NA	NA	NA	NA
88	88	88	88	88	88	88	88	88	88	NA	NA	NA	NA
88	88	88	88	88	88	88	88	88	88	NA	NA	NA	NA
88	88	88	88	88	88	88	88	88	88	NA	NA	NA	NA
88	88	88	88	88	88	88	88	88	88	NA	NA	NA	NA
88	88	88	88	88	88	88	88	88	88	NA	NA	NA	NA
88	88	88	88	88	88	88	88	88	88	NA	NA	NA	NA
88	88	88	88	88	88	88	88	88	88	NA	NA	NA	NA
88	88	88	88	88	88	88	88	88	88	NA	NA	NA	NA
88	88	88	88	88	88	88	88	88	88	NA	NA	NA	NA
88	88	No	88	88	88	88	88	88	88	NA	NA	NA	NA
88	88	88	88	88	88	88	88	88	88	NA	NA	NA	NA
88	88	88	88	88	88	88	88	88	88	NA	NA	NA	NA
88	88	88	88	88	88	88	88	88	88	NA	NA	NA	NA

88	88	88	88	88	88	88	88	88	88	NA	NA	NA	NA
88	88	No	88	88	88	88	88	88	88	NA	NA	NA	NA
88	88	88	88	88	88	88	88	88	88	NA	NA	NA	NA
88	88	88	88	88	88	88	88	88	88	NA	NA	NA	NA
88	88	88	88	88	88	88	88	88	88	NA	NA	NA	NA
88	88	88	88	88	88	88	88	88	88	NA	NA	NA	NA
88	88	88	88	88	88	88	88	88	88	NA	NA	NA	NA
88	88	88	88	88	88	88	88	88	88	NA	NA	NA	NA
88	88	88	88	88	88	88	88	88	88	NA	NA	NA	NA
88	88	88	88	88	88	88	88	88	88	NA	NA	NA	NA
88	88	88	88	88	88	88	88	88	88	NA	NA	NA	NA
88	88	No	88	88	88	88	88	88	88	NA	NA	NA	NA
88	88	88	88	88	88	88	88	88	88	NA	NA	NA	NA
88	88	88	88	88	88	88	88	88	88	NA	NA	NA	NA
88	88	88	88	88	88	88	88	88	88	NA	NA	NA	NA
88	88	88	88	88	88	88	88	88	88	NA	NA	NA	NA
88	88	No	88	88	88	88	88	88	88	NA	NA	NA	NA
88	88	88	88	88	88	88	88	88	88	NA	NA	NA	NA
88	88	88	88	88	88	88	88	88	88	NA	NA	NA	NA
88	88	88	88	88	88	88	88	88	88	NA	NA	NA	NA
88	88	88	88	88	88	88	88	88	88	NA	NA	NA	NA
88	88	88	88	88	88	88	88	88	88	NA	NA	NA	NA
88	88	88	88	88	88	88	88	88	88	NA	NA	NA	NA
88	88	88	88	88	88	88	88	88	88	NA	NA	NA	NA
88	88	88	88	88	88	88	88	88	88	NA	NA	NA	NA
88	88	88	88	88	88	88	88	88	88	NA	NA	NA	NA
88	88	88	88	88	88	88	88	88	88	NA	NA	NA	NA
88	88	88	88	88	88	88	88	88	88	NA	NA	NA	NA
88	88	88	88	88	88	88	88	88	88	NA	NA	NA	NA
88	88	88	88	88	88	88	88	88	88	NA	NA	NA	NA
88	88	88	88	88	88	88	88	88	88	NA	NA	NA	NA
88	88	88	88	88	88	88	88	88	88	NA	NA	NA	NA
88	88	88	88	88	88	88	88	88	88	NA	NA	NA	NA
88	88	88	88	88	88	88	88	88	88	NA	NA	NA	NA
88	88	88	88	88	88	88	88	88	88	NA	NA	NA	NA
88	88	88	88	88	88	88	88	88	88	NA	NA	NA	NA
88	88	88	88	88	88	88	88	88	88	NA	NA	NA	NA
88	88	88	88	88	88	Do	88	88	88	NA	NA	NA	NA
88	88	88	88	88	88	88	88	88	88	NA	NA	NA	NA
88	88	88	88	88	88	88	88	88	88	NA	NA	NA	NA
88	88	88	88	88	88	88	88	88	88	NA	NA	NA	NA
88	88	88	88	88	88	88	88	88	88	NA	NA	NA	NA
88	88	No	88	88	88	88	88	88	88	NA	NA	NA	NA
88	88	88	88	88	88	88	88	88	88	NA	NA	NA	NA
88	88	88	88	88	88	88	88	88	88	NA	NA	NA	NA
88	88	88	88	88	88	88	88	88	88	NA	NA	NA	NA
88	88	88	88	88	88	88	88	88	88	NA	NA	NA	NA

88	88	88	88	88	88	88	88	88	88	NA	NA	NA	NA
88	88	88	88	88	88	88	88	88	88	NA	NA	NA	NA
88	88	88	88	88	88	88	88	88	88	NA	NA	NA	NA
88	88	No	88	88	88	88	88	88	88	NA	NA	NA	NA
88	88	88	88	88	88	88	88	88	88	NA	NA	NA	NA
88	88	88	88	88	88	88	88	88	88	NA	NA	NA	NA
88	88	88	88	88	88	88	88	88	88	NA	NA	NA	NA
88	88	88	88	88	88	88	88	88	88	NA	NA	NA	NA
88	88	88	88	88	88	88	88	88	88	NA	NA	NA	NA
88	88	88	88	88	88	88	88	88	88	NA	NA	NA	NA
88	88	No	88	88	88	88	88	88	88	NA	NA	NA	NA
88	88	88	88	88	88	88	88	88	88	NA	NA	NA	NA
88	88	88	88	88	88	88	88	88	88	NA	NA	NA	NA
88	88	88	88	88	88	88	88	88	88	NA	NA	NA	NA
88	88	88	88	88	88	88	88	88	88	NA	NA	NA	NA
88	88	88	88	88	88	88	88	88	88	NA	NA	NA	NA
88	88	88	88	88	88	88	88	88	88	NA	NA	NA	NA
88	88	88	88	88	88	88	88	88	88	NA	NA	NA	NA
88	88	No	88	88	88	88	88	88	88	NA	NA	NA	NA
88	88	88	88	88	88	88	88	88	88	NA	NA	NA	NA
88	88	88	88	88	88	88	88	88	88	NA	NA	NA	NA
88	88	88	88	88	88	88	88	88	88	NA	NA	NA	NA
88	88	88	88	88	88	88	88	88	88	NA	NA	NA	NA
88	88	No	88	88	88	88	88	88	88	NA	NA	NA	NA
88	88	88	88	88	88	88	88	88	88	NA	NA	NA	NA
88	88	88	88	88	88	88	88	88	88	NA	NA	NA	NA
88	88	88	88	88	88	88	88	88	88	NA	NA	NA	NA
88	88	88	88	88	88	88	88	88	88	NA	NA	NA	NA
88	88	88	88	88	88	88	88	88	88	NA	NA	NA	NA
88	88	Ye	88	88	88	88	88	88	88	NA	NA	NA	NA
88	88	88	88	88	88	88	88	88	88	NA	NA	NA	NA
88	88	88	88	88	88	88	88	88	88	NA	NA	NA	NA
88	88	88	88	88	88	Do	88	88	88	NA	NA	NA	NA
88	88	88	88	88	88	88	88	88	88	NA	NA	NA	NA
88	88	88	88	88	88	88	88	88	88	NA	NA	NA	NA
88	88	88	88	88	88	88	88	88	88	NA	NA	NA	NA
88	88	88	88	88	88	88	88	88	88	NA	NA	NA	NA
88	88	88	88	88	88	88	88	88	88	NA	NA	NA	NA
88	88	88	88	88	88	88	88	88	88	NA	NA	NA	NA
88	88	88	88	88	88	88	88	88	88	NA	NA	NA	NA
88	88	No	88	88	88	88	88	88	88	NA	NA	NA	NA
88	88	88	88	88	88	88	88	88	88	NA	NA	NA	NA
88	88	88	88	88	88	88	88	88	88	NA	NA	NA	NA
88	88	88	88	88	88	88	88	88	88	NA	NA	NA	NA
88	88	88	88	88	88	88	88	88	88	NA	NA	NA	NA
88	88	88	88	88	88	88	88	88	88	NA	NA	NA	NA
88	88	Ye	88	88	88	88	88	88	88	NA	NA	NA	NA

	88	88	88	88	88	88	88	88	88	88	NA	NA	NA	NA
	88	88	88	88	88	88	88	88	88	88	NA	NA	NA	NA
	88	88	88	88	88	88	88	88	88	88	NA	NA	NA	NA
	88	88	88	88	88	88	88	88	88	88	NA	NA	NA	NA
	88	88	88	88	88	88	88	88	88	88	NA	NA	NA	NA
	88	88	88	88	88	88	88	88	88	88	NA	NA	NA	NA
	88	88	88	88	88	88	88	88	88	88	NA	NA	NA	NA
	88	88	88	88	88	88	88	88	88	88	NA	NA	NA	NA
	88	88	88	88	88	88	88	88	88	88	NA	NA	NA	NA
	88	88	88	88	88	88	88	88	88	88	NA	NA	NA	NA
Ye		88	No	88	No	88	88	88	88	88	NA	NA	NA	NA
	88	88	88	88	88	88	88	88	88	88	NA	NA	NA	NA
	88	88	No	88	88	88	88	88	88	88	NA	NA	NA	NA
	88	88	No	88	88	88	88	88	88	88	NA	NA	NA	NA
	88	88	88	88	88	88	88	88	88	88	NA	NA	NA	NA
	88	88	88	88	88	88	88	88	88	88	NA	NA	NA	NA
	88	88	88	88	88	88	88	88	88	88	NA	NA	NA	NA
	88	88	88	88	88	88	88	88	88	88	NA	NA	NA	NA

q33other	q33other1	q33other2	q33other3	q34	q35i	q35i1	q35i2	q35i3	q36d	q36d1
NA	NA	NA	NA	88	Mo	NA	NA	NA	Y	8
NA	NA	NA	NA	88	Ot	NA	NA	NA	Y	8
NA	NA	NA	NA	88	NA	NA	NA	NA	Y	8
NA	NA	NA	NA	88	NA	NA	NA	NA	Y	8
NA	NA	NA	NA	88	Fa	NA	NA	NA	Y	8
NA	NA	NA	NA	88	Mo	NA	NA	NA	Y	8
NA	NA	NA	NA	88	Fa	NA	Fa	NA	Y	8
NA	NA	NA	NA	88	Fa	NA	NA	NA	Y	8
NA	NA	NA	NA	88	Mo	NA	NA	NA	Y	8
NA	NA	NA	NA	88	Mo	NA	NA	NA	Y	8
NA	NA	NA	NA	88	NA	NA	NA	NA	Y	8
NA	NA	NA	NA	88	Mo	NA	NA	NA	Y	8
NA	NA	NA	NA	88	Fa	NA	NA	NA	Y	8
NA	NA	NA	NA	88	Mo	NA	NA	NA	Y	8
NA	NA	NA	NA	88	Fa	NA	NA	NA	Y	8
NA	NA	NA	NA	88	NA	NA	NA	NA	Y	8
NA	NA	NA	NA	88	NA	NA	NA	NA	Y	8
NA	NA	NA	NA	88	NA	NA	NA	NA	Y	8
NA	NA	NA	NA	88	NA	NA	NA	NA	Y	8
NA	NA	NA	NA	88	Mo	NA	NA	NA	Y	8
NA	NA	NA	NA	88	Mo	NA	NA	NA	Y	8
NA	NA	NA	NA	88	Mo	NA	NA	NA	Y	8
NA	NA	NA	NA	88	Mo	NA	NA	NA	Y	8
NA	NA	NA	NA	88	NA	NA	NA	NA	Y	8
NA	NA	NA	NA	88	Mo	NA	NA	NA	Y	8
NA	NA	NA	NA	88	Mo	NA	NA	NA	N	8
NA	NA	NA	NA	88	Mo	NA	NA	NA	Y	8
NA	NA	NA	NA	88	NA	NA	NA	NA	N	8
DID NOT EAT THAT DAY	NA	NA	NA	88	NA	NA	NA	NA	Y	8
NA	NA	NA	NA	88	Mo	NA	NA	NA	Y	8
NA	NA	NA	NA	88	Ot	NA	NA	NA	Y	8
NA	NA	NA	NA	Ev	Fa	NA	NA	NA	Y	8
NA	NA	NA	NA	88	Mo	NA	NA	NA	Y	8
NA	NA	NA	NA	88	NA	NA	NA	NA	Y	8
NA	NA	NA	NA	88	NA	NA	NA	NA	Y	8
NA	NA	NA	NA	88	NA	NA	NA	NA	Y	8
NA	NA	NA	NA	88	NA	NA	NA	NA	Y	8
NA	NA	NA	NA	88	Mo	NA	NA	NA	Y	8
NA	NA	NA	NA	88	Mo	NA	NA	NA	Y	8
NA	NA	NA	NA	88	Ot	NA	Ot	NA	Y	8
NA	NA	NA	NA	88	Ot	NA	NA	NA	Y	8
NA	NA	NA	NA	88	Mo	NA	Mo	NA	Y	8
NA	NA	NA	NA	88	Mo	NA	Mo	NA	N	8
NA	NA	NA	NA	88	Ot	NA	NA	NA	Y	8
NA	NA	NA	NA	88	Mo	NA	NA	NA	Y	8
NA	NA	NA	NA	88	Ot	NA	NA	NA	Y	8

NA	NA	NA	NA	88	Ot	NA	NA	NA	Y	8
NA	NA	NA	NA	88	Mo	NA	NA	NA	Y	8
NA	NA	NA	NA	88	NA	NA	NA	NA	Y	8
NA	NA	NA	NA	88	NA	NA	NA	NA	Y	8
NA	NA	NA	NA	88	Ot	NA	NA	NA	Y	8
NA	NA	NA	NA	88	NA	NA	NA	NA	Y	8
NA	NA	NA	NA	88	Mo	NA	NA	NA	Y	8
NA	NA	NA	NA	88	Mo	NA	NA	NA	Y	8
NA	NA	NA	NA	88	NA	NA	NA	NA	Y	8
NA	NA	NA	NA	88	NA	NA	NA	NA	Y	8
NA	NA	NA	NA	88	Ot	NA	NA	NA	Y	8
NA	NA	NA	NA	88	Mo	NA	NA	NA	Y	8
NA	NA	NA	NA	88	Mo	NA	NA	NA	Y	8
NA	NA	NA	NA	88	Mo	NA	NA	NA	Y	8
NA	NA	NA	NA	88	NA	NA	NA	NA	Y	8
NA	NA	NA	NA	88	Ot	NA	NA	NA	Y	8
NA	NA	NA	NA	88	Mo	NA	NA	NA	Y	8
NA	NA	NA	NA	88	Mo	NA	NA	NA	Y	8
NA	NA	NA	NA	88	Mo	NA	NA	NA	Y	8
NA	NA	NA	NA	88	Mo	NA	Mo	NA	Y	8
NA	NA	NA	NA	Ye	Mo	NA	NA	NA	Y	8
NA	NA	NA	NA	88	Mo	NA	NA	NA	Y	8
NA	NA	NA	NA	88	Mo	NA	NA	NA	N	8
NA	NA	NA	NA	88	NA	NA	NA	NA	Y	8
NA	NA	NA	NA	88	Mo	NA	NA	NA	Y	8
NA	NA	NA	NA	88	NA	NA	NA	NA	Y	8
NA	NA	NA	NA	88	Ot	NA	NA	NA	Y	8
NA	NA	NA	NA	88	NA	NA	NA	NA	Y	8
NA	NA	NA	NA	88	Fa	NA	NA	NA	Y	8
NA	NA	NA	NA	88	Mo	NA	NA	NA	Y	8
NA	NA	NA	NA	88	Mo	NA	NA	NA	Y	8
NA	NA	NA	NA	88	Mo	NA	NA	NA	Y	8
NA	NA	NA	NA	88	NA	NA	NA	NA	Y	8
NA	NA	NA	NA	88	NA	NA	NA	NA	Y	8
NA	NA	NA	NA	88	NA	NA	NA	NA	Y	8
NA	NA	NA	NA	88	Mo	NA	NA	NA	Y	8
NA	NA	NA	NA	88	Mo	NA	NA	NA	Y	8
NA	NA	NA	NA	88	Mo	NA	NA	NA	Y	8
NA	NA	NA	NA	88	Mo	NA	NA	NA	Y	8
NA	NA	NA	NA	88	Mo	NA	NA	NA	Y	8
NA	NA	NA	NA	88	Mo	NA	Mo	NA	Y	8
NA	NA	NA	NA	88	Mo	NA	NA	NA	Y	8
NA	NA	NA	NA	88	Mo	NA	NA	NA	Y	8
NA	NA	NA	NA	88	Mo	NA	NA	NA	Y	8
NA	NA	NA	NA		Mo	NA	NA	NA	Y	8
NA	NA	NA	NA	88	Mo	NA	NA	NA	Y	8
NA	NA	NA	NA	Ye	NA	NA	NA	NA	Y	8

NA	NA	NA	NA	88	NA	NA	NA	NA	Y	8
NA	NA	NA	NA	88	Ot	NA	NA	NA	Y	8
NA	NA	NA	NA	88	NA	NA	NA	NA	Y	8
NA	NA	NA	NA	88	NA	NA	NA	NA	N	8
NA	NA	NA	NA		Mo	NA	NA	NA	Y	8
NA	NA	NA	NA	88	Mo	NA	NA	NA	Y	8
NA	NA	NA	NA	88	Mo	NA	NA	NA	Y	8
NA	NA	NA	NA	88	Mo	NA	NA	NA	Y	8
NA	NA	NA	NA	88	Fa	NA	NA	NA	Y	8
NA	NA	NA	NA	88	Mo	NA	NA	NA	Y	8
NA	NA	NA	NA	88	Mo	NA	NA	NA	Y	8
NA	NA	NA	NA	88	Mo	NA	NA	NA	Y	8
NA	NA	NA	NA	88	Mo				Y	8
NA	NA	NA	NA	88	NA	NA	NA	NA	Y	8
NA	NA	NA	NA	88	NA	NA	NA	NA	Y	8
NA	NA	NA	NA	88	Mo	NA	NA	NA	Y	8
NA	NA	NA	NA	88	NA	NA	NA	NA	Y	8
NA	NA	NA	NA	88	NA	NA	NA	NA	Y	8
NA	NA	NA	NA	88	NA	NA	NA	NA	Y	8
NA	NA	NA	NA	88	Ot	NA	NA	NA	Y	8
NA	NA	NA	NA	88	NA	NA	NA	NA	Y	8
NA	NA	NA	NA	88	Mo	NA	NA	NA	Y	8
NA	NA	NA	NA	88	Fa	NA	NA	NA	Y	8
NA	NA	NA	NA	88	Mo	NA	NA	NA	Y	8
NA	NA	NA	NA	88	Mo	NA	NA	NA	Y	8
NA	NA	NA	NA	88	Mo	NA	NA	NA	Y	8
NA	NA	NA	NA	88	Mo	NA	NA	NA	Y	8
NA	NA	NA	NA	88	NA	NA	NA	NA	Y	8
NA	NA	NA	NA	88	NA	NA	NA	NA	Y	8
NA	NA	NA	NA	88	Mo	NA	NA	NA	Y	8
NA	NA	NA	NA	88	Mo	NA	NA	NA	Y	8
NA	NA	NA	NA	88	Ot	NA	NA	NA	Y	8
NA	NA	NA	NA	88	Ot	NA	NA	NA	Y	8
NA	NA	NA	NA	88	Mo	NA	NA	NA	Y	8
NA	NA	NA	NA	Ev	Mo	NA	NA	NA	Y	8
NA	NA	NA	NA	88	Mo	NA	NA	NA	Y	8
NA	NA	NA	NA	88	Mo	NA	NA	NA	Y	8
NA	NA	NA	NA	88	Mo	NA	NA	NA	Y	8
NA	NA	NA	NA	88	NA	NA	NA	NA	Y	8
NA	NA	NA	NA	88	NA	NA	NA	NA	Y	8
NA	NA	NA	NA	88	Ot	NA	NA	NA	Y	8
NA	NA	NA	NA	88	NA	NA	NA	NA	Y	8
NA	NA	NA	NA	88	NA	NA	NA	NA	Y	8
NA	NA	NA	NA	88	Mo	NA	NA	NA	Y	8
NA	NA	NA	NA	88	NA	NA	NA	NA	Y	8
NA	NA	NA	NA	88	NA	NA	NA	NA	Y	8
NA	NA	NA	NA	88	NA	NA	NA	NA	Y	8

NA	NA	NA	NA	88	NA	NA	NA	NA	Y	8
NA	NA	NA	NA	88	Fa	NA	NA	NA	Y	8
NA	NA	NA	NA	88	Mo	NA	NA	NA	Y	8
NA	NA	NA	NA	88	NA	NA	NA	NA	Y	8
NA	NA	NA	NA	88	Mo	NA	NA	NA	Y	8
NA	NA	NA	NA	88	NA	NA	NA	NA	Y	8
NA	NA	NA	NA	88	Mo	NA	NA	NA	Y	8
NA	NA	NA	NA	88	Mo	NA	NA	NA	Y	8
NA	NA	NA	NA	88	Mo	NA	NA	NA	Y	8
NA	NA	NA	NA	88	Fa	NA	NA	NA	N	8
NA	NA	NA	NA	88	Mo	NA	NA	NA	N	8
NA	NA	NA	NA	88	Mo	NA	NA	NA	Y	8
NA	NA	NA	NA	88	Mo	NA	NA	NA	Y	8
NA	NA	NA	NA	88	Mo	NA	NA	NA	Y	8
NA	NA	NA	NA	88	Mo	NA	NA	NA	Y	8
NA	NA	NA	NA	88	Fa	NA	NA	NA	Y	8
NA	NA	NA	NA	88	Mo	NA	NA	NA	Y	8
NA	NA	NA	NA	88	Ot	NA	NA	NA	Y	8
NA	NA	NA	NA	88	Ot	NA	NA	NA	Y	8
NA	NA	NA	NA	88	Mo	NA	NA	NA	Y	8
NA	NA	NA	NA	88	Mo	NA	NA	NA	Y	8
NA	NA	NA	NA	88	NA	NA	NA	NA	Y	8
NA	NA	NA	NA	88	Mo	NA	NA	NA	Y	8
NA	NA	NA	NA	88	Mo	NA	NA	NA	Y	8
NA	NA	NA	NA	88	Ot	NA	NA	NA	Y	8
NA	NA	NA	NA	88	Mo	NA	NA	NA	Y	8
NA	NA	NA	NA	88	Mo	NA	NA	NA	Y	8
NA	NA	NA	NA	88	Mo	NA	NA	NA	Y	8
NA	NA	NA	NA	88	Mo	NA	NA	NA	Y	8
NA	NA	NA	NA	88	Mo	NA	NA	NA	Y	8
NA	NA	NA	NA	88	Mo	NA	NA	NA	Y	8
NA	NA	NA	NA	88	Mo	NA	NA	NA	Y	8
NA	NA	NA	NA	88	Mo	NA	NA	NA	Y	8
NA	NA	NA	NA	88	Mo	NA	NA	NA	Y	8
NA	NA	NA	NA	88	Mo	NA	NA	NA	Y	8
NA	NA	NA	NA	88	NA	NA	NA	NA	Y	8
NA	NA	NA	NA	88	Mo	NA	NA	NA	Y	8
NA	NA	NA	NA	88	Mo	NA	NA	NA	Y	8
NA	NA	NA	NA	88	Fa	NA	NA	NA	Y	8
NA	NA	NA	NA	88	Mo	NA	NA	NA	Y	8
NA	NA	NA	NA	88	Mo	NA	NA	NA	Y	8
NA	NA	NA	NA	88	Mo	NA	NA	NA	Y	8
NA	NA	NA	NA	88	NA	NA	NA	NA	Y	8
NA	NA	NA	NA	88	Mo	NA	NA	NA	Y	8
NA	NA	NA	NA	88	Mo	NA	NA	NA	Y	8
NA	NA	NA	NA	88	Mo	NA	NA	NA	Y	8
NA	NA	NA	NA	88	Mo	NA	NA	NA	Y	8

NA	NA	NA	NA	88	Mo	NA	NA	NA	Y	8
NA	NA	NA	NA	88	Mo	NA	NA	NA	Y	8
NA	NA	NA	NA	88	Mo	NA	NA	NA	Y	8
NA	NA	NA	NA	88	Mo	NA	NA	NA	Y	8
NA	NA	NA	NA	88	Mo	NA	NA	NA	Y	8
NA	NA	NA	NA	88	Ot	NA	NA	NA	Y	8
NA	NA	NA	NA	88	Mo	NA	NA	NA	Y	8
NA	NA	NA	NA	88	Mo	NA	NA	NA	Y	8
NA	NA	NA	NA	88	Mo	NA	NA	NA	Y	8
NA	NA	NA	NA	88	Mo	NA	NA	NA	Y	8
NA	NA	NA	NA	88	Mo	NA	NA	NA	Y	8
NA	NA	NA	NA	No	Mo	NA	NA	NA	Y	8
NA	NA	NA	NA	88	Mo	NA	NA	NA	Y	8
NA	NA	NA	NA	88	Mo	NA	NA	NA	Y	8
NA	NA	NA	NA	88	NA	NA	NA	NA	Y	8
NA	NA	NA	NA	88	Mo	NA	NA	NA	Y	8
NA	NA	NA	NA	88	Fa	NA	NA	NA	Y	8
NA	NA	NA	NA	88	Mo	NA	NA	NA	Y	8
NA	NA	NA	NA	88	Mo	NA	NA	NA	Y	8
NA	NA	NA	NA	88	NA	NA	NA	NA	Y	8
NA	NA	NA	NA	88	NA	NA	NA	NA	Y	8
NA	NA	NA	NA	88	Mo	NA	NA	NA	Y	8
NA	NA	NA	NA	88	Mo	NA	NA	NA	Y	8
NA	NA	NA	NA	88	Mo	NA	NA	NA	Y	8
NA	NA	NA	NA	88	NA	NA	NA	NA	Y	8
NA	NA	NA	NA	88	Mo	NA	NA	NA	Y	8
NA	NA	NA	NA	88	Mo	NA	NA	NA	Y	8
NA	NA	NA	NA	88	Fa	NA	NA	NA	Y	8
NA	NA	NA	NA	88	NA	NA	NA	NA	Y	8
NA	NA	NA	NA	88	NA	NA	NA	NA	Y	8
NA	NA	NA	NA	88	NA	NA	NA	NA	Y	8
NA	NA	NA	NA	88	Mo	NA	NA	NA	Y	8
NA	NA	NA	NA	88	NA	NA	NA	NA	Y	8
NA	NA	NA	NA	88	NA	NA	NA	NA	Y	8
NA	NA	NA	NA	88	NA	NA	NA	NA	Y	8
NA	NA	NA	NA	88	NA	NA	NA	NA	Y	8
NA	NA	NA	NA	88	Ot	NA	NA	NA	Y	8
NA	NA	NA	NA	88	NA	NA	NA	NA	Y	8
NA	NA	NA	NA	88	Ot	NA	NA	NA	Y	8
NA	NA	NA	NA	88	Mo	NA	NA	NA	Y	8
NA	NA	NA	NA	88	Mo	NA	NA	NA	Y	8
NA	NA	NA	NA	88	Ot	NA	NA	NA	Y	8
NA	NA	NA	NA	88	Mo	NA	NA	NA	Y	8
NA	NA	NA	NA	88	Mo	NA	Mo	NA	Y	8
NA	NA	NA	NA	88	NA	NA	NA	NA	Y	8
NA	NA	NA	NA	88	NA	NA	NA	NA	Y	8
NA	NA	NA	NA	88	Mo	NA	NA	NA	Y	8

NA	NA	NA	NA	88	Ot	NA	NA	NA	Y	8
NA	NA	NA	NA	88	Mo	NA	NA	NA	Y	8
NA	NA	NA	NA	88	Mo	NA	NA	NA	Y	8
NA	NA	NA	NA	88	Mo	NA	NA	NA	Y	8
NA	NA	NA	NA	88	Mo	NA	NA	NA	Y	8
NA	NA	NA	NA	88	Mo	NA	NA	NA	Y	8
NA	NA	NA	NA	88	NA	NA	NA	NA	Y	8
NA	NA	NA	NA	88	Mo	NA	NA	NA	Y	8
NA	NA	NA	NA	88	NA	NA	NA	NA	Y	8
NA	NA	NA	NA	88	NA	NA	NA	NA	Y	8
NA	NA	NA	NA	88	Mo	NA	NA	NA	Y	8
NA	NA	NA	NA	88	Mo	NA	NA	NA	Y	8
NA	NA	NA	NA	88	Mo	NA	NA	NA	Y	8
NA	NA	NA	NA	88	Mo	NA	NA	NA	Y	8
NA	NA	NA	NA	88	Mo	NA	NA	NA	Y	8
NA	NA	NA	NA	88	Mo	NA	NA	NA	Y	8
NA	NA	NA	NA	88	Mo	NA	NA	NA	Y	8
NA	NA	NA	NA	88	Mo	NA	NA	NA	Y	8
NA	NA	NA	NA	88	Mo	NA	NA	NA	Y	8
NA	NA	NA	NA	88	Mo	NA	NA	NA	Y	8
NA	NA	NA	NA	88	Mo	NA	NA	NA	Y	8
NA	NA	NA	NA	88	Mo	NA	NA	NA	Y	8
NA	NA	NA	NA	88	NA	NA	NA	NA	Y	8
NA	NA	NA	NA	88	Mo	NA	NA	NA	Y	8
NA	NA	NA	NA	88	Mo	NA	NA	NA	Y	8
NA	NA	NA	NA	88	Mo	NA	NA	NA	Y	8
NA	NA	NA	NA	88	Mo	NA	NA	NA	Y	8
NA	NA	NA	NA	88	Mo	NA	NA	NA	Y	8
NA	NA	NA	NA	88	NA	NA	NA	NA	Y	8
NA	NA	NA	NA	88	Mo	NA	NA	NA	Y	8
NA	NA	NA	NA	88	Mo	NA	NA	NA	Y	8
NA	NA	NA	NA	88	Mo	NA	NA	NA	Y	8
NA	NA	NA	NA	88	NA	NA	NA	NA	Y	8
NA	NA	NA	NA	88	Mo	NA	NA	NA	Y	8
NA	NA	NA	NA	88	Mo	NA	NA	NA	Y	8
NA	NA	NA	NA	88	Mo	NA	NA	NA	Y	8
NA	NA	NA	NA	88	NA	NA	NA	NA	Y	8
NA	NA	NA	NA	88	Mo	NA	NA	NA	Y	8
NA	NA	NA	NA	88	Ot	NA	NA	NA	Y	8
NA	NA	NA	NA	88	Mo	NA	NA	NA	Y	8
NA	NA	NA	NA	88	Mo	NA	NA	NA	Y	8
NA	NA	NA	NA	88	Ot	NA	NA	NA	Y	8
NA	NA	NA	NA	88	Ot	NA	NA	NA	Y	8
NA	NA	NA	NA	88	Mo	NA	NA	NA	Y	8
NA	NA	NA	NA	88	Ot	NA	NA	NA	Y	8
NA	NA	NA	NA	88	Mo	NA	NA	NA	Y	8
NA	NA	NA	NA	88	Ot	NA	NA	NA	Y	8

NA	NA	NA	NA	88	Mo	NA	NA	NA	Y	8
NA	NA	NA	NA	88	Ot	NA	NA	NA	Y	8
NA	NA	NA	NA	No	Fa	NA	NA	NA	Y	8
NA	NA	NA	NA	88	Mo	NA	NA	NA	Y	8
NA	NA	NA	NA	88	Mo	NA	NA	NA	Y	8
NA	NA	NA	NA	88	Mo	NA	NA	NA	Y	8
NA	NA	NA	NA	88	Mo	NA	NA	NA	Y	8
NA	NA	NA	NA	88	Mo	NA	NA	NA	Y	8
NA	NA	NA	NA	88	Mo	NA	NA	NA	Y	8
NA	NA	NA	NA	88	Mo	NA	NA	NA	Y	8
NA	NA	NA	NA	88	Mo	NA	Mo	NA	Y	8
NA	NA	NA	NA	88	Mo	NA	NA	NA	Y	8
NA	NA	NA	NA	88	Mo	NA	NA	NA	Y	8
NA	NA	NA	NA	88	Mo	NA	NA	NA	Y	8
NA	NA	NA	NA	88	Mo	NA	NA	NA	Y	8
NA	NA	NA	NA	88	Ot	NA	NA	NA	Y	8
NA	NA	NA	NA	88	Mo	NA	NA	NA	Y	8
NA	NA	NA	NA	88	Mo	NA	NA	NA	Y	8

q36d2	q36d3	q37i	q37i1	q37i2	q37i3	q37anti	q37anti1	q37anti2	q37anti3	q37other	q37other1
8	8	88	88	88	88	NA	NA	NA	NA	NA	NA
8	8	88	88	88	88	NA	NA	NA	NA	NA	NA
8	8	88	88	88	88	NA	NA	NA	NA	NA	NA
8	8	88	88	88	88	NA	NA	NA	NA	NA	NA
8	8	88	88	88	88	NA	NA	NA	NA	NA	NA
8	8	88	88	88	88	NA	NA	NA	NA	NA	NA
8	8	88	88	88	88	NA	NA	NA	NA	NA	NA
8	8	88	88	88	88	NA	NA	NA	NA	NA	NA
8	8	88	88	88	88	NA	NA	NA	NA	NA	NA
8	8	88	88	88	88	NA	NA	NA	NA	NA	NA
8	8	88	88	88	88	NA	NA	NA	NA	NA	NA
8	8	88	88	88	88	NA	NA	NA	NA	NA	NA
8	8	88	88	88	88	NA	NA	NA	NA	NA	NA
8	8	88	88	88	88	NA	NA	NA	NA	NA	NA
8	8	88	88	88	88	NA	NA	NA	NA	NA	NA
8	8	88	88	88	88	NA	NA	NA	NA	NA	NA
8	8	88	88	88	88	NA	NA	NA	NA	NA	NA
8	8	88	88	88	88	NA	NA	NA	NA	NA	NA
8	8	88	88	88	88	NA	NA	NA	NA	NA	NA
8	8	88	88	88	88	NA	NA	NA	NA	NA	NA
8	8	88	88	88	88	NA	NA	NA	NA	NA	NA
8	8	88	88	88	88	NA	NA	NA	NA	NA	NA
8	8	88	88	88	88	NA	NA	NA	NA	NA	NA
8	8	88	88	88	88	NA	NA	NA	NA	NA	NA
8	8	88	88	88	88	NA	NA	NA	NA	NA	NA
8	8	88	88	88	88	NA	NA	NA	NA	NA	NA
8	8	88	88	88	88	NA	NA	NA	NA	NA	NA
8	8	ot	88	88	88	NA	NA	NA	NA	WAS TOO	NA
8	8	88	88	88	88	NA	NA	NA	NA	NA	NA
8	8	88	88	88	88	NA	NA	NA	NA	NA	NA
8	8	88	88	88	88	NA	NA	NA	NA	NA	NA
8	8	88	88	88	88	NA	NA	NA	NA	NA	NA
8	8	88	88	88	88	NA	NA	NA	NA	NA	NA
8	8	88	88	88	88	NA	NA	NA	NA	NA	NA
8	8	88	88	88	88	NA	NA	NA	NA	NA	NA
8	8	88	88	88	88	NA	NA	NA	NA	NA	NA
8	8	88	88	88	88	NA	NA	NA	NA	NA	NA
8	8	88	88	88	88	NA	NA	NA	NA	NA	NA
8	8	88	88	88	88	NA	NA	NA	NA	NA	NA
8	8	88	88	88	88	NA	NA	NA	NA	NA	NA
8	8	88	88	88	88	NA	NA	NA	NA	NA	NA
8	8	88	88	88	88	NA	NA	NA	NA	NA	NA
N	8	ot	88	88	88	NA	NA	NA	NA	CHILD WAS	NA
8	8	88	88	88	88	NA	NA	NA	NA	NA	NA
8	8	88	88	88	88	NA	NA	NA	NA	NA	NA
8	8	88	88	88	88	NA	NA	NA	NA	NA	NA

8	8	88	88	88	88	NA	NA	NA	NA	NA	NA
8	8	88	88	88	88	NA	NA	NA	NA	NA	NA
8	8	88	88	88	88	NA	NA	NA	NA	NA	NA
8	8	88	88	88	88	NA	NA	NA	NA	NA	NA
8	8	88	88	88	88	NA	NA	NA	NA	NA	NA
8	8	88	88	88	88	NA	NA	NA	NA	NA	NA
8	8	88	88	88	88	NA	NA	NA	NA	NA	NA
8	8	88	88	88	88	NA	NA	NA	NA	NA	NA
8	8	88	88	88	88	NA	NA	NA	NA	NA	NA
8	8	88	88	88	88	NA	NA	NA	NA	NA	NA
8	8	88	88	88	88	NA	NA	NA	NA	NA	NA
8	8	88	88	88	88	NA	NA	NA	NA	NA	NA
8	8	88	88	88	88	NA	NA	NA	NA	NA	NA
8	8	88	88	88	88	NA	NA	NA	NA	NA	NA
8	8	88	88	88	88	NA	NA	NA	NA	NA	NA
8	8	88	88	88	88	NA	NA	NA	NA	NA	NA
8	8	88	88	88	88	NA	NA	NA	NA	NA	NA
8	8	88	88	88	88	NA	NA	NA	NA	NA	NA
8	8	88	88	88	88	NA	NA	NA	NA	NA	NA
8	8	88	88	88	88	NA	NA	NA	NA	NA	NA
8	8	88	88	88	88	NA	NA	NA	NA	NA	NA
8	8	88	88	88	88	NA	NA	NA	NA	NA	NA
8	8	Fo	88	88	88	NA	NA	NA	NA	NA	NA
8	8	88	88	88	88	NA	NA	NA	NA	NA	NA
8	8	88	88	88	88	NA	NA	NA	NA	NA	NA
8	8	88	88	88	88	NA	NA	NA	NA	NA	NA
8	8	88	88	88	88	NA	NA	NA	NA	NA	NA
8	8	88	88	88	88	NA	NA	NA	NA	NA	NA
8	8	88	88	88	88	NA	NA	NA	NA	NA	NA
8	8	88	88	88	88	NA	NA	NA	NA	NA	NA
8	8	88	88	88	88	NA	NA	NA	NA	NA	NA
8	8	88	88	88	88	NA	NA	NA	NA	NA	NA
8	8	88	88	88	88	NA	NA	NA	NA	NA	NA
8	8	88	88	88	88	NA	NA	NA	NA	NA	NA
8	8	88	88	88	88	NA	NA	NA	NA	NA	NA
8	8	88	88	88	88	NA	NA	NA	NA	NA	NA
8	8	88	88	88	88	NA	NA	NA	NA	NA	NA
8	8	88	88	88	88	NA	NA	NA	NA	NA	NA
8	8	88	88	88	88	NA	NA	NA	NA	NA	NA
8	8	88	88	88	88	NA	NA	NA	NA	NA	NA
8	8	88	88	88	88	NA	NA	NA	NA	NA	NA
8	8	88	88	88	88	NA	NA	NA	NA	NA	NA
8	8	88	88	88	88	NA	NA	NA	NA	NA	NA
8	8	88	88	88	88	NA	NA	NA	NA	NA	NA
8	8	88	88	88	88	NA	NA	NA	NA	NA	NA
8	8	88	88	88	88	NA	NA	NA	NA	NA	NA
8	8	88	88	88	88	NA	NA	NA	NA	NA	NA

8	8	88	88	88	88	NA	NA	NA	NA	NA	NA
8	8	88	88	88	88	NA	NA	NA	NA	NA	NA
8	8	88	88	88	88	NA	NA	NA	NA	NA	NA
8	8	Si	88	88	88	NA	NA	NA	NA	NA	NA
8	8	88	88	88	88	NA	NA	NA	NA	NA	NA
8	8	88	88	88	88	NA	NA	NA	NA	NA	NA
8	8	88	88	88	88	NA	NA	NA	NA	NA	NA
8	8	88	88	88	88	NA	NA	NA	NA	NA	NA
8	8	88	88	88	88	NA	NA	NA	NA	NA	NA
8	8	88	88	88	88	NA	NA	NA	NA	NA	NA
8	8	88	88	88	88	NA	NA	NA	NA	NA	NA
8	8	88	88	88	88	NA	NA	NA	NA	NA	NA
8	8	88	88	88	88	NA	NA	NA	NA	NA	NA
8	8	88	88	88	88	NA	NA	NA	NA	NA	NA
8	8	88	88	88	88	NA	NA	NA	NA	NA	NA
8	8	88	88	88	88	NA	NA	NA	NA	NA	NA
8	8	88	88	88	88	NA	NA	NA	NA	NA	NA
8	8	88	88	88	88	NA	NA	NA	NA	NA	NA
8	8	88	88	88	88	NA	NA	NA	NA	NA	NA
8	8	88	88	88	88	NA	NA	NA	NA	NA	NA
8	8	88	88	88	88	NA	NA	NA	NA	NA	NA
8	8	88	88	88	88	NA	NA	NA	NA	NA	NA
8	8	88	88	88	88	NA	NA	NA	NA	NA	NA
8	8	88	88	88	88	NA	NA	NA	NA	NA	NA
8	8	88	88	88	88	NA	NA	NA	NA	NA	NA
8	8	88	88	88	88	NA	NA	NA	NA	NA	NA
8	8	88	88	88	88	NA	NA	NA	NA	NA	NA
8	8	88	88	88	88	NA	NA	NA	NA	NA	NA
8	8	88	88	88	88	NA	NA	NA	NA	NA	NA
8	8	88	88	88	88	NA	NA	NA	NA	NA	NA
8	8	88	88	88	88	NA	NA	NA	NA	NA	NA
8	8	88	88	88	88	NA	NA	NA	NA	NA	NA
8	8	88	88	88	88	NA	NA	NA	NA	NA	NA
8	8	88	88	88	88	NA	NA	NA	NA	NA	NA
8	8	88	88	88	88	NA	NA	NA	NA	NA	NA
8	8	88	88	88	88	NA	NA	NA	NA	NA	NA
8	8	88	88	88	88	NA	NA	NA	NA	NA	NA
8	8	88	88	88	88	NA	NA	NA	NA	NA	NA
8	8	88	88	88	88	NA	NA	NA	NA	NA	NA
8	8	88	88	88	88	NA	NA	NA	NA	NA	NA
8	8	88	88	88	88	NA	NA	NA	NA	NA	NA
8	8	88	88	88	88	NA	NA	NA	NA	NA	NA
8	8	88	88	88	88	NA	NA	NA	NA	NA	NA
8	8	88	88	88	88	NA	NA	NA	NA	NA	NA
8	8	88	88	88	88	NA	NA	NA	NA	NA	NA
8	8	88	88	88	88	NA	NA	NA	NA	NA	NA
8	8	88	88	88	88	NA	NA	NA	NA	NA	NA

8	8	88	88	88	88	NA	NA	NA	NA	NA	NA
8	8	88	88	88	88	NA	NA	NA	NA	NA	NA
8	8	88	88	88	88	NA	NA	NA	NA	NA	NA
8	8	88	88	88	88	NA	NA	NA	NA	NA	NA
8	8	88	88	88	88	NA	NA	NA	NA	NA	NA
8	8	88	88	88	88	NA	NA	NA	NA	NA	NA
8	8	88	88	88	88	NA	NA	NA	NA	NA	NA
8	8	88	88	88	88	NA	NA	NA	NA	NA	NA
8	8	88	88	88	88	NA	NA	NA	NA	NA	NA
N	8	Fo	88	88	88	NA	NA	NA	NA	NA	NA
N	8	No	88	88	88	NA	NA	NA	NA	NA	NA
8	8	88	88	88	88	NA	NA	NA	NA	NA	NA
8	8	88	88	88	88	NA	NA	NA	NA	NA	NA
8	8	88	88	88	88	NA	NA	NA	NA	NA	NA
8	8	88	88	88	88	NA	NA	NA	NA	NA	NA
8	8	88	88	88	88	NA	NA	NA	NA	NA	NA
8	8	88	88	88	88	NA	NA	NA	NA	NA	NA
8	8	88	88	88	88	NA	NA	NA	NA	NA	NA
8	8	88	88	88	88	NA	NA	NA	NA	NA	NA
8	8	88	88	88	88	NA	NA	NA	NA	NA	NA
8	8	88	88	88	88	NA	NA	NA	NA	NA	NA
8	8	88	88	88	88	NA	NA	NA	NA	NA	NA
8	8	88	88	88	88	NA	NA	NA	NA	NA	NA
8	8	88	88	88	88	NA	NA	NA	NA	NA	NA
8	8	88	88	88	88	NA	NA	NA	NA	NA	NA
8	8	88	88	88	88	NA	NA	NA	NA	NA	NA
8	8	88	88	88	88	NA	NA	NA	NA	NA	NA
8	8	88	88	88	88	NA	NA	NA	NA	NA	NA
8	8	88	88	88	88	NA	NA	NA	NA	NA	NA
8	8	88	88	88	88	NA	NA	NA	NA	NA	NA
8	8	88	88	88	88	NA	NA	NA	NA	NA	NA
8	8	88	88	88	88	NA	NA	NA	NA	NA	NA
8	8	88	88	88	88	NA	NA	NA	NA	NA	NA
8	8	88	88	88	88	NA	NA	NA	NA	NA	NA
8	8	88	88	88	88	NA	NA	NA	NA	NA	NA
8	8	88	88	88	88	NA	NA	NA	NA	NA	NA
8	8	88	88	88	88	NA	NA	NA	NA	NA	NA
8	8	88	88	88	88	NA	NA	NA	NA	NA	NA
8	8	88	88	88	88	NA	NA	NA	NA	NA	NA
8	8	88	88	88	88	NA	NA	NA	NA	NA	NA
8	8	88	88	88	88	NA	NA	NA	NA	NA	NA
8	8	88	88	88	88	NA	NA	NA	NA	NA	NA
8	8	88	88	88	88	NA	NA	NA	NA	NA	NA
8	8	88	88	88	88	NA	NA	NA	NA	NA	NA
8	8	88	88	88	88	NA	NA	NA	NA	NA	NA
8	8	88	88	88	88	NA	NA	NA	NA	NA	NA
8	8	88	88	88	88	NA	NA	NA	NA	NA	NA

8	8	88	88	88	88	NA	NA	NA	NA	NA	NA
8	8	88	88	88	88	NA	NA	NA	NA	NA	NA
8	8	88	88	88	88	NA	NA	NA	NA	NA	NA
8	8	88	88	88	88	NA	NA	NA	NA	NA	NA
8	8	88	88	88	88	NA	NA	NA	NA	NA	NA
8	8	88	88	88	88	NA	NA	NA	NA	NA	NA
8	8	88	88	88	88	NA	NA	NA	NA	NA	NA
8	8	88	88	88	88	NA	NA	NA	NA	NA	NA
8	8	88	88	88	88	NA	NA	NA	NA	NA	NA
8	8	88	88	88	88	NA	NA	NA	NA	NA	NA
8	8	88	88	88	88	NA	NA	NA	NA	NA	NA
8	8	88	88	88	88	NA	NA	NA	NA	NA	NA
8	8	88	88	88	88	NA	NA	NA	NA	NA	NA
8	8	88	88	88	88	NA	NA	NA	NA	NA	NA
8	8	88	88	88	88	NA	NA	NA	NA	NA	NA
8	8	88	88	88	88	NA	NA	NA	NA	NA	NA
8	8	88	88	88	88	NA	NA	NA	NA	NA	NA
8	8	88	88	88	88	NA	NA	NA	NA	NA	NA
8	8	88	88	88	88	NA	NA	NA	NA	NA	NA
8	8	88	88	88	88	NA	NA	NA	NA	NA	NA
8	8	88	88	88	88	NA	NA	NA	NA	NA	NA
8	8	88	88	88	88	NA	NA	NA	NA	NA	NA
8	8	88	88	88	88	NA	NA	NA	NA	NA	NA
8	8	88	88	88	88	NA	NA	NA	NA	NA	NA
8	8	88	88	88	88	NA	NA	NA	NA	NA	NA
8	8	88	88	88	88	NA	NA	NA	NA	NA	NA
8	8	88	88	88	88	NA	NA	NA	NA	NA	NA
8	8	88	88	88	88	NA	NA	NA	NA	NA	NA
8	8	88	88	88	88	NA	NA	NA	NA	NA	NA
8	8	88	88	88	88	NA	NA	NA	NA	NA	NA
8	8	88	88	88	88	NA	NA	NA	NA	NA	NA
8	8	88	88	88	88	NA	NA	NA	NA	NA	NA
8	8	88	88	88	88	NA	NA	NA	NA	NA	NA
8	8	88	88	88	88	NA	NA	NA	NA	NA	NA
8	8	88	88	88	88	NA	NA	NA	NA	NA	NA
8	8	88	88	88	88	NA	NA	NA	NA	NA	NA
8	8	88	88	88	88	NA	NA	NA	NA	NA	NA
8	8	88	88	88	88	NA	NA	NA	NA	NA	NA
8	8	88	88	88	88	NA	NA	NA	NA	NA	NA
8	8	88	88	88	88	NA	NA	NA	NA	NA	NA
8	8	88	88	88	88	NA	NA	NA	NA	NA	NA
8	8	88	88	88	88	NA	NA	NA	NA	NA	NA
8	8	88	88	88	88	NA	NA	NA	NA	NA	NA
8	8	88	88	88	88	NA	NA	NA	NA	NA	NA
8	8	88	88	88	88	NA	NA	NA	NA	NA	NA
8	8	88	88	88	88	NA	NA	NA	NA	NA	NA
8	8	88	88	88	88	NA	NA	NA	NA	NA	NA

8	8	88	88	88	88	NA	NA	NA	NA	NA	NA
8	8	88	88	88	88	NA	NA	NA	NA	NA	NA
8	8	88	88	88	88	NA	NA	NA	NA	NA	NA
8	8	88	88	88	88	NA	NA	NA	NA	NA	NA
8	8	88	88	88	88	NA	NA	NA	NA	NA	NA
8	8	88	88	88	88	NA	NA	NA	NA	NA	NA
8	8	88	88	88	88	NA	NA	NA	NA	NA	NA
8	8	88	88	88	88	NA	NA	NA	NA	NA	NA
8	8	88	88	88	88	NA	NA	NA	NA	NA	NA
8	8	88	88	88	88	NA	NA	NA	NA	NA	NA
8	8	88	88	88	88	NA	NA	NA	NA	NA	NA
8	8	88	88	88	88	NA	NA	NA	NA	NA	NA
8	8	88	88	88	88	NA	NA	NA	NA	NA	NA
8	8	88	88	88	88	NA	NA	NA	NA	NA	NA
8	8	88	88	88	88	NA	NA	NA	NA	NA	NA
8	8	88	88	88	88	NA	NA	NA	NA	NA	NA
8	8	88	88	88	88	NA	NA	NA	NA	NA	NA
8	8	88	88	88	88	NA	NA	NA	NA	NA	NA
8	8	88	88	88	88	NA	NA	NA	NA	NA	NA
8	8	88	88	88	88	NA	NA	NA	NA	NA	NA
8	8	88	88	88	88	NA	NA	NA	NA	NA	NA
8	8	88	88	88	88	NA	NA	NA	NA	NA	NA
8	8	88	88	88	88	NA	NA	NA	NA	NA	NA
8	8	88	88	88	88	NA	NA	NA	NA	NA	NA
8	8	88	88	88	88	NA	NA	NA	NA	NA	NA
8	8	88	88	88	88	NA	NA	NA	NA	NA	NA
8	8	88	88	88	88	NA	NA	NA	NA	NA	NA
8	8	88	88	88	88	NA	NA	NA	NA	NA	NA
8	8	88	88	88	88	NA	NA	NA	NA	NA	NA
8	8	88	88	88	88	NA	NA	NA	NA	NA	NA
8	8	88	88	88	88	NA	NA	NA	NA	NA	NA
8	8	88	88	88	88	NA	NA	NA	NA	NA	NA
8	8	88	88	88	88	NA	NA	NA	NA	NA	NA
8	8	88	88	88	88	NA	NA	NA	NA	NA	NA
8	8	88	88	88	88	NA	NA	NA	NA	NA	NA
8	8	88	88	88	88	NA	NA	NA	NA	NA	NA
8	8	88	88	88	88	NA	NA	NA	NA	NA	NA
8	8	88	88	88	88	NA	NA	NA	NA	NA	NA
8	8	88	88	88	88	NA	NA	NA	NA	NA	NA
8	8	88	88	88	88	NA	NA	NA	NA	NA	NA
8	8	88	88	88	88	NA	NA	NA	NA	NA	NA
8	8	88	88	88	88	NA	NA	NA	NA	NA	NA
8	8	88	88	88	88	NA	NA	NA	NA	NA	NA
8	8	88	88	88	88	NA	NA	NA	NA	NA	NA
8	8	88	88	88	88	NA	NA	NA	NA	NA	NA
8	8	88	88	88	88	NA	NA	NA	NA	NA	NA
8	8	88	88	88	88	NA	NA	NA	NA	NA	NA

8	8	88	88	88	88	NA	NA	NA	NA	NA	NA
8	8	88	88	88	88	NA	NA	NA	NA	NA	NA
8	8	88	88	88	88	NA	NA	NA	NA	NA	NA
8	8	88	88	88	88	NA	NA	NA	NA	NA	NA
8	8	88	88	88	88	NA	NA	NA	NA	NA	NA
8	8	88	88	88	88	NA	NA	NA	NA	NA	NA
8	8	88	88	88	88	NA	NA	NA	NA	NA	NA
8	8	88	88	88	88	NA	NA	NA	NA	NA	NA
8	8	88	88	88	88	NA	NA	NA	NA	NA	NA
8	8	88	88	88	88	NA	NA	NA	NA	NA	NA
8	8	88	88	88	88	NA	NA	NA	NA	NA	NA
8	8	88	88	88	88	NA	NA	NA	NA	NA	NA
8	8	88	88	88	88	NA	NA	NA	NA	NA	NA
8	8	88	88	88	88	NA	NA	NA	NA	NA	NA
8	8	88	88	88	88	NA	NA	NA	NA	NA	NA
8	8	88	88	88	88	NA	NA	NA	NA	NA	NA
8	8	88	88	88	88	NA	NA	NA	NA	NA	NA
8	8	88	88	88	88	NA	NA	NA	NA	NA	NA

q37other2	q37other3	q38h	q38h1	q38h2	q38h3	q39t	q39t1	q39t2	q39t3	q40t	q40t1	q40t2	q40t3
NA	NA	2	88	88	88	Ev	88	88	88	Ye	88	88	88
NA	NA	2	88	88	88	Af	88	88	88	Ye	88	88	88
NA	NA	3	88	88	88	Ev	88	88	88	Ye	88	88	88
NA	NA	3	88	88	88	Mo	88	88	88	Ye	88	88	88
NA	NA	2	88	88	88	Mo	88	88	88	Ye	88	88	88
NA	NA	2	88	88	88	Ev	88	88	88	Ye	88	88	88
NA	NA	1	88	1	88	Mo	88	Ev	88	Ye	88	Ye	88
NA	NA	1	88	1	88	Mo	88	88	88	No	88	88	88
NA	NA	2	88	88	88	Mo	88	88	88	Ye	88	88	88
NA	NA	2	88	88	88	Ev	88	88	88	Ye	88	88	88
NA	NA	3	88	88	88	Mo	88	88	88	Ye	88	88	88
NA	NA	2	88	88	88	Ev	88	88	88	Ye	88	88	88
NA	NA	1	88	1	88	Mo	88	Ev	88	Ye	88	Ye	88
NA	NA	1	88	1	88	Mo	88	Ev	88	Ye	88	Ye	88
NA	NA	88	88	88	88	Af	88	88	88	Ye	88	88	88
NA	NA	3	88	88	88	Mo	88	88	88	Ye	88	88	88
NA	NA	3	88	88	88	Ev	88	88	88	Ye	88	88	88
NA	NA	3	88	88	88	Ev	88	88	88	Ye	88	88	88
NA	NA	1	88	1	88	Mo	88	Ev	88	Ye	88	Ye	88
NA	NA	2	88	88	88	Ev	88	88	88	Ye	88	88	88
NA	NA	2	88	88	88	Ev	88	88	88	Ye	88	88	88
NA	NA	1	88	1	88	Mo	88	Ev	88	Ye	88	Ye	88
NA	NA	2	88	88	88	Ev	88	88	88	Ye	88	88	88
NA	NA	3	88	88	88	Ev	88	88	88	Ye	88	88	88
NA	NA	3	88	88	88	Ev	88	88	88	Ye	88	88	88
NA	NA	2	88	88	88	Ev	88	88	88	Ye	88	88	88
NA	NA	1	88	88	88	Af	88	88	88	Ye	88	88	88
NA	NA	88	88	88	88	88	88	88	88	88	88	88	88
NA	NA	1	88	88	88	Mo	88	88	88	Ye	88	88	88
NA	NA	1	88	88	88	Af	88	88	88	Ye	88	88	88
NA	NA	2	88	88	88	Mo	88	88	88	Ye	88	88	88
NA	NA	2	88	88	88	Mo	88	88	88	Ye	88	88	88
NA	NA	1	88	88	88	Ev	88	88	88	Ye	88	88	88
NA	NA	1	88	1	88	Mo	88	Ev	88	Ye	88	Ye	88
NA	NA	1	88	1	88	Mo	88	Ev	88	Ye	88	Ye	88
NA	NA	1	88	1	88	Mo	88	Ev	88	Ye	88	Ye	88
NA	NA	1	88	1	88	Mo	88	Ev	88	Ye	88	Ye	88
NA	NA	1	88	1	88	Mo	88	Ev	88	Ye	88	Ye	88
NA	NA	1	88	1	88	Mo	88	Ev	88	Ye	88	Ye	88
NA	NA	1	88	1	88	Mo	88	Ev	88	Ye	88	Ye	88
NA	NA	1	88	1	88	Mo	88	Ev	88	Ye	88	Ye	88
NA	NA	1	88	1	88	Mo	88	Ev	88	Ye	88	Ye	88
NA	NA	88	88	88	88	88	88	88	88	88	88	88	88
NA	NA	2	88	88	88	Ev	88	88	88	Ye	88	88	88
NA	NA	1	88	88	88	Mo	88	88	88	Ye	88	88	88
NA	NA	1	88	1	88	Mo	88	Ev	88	Ye	88	Ye	88

NA	NA	2	88	88	88	Mo	88	88	88	Ye	88	88	88
NA	NA	1	88	88	88	Mo	88	88	88	Ye	88	88	88
NA	NA	3	88	88	88	Mo	88	88	88	Ye	88	88	88
NA	NA	1	88	1	88	Mo	88	Ev	88	Ye	88	Ye	88
NA	NA	2	88	88	88	Ev	88	88	88	Ye	88	88	88
NA	NA	3	88	88	88	Mo	88	88	88	Ye	88	88	88
NA	NA	2	88	88	88	Ev	88	88	88	Ye	88	88	88
NA	NA	2	88	88	88	Ev	88	88	88	Ye	88	88	88
NA	NA	3	88	88	88	Ev	88	88	88	Ye	88	88	88
NA	NA	3	88	88	88	Ev	88	88	88	Ye	88	88	88
NA	NA	2	88	88	88	Ev	88	88	88	Ye	88	88	88
NA	NA	2	88	88	88	Mo	88	88	88	Ye	88	88	88
NA	NA	2	88	88	88	Ev	88	88	88	Ye	88	88	88
NA	NA	2	88	88	88	Mo	88	88	88	Ye	88	88	88
NA	NA	3	88	88	88	Ev	88	88	88	Ye	88	88	88
NA	NA	2	88	88	88	Mo	88	88	88	Ye	88	88	88
NA	NA	2	88	88	88	Mo	88	88	88	Ye	88	88	88
NA	NA	2	88	88	88	Ev	88	88	88	Ye	88	88	88
NA	NA	2	88	88	88	Mo	88	88	88	Ye	88	88	88
NA	NA	1	88	1	88	Mo	88	Ev	88	Ye	88	Ye	88
NA	NA	1	88	88	88	Ev	88	88	88	Ye	88	88	88
NA	NA	2	88	88	88	Mo	88	88	88	Ye	88	88	88
NA	NA	88	88	88	88	88	88	88	88	88	88	88	88
NA	NA	3	88	88	88	Ev	88	88	88	Ye	88	88	88
NA	NA	2	88	88	88	Ev	88	88	88	Ye	88	88	88
NA	NA	3	88	88	88	Ev	88	88	88	Ye	88	88	88
NA	NA	2	88	88	88	Mo	88	88	88	Ye	88	88	88
NA	NA	3	88	88	88	Ev	88	88	88	Ye	88	88	88
NA	NA	2	88	88	88	Af	88	88	88	Ye	88	88	88
NA	NA	2	88	88	88	Mo	88	88	88	Ye	88	88	88
NA	NA	2	88	88	88	Ev	88	88	88	No	88	88	88
NA	NA	2	88	88	88	Mo	88	88	88	Ye	88	88	88
NA	NA	3	88	88	88	Ev	88	88	88	Ye	88	88	88
NA	NA	3	88	88	88	Af	88	88	88	Ye	88	88	88
NA	NA	3	88	88	88	Af	88	88	88	Ye	88	88	88
NA	NA	2	88	88	88	Af	88	88	88	Ye	88	88	88
NA	NA	2	88	88	88	Mo	88	88	88	Ye	88	88	88
NA	NA	2	88	88	88	Ev	88	88	88	No	88	88	88
NA	NA	2	88	88	88	Ev	88	88	88	Ye	88	88	88
NA	NA	2	88	88	88	Mo	88	88	88	Ye	88	88	88
NA	NA	1	88	1	88	Mo	88	Ev	88	Ye	88	Ye	88
NA	NA	3	88	88	88	Mo	88	88	88	Ye	88	88	88
NA	NA	1	88	88	88	Mo	88	88	88	Ye	88	88	88
NA	NA	1	88	88	88	Mo	88	88	88	Ye	88	88	88
NA	NA	2	88	88	88	Af	88	88	88	Ye	88	88	88
NA	NA	2	88	88	88	Mo	88	88	88	Ye	88	88	88
NA	NA	3	88	88	88	Mo	88	88	88	Ye	88	88	88

NA	NA	3	88	88	88	Mo	88	88	88	Ye	88	88	88
NA	NA	2	88	88	88	Mo	88	88	88	Ye	88	88	88
NA	NA	3	88	88	88	Ev	88	88	88	Ye	88	88	88
NA	NA	88	88	88	88	88	88	88	88	88	88	88	88
NA	NA	2	88	88	88	Af	88	88	88	Ye	88	88	88
NA	NA	2	88	88	88	Ev	88	88	88	Ye	88	88	88
NA	NA	2	88	88	88	Ev	88	88	88	Ye	88	88	88
NA	NA	2	88	88	88	Ev	88	88	88	Ye	88	88	88
NA	NA	2	88	88	88	Af	88	88	88	Ye	88	88	88
NA	NA	1	88	88	88	Ev	88	88	88	Ye	88	88	88
NA	NA	2	88	88	88	Mo	88	88	88	Ye	88	88	88
NA	NA	2	88	88	88	Af	88	88	88	Ye	88	88	88
NA	NA	2	88	88	88	Mo	88	88	88	Ye	88	88	88
NA	NA	2	88	88	88	Mo	88	88	88	No	88	88	88
NA	NA	3	88	88	88	Mo	88	88	88	Ye	88	88	88
NA	NA	2	88	88	88	Mo	88	88	88	Ye	88	88	88
NA	NA	3	88	88	88	Mo	88	88	88	Ye	88	88	88
NA	NA	3	88	88	88	Mo	88	88	88	Ye	88	88	88
NA	NA	2	88	88	88	Af	88	88	88	Ye	88	88	88
NA	NA	2	88	88	88	Mo	88	88	88	Ye	88	88	88
NA	NA	2	88	88	88	Ev	88	88	88	Ye	88	88	88
NA	NA	2	88	88	88	Ev	88	88	88	Ye	88	88	88
NA	NA	2	88	88	88	Ev	88	88	88	Ye	88	88	88
NA	NA	2	88	88	88	Ev	88	88	88	Ye	88	88	88
NA	NA	2	88	88	88	Mo	88	88	88	Ye	88	88	88
NA	NA	2	88	88	88	Ev	88	88	88	Ye	88	88	88
NA	NA	2	88	88	88	Mo	88	88	88	Ye	88	88	88
NA	NA	3	88	88	88	Mo	88	88	88	Ye	88	88	88
NA	NA	3	88	88	88	Mo	88	88	88	Ye	88	88	88
NA	NA	1	88	88	88	Af	88	88	88	Ye	88	88	88
NA	NA	1	88	1	88	Mo	88	Ev	88	Ye	88	Ye	88
NA	NA	2	88	88	88	Mo	88	88	88	Ye	88	88	88
NA	NA	2	88	88	88	Mo	88	88	88	Ye	88	88	88
NA	NA	1	88	88	88	Mo	88	88	88	Ye	88	88	88
NA	NA	1	88	1	88	Mo	88	Ev	88	Ye	88	Ye	88
NA	NA	2	88	88	88	Mo	88	88	88	Ye	88	88	88
NA	NA	2	88	88	88	Mo	88	88	88	Ye	88	88	88
NA	NA	2	88	88	88	Mo	88	88	88	Ye	88	88	88
NA	NA	3	88	88	88	Mo	88	88	88	Ye	88	88	88
NA	NA	3	88	88	88	Mo	88	88	88	Ye	88	88	88
NA	NA	2	88	88	88	Mo	88	88	88	Ye	88	88	88
NA	NA	3	88	88	88	Mo	88	88	88	Ye	88	88	88
NA	NA	3	88	88	88	Mo	88	88	88	Ye	88	88	88
NA	NA	2	88	88	88	Mo	88	88	88	Ye	88	88	88
NA	NA	2	88	88	88	Mo	88	88	88	Ye	88	88	88
NA	NA	3	88	88	88	Mo	88	88	88	Ye	88	88	88
NA	NA	3	88	88	88	Mo	88	88	88	Ye	88	88	88

NA	NA	3	88	88	88	Af	88	88	88	Ye	88	88	88
NA	NA	1	88	88	88	Mo	88	88	88	Ye	88	88	88
NA	NA	1	88	88	88	Mo	88	88	88	Ye	88	88	88
NA	NA	3	88	88	88	Mo	88	88	88	Ye	88	88	88
NA	NA	1	88	88	88	Mo	88	88	88	Ye	88	88	88
NA	NA	3	88	88	88	Mo	88	88	88	Ye	88	88	88
NA	NA	2	88	88	88	Mo	88	88	88	Ye	88	88	88
NA	NA	2	88	88	88	Mo	88	88	88	Ye	88	88	88
NA	NA	2	88	88	88	Mo	88	88	88	Ye	88	88	88
NA	NA	88	88	88	88	88	88	88	88	88	88	88	88
NA	NA	88	88	88	88	88	88	88	88	88	88	88	88
NA	NA	2	88	88	88	Ev	88	88	88	Ye	88	88	88
NA	NA	2	88	88	88	Mo	88	88	88	Ye	88	88	88
NA	NA	2	88	88	88	Mo	88	88	88	Ye	88	88	88
NA	NA	2	88	88	88	Ev	88	88	88	Ye	88	88	88
NA	NA	2	88	88	88	Mo	88	88	88	Ye	88	88	88
NA	NA	2	88	88	88	Mo	88	88	88	Ye	88	88	88
NA	NA	2	88	88	88	Mo	88	88	88	Ye	88	88	88
NA	NA	2	88	88	88	Ev	88	88	88	Ye	88	88	88
NA	NA	2	88	88	88	Ev	88	88	88	Ye	88	88	88
NA	NA	2	88	88	88	Ev	88	88	88	Ye	88	88	88
NA	NA	2	88	88	88	Mo	88	88	88	Ye	88	88	88
NA	NA	2	88	88	88	Mo	88	88	88	Ye	88	88	88
NA	NA	2	88	88	88	Ev	88	88	88	Ye	88	88	88
NA	NA	2	88	88	88	Mo	88	88	88	Ye	88	88	88
NA	NA	2	88	88	88	Mo	88	88	88	Ye	88	88	88
NA	NA	1	88	88	88	Mo	88	88	88	Ye	88	88	88
NA	NA	1	88	88	88	Af	88	88	88	Ye	88	88	88
NA	NA	2	88	88	88	Af	88	88	88	Ye	88	88	88
NA	NA	1	88	1	88	Mo	88	Ev	88	Ye	88	Ye	88
NA	NA	1	88	88	88	Af	88	88	88	Ye	88	88	88
NA	NA	2	88	88	88	Ev	88	88	88	Ye	88	88	88
NA	NA	2	88	88	88	Mo	88	88	88	Ye	88	88	88
NA	NA	2	88	88	88	Mo	88	88	88	Ye	88	88	88
NA	NA	2	88	88	88	Mo	88	88	88	Ye	88	88	88
NA	NA	3	88	88	88	Mo	88	88	88	Ye	88	88	88
NA	NA	2	88	88	88	Ev	88	88	88	Ye	88	88	88
NA	NA	1	88	88	88	Af	88	88	88	Ye	88	88	88
NA	NA	2	88	88	88	Ev	88	88	88	Ye	88	88	88
NA	NA	1	88	88	88	Mo	88	88	88	Ye	88	88	88
NA	NA	2	88	88	88	Mo	88	88	88	Ye	88	88	88
NA	NA	1	88	88	88	Af	88	88	88	Ye	88	88	88
NA	NA	3	88	88	88	Af	88	88	88	Ye	88	88	88
NA	NA	2	88	88	88	Mo	88	88	88	Ye	88	88	88
NA	NA	2	88	88	88	Mo	88	88	88	Ye	88	88	88
NA	NA	2	88	88	88	Mo	88	88	88	Ye	88	88	88
NA	NA	2	88	88	88	Ev	88	88	88	Ye	88	88	88

NA	NA	2	88	88	88	Ev	88	88	88	Ye	88	88	88
NA	NA	1	88	88	88	Mo	88	88	88	Ye	88	88	88
NA	NA	3	88	88	88	Ev	88	88	88	Ye	88	88	88
NA	NA	2	88	88	88	Ev	88	88	88	Ye	88	88	88
NA	NA	2	88	88	88	Ev	88	88	88	Ye	88	88	88
NA	NA	2	88	88	88	Mo	88	88	88	Ye	88	88	88
NA	NA	1	88	88	88	Mo	88	88	88	Ye	88	88	88
NA	NA	2	88	88	88	Mo	88	88	88	Ye	88	88	88
NA	NA	2	88	88	88	Af	88	88	88	Ye	88	88	88
NA	NA	2	88	88	88	Mo	88	88	88	Ye	88	88	88
NA	NA	2	88	88	88	Mo	88	Af	88	Ye	88	88	88
NA	NA	2	88	88	88	Mo	88	88	88	Ye	88	88	88
NA	NA	1	88	88	88	Mo	88	88	88	Ye	88	88	88
NA	NA	2	88	88	88	Af	88	88	88	Ye	88	88	88
NA	NA	3	88	88	88	Mo	88	88	88	Ye	88	88	88
NA	NA	2	88	88	88	Mo	88	88	88	Ye	88	88	88
NA	NA	2	88	88	88	Mo	88	88	88	Ye	88	88	88
NA	NA	2	88	88	88	Mo	88	88	88	Ye	88	88	88
NA	NA	1	88	88	88	Mo	88	88	88	Ye	88	88	88
NA	NA	3	88	88	88	Mo	88	88	88	Ye	88	88	88
NA	NA	2	88	88	88	Ev	88	88	88	Ye	88	88	88
NA	NA	2	88	88	88	Ev	88	88	88	Ye	88	88	88
NA	NA	1	88	88	88	Mo	88	88	88	Ye	88	88	88
NA	NA	2	88	88	88	Ev	88	88	88	Ye	88	88	88
NA	NA	3	88	88	88	Mo	88	88	88	Ye	88	88	88
NA	NA	2	88	88	88	Mo	88	88	88	Ye	88	88	88
NA	NA	2	88	88	88	Mo	88	88	88	Ye	88	88	88
NA	NA	2	88	88	88	Mo	88	88	88	Ye	88	88	88
NA	NA	3	88	88	88	Ev	88	88	88	Ye	88	88	88
NA	NA	3	88	88	88	Mo	88	88	88	Ye	88	88	88
NA	NA	3	88	88	88	Ev	88	88	88	Ye	88	88	88
NA	NA	2	88	88	88	Ev	88	88	88	Ye	88	88	88
NA	NA	3	88	88	88	Mo	88	88	88	Ye	88	88	88
NA	NA	3	88	88	88	Af	88	88	88	Ye	88	88	88
NA	NA	3	88	88	88	Af	88	88	88	Ye	88	88	88
NA	NA	3	88	88	88	Ev	88	88	88	Ye	88	88	88
NA	NA	3	88	88	88	Ev	88	88	88	Ye	88	88	88
NA	NA	2	88	88	88	Mo	88	88	88	Ye	88	88	88
NA	NA	2	88	88	88	Mo	88	88	88	Ye	88	88	88
NA	NA	2	88	88	88	Mo	88	88	88	Ye	88	88	88
NA	NA	2	88	88	88	Mo	88	88	88	Ye	88	88	88
NA	NA	2	88	88	88	Mo	88	88	88	Ye	88	88	88
NA	NA	2	88	88	88	Mo	88	88	88	Ye	88	88	88
NA	NA	1	88	1	88	Mo	88	Ev	88	Ye	88	Ye	88
NA	NA	3	88	88	88	Ev	88	88	88	Ye	88	88	88
NA	NA	3	88	88	88	Ev	88	88	88	Ye	88	88	88
NA	NA	2	88	88	88	Ev	88	88	88	Ye	88	88	88

NA	NA	2	88	88	88	Ev	88	88	88	Ye	88	88	88
NA	NA	1	88	88	88	Mo	88	88	88	Ye	88	88	88
NA	NA	1	88	88	88	Mo	88	88	88	Ye	88	88	88
NA	NA	2	88	88	88	Af	88	88	88	Ye	88	88	88
NA	NA	2	88	88	88	Af	88	88	88	Ye	88	88	88
NA	NA	2	88	88	88	Ev	88	88	88	Ye	88	88	88
NA	NA	2	88	88	88	Ev	88	88	88	Ye	88	88	88
NA	NA	2	88	88	88	Af	88	88	88	Ye	88	88	88
NA	NA	2	88	88	88	Af	88	88	88	Ye	88	88	88
NA	NA	3	88	88	88	Mo	88	88	88	Ye	88	88	88
NA	NA	2	88	88	88	Mo	88	88	88	Ye	88	88	88
NA	NA	1	88	1	88	Mo	88	Ev	88	Ye	88	Ye	88
NA	NA	2	88	88	88	Ev	88	88	88	Ye	88	88	88
NA	NA	2	88	88	88	Mo	88	88	88	Ye	88	88	88
NA	NA	2	88	8	88	Mo	88	88	88	Ye	88	88	88
NA	NA	2	88	88	88	Mo	88	88	88	Ye	88	88	88
NA	NA	2	88	88	88	Mo	88	88	88	Ye	88	88	88
NA	NA	2	88	88	88	Mo	88	88	88	Ye	88	88	88
NA	NA	2	88	88	88	Mo	88	88	88	Ye	88	88	88
NA	NA	2	88	88	88	Ev	88	88	88	Ye	88	88	88
NA	NA	2	88	88	88	Mo	88	88	88	Ye	88	88	88
NA	NA	2	88	88	88	Mo	88	88	88	Ye	88	88	88
NA	NA	3	88	88	88	Mo	88	88	88	Ye	88	88	88
NA	NA	2	88	88	88	Mo	88	88	88	Ye	88	88	88
NA	NA	1	88	1	88	Mo	88	Ev	88	Ye	88	Ye	88
NA	NA	2	88	88	88	Mo	88	88	88	Ye	88	88	88
NA	NA	2	88	88	88	Ev	88	88	88	Ye	88	88	88
NA	NA	1	88	88	88	Mo	88	88	88	Ye	88	88	88
NA	NA	2	88	88	88	Mo	88	88	88	Ye	88	88	88
NA	NA	2	88	88	88	Mo	88	88	88	Ye	88	88	88
NA	NA	2	88	88	88	Ev	88	88	88	Ye	88	88	88
NA	NA	2	88	88	88	Mo	88	88	88	Ye	88	88	88
NA	NA	2	88	88	88	Mo	88	88	88	Ye	88	88	88
NA	NA	2	88	88	88	Mo	88	88	88	Ye	88	88	88
NA	NA	1	88	1	88	Mo	88	Ev	88	Ye	88	Ye	88
NA	NA	1	88	1	88	Mo	88	Ev	88	Ye	88	Ye	88
NA	NA	1	88	88	88	Mo	88	88	88	Ye	88	88	88
NA	NA	2	88	88	88	Ev	88	88	88	Ye	88	88	88
NA	NA	2	88	88	88	Ev	88	88	88	Ye	88	88	88
NA	NA	2	88	88	88	Ev	88	88	88	Ye	88	88	88
NA	NA	1	88	1	88	Mo	88	Ev	88	88	88	88	88
NA	NA	1	88	1	88	Mo	88	Ev	88	Ye	88	Ye	88
NA	NA	1	88	1	88	Mo	88	Mo	88	Ye	88	Do	88
NA	NA	2	88	88	88	88	88	88	88	88	88	88	88
NA	NA	2	88	88	88	Mo	88	88	88	Ye	88	88	88
NA	NA	2	88	88	88	Af	88	88	88	Ye	88	88	88
NA	NA	2	88	88	88	Af	88	88	88	Ye	88	88	88

NA	NA	1	88	88	88	Mo	88	88	88	Ye	88	88	88
NA	NA	2	88	88	88	Mo	88	88	88	Ye	88	88	88
NA	NA	2	88	88	88	Mo	88	88	88	Ye	88	88	88
NA	NA	2	88	88	88	Af	88	88	88	Ye	88	88	88
NA	NA	2	88	88	88	Mo	88	88	88	Ye	88	88	88
NA	NA	2	88	88	88	Mo	88	88	88	Ye	88	88	88
NA	NA	2	88	88	88	Mo	88	88	88	Ye	88	88	88
NA	NA	2	88	88	88	Mo	88	88	88	Ye	88	88	88
NA	NA	2	88	88	88	Mo	88	88	88	Ye	88	88	88
NA	NA	2	88	88	88	Mo	88	88	88	Ye	88	88	88
NA	NA	1	88	1	88	Mo	88	Ev	88	Ye	88	Ye	88
NA	NA	2	88	88	88	Mo	88	88	88	Ye	88	88	88
NA	NA	2	88	88	88	Mo	88	88	88	Ye	88	88	88
NA	NA	1	88	88	88	Mo	88	88	88	Ye	88	88	88
NA	NA	2	88	88	88	Ev	88	88	88	Ye	88	88	88
NA	NA	2	88	88	88	Mo	88	88	88	Ye	88	88	88
NA	NA	2	88	88	88	Mo	88	88	88	Ye	88	88	88
NA	NA	2	88	88	88	Ev	88	88	88	Ye	88	88	88

q41t	q41t1	q41t2	q41t3	q42v	q42v1	q42v2	q42v3	q43i	q43i1	q43i2	q43i3	q44i	q45w	q45w1	q45w2
No	88	88	88	No	88	88	88	88	88	88	88	88	Ye	88	88
No	88	88	88	No	88	88	88	88	88	88	88	88	4	88	88
No	88	88	88	No	88	88	88	88	88	88	88	88	88	88	88
No	88	No	88	No	88	88	88	88	88	88	88	88	88	88	88
No	88	88	88	Ye	88	88	88	No	88	88	88	88	No	88	88
No	88	88	88	No	88	88	88	88	88	88	88	88	Ye	88	88
No	88	No	88	No	88	88	88	88	88	88	88	88	No	88	No
No	88	88	88	88	88	88	88	88	88	88	88	Af	No	88	No
No	88	88	88	Ye	88	88	88	No	88	88	88	Mo	88	88	88
No	88	88	88	No	88	88	88	88	88	88	88	88	Ye	88	88
No	88	88	88	No	88	88	88	88	88	88	88	88	88	88	88
No	88	88	88	No	88	88	88	88	88	88	88	88	Ye	88	88
No	88	No	88	No	88	88	88	88	88	88	88	88	No	88	No
No	88	No	88	No	88	88	88	88	88	88	88	88	Ye	88	Ye
No	88	88	88	No	88	88	88	88	88	88	88	88	No	88	88
No	88	88	88	No	88	88	88	88	88	88	88	88	88	88	88
No	88	88	88	No	88	88	88	88	88	88	88	88	88	88	88
No	88	88	88	No	88	88	88	88	88	88	88	88	88	88	88
No	88	No	88	No	88	88	88	88	88	88	88	88	88	88	88
No	88	88	88	No	88	88	88	88	88	88	88	88	Ye	88	88
No	88	88	88	No	88	88	88	88	88	88	88	88	Ye	88	88
No	88	No	88	No	88	No	88	88	88	88	88	88	Ye	88	Ye
No	88	88	88	No	88	88	88	88	88	88	88	88	Ye	88	88
No	88	88	88	No	88	88	88	88	88	88	88	88	88	88	88
No	88	88	88	No	88	88	88	88	88	88	88	88	Ye	88	88
No	88	88	88	No	88	88	88	88	88	88	88	88	Ye	88	88
No	88	88	88	No	88	88	88	88	88	88	88	88	Ye	88	88
88	88	88	88	88	88	88	88	88	88	88	88	88	88	88	88
No	88	88	88	No	88	88	88	88	88	88	88	88	88	88	88
Ye	88	88	88	No	88	88	88	88	88	88	88	88	Ye	88	88
No	88	88	88	No	88	88	88	88	88	88	88	88	4	88	88
Ye	88	88	88	Ye	88	88	88	No	88	88	88	88	No	88	88
No	88	88	88	No	88	88	88	88	88	88	88	88	Ye	88	88
No	88	No	88	No	88	88	88	88	88	88	88	88	88	88	88
No	88	No	88	No	88	88	88	88	88	88	88	88	88	88	88
No	88	No	88	No	88	88	88	88	88	88	88	88	88	88	88
No	88	No	88	No	88	88	88	88	88	88	88	88	88	88	88
No	88	No	88	No	88	88	88	88	88	88	88	88	Ye	88	Ye
No	88	No	88	No	88	No	88	88	88	88	88	88	Ye	88	Ye
No	88	No	88	No	88	88	88	88	88	88	88	88	4	88	4
No	88	No	88	No	88	88	88	88	88	88	88	88	4	88	4
No	88	No	88	No	88	88	88	88	88	88	88	88	Ye	88	Ye
88	88	88	88	88	88	88	88	88	88	88	88	88	88	88	88
No	88	88	88	No	88	88	88	88	88	88	88	88	4	88	88
Ye	88	88	88	Ye	88	88	88	No	88	88	88	88	Ye	88	88
No	88	No	88	No	88	88	88	88	88	88	88	88	4	88	88

No	88	88	88	No	88	88	88	88	88	88	88	88	4	88	88
Ye	88	88	88	No	88	88	88	88	88	88	88	88	Ye	88	88
No	88	88	88	No	88	88	88	88	88	88	88	88	88	88	88
No	88	No	88	No	88	88	88	88	88	88	88	88	Ye	88	Ye
No	88	88	88	No	88	88	88	88	88	88	88	88	4	88	88
No	88	88	88	No	88	88	88	88	88	88	88	88	88	88	88
No	88	88	88	No	88	88	88	88	88	88	88	88	Ye	88	88
No	88	88	88	No	88	88	88	88	88	88	88	88	Ye	88	88
No	88	88	88	No	88	88	88	88	88	88	88	88	88	88	88
No	88	88	88	No	88	88	88	88	88	88	88	88	88	88	88
No	88	88	88	No	88	88	88	88	88	88	88	88	4	88	88
No	88	88	88	No	88	88	88	88	88	88	88	88	Ye	88	88
No	88	88	88	No	88	88	88	88	88	88	88	88	Ye	88	88
No	88	88	88	No	88	88	88	88	88	88	88	88	Ye	88	88
No	88	88	88	No	88	88	88	88	88	88	88	88	88	88	88
No	88	88	88	No	88	88	88	88	88	88	88	88	4	88	88
Ye	88	88	88	No	88	88	88	88	88	88	88	88	Ye	88	88
No	88	88	88	No	88	88	88	88	88	88	88	88	Ye	88	88
Ye	88	88	88	No	88	88	88	88	88	88	88	88	Ye	88	88
No	88	No	88	No	88	88	88	88	88	88	88	88	Ye	88	Ye
Ye	88	88	88	Ye	88	88	88	No	88	88	88	88	Ye	88	88
No	88	88	88	No	88	88	88	88	88	88	88	88	Ye	88	88
88	88	88	88	88	88	88	88	88	88	88	88	88	88	88	88
No	88	88	88	No	88	88	88	88	88	88	88	88	88	88	88
No	88	88	88	No	88	88	88	88	88	88	88	88	Ye	88	88
No	88	88	88	No	88	88	88	88	88	88	88	88	88	88	88
No	88	88	88	No	88	88	88	88	88	88	88	88	4	88	88
No	88	88	88	No	88	88	88	88	88	88	88	88	88	88	88
No	88	88	88	No	88	88	88	88	88	88	88	88	No	88	88
No	88	88	88	No	88	88	88	88	88	88	88	88	Ye	88	88
No	88	88	88	Ye	88	88	88	No	88	88	88	88	Ye	88	88
No	88	88	88	No	88	88	88	88	88	88	88	88	Ye	88	88
No	88	88	88	No	88	88	88	88	88	88	88	88	88	88	88
No	88	88	88	No	88	88	88	88	88	88	88	88	88	88	88
No	88	88	88	No	88	88	88	88	88	88	88	88	88	88	88
No	88	88	88	No	88	88	88	88	88	88	88	88	Ye	88	88
No	88	88	88	No	88	88	88	88	88	88	88	88	Ye	88	88
No	88	88	88	No	88	88	88	88	88	88	88	88	No	88	88
No	88	88	88	No	88	88	88	88	88	88	88	88	Ye	88	88
Ye	88	88	88	No	88	88	88	88	88	88	88	88	Ye	88	88
No	88	No	88	No	88	88	88	88	88	88	88	88	Ye	88	Ye
No	88	88	88	No	88	88	88	88	88	88	88	88	Ye	88	88
Ye	88	88	88	Ye	88	88	88	No	88	88	88	88	Ye	88	88
Ye	88	88	88	Ye	88	88	88	No	88	88	88	88	Ye	88	88
No	88	88	88	Ye	88	88	88	Ye	88	88	88	88	Ye	88	88
No	88	88	88	No	88	88	88	88	88	88	88	88	Ye	88	88
No	88	88	88	No	88	88	88	88	88	88	88	88	88	88	88

No	88	88	88	No	88	88	88	88	88	88	88	88	88	88	88
No	88	88	88	No	88	88	88	88	88	88	88	88	4	88	88
No	88	88	88	No	88	88	88	88	88	88	88	88	88	88	88
88	88	88	88	88	88	88	88	88	88	88	88	88	88	88	88
No	88	88	88	No	88	88	88	88	88	88	88	88	Ye	88	88
No	88	88	88	No	88	88	88	88	88	88	88	88	Ye	88	88
No	88	88	88	No	88	88	88	88	88	88	88	88	Ye	88	88
No	88	88	88	No	88	88	88	88	88	88	88	88	Ye	88	88
No	88	88	88	No	88	88	88	88	88	88	88	88	Ye	88	88
No	88	88	88	No	88	88	88	88	88	88	88	88	Ye	88	88
No	88	88	88	No	88	88	88	88	88	88	88	88	Ye	88	88
No	88	88	88	No	88	88	88	88	88	88	88	88	Ye	88	88
No	88	88	88	No	88	88	88	88	88	88	88	88	Ye	88	88
No	88	88	88	No	88	88	88	No	88	88	88	88	No	88	88
No	88	88	88	No	88	88	88	88	88	88	88	88	88	88	88
No	88	88	88	No	88	88	88	88	88	88	88	88	Ye	88	88
No	88	88	88	No	88	88	88	88	88	88	88	88	88	88	88
No	88	88	88	No	88	88	88	88	88	88	88	88	88	88	88
Ye	88	88	88	No	88	88	88	88	88	88	88	88	Ye	88	88
No	88	88	88	No	88	88	88	88	88	88	88	88	4	88	88
No	88	88	88	No	88	88	88	88	88	88	88	88	Ye	88	88
No	88	88	88	No	88	88	88	88	88	88	88	88	Ye	88	88
No	88	88	88	No	88	88	88	88	88	88	88	88	No	88	88
No	88	88	88	Ye	88	88	88	No	88	88	88	88	Ye	88	88
No	88	88	88	No	88	88	88	88	88	88	88	88	Ye	88	88
No	88	88	88	No	88	88	88	88	88	88	88	88	Ye	88	88
No	88	88	88	No	88	88	88	88	88	88	88	88	Ye	88	88
No	88	88	88	No	88	88	88	88	88	88	88	88	88	88	88
No	88	88	88	No	88	88	88	88	88	88	88	88	88	88	88
Ye	88	88	88	No	88	88	88	88	88	88	88	88	Ye	88	88
No	88	No	88	No	88	88	88	88	88	88	88	88	Ye	88	Ye
No	88	88	88	No	88	88	88	88	88	88	88	88	4	88	88
No	88	88	88	No	88	88	88	88	88	88	88	88	4	88	88
No	88	88	88	No	88	88	88	88	88	88	88	88	Ye	88	88
No	88	No	88	No	88	88	88	88	88	88	88	88	Ye	88	Ye
No	88	88	88	No	88	88	88	88	88	88	88	88	Ye	88	88
No	88	88	88	No	88	88	88	88	88	88	88	88	Ye	88	88
No	88	88	88	No	88	88	88	88	88	88	88	88	Ye	88	88
No	88	88	88	No	88	88	88	88	88	88	88	88	88	88	88
No	88	88	88	No	88	88	88	88	88	88	88	88	88	88	88
No	88	88	88	No	88	88	88	88	88	88	88	88	4	88	88
No	88	88	88	No	88	88	88	88	88	88	88	88	88	88	88
No	88	88	88	No	88	88	88	88	88	88	88	88	88	88	88
No	88	88	88	No	88	88	88	88	88	88	88	88	Ye	88	88
No	88	88	88	No	88	88	88	88	88	88	88	88	Ye	88	88
No	88	88	88	No	88	88	88	88	88	88	88	88	88	88	88
No	88	88	88	No	88	88	88	88	88	88	88	88	88	88	88

No	88	88	88	No	88	88	88	88	88	88	88	88	88	88	88
No	88	88	88	No	88	88	88	88	88	88	88	88	No	88	88
No	88	88	88	No	88	88	88	88	88	88	88	88	Ye	88	88
No	88	88	88	No	88	88	88	88	88	88	88	88	88	88	88
Ye	88	88	88	No	88	88	88	88	88	88	88	88	Ye	88	88
No	88	88	88	No	88	88	88	88	88	88	88	88	88	88	88
Ye	88	88	88	No	88	88	88	88	88	88	88	88	Ye	88	88
No	88	88	88	No	88	88	88	88	88	88	88	88	Ye	88	88
Ye	88	88	88	No	88	88	88	88	88	88	88	88	Ye	88	88
88	88	88	88	88	88	88	88	88	88	88	88	88	88	88	88
88	88	88	88	88	88	88	88	88	88	88	88	88	88	88	88
No	88	88	88	No	88	88	88	88	88	88	88	88	Ye	88	88
No	88	88	88	No	88	88	88	88	88	88	88	88	Ye	88	88
No	88	88	88	No	88	88	88	88	88	88	88	88	Ye	88	88
No	88	88	88	No	88	88	88	88	88	88	88	88	Ye	88	88
No	88	88	88	No	88	88	88	88	88	88	88	88	No	88	88
No	88	88	88	No	88	88	88	88	88	88	88	88	Ye	88	88
No	88	88	88	No	88	88	88	88	88	88	88	88	Ye	88	88
No	88	88	88	No	88	88	88	88	88	88	88	88	4	88	88
No	88	88	88	No	88	88	88	88	88	88	88	88	Ye	88	88
No	88	88	88	No	88	88	88	88	88	88	88	88	Ye	88	88
No	88	88	88	No	88	88	88	88	88	88	88	88	Ye	88	88
No	88	88	88	No	88	88	88	88	88	88	88	88	Ye	88	88
No	88	88	88	Ye	88	88	88	No	88	88	88	88	Ye	88	88
No	88	88	88	No	88	88	88	88	88	88	88	88	4	88	88
No	88	88	88	No	88	88	88	88	88	88	88	88	Ye	88	88
No	88	88	88	No	88	88	88	88	88	88	88	88	Ye	88	88
No	88	88	88	No	88	88	88	88	88	88	88	88	Ye	88	88
No	88	88	88	No	88	88	88	88	88	88	88	88	Ye	88	88
No	88	No	88	No	88	88	88	88	88	88	88	88	Ye	88	88
Ye	88	88	88	No	88	88	88	88	88	88	88	88	Ye	88	88
No	88	88	88	No	88	88	88	88	88	88	88	88	Ye	88	88
No	88	88	88	No	88	88	88	88	88	88	88	88	Ye	88	88
No	88	88	88	No	88	88	88	88	88	88	88	88	Ye	88	88
No	88	88	88	No	88	88	88	88	88	88	88	88	Ye	88	88
No	88	88	88	No	88	88	88	88	88	88	88	88	88	88	88
No	88	88	88	No	88	88	88	88	88	88	88	88	Ye	88	88
No	88	88	88	No	88	88	88	88	88	88	88	88	Ye	88	88
No	88	88	88	No	88	88	88	88	88	88	88	88	No	88	88
Ye	88	88	88	No	88	88	88	88	88	88	88	88	Ye	88	88
No	88	88	88	No	88	88	88	88	88	88	88	88	Ye	88	88
Ye	88	88	88	No	88	88	88	88	88	88	88	88	Ye	88	88
No	88	88	88	No	88	88	88	88	88	88	88	88	88	88	88
No	88	88	88	Ye	88	88	88	No	88	88	88	88	Ye	88	88
No	88	88	88	No	88	88	88	88	88	88	88	88	Ye	88	88
Ye	88	88	88	No	88	88	88	88	88	88	88	88	Ye	88	88
No	88	88	88	No	88	88	88	88	88	88	88	88	Ye	88	88

No	88	88	88	No	88	88	88	88	88	88	88	88	Ye	88	88
No	88	88	88	Ye	88	88	88	No	88	88	88	88	Ye	88	88
No	88	88	88	No	88	88	88	88	88	88	88	88	Ye	88	88
No	88	88	88	No	88	88	88	88	88	88	88	88	Ye	88	88
No	88	88	88	No	88	88	88	88	88	88	88	88	Ye	88	88
No	88	88	88	No	88	88	88	88	88	88	88	88	4	88	88
Ye	88	88	88	No	88	88	88	88	88	88	88	88	Ye	88	88
No	88	88	88	No	88	88	88	88	88	88	88	88	Ye	88	88
No	88	88	88	No	88	88	88	88	88	88	88	88	Ye	88	88
No	88	88	88	No	88	88	88	88	88	88	88	88	Ye	88	88
No	88	88	88	No	88	88	88	88	88	88	88	88	Ye	88	88
No	88	88	88	No	88	88	88	88	88	88	88	88	Ye	88	88
No	88	88	88	No	88	88	88	88	88	88	88	88	Ye	88	88
No	88	88	88	No	88	88	88	88	88	88	88	88	Ye	88	88
No	88	88	88	No	88	88	88	88	88	88	88	88	88	88	88
No	88	88	88	No	88	88	88	88	88	88	88	88	Ye	88	88
No	88	88	88	No	88	88	88	88	88	88	88	88	No	88	88
No	88	88	88	No	88	88	88	88	88	88	88	88	Ye	88	88
Ye	88	88	88	No	88	88	88	88	88	88	88	88	Ye	88	88
No	88	88	88	No	88	88	88	88	88	88	88	88	88	88	88
No	88	88	88	No	88	88	88	88	88	88	88	88	88	88	88
No	88	88	88	No	88	88	88	88	88	88	88	88	Ye	88	88
Ye	88	88	88	No	88	88	88	88	88	88	88	88	Ye	88	88
No	88	88	88	No	88	88	88	88	88	88	88	88	Ye	88	88
No	88	88	88	No	88	88	88	88	88	88	88	88	88	88	88
No	88	88	88	No	88	88	88	88	88	88	88	88	Ye	88	88
No	88	88	88	No	88	88	88	88	88	88	88	88	Ye	88	88
No	88	88	88	No	88	88	88	88	88	88	88	88	Ye	88	88
No	88	88	88	No	88	88	88	88	88	88	88	88	88	88	88
No	88	88	88	No	88	88	88	88	88	88	88	88	88	88	88
No	88	88	88	No	88	88	88	88	88	88	88	88	88	88	88
No	88	88	88	No	88	88	88	88	88	88	88	88	Ye	88	88
No	88	88	88	No	88	88	88	88	88	88	88	88	88	88	88
No	88	88	88	No	88	88	88	88	88	88	88	88	88	88	88
No	88	88	88	No	88	88	88	88	88	88	88	88	88	88	88
No	88	88	88	No	88	88	88	88	88	88	88	88	88	88	88
No	88	88	88	No	88	88	88	88	88	88	88	88	4	88	88
Ye	88	88	88	No	88	88	88	88	88	88	88	88	Ye	88	88
No	88	88	88	No	88	88	88	88	88	88	88	88	4	88	88
No	88	88	88	No	88	88	88	88	88	88	88	88	Ye	88	88
No	88	88	88	Ye	88	88	88	No	88	88	88	88	Ye	88	88
No	88	88	88	No	88	88	88	88	88	88	88	88	4	88	88
No	88	88	88	No	88	88	88	88	88	88	88	88	Ye	88	88
No	88	No	88	No	88	88	88	88	88	88	88	88	Ye	88	Ye
No	88	88	88	No	88	88	88	88	88	88	88	88	88	88	88
No	88	88	88	No	88	88	88	88	88	88	88	88	88	88	88
No	88	88	88	No	88	88	88	88	88	88	88	88	Ye	88	88

No	88	88	88	No	88	88	88	88	88	88	88	88	4	88	88
Ye	88	88	88	No	88	88	88	88	88	88	88	88	Ye	88	88
No	88	88	88	No	88	88	88	88	88	88	88	88	Ye	88	88
No	88	88	88	No	88	88	88	88	88	88	88	88	Ye	88	88
No	88	88	88	No	88	88	88	88	88	88	88	88	Ye	88	88
No	88	88	88	No	88	88	88	88	88	88	88	88	Ye	88	88
No	88	88	88	No	88	88	88	88	88	88	88	88	Ye	88	88
No	88	88	88	No	88	88	88	88	88	88	88	88	Ye	88	88
No	88	88	88	No	88	88	88	88	88	88	88	88	Ye	88	88
No	88	88	88	No	88	88	88	88	88	88	88	88	88	88	88
No	88	88	88	No	88	88	88	88	88	88	88	88	No	88	88
No	88	No	88	No	88	88	88	88	88	88	88	88	Ye	88	Ye
No	88	88	88	No	88	88	88	88	88	88	88	88	Ye	88	88
No	88	88	88	No	88	88	88	88	88	88	88	88	Ye	88	88
No	88	88	88	No	88	88	88	88	88	88	88	88	Ye	88	88
No	88	88	88	No	88	88	88	88	88	88	88	88	Ye	88	88
No	88	88	88	No	88	88	88	88	88	88	88	88	Ye	88	88
No	88	88	88	No	88	88	88	88	88	88	88	88	Ye	88	88
No	88	88	88	No	88	88	88	88	88	88	88	88	Ye	88	88
No	88	88	88	No	88	88	88	88	88	88	88	88	Ye	88	88
No	88	88	88	No	88	88	88	88	88	88	88	88	Ye	88	88
No	88	88	88	No	88	88	88	88	88	88	88	88	Ye	88	88
No	88	88	88	No	88	88	88	88	88	88	88	88	88	88	88
No	88	88	88	No	88	88	88	88	88	88	88	88	Ye	88	88
No	88	No	88	No	88	88	88	88	88	88	88	88	Ye	88	Ye
No	88	88	88	No	88	88	88	88	88	88	88	88	Ye	88	88
No	88	88	88	No	88	88	88	88	88	88	88	88	Ye	88	88
Ye	88	88	88	No	88	88	88	88	88	88	88	88	Ye	88	88
Ye	88	88	88	Ye	88	88	88	No	88	88	88	88	Ye	88	88
Ye	88	88	88	No	88	88	88	88	88	88	88	88	Ye	88	88
No	88	88	88	No	88	88	88	88	88	88	88	88	Ye	88	88
No	88	88	88	No	88	88	88	88	88	88	88	88	Ye	88	88
No	88	88	88	No	88	88	88	88	88	88	88	88	Ye	88	88
No	88	88	88	No	88	88	88	88	88	88	88	88	Ye	88	88
No	88	No	88	No	88	88	88	88	88	88	88	88	Ye	88	Ye
No	88	No	88	No	88	88	88	88	88	88	88	88	Ye	88	Ye
No	88	88	88	No	88	88	88	88	88	88	88	88	Ye	88	88
No	88	88	88	No	88	88	88	88	88	88	88	88	Ye	88	88
No	88	88	88	No	88	88	88	88	88	88	88	88	4	88	88
No	88	88	88	No	88	88	88	88	88	88	88	88	Ye	88	88
88	88	88	88	Ye	88	Ye	88	Ye	88	No	88	88	Ye	88	Ye
No	88	No	88	No	88	88	88	88	88	88	88	88	No	88	No
No	88	No	88	No	88	88	88	88	88	88	88	88	4	88	4
88	88	88	88	88	88	88	88	88	88	88	88	88	88	88	88
No	88	88	88	No	88	88	88	88	88	88	88	88	4	88	88
No	88	88	88	No	88	88	88	88	88	88	88	88	Ye	88	88
No	88	88	88	No	88	88	88	88	88	88	88	88	4	88	88

Ye	88	88	88	No	88	88	88	88	88	88	88	88	Ye	88	88
No	88	88	88	No	88	88	88	88	88	88	88	88	4	88	88
No	88	88	88	No	88	88	88	88	88	88	88	88	Ye	88	88
No	88	88	88	No	88	88	88	88	88	88	88	88	Ye	88	88
No	88	88	88	No	88	88	88	88	88	88	88	88	Ye	88	88
No	88	88	88	No	88	88	88	88	88	88	88	88	Ye	88	88
No	88	88	88	No	88	88	88	88	88	88	88	88	Ye	88	88
Ye	88	88	88	No	88	88	88	88	88	88	88	88	Ye	88	88
No	88	88	88	No	88	88	88	88	88	88	88	88	Ye	88	88
No	88	88	88	No	88	88	88	88	88	88	88	88	Ye	88	88
No	88	No	88	No	88	88	88	88	88	88	88	88	Ye	88	Ye	
No	88	88	88	No	88	88	88	88	88	88	88	88	Ye	88	88
No	88	88	88	Ye	88	88	88	No	88	88	88	88	Ye	88	88
No	88	88	88	No	88	88	88	88	88	88	88	88	Ye	88	88
No	88	88	88	No	88	88	88	88	88	88	88	88	Ye	88	88
No	88	88	88	No	88	88	88	88	88	88	88	88	4	88	88
No	88	88	88	No	88	88	88	88	88	88	88	88	Ye	88	88
No	88	88	88	No	88	88	88	88	88	88	88	88	Ye	88	88

q45w3	q46d	q46d1	q46d2	q46d3	q47i	q47i1	q47i2	q47i3	q47anti	q47anti1	q47anti2	q47anti3
88	Y	8	8	8	88	88	88	88	NA	NA	NA	NA
88	Y	8	8	8	88	88	88	88	NA	NA	NA	NA
88	Y	8	8	8	88	88	88	88	NA	NA	NA	NA
88	Y	8	8	8	88	88	88	88	NA	NA	NA	NA
88	Y	8	8	8	88	88	88	88	NA	NA	NA	NA
88	Y	8	8	8	88	88	88	88	NA	NA	NA	NA
88	Y	8	8	8	88	88	88	88	NA	NA	NA	NA
88	Y	8	8	8	88	88	88	88	NA	NA	NA	NA
88	Y	8	8	8	88	88	88	88	NA	NA	NA	NA
88	Y	8	8	8	88	88	88	88	NA	NA	NA	NA
88	N	8	8	8	ot	88	88	88	NA	NA	NA	NA
88	Y	8	8	8	88	88	88	88	NA	NA	NA	NA
88	Y	8	8	8	88	88	88	88	NA	NA	NA	NA
88	Y	8	8	8	88	88	88	88	NA	NA	NA	NA
88	Y	8	8	8	88	88	88	88	NA	NA	NA	NA
88	Y	8	8	8	88	88	88	88	NA	NA	NA	NA
88	Y	8	8	8	88	88	88	88	NA	NA	NA	NA
88	Y	8	8	8	88	88	88	88	NA	NA	NA	NA
88	Y	8	8	8	88	88	88	88	NA	NA	NA	NA
88	Y	8	8	8	88	88	88	88	NA	NA	NA	NA
88	Y	8	8	8	88	88	88	88	NA	NA	NA	NA
88	N	8	N	8	ot	88	ot	88	NA	NA	NA	NA
88	Y	8	8	8	88	88	88	88	NA	NA	NA	NA
88	Y	8	8	8	88	88	88	88	NA	NA	NA	NA
88	Y	8	8	8	88	88	88	88	NA	NA	NA	NA
88	Y	8	8	8	88	88	88	88	NA	NA	NA	NA
88	Y	8	8	8	88	88	88	88	NA	NA	NA	NA
88	N	8	8	8	No	88	88	88	NA	NA	NA	NA
88	Y	8	8	8	88	88	88	88	NA	NA	NA	NA
88	Y	8	8	8	88	88	88	88	NA	NA	NA	NA
88	Y	8	8	8	88	88	88	88	NA	NA	NA	NA
88	Y	8	8	8	88	88	88	88	NA	NA	NA	NA
88	Y	8	8	8	88	88	88	88	NA	NA	NA	NA
88	Y	8	8	8	88	88	88	88	NA	NA	NA	NA
88	Y	8	8	8	88	88	88	88	NA	NA	NA	NA
88	Y	8	8	8	88	88	88	88	NA	NA	NA	NA
88	N	8	8	8	88	88	88	88	NA	NA	NA	NA
88	N	8	N	8	Fo	88	No	88	NA	NA	NA	NA
88	Y	8	8	8	88	88	88	88	NA	NA	NA	NA
88	Y	8	8	8	88	88	88	88	NA	NA	NA	NA
88	Y	8	8	8	88	88	88	88	NA	NA	NA	NA
88	Y	8	8	8	88	88	88	88	NA	NA	NA	NA
88	Y	8	8	8	88	88	88	88	NA	NA	NA	NA
88	Y	8	8	8	88	88	88	88	NA	NA	NA	NA
88	Y	8	8	8	88	88	88	88	NA	NA	NA	NA
88	Y	8	8	8	88	88	88	88	NA	NA	NA	NA

88	Y	8	8	8	88	88	88	88	NA	NA	NA	NA
88	Y	8	8	8	88	88	88	88	NA	NA	NA	NA
88	Y	8	8	8	88	88	88	88	NA	NA	NA	NA
88	Y	8	8	8	88	88	88	88	NA	NA	NA	NA
88	Y	8	8	8	88	88	88	88	NA	NA	NA	NA
88	Y	8	8	8	88	88	88	88	NA	NA	NA	NA
88	Y	8	8	8	88	88	88	88	NA	NA	NA	NA
88	Y	8	8	8	88	88	88	88	NA	NA	NA	NA
88	Y	8	8	8	88	88	88	88	NA	NA	NA	NA
88	Y	8	8	8	88	88	88	88	NA	NA	NA	NA
88	Y	8	8	8	88	88	88	88	NA	NA	NA	NA
88	Y	8	8	8	88	88	88	88	NA	NA	NA	NA
88	Y	8	8	8	88	88	88	88	NA	NA	NA	NA
88	Y	8	8	8	88	88	88	88	NA	NA	NA	NA
88	Y	8	8	8	88	88	88	88	NA	NA	NA	NA
88	Y	8	8	8	88	88	88	88	NA	NA	NA	NA
88	N	8	8	8	Me	88	88	88	NA	NA	NA	NA
88	Y	8	8	8	88	88	88	88	NA	NA	NA	NA
88	Y	8	8	8	88	88	88	88	NA	NA	NA	NA
88	Y	8	8	8	88	88	88	88	NA	NA	NA	NA
88	Y	8	8	8	88	88	88	88	NA	NA	NA	NA
88	Y	8	8	8	88	88	88	88	NA	NA	NA	NA
88	Y	8	8	8	88	88	88	88	NA	NA	NA	NA
88	Y	8	8	8	88	88	88	88	NA	NA	NA	NA
88	Y	8	8	8	88	88	88	88	NA	NA	NA	NA
88	Y	8	8	8	88	88	88	88	NA	NA	NA	NA
88	Y	8	8	8	88	88	88	88	NA	NA	NA	NA
88	Y	8	8	8	88	88	88	88	NA	NA	NA	NA
88	N	8	8	8	Me	88	88	88	NA	NA	NA	NA
88	Y	8	8	8	88	88	88	88	NA	NA	NA	NA
88	Y	8	8	8	88	88	88	88	NA	NA	NA	NA
88	Y	8	8	8	88	88	88	88	NA	NA	NA	NA
88	Y	8	8	8	88	88	88	88	NA	NA	NA	NA
88	Y	8	8	8	88	88	88	88	NA	NA	NA	NA
88	Y	8	8	8	88	88	88	88	NA	NA	NA	NA
88	Y	8	8	8	88	88	88	88	NA	NA	NA	NA
88	Y	8	8	8	88	88	88	88	NA	NA	NA	NA
88	Y	8	8	8	88	88	88	88	NA	NA	NA	NA
88	Y	8	8	8	88	88	88	88	NA	NA	NA	NA
88	Y	8	8	8	88	88	88	88	NA	NA	NA	NA
88	Y	8	8	8	88	88	88	88	NA	NA	NA	NA
88	Y	8	8	8	88	88	88	88	NA	NA	NA	NA
88	N	8	N	8	Me	88	88	88	NA	NA	NA	NA
88	N	8	N	8	Me	88	88	88	NA	NA	NA	NA
88	N	8	N	8	Me	88	88	88	NA	NA	NA	NA
88	Y	8	8	8	88	88	88	88	NA	NA	NA	NA
88	Y	8	8	8	88	88	88	88	NA	NA	NA	NA

88	8	8	8	8	88	88	88	88	NA	NA	NA	NA
88	N	8	8	8	Ca	88	88	88	NA	NA	NA	NA
88	Y	8	8	8	88	88	88	88	NA	NA	NA	NA
88	N	8	8	8	Si	88	88	88	NA	NA	NA	NA
88	Y	8	8	8	88	88	88	88	NA	NA	NA	NA
88	Y	8	8	8	88	88	88	88	NA	NA	NA	NA
88	Y	8	8	8	88	88	88	88	NA	NA	NA	NA
88	Y	8	8	8	88	88	88	88	NA	NA	NA	NA
88	Y	8	8	8	88	88	88	88	NA	NA	NA	NA
88	Y	8	8	8	88	88	88	88	NA	NA	NA	NA
88	Y	8	8	8	88	88	88	88	NA	NA	NA	NA
88	Y	8	8	8	88	88	88	88	NA	NA	NA	NA
88	Y	8	8	8	88	88	88	88	NA	NA	NA	NA
88	Y	8	8	8	88	88	88	88	NA	NA	NA	NA
88	Y	8	8	8	88	88	88	88	NA	NA	NA	NA
88	Y	8	8	8	88	88	88	88	NA	NA	NA	NA
88	Y	8	8	8	88	88	88	88	NA	NA	NA	NA
88	N	8	8	8	No	88	88	88	NA	NA	NA	NA
88	Y	8	8	8	88	88	88	88	NA	NA	NA	NA
88	Y	8	8	8	88	88	88	88	NA	NA	NA	NA
88	Y	8	8	8	88	88	88	88	NA	NA	NA	NA
88	Y	8	8	8	88	88	88	88	NA	NA	NA	NA
88	Y	8	8	8	88	88	88	88	NA	NA	NA	NA
88	N	8	8	8	Do	88	88	88	NA	NA	NA	NA
88	Y	8	8	8	88	88	88	88	NA	NA	NA	NA
88	Y	8	8	8	88	88	88	88	NA	NA	NA	NA
88	Y	8	8	8	88	88	88	88	NA	NA	NA	NA
88	Y	8	8	8	88	88	88	88	NA	NA	NA	NA
88	Y	8	8	8	88	88	88	88	NA	NA	NA	NA
88	Y	8	8	8	88	88	88	88	NA	NA	NA	NA
88	Y	8	8	8	88	88	88	88	NA	NA	NA	NA
88	Y	8	8	8	88	88	88	88	NA	NA	NA	NA
88	N	8	8	8	Ca	88	88	88	NA	NA	NA	NA
88	Y	8	8	8	88	88	88	88	NA	NA	NA	NA
88	Y	8	8	8	88	88	88	88	NA	NA	NA	NA
88	Y	8	8	8	88	88	88	88	NA	NA	NA	NA
88	Y	8	8	8	88	88	88	88	NA	NA	NA	NA
88	Y	8	8	8	88	88	88	88	NA	NA	NA	NA
88	Y	8	8	8	88	88	88	88	NA	NA	NA	NA
88	Y	8	8	8	88	88	88	88	NA	NA	NA	NA
88	Y	8	8	8	88	88	88	88	NA	NA	NA	NA
88	Y	8	8	8	88	88	88	88	NA	NA	NA	NA
88	Y	8	8	8	88	88	88	88	NA	NA	NA	NA
88	N	8	N	8	Me	88	88	88	NA	NA	NA	NA
88	Y	8	8	8	88	88	88	88	NA	NA	NA	NA
88	Y	8	8	8	88	88	88	88	NA	NA	NA	NA
88	N	8	N	8	Fo	88	88	88	NA	NA	NA	NA

88	Y	8	8	8	88	88	88	88	NA	NA	NA	NA
88	Y	8	8	8	88	88	88	88	NA	NA	NA	NA
88	Y	8	8	8	88	88	88	88	NA	NA	NA	NA
88	Y	8	8	8	88	88	88	88	NA	NA	NA	NA
88	Y	8	8	8	88	88	88	88	NA	NA	NA	NA
88	Y	8	8	8	88	88	88	88	NA	NA	NA	NA
88	Y	8	8	8	88	88	88	88	NA	NA	NA	NA
88	Y	8	8	8	88	88	88	88	NA	NA	NA	NA
88	Y	8	8	8	88	88	88	88	NA	NA	NA	NA
88	Y	8	8	8	88	88	88	88	NA	NA	NA	NA
88	N	8	N	8	No	88	88	88	NA	NA	NA	NA
88	Y	8	8	8	88	88	88	88	NA	NA	NA	NA
88	Y	8	8	8	88	88	88	88	NA	NA	NA	NA
88	Y	8	8	8	88	88	88	88	NA	NA	NA	NA
88	Y	8	8	8	88	88	88	88	NA	NA	NA	NA
88	N	8	8	8	Fo	88	88	88	NA	NA	NA	NA
88	Y	8	8	8	88	88	88	88	NA	NA	NA	NA
88	Y	8	8	8	88	88	88	88	NA	NA	NA	NA
88	Y	8	8	8	88	88	88	88	NA	NA	NA	NA
88	N	8	8	8	88	88	88	88	NA	NA	NA	NA
88	Y	8	8	8	88	88	88	88	NA	NA	NA	NA
88	Y	8	8	8	88	88	88	88	NA	NA	NA	NA
88	Y	8	8	8	88	88	88	88	NA	NA	NA	NA
88	Y	8	8	8	88	88	88	88	NA	NA	NA	NA
88	Y	8	8	8	88	88	88	88	NA	NA	NA	NA
88	Y	8	8	8	88	88	88	88	NA	NA	NA	NA
88	Y	8	8	8	88	88	88	88	NA	NA	NA	NA
88	Y	8	8	8	88	88	88	88	NA	NA	NA	NA
88	N	8	8	8	No	88	88	88	NA	NA	NA	NA
88	Y	8	8	8	88	88	88	88	NA	NA	NA	NA
88	Y	8	8	8	88	88	88	88	NA	NA	NA	NA
88	Y	8	8	8	88	88	88	88	NA	NA	NA	NA
88	Y	8	8	8	88	88	88	88	NA	NA	NA	NA
88	Y	8	8	8	88	88	88	88	NA	NA	NA	NA
88	Y	8	8	8	88	88	88	88	NA	NA	NA	NA
88	Y	8	8	8	88	88	88	88	NA	NA	NA	NA
88	Y	8	8	8	88	88	88	88	NA	NA	NA	NA
88	Y	8	8	8	88	88	88	88	NA	NA	NA	NA
88	Y	8	8	8	88	88	88	88	NA	NA	NA	NA
88	Y	8	8	8	88	88	88	88	NA	NA	NA	NA
88	Y	8	8	8	88	88	88	88	NA	NA	NA	NA
88	Y	8	8	8	88	88	88	88	NA	NA	NA	NA
88	Y	8	8	8	88	88	88	88	NA	NA	NA	NA
88	N	8	8	8	ot	88	88	88	NA	NA	NA	NA
88	Y	8	8	8	88	88	88	88	NA	NA	NA	NA
88	Y	8	8	8	88	88	88	88	NA	NA	NA	NA
88	Y	8	8	8	88	88	88	88	NA	NA	NA	NA

88	Y	8	8	8	88	88	88	88	NA	NA	NA	NA
88	N	8	N	8	Si	88	88	88	NA	NA	NA	NA
88	Y	8	8	8	88	88	88	88	NA	NA	NA	NA
88	N	8	8	8	88	88	88	88	NA	NA	NA	NA
88	Y	8	8	8	88	88	88	88	NA	NA	NA	NA
88	Y	8	8	8	88	88	88	88	NA	NA	NA	NA
88	Y	8	8	8	88	88	88	88	NA	NA	NA	NA
88	Y	8	8	8	88	88	88	88	NA	NA	NA	NA
88	Y	8	8	8	88	88	88	88	NA	NA	NA	NA
88	Y	8	8	8	88	88	88	88	NA	NA	NA	NA
88	Y	8	8	8	88	88	88	88	NA	NA	NA	NA
88	Y	8	8	8	88	88	88	88	NA	NA	NA	NA
88	Y	8	8	8	88	88	88	88	NA	NA	NA	NA
88	Y	8	8	8	88	88	88	88	NA	NA	NA	NA
88	Y	8	8	8	88	88	88	88	NA	NA	NA	NA
88	Y	8	8	8	88	88	88	88	NA	NA	NA	NA
88	Y	8	8	8	88	88	88	88	NA	NA	NA	NA
88	Y	8	8	8	88	88	88	88	NA	NA	NA	NA
88	Y	8	8	8	88	88	88	88	NA	NA	NA	NA
88	Y	8	8	8	88	88	88	88	NA	NA	NA	NA
88	Y	8	8	8	88	88	88	88	NA	NA	NA	NA
88	Y	8	8	8	88	88	88	88	NA	NA	NA	NA
88	Y	8	8	8	88	88	88	88	NA	NA	NA	NA
88	Y	8	8	8	88	88	88	88	NA	NA	NA	NA
88	Y	8	8	8	88	88	88	88	NA	NA	NA	NA
88	N	8	N	8	No	88	88	88	NA	NA	NA	NA
88	Y	8	8	8	88	88	88	88	NA	NA	NA	NA
88	Y	8	8	8	88	88	88	88	NA	NA	NA	NA
88	Y	8	8	8	88	88	88	88	NA	NA	NA	NA
88	Y	8	8	8	88	88	88	88	NA	NA	NA	NA
88	Y	8	8	8	88	88	88	88	NA	NA	NA	NA
88	Y	8	8	8	88	88	88	88	NA	NA	NA	NA
88	Y	8	8	8	88	88	88	88	NA	NA	NA	NA
88	Y	8	8	8	88	88	88	88	NA	NA	NA	NA
88	Y	8	8	8	88	88	88	88	NA	NA	NA	NA
88	N	8	8	8	ot	88	88	88	NA	NA	NA	NA
88	Y	8	8	8	88	88	88	88	NA	NA	NA	NA
88	Y	8	8	8	88	88	88	88	NA	NA	NA	NA
88	Y	8	8	8	88	88	88	88	NA	NA	NA	NA
88	Y	8	8	8	88	88	88	88	NA	NA	NA	NA
88	Y	8	8	8	88	88	88	88	NA	NA	NA	NA
88	Y	8	8	8	88	88	88	88	NA	NA	NA	NA
88	Y	8	8	8	88	88	88	88	NA	NA	NA	NA
88	Y	8	8	8	88	88	88	88	NA	NA	NA	NA
88	Y	8	8	8	88	88	88	88	NA	NA	NA	NA
88	Y	8	8	8	88	88	88	88	NA	NA	NA	NA
88	Y	8	8	8	88	88	88	88	NA	NA	NA	NA

88	Y	8	8	8	88	88	88	88	NA	NA	NA	NA
88	Y	8	8	8	88	88	88	88	NA	NA	NA	NA
88	Y	8	8	8	88	88	88	88	NA	NA	NA	NA
88	Y	8	8	8	88	88	88	88	NA	NA	NA	NA
88	Y	8	8	8	88	88	88	88	NA	NA	NA	NA
88	Y	8	8	8	88	88	88	88	NA	NA	NA	NA
88	Y	8	8	8	88	88	88	88	NA	NA	NA	NA
88	Y	8	8	8	88	88	88	88	NA	NA	NA	NA
88	Y	8	8	8	88	88	88	88	NA	NA	NA	NA
88	N	8	8	8	Do	88	88	88	NA	NA	NA	NA
88	Y	8	8	8	88	88	88	88	NA	NA	NA	NA
88	Y	8	8	8	88	88	88	88	NA	NA	NA	NA
88	Y	8	8	8	88	88	88	88	NA	NA	NA	NA
88	Y	8	8	8	88	88	88	88	NA	NA	NA	NA
88	Y	8	8	8	88	88	88	88	NA	NA	NA	NA
88	Y	8	8	8	88	88	88	88	NA	NA	NA	NA
88	Y	8	8	8	88	88	88	88	NA	NA	NA	NA
88	N	8	N	8	Ca	88	88	88	NA	NA	NA	NA
88	Y	8	8	8	88	88	88	88	NA	NA	NA	NA
88	Y	8	8	8	88	88	88	88	NA	NA	NA	NA
88	Y	8	8	8	88	88	88	88	NA	NA	NA	NA
88	Y	8	8	8	88	88	88	88	NA	NA	NA	NA
88	Y	8	8	8	88	88	88	88	NA	NA	NA	NA
88	Y	8	8	8	88	88	88	88	NA	NA	NA	NA
88	Y	8	8	8	88	88	88	88	NA	NA	NA	NA
88	N	8	N	8	Si	88	88	88	NA	NA	NA	NA
88	Y	8	8	8	88	88	88	88	NA	NA	NA	NA
88	Y	8	8	8	88	88	88	88	NA	NA	NA	NA
88	Y	8	8	8	88	88	88	88	NA	NA	NA	NA
88	N	8	N	8	Me	88	88	88	NA	NA	NA	NA
88	Y	8	8	8	88	88	88	88	NA	NA	NA	NA
88	Y	8	8	8	88	88	88	88	NA	NA	NA	NA
88	Y	8	8	8	88	88	88	88	NA	NA	NA	NA
88	Y	8	8	8	88	88	88	88	NA	NA	NA	NA
88	Y	8	8	8	88	88	88	88	NA	NA	NA	NA
88	Y	8	8	8	88	88	88	88	NA	NA	NA	NA
88	Y	8	8	8	88	88	88	88	NA	NA	NA	NA
88	Y	8	8	8	88	88	88	88	NA	NA	NA	NA
88	Y	8	8	8	88	88	88	88	NA	NA	NA	NA
88	Y	8	8	8	88	88	88	88	NA	NA	NA	NA
88	Y	8	8	8	88	88	88	88	NA	NA	NA	NA
88	Y	8	8	8	88	88	88	88	NA	NA	NA	NA
88	N	8	8	8	Fo	88	88	88	NA	NA	NA	NA
88	Y	8	8	8	88	88	88	88	NA	NA	NA	NA
88	Y	8	8	8	88	88	88	88	NA	NA	NA	NA
88	Y	8	8	8	88	88	88	88	NA	NA	NA	NA
88	Y	8	8	8	88	88	88	88	NA	NA	NA	NA

88	Y	8		8	8		88	88	88	88	NA	NA	NA	NA
88	Y	8		8	8		88	88	88	88	NA	NA	NA	NA
88	Y	8		8	8		88	88	88	88	NA	NA	NA	NA
88	Y	8		8	8		88	88	88	88	NA	NA	NA	NA
88	Y	8		8	8		88	88	88	88	NA	NA	NA	NA
88	N	8	N		8	Do		88	88	88	NA	NA	NA	NA
88	Y	8		8	8		88	88	88	88	NA	NA	NA	NA
88	Y	8		8	8		88	88	88	88	NA	NA	NA	NA
88	Y	8		8	8		88	88	88	88	NA	NA	NA	NA
88	Y	8		8	8		88	88	88	88	NA	NA	NA	NA
88	Y	8		8	8		88	88	88	88	NA	NA	NA	NA
88	Y	8		8	8		88	88	88	88	NA	NA	NA	NA
88	N	8	N		8	Do		88	88	88	NA	NA	NA	NA
88	Y	8		8	8		88	88	88	88	NA	NA	NA	NA
88	Y	8		8	8		88	88	88	88	NA	NA	NA	NA
88	Y	8		8	8		88	88	88	88	NA	NA	NA	NA
88	Y	8		8	8		88	88	88	88	NA	NA	NA	NA
88	Y	8		8	8		88	88	88	88	NA	NA	NA	NA

q47other	q47other1	q47other2	q47other3	q48h	q48h1	q48h2	q48h3	q49t	q49t1	q49t2
NA	NA	NA	NA	2	88	88	88	Ev	88	88
NA	NA	NA	NA	2	88	88	88	Af	88	88
NA	NA	NA	NA	3	88	88	88	Ev	88	88
NA	NA	NA	NA	3	88	88	88	Mo	88	88
NA	NA	NA	NA	2	88	88	88	Mo	88	88
NA	NA	NA	NA	2	88	88	88	Ev	88	88
NA	NA	NA	NA	1	88	1	88	Mo	88	Ev
NA	NA	NA	NA	1	88	1	88	Mo	88	Ev
NA	NA	NA	NA	2	88	88	88	Mo	88	88
NA	NA	NA	NA	2	88	88	88	Ev	88	88
DID NOT EAT	NA	NA	NA	88	88	88	88	88	88	88
NA	NA	NA	NA	2	88	88	88	Ev	88	88
NA	NA	NA	NA	1	88	1	88	Mo	88	Ev
NA	NA	NA	NA	1	88	1	88	Mo	88	Ev
NA	NA	NA	NA	2	88	88	88	Af	88	88
NA	NA	NA	NA	3	88	88	88	Mo	88	88
NA	NA	NA	NA	3	88	88	88	Ev	88	88
NA	NA	NA	NA	3	88	88	88	Ev	88	88
NA	NA	NA	NA	1	88	1	88	Mo	88	Ev
NA	NA	NA	NA	2	88	88	88	Ev	88	88
NA	NA	NA	NA	2	88	88	88	Ev	88	88
BECAUSE THE CHILD BO	NA	TEMPETUF	NA	88	88	88	88	88	88	88
NA	NA	NA	NA	2	88	88	88	Ev	88	88
NA	NA	NA	NA	3	88	88	88	Ev	88	88
NA	NA	NA	NA	3	88	88	88	Ev	88	88
NA	NA	NA	NA	2	88	88	88	Ev	88	88
NA	NA	NA	NA	1	88	88	88	Af	88	88
NA	NA	NA	NA	88	88	88	88	88	88	88
NA	NA	NA	NA	1	88	88	88	Mo	88	88
NA	NA	NA	NA	1	88	88	88	Af	88	88
NA	NA	NA	NA	2	88	88	88	Ev	88	88
NA	NA	NA	NA	2	88	88	88	Af	88	88
NA	NA	NA	NA	1	88	88	88	Ev	88	88
NA	NA	NA	NA	1	88	1	88	Mo	88	Ev
NA	NA	NA	NA	1	88	1	88	Mo	88	Ev
NA	NA	NA	NA	1	88	1	88	Mo	88	Ev
NA	NA	NA	NA	88	88	88	88	88	88	88
NA	NA	NA	NA	88	88	88	88	88	88	88
NA	NA	NA	NA	1	88	1	88	Mo	88	Ev
NA	NA	NA	NA	1	88	1	88	Mo	88	Ev
NA	NA	NA	NA	1	88	2	88	Mo	88	Ev
NA	NA	NA	NA	1	88	1	88	Mo	88	Ev
NA	NA	NA	NA	1	88	1	88	Mo	88	Ev
NA	NA	NA	NA	2	88	88	88	Ev	88	88
NA	NA	NA	NA	1	88	88	88	Mo	88	88
NA	NA	NA	NA	1	88	1	88	Mo	88	Ev

NA	NA	NA	NA	2	88	88	88	Mo	88	88
NA	NA	NA	NA	1	88	88	88	Mo	88	88
NA	NA	NA	NA	3	88	88	88	Mo	88	88
NA	NA	NA	NA	1	88	1	88	Mo	88	Ev
NA	NA	NA	NA	2	88	88	88	Ev	88	88
NA	NA	NA	NA	3	88	88	88	Mo	88	88
NA	NA	NA	NA	2	88	88	88	Ev	88	88
NA	NA	NA	NA	2	88	88	88	Ev	88	88
NA	NA	NA	NA	3	88	88	88	Ev	88	88
NA	NA	NA	NA	3	88	88	88	Ev	88	88
NA	NA	NA	NA	2	88	88	88	Ev	88	88
NA	NA	NA	NA	2	88	88	88	Mo	88	88
NA	NA	NA	NA	2	88	88	88	Ev	88	88
NA	NA	NA	NA	2	88	88	88	Mo	88	88
NA	NA	NA	NA	3	88	88	88	Ev	88	88
NA	NA	NA	NA	2	88	88	88	Mo	88	88
NA	NA	NA	NA	88	88	88	88	88	88	88
NA	NA	NA	NA	2	88	88	88	Ev	88	88
NA	NA	NA	NA	2	88	88	88	Mo	88	88
NA	NA	NA	NA	1	88	1	88	Mo	88	Ev
NA	NA	NA	NA	1	88	88	88	Ev	88	88
NA	NA	NA	NA	2	88	88	88	Mo	88	88
NA	NA	NA	NA	2	88	88	88	Ev	88	88
NA	NA	NA	NA	3	88	88	88	Ev	88	88
NA	NA	NA	NA	2	88	88	88	Ev	88	88
NA	NA	NA	NA	3	88	88	88	Ev	88	88
NA	NA	NA	NA	2	88	88	88	Mo	88	88
NA	NA	NA	NA	3	88	88	88	Ev	88	88
NA	NA	NA	NA	88	88	88	88	88	88	88
NA	NA	NA	NA	2	88	88	88	Mo	88	88
NA	NA	NA	NA	2	88	88	88	Ev	88	88
NA	NA	NA	NA	2	88	88	88	Mo	88	88
NA	NA	NA	NA	3	88	88	88	Ev	88	88
NA	NA	NA	NA	3	88	88	88	Af	88	88
NA	NA	NA	NA	3	88	88	88	Af	88	88
NA	NA	NA	NA	2	88	88	88	Af	88	88
NA	NA	NA	NA	2	88	88	88	Mo	88	88
NA	NA	NA	NA	2	88	88	88	Af	88	88
NA	NA	NA	NA	2	88	88	88	Ev	88	88
NA	NA	NA	NA	2	88	88	88	Mo	88	88
NA	NA	NA	NA	1	88	1	88	Mo	88	Ev
NA	NA	NA	NA	3	88	88	88	Mo	88	88
NA	NA	NA	NA	88	88	88	88	88	88	88
NA	NA	NA	NA	88	88	88	88	88	88	88
NA	NA	NA	NA	88	88	88	88	88	88	88
NA	NA	NA	NA	2	88	88	88	Mo	88	88
NA	NA	NA	NA	3	88	88	88	Mo	88	88

NA	NA	NA	NA	3	88	88	88	Mo	88	88
NA	NA	NA	NA	88	88	88	88	88	88	88
NA	NA	NA	NA	3	88	88	88	Ev	88	88
NA	NA	NA	NA	88	88	88	88	88	88	88
NA	NA	NA	NA	2	88	88	88	Af	88	88
NA	NA	NA	NA	2	88	88	88	Ev	88	88
NA	NA	NA	NA	2	88	88	88	Ev	88	88
NA	NA	NA	NA	2	88	88	88	Ev	88	88
NA	NA	NA	NA	2	88	88	88	Af	88	88
NA	NA	NA	NA	1	88	88	88	Ev	88	88
NA	NA	NA	NA	2	88	88	88	Ev	88	88
NA	NA	NA	NA	2	88	88	88	Ev	88	88
NA	NA	NA	NA	2	88	88	88	Mo	88	88
NA	NA	NA	NA	2	88	88	88	Mo	88	88
NA	NA	NA	NA	3	88	88	88	Mo	88	88
NA	NA	NA	NA	2	88	88	88	Mo	88	88
NA	NA	NA	NA	3	88	88	88	Ev	88	88
NA	NA	NA	NA	88	88	88	88	88	88	88
NA	NA	NA	NA	2	88	88	88	Af	88	88
NA	NA	NA	NA	2	88	88	88	Mo	88	88
NA	NA	NA	NA	2	88	88	88	Ev	88	88
NA	NA	NA	NA	2	88	88	88	Ev	88	88
NA	NA	NA	NA	2	88	88	88	Ev	88	88
NA	NA	NA	NA	88	88	88	88	88	88	88
NA	NA	NA	NA	2	88	88	88	Af	88	88
NA	NA	NA	NA	2	88	88	88	Ev	88	88
NA	NA	NA	NA	2	88	88	88	Mo	88	88
NA	NA	NA	NA	3	88	88	88	Mo	88	88
NA	NA	NA	NA	3	88	88	88	Mo	88	88
NA	NA	NA	NA	1	88	88	88	Af	88	88
NA	NA	NA	NA	1	88	1	88	Mo	88	Ev
NA	NA	NA	NA	2	88	88	88	Mo	88	88
NA	NA	NA	NA	88	88	88	88	88	88	88
NA	NA	NA	NA	1	88	1	88	Mo	88	88
NA	NA	NA	NA	1	88	88	88	Mo	88	88
NA	NA	NA	NA	2	88	88	88	Mo	88	88
NA	NA	NA	NA	2	88	88	88	Mo	88	88
NA	NA	NA	NA	2	88	88	88	Mo	88	88
NA	NA	NA	NA	3	88	88	88	Mo	88	88
NA	NA	NA	NA	3	88	88	88	Mo	88	88
NA	NA	NA	NA	2	88	88	88	Mo	88	88
NA	NA	NA	NA	3	88	88	88	Ev	88	88
NA	NA	NA	NA	3	88	88	88	Mo	88	88
NA	NA	NA	NA	88	88	88	88	88	88	88
NA	NA	NA	NA	2	88	88	88	Mo	88	88
NA	NA	NA	NA	3	88	88	88	Mo	88	88
NA	NA	NA	NA	88	88	88	88	88	88	88

NA	NA	NA	NA	2	88	88	88	Af	88	88
NA	NA	NA	NA	1	88	1	88	Mo	88	Ev
NA	NA	NA	NA	1	88	1	88	Mo	88	Ev
NA	NA	NA	NA	3	88	88	88	Mo	88	88
NA	NA	NA	NA	1	88	88	88	Mo	88	88
NA	NA	NA	NA	3	88	88	88	Mo	88	88
NA	NA	NA	NA	2	88	88	88	Ev	88	88
NA	NA	NA	NA	2	88	88	88	Mo	88	88
NA	NA	NA	NA	2	88	88	88	Ev	88	88
NA	NA	NA	NA	1	88	88	88	Mo	88	88
NA	NA	NA	NA	88	88	88	88	88	88	88
NA	NA	NA	NA	2	88	88	88	Ev	88	88
NA	NA	NA	NA	2	88	88	88	Mo	88	88
NA	NA	NA	NA	2	88	88	88	Mo	88	88
NA	NA	NA	NA	2	88	88	88	Ev	88	88
NA	NA	NA	NA	88	88	88	88	88	88	88
NA	NA	NA	NA	2	88	88	88	Mo	88	88
NA	NA	NA	NA	2	88	88	88	Mo	88	88
NA	NA	NA	NA	2	88	88	88	Ev	88	88
NA	NA	NA	NA	2	88	88	88	Ev	88	88
NA	NA	NA	NA	2	88	88	88	Ev	88	88
NA	NA	NA	NA	2	88	88	88	Mo	88	88
NA	NA	NA	NA	2	88	88	88	Mo	88	88
NA	NA	NA	NA	2	88	88	88	Ev	88	88
NA	NA	NA	NA	2	88	88	88	Mo	88	88
NA	NA	NA	NA	2	88	88	88	Mo	88	88
NA	NA	NA	NA	1	88	88	88	Mo	88	88
NA	NA	NA	NA	1	88	88	88	Mo	88	88
NA	NA	NA	NA	88	88	88	88	88	88	88
NA	NA	NA	NA	1	88	1	88	Mo	88	Ev
NA	NA	NA	NA	1	88	88	88	Af	88	88
NA	NA	NA	NA	2	88	88	88	Ev	88	88
NA	NA	NA	NA	2	88	88	88	Mo	88	88
NA	NA	NA	NA	2	88	88	88	Mo	88	88
NA	NA	NA	NA	2	88	88	88	Mo	88	88
NA	NA	NA	NA	3	88	88	88	Mo	88	88
NA	NA	NA	NA	2	88	88	88	Ev	88	88
NA	NA	NA	NA	1	88	88	88	Af	88	88
NA	NA	NA	NA	2	88	88	88	Ev	88	88
NA	NA	NA	NA	1	88	88	88	Mo	88	88
NA	NA	NA	NA	2	88	88	88	Mo	88	88
NA	NA	NA	NA	1	88	88	88	Af	88	88
NA	NA	NA	NA	2	88	88	88	Af	88	88
CHILD REFEWS TO TAKE	NA	NA	NA	88	88	88	88	88	88	88
NA	NA	NA	NA	2	88	88	88	Mo	88	88
NA	NA	NA	NA	2	88	88	88	Mo	88	88
NA	NA	NA	NA	2	88	88	88	Ev	88	88

NA	NA	NA	NA	2	88	88	88	Ev	88	88
NA	NA	NA	NA	88	88	88	88	88	88	88
NA	NA	NA	NA	3	88	88	88	Ev	88	88
NA	NA	NA	NA	2	88	88	88	Ev	88	88
NA	NA	NA	NA	2	88	88	88	Ev	88	88
NA	NA	NA	NA	2	88	88	88	Mo	88	88
NA	NA	NA	NA	1	88	88	88	Mo	88	88
NA	NA	NA	NA	2	88	88	88	Mo	88	88
NA	NA	NA	NA	2	88	88	88	Af	88	88
NA	NA	NA	NA	2	88	88	88	Mo	88	88
NA	NA	NA	NA	2	88	88	88	Mo	88	88
NA	NA	NA	NA	2	88	88	88	Mo	88	88
NA	NA	NA	NA	1	88	88	88	Mo	88	88
NA	NA	NA	NA	2	88	88	88	Af	88	88
NA	NA	NA	NA	3	88	88	88	Mo	88	88
NA	NA	NA	NA	2	88	88	88	Mo	88	88
NA	NA	NA	NA	2	88	88	88	Mo	88	88
NA	NA	NA	NA	2	88	88	88	Mo	88	88
NA	NA	NA	NA	1	88	88	88	Mo	88	88
NA	NA	NA	NA	3	88	88	88	Mo	88	88
NA	NA	NA	NA	2	88	88	88	Ev	88	88
NA	NA	NA	NA	2	88	88	88	Ev	88	88
NA	NA	NA	NA	1	88	88	88	Mo	88	88
NA	NA	NA	NA	2	88	88	88	Mo	88	88
NA	NA	NA	NA	3	88	88	88	Mo	88	88
NA	NA	NA	NA	88	88	88	88	88	88	88
NA	NA	NA	NA	2	88	88	88	Mo	88	88
NA	NA	NA	NA	2	88	88	88	Mo	88	88
NA	NA	NA	NA	3	88	88	88	Ev	88	88
NA	NA	NA	NA	3	88	88	88	Mo	88	88
NA	NA	NA	NA	3	88	88	88	Ev	88	88
NA	NA	NA	NA	2	88	88	88	Ev	88	88
NA	NA	NA	NA	3	88	88	88	Mo	88	88
NA	NA	NA	NA	3	88	88	88	Af	88	88
NA	NA	NA	NA	3	88	88	88	Af	88	88
DID NOT EAT	NA	NA	NA	88	88	88	88	88	88	88
NA	NA	NA	NA	3	88	88	88	Ev	88	88
NA	NA	NA	NA	2	88	88	88	Mo	88	88
NA	NA	NA	NA	2	88	88	88	Mo	88	88
NA	NA	NA	NA	2	88	88	88	Mo	88	88
NA	NA	NA	NA	2	88	88	88	Mo	88	88
NA	NA	NA	NA	2	88	88	88	Mo	88	88
NA	NA	NA	NA	2	88	88	88	Mo	88	88
NA	NA	NA	NA	1	88	1	88	Mo	88	Ev
NA	NA	NA	NA	3	88	88	88	Ev	88	88
NA	NA	NA	NA	3	88	88	88	Ev	88	88
NA	NA	NA	NA	2	88	88	88	Ev	88	88

NA	NA	NA	NA	2	88	88	88	Ev	88	88
NA	NA	NA	NA	1	88	88	88	Mo	88	88
NA	NA	NA	NA	1	88	88	88	Mo	88	88
NA	NA	NA	NA	2	88	88	88	Af	88	88
NA	NA	NA	NA	2	88	88	88	Af	88	88
NA	NA	NA	NA	2	88	88	88	Ev	88	88
NA	NA	NA	NA	2	88	88	88	Ev	88	88
NA	NA	NA	NA	2	88	88	88	Af	88	88
NA	NA	NA	NA	2	88	88	88	Af	88	88
NA	NA	NA	NA	88	88	88	88	88	88	88
NA	NA	NA	NA	2	88	88	88	Mo	88	88
NA	NA	NA	NA	1	88	1	88	Mo	88	Ev
NA	NA	NA	NA	2	88	88	88	Ev	88	88
NA	NA	NA	NA	2	88	88	88	Mo	88	88
NA	NA	NA	NA	2	88	88	88	Mo	88	88
NA	NA	NA	NA	2	88	88	88	Mo	88	88
NA	NA	NA	NA	2	88	88	88	Mo	88	88
NA	NA	NA	NA	88	88	88	88	88	88	88
NA	NA	NA	NA	2	88	88	88	Mo	88	88
NA	NA	NA	NA	2	88	88	88	Ev	88	88
NA	NA	NA	NA	2	88	88	88	Mo	88	88
NA	NA	NA	NA	2	88	88	88	Mo	88	88
NA	NA	NA	NA	3	88	88	88	Mo	88	88
NA	NA	NA	NA	2	88	88	88	Mo	88	88
NA	NA	NA	NA	1	88	88	88	Mo	88	88
NA	NA	NA	NA	88	88	88	88	88	88	88
NA	NA	NA	NA	2	88	88	88	Ev	88	88
NA	NA	NA	NA	1	88	88	88	Mo	88	88
NA	NA	NA	NA	2	88	88	88	Mo	88	88
NA	NA	NA	NA	88	88	88	88	88	88	88
NA	NA	NA	NA	2	88	88	88	Ev	88	88
NA	NA	NA	NA	2	88	88	88	Mo	88	88
NA	NA	NA	NA	2	88	88	88	Mo	88	88
NA	NA	NA	NA	2	88	88	88	Mo	88	88
NA	NA	NA	NA	1	88	1	88	Mo	88	Ev
NA	NA	NA	NA	1	88	1	88	Mo	88	Ev
NA	NA	NA	NA	1	88	88	88	88	88	88
NA	NA	NA	NA	2	88	88	88	Ev	88	88
NA	NA	NA	NA	2	88	88	88	Ev	88	88
NA	NA	NA	NA	1	88	88	88	Mo	88	88
NA	NA	NA	NA	1	88	1	88	Mo		Ev
NA	NA	NA	NA	1	88	1	88	Mo	88	Ev
NA	NA	NA	NA	88	88	1	88	88	88	Ev
NA	NA	NA	NA	2	88	88	88	Mo	88	88
NA	NA	NA	NA	2	88	88	88	Mo	88	88
NA	NA	NA	NA	2	88	88	88	Af	88	88
NA	NA	NA	NA	2	88	88	88	Af	88	88

NA	NA	NA	NA	1	88	88	88	Mo	88	88
NA	NA	NA	NA	2	88	88	88	Mo	88	88
NA	NA	NA	NA	2	88	88	88	Mo	88	88
NA	NA	NA	NA	2	88	88	88	Af	88	88
NA	NA	NA	NA	2	88	88	88	Mo	88	88
NA	NA	NA	NA	88	88	88	88	88	88	88
NA	NA	NA	NA	2	88	88	88	Mo	88	88
NA	NA	NA	NA	2	88	88	88	Ev	88	88
NA	NA	NA	NA	2	88	88	88	Mo	88	88
NA	NA	NA	NA	2	88	88	88	Mo	88	88
NA	NA	NA	NA	1	88	88	88	88	88	88
NA	NA	NA	NA	2	88	88	88	Mo	88	88
NA	NA	NA	NA	88	88	88	88	88	88	88
NA	NA	NA	NA	1	88	88	88	Mo	88	88
NA	NA	NA	NA	2	88	88	88	Ev	88	88
NA	NA	NA	NA	2	88	88	88	Mo	88	88
NA	NA	NA	NA	2	88	88	88	Mo	88	88
NA	NA	NA	NA	2	88	88	88	Ev	88	88

q49t3	q50t	q50t1	q50t2	q50t3	q51t	q51t1	q51t2	q51t3	q52v	q52v1	q52v2	q52v3	q53i	q53i1	q53i2
88	Ye	88	88	88	No	88	88	88	No	88	88	88	88	88	88
88	Ye	88	88	88	No	88	88	88	No	88	88	88	88	88	88
88	Ye	88	88	88	No	88	88	88	No	88	88	88	88	88	88
88	Ye	88	88	88	No	88	88	88	No	88	88	88	88	88	88
88	Ye	88	88	88	No	88	88	88	Ye	88	88	88	No	88	88
88	Ye	88	88	88	No	88	88	88	No	88	88	88	88	88	88
88	Ye	88	Ye	88	No	88	No	88	No	88	No	88	88	88	88
88	Ye	88	No	88	No	88	No	88	No	88	88	88	88	88	88
88	Ye	88	88	88	No	88	88	88	No	88	88	88	88	88	88
88	Ye	88	88	88	No	88	88	88	No	88	88	88	88	88	88
88	88	88	88	88	88	88	88	88	88	88	88	88	88	88	88
88	Ye	88	88	88	No	88	88	88	No	88	88	88	88	88	88
88	Ye	88	Ye	88	No	88	No	88	No	88	88	88	88	88	88
88	Ye	88	Ye	88	No	88	No	88	No	88	88	88	88	88	88
88	Ye	88	88	88	No	88	88	88	No	88	88	88	88	88	88
88	Ye	88	88	88	No	88	88	88	No	88	88	88	88	88	88
88	Ye	88	88	88	No	88	88	88	No	88	88	88	88	88	88
88	Ye	88	88	88	No	88	88	88	No	88	88	88	88	88	88
88	Ye	88	Ye	88	No	88	No	88	No	88	88	88	88	88	88
88	Ye	88	88	88	No	88	88	88	No	88	88	88	88	88	88
88	Ye	88	88	88	No	88	88	88	No	88	88	88	88	88	88
88	88	88	88	88	88	88	88	88	88	88	88	88	88	88	88
88	Ye	88	88	88	No	88	88	88	No	88	88	88	88	88	88
88	Ye	88	88	88	No	88	88	88	No	88	88	88	88	88	88
88	Ye	88	88	88	No	88	88	88	No	88	88	88	88	88	88
88	Ye	88	88	88	No	88	88	88	No	88	88	88	88	88	88
88	Ye	88	88	88	No	88	88	88	No	88	88	88	88	88	88
88	88	88	88	88	88	88	88	88	88	88	88	88	88	88	88
88	Ye	88	88	88	No	88	88	88	No	88	88	88	88	88	88
88	Ye	88	88	88	No	88	88	88	No	88	88	88	88	88	88
88	Ye	88	88	88	No	88	88	88	No	88	88	88	88	88	88
88	Ye	88	88	88	Ye	88	88	88	Ye	88	88	88	No	88	88
88	Ye	88	88	88	No	88	88	88	No	88	88	88	88	88	88
88	Ye	88	Ye	88	No	88	No	88	No	88	88	88	88	88	88
88	Ye	88	Ye	88	No	88	No	88	No	88	88	88	88	88	88
88	Ye	88	Ye	88	No	88	No	88	No	88	88	88	88	88	88
88	88	88	88	88	88	88	88	88	88	88	88	88	88	88	88
88	88	88	88	88	88	88	88	88	88	88	88	88	88	88	88
88	Ye	88	Ye	88	No	88	No	88	No	88	88	88	88	88	88
88	Ye	88	Ye	88	No	88	No	88	No	88	88	88	88	88	88
88	Ye	88	Ye	88	No	88	No	88	No	88	88	88	88	88	88
88	Ye	88	Ye	88	No	88	No	88	No	88	88	88	88	88	88
88	Ye	88	Ye	88	No	88	No	88	No	88	88	88	88	88	88
88	Ye	88	88	88	No	88	88	88	No	88	88	88	88	88	88
88	Ye	88	88	88	Ye	88	88	88	No	88	88	88	88	88	88
88	Ye	88	Ye	88	No	88	No	88	No	88	88	88	88	88	88

88	Ye	88	88	88	No	88	88	88	No	88	88	88	88	88	88
88	Ye	88	88	88	Ye	88	88	88	No	88	88	88	88	88	88
88	Ye	88	88	88	No	88	88	88	No	88	88	88	88	88	88
88	Ye	88	Ye	88	Ye	88	No	88	No	88	88	88	88	88	88
88	Ye	88	88	88	No	88	88	88	No	88	88	88	88	88	88
88	Ye	88	88	88	No	88	88	88	No	88	88	88	88	88	88
88	Ye	88	88	88	No	88	88	88	No	88	88	88	88	88	88
88	Ye	88	88	88	No	88	88	88	No	88	88	88	88	88	88
88	Ye	88	88	88	No	88	88	88	No	88	88	88	88	88	88
88	Ye	88	88	88	No	88	88	88	No	88	88	88	88	88	88
88	Ye	88	88	88	No	88	88	88	No	88	88	88	88	88	88
88	Ye	88	88	88	No	88	88	88	No	88	88	88	88	88	88
88	Ye	88	88	88	No	88	88	88	No	88	88	88	88	88	88
88	Ye	88	88	88	No	88	88	88	No	88	88	88	88	88	88
88	Ye	88	88	88	No	88	88	88	No	88	88	88	88	88	88
88	Ye	88	88	88	No	88	88	88	No	88	88	88	88	88	88
88	88	88	88	88	88	88	88	88	88	88	88	88	88	88	88
88	Ye	88	88	88	No	88	88	88	No	88	88	88	88	88	88
88	Ye	88	88	88	Ye	88	88	88	No	88	88	88	88	88	88
88	Ye	88	Ye	88	No	88	No	88	No	88	88	88	88	88	88
88	Ye	88	88	88	Ye	88	88	88	Ye	88	88	88	No	88	88
88	Ye	88	88	88	No	88	88	88	No	88	88	88	88	88	88
88	Ye	88	88	88	No	88	88	88	No	88	88	88	88	88	88
88	Ye	88	88	88	No	88	88	88	No	88	88	88	88	88	88
88	Ye	88	88	88	No	88	88	88	No	88	88	88	88	88	88
88	Ye	88	88	88	No	88	88	88	No	88	88	88	88	88	88
88	Ye	88	88	88	No	88	88	88	No	88	88	88	88	88	88
88	Ye	88	88	88	No	88	88	88	No	88	88	88	88	88	88
88	88	88	88	88	88	88	88	88	88	88	88	88	88	88	88
88	Ye	88	88	88	Ye	88	88	88	No	88	88	88	88	88	88
88	Ye	88	88	88	No	88	88	88	No	88	88	88	88	88	88
88	Ye	88	88	88	No	88	88	88	No	88	88	88	88	88	88
88	Ye	88	88	88	No	88	88	88	No	88	88	88	88	88	88
88	Ye	88	88	88	No	88	88	88	No	88	88	88	88	88	88
88	Ye	88	88	88	No	88	88	88	No	88	88	88	88	88	88
88	Ye	88	88	88	No	88	88	88	No	88	88	88	88	88	88
88	Ye	88	88	88	No	88	88	88	No	88	88	88	88	88	88
88	Ye	88	88	88	No	88	88	88	No	88	88	88	88	88	88
88	Ye	88	88	88	No	88	88	88	No	88	88	88	88	88	88
88	Ye	88	88	88	No	88	88	88	No	88	88	88	88	88	88
88	Ye	88	Ye	88	No	88	No	88	No	88	88	88	88	88	88
88	Ye	88	88	88	No	88	88	88	No	88	88	88	88	88	88
88	88	88	88	88	88	88	88	88	88	88	88	88	88	88	88
88	88	88	88	88	88	88	88	88	88	88	88	88	88	88	88
88	88	88	88	88	88	88	88	88	88	88	88	88	88	88	88
88	Ye	88	88	88	No	88	88	88	No	88	88	88	88	88	88
88	Ye	88	88	88	No	88	88	88	No	88	88	88	88	88	88

88	Ye	88	88	88	No	88	88	88	No	88	88	88	88	88	88
88	88	88	88	88	88	88	88	88	88	88	88	88	88	88	88
88	Ye	88	88	88	No	88	88	88	No	88	88	88	88	88	88
88	88	88	88	88	88	88	88	88	88	88	88	88	88	88	88
88	Ye	88	88	88	No	88	88	88	No	88	88	88	88	88	88
88	Ye	88	88	88	No	88	88	88	No	88	88	88	88	88	88
88	Ye	88	88	88	No	88	88	88	No	88	88	88	88	88	88
88	Ye	88	88	88	No	88	88	88	No	88	88	88	88	88	88
88	Ye	88	88	88	No	88	88	88	No	88	88	88	88	88	88
88	Ye	88	88	88	No	88	88	88	No	88	88	88	88	88	88
88	Ye	88	88	88	No	88	88	88	No	88	88	88	88	88	88
88	Ye	88	88	88	No	88	88	88	No	88	88	88	88	88	88
88	Ye	88	88	88	No	88	88	88	No	88	88	88	88	88	88
88	Ye	88	88	88	No	88	88	88	No	88	88	88	88	88	88
88	Ye	88	88	88	No	88	88	88	No	88	88	88	88	88	88
88	Ye	88	88	88	No	88	88	88	No	88	88	88	88	88	88
88	Ye	88	88	88	No	88	88	88	No	88	88	88	88	88	88
88	88	88	88	88	88	88	88	88	88	88	88	88	88	88	88
88	Ye	88	88	88	Ye	88	88	88	No	88	88	88	88	88	88
88	Ye	88	88	88	No	88	88	88	No	88	88	88	88	88	88
88	Ye	88	88	88	No	88	88	88	No	88	88	88	88	88	88
88	Ye	88	88	88	No	88	88	88	No	88	88	88	88	88	88
88	Ye	88	88	88	No	88	88	88	No	88	88	88	88	88	88
88	88	88	88	88	88	88	88	88	88	88	88	88	88	88	88
88	Ye	88	88	88	No	88	88	88	No	88	88	88	88	88	88
88	Ye	88	88	88	No	88	88	88	No	88	88	88	88	88	88
88	Ye	88	88	88	No	88	88	88	No	88	88	88	88	88	88
88	Ye	88	88	88	No	88	88	88	No	88	88	88	88	88	88
88	Ye	88	88	88	No	88	88	88	No	88	88	88	88	88	88
88	Ye	88	88	88	Ye	88	88	88	No	88	88	88	88	88	88
88	Ye	88	Ye	88	No	88	No	88	No	88	88	88	88	88	88
88	Ye	88	88	88	No	88	88	88	No	88	88	88	88	88	88
88	88	88	88	88	88	88	88	88	88	88	88	88	88	88	88
88	Ye	88	88	88	No	88	88	88	No	88	88	88	88	88	88
88	Ye	88	88	88	No	88	88	88	No	88	88	88	88	88	88
88	Ye	88	88	88	No	88	88	88	No	88	88	88	88	88	88
88	Ye	88	88	88	No	88	88	88	No	88	88	88	88	88	88
88	Ye	88	88	88	No	88	88	88	No	88	88	88	88	88	88
88	Ye	88	88	88	No	88	88	88	No	88	88	88	88	88	88
88	Ye	88	88	88	No	88	88	88	No	88	88	88	88	88	88
88	Ye	88	88	88	No	88	88	88	No	88	88	88	88	88	88
88	Ye	88	88	88	No	88	88	88	No	88	88	88	88	88	88
88	Ye	88	88	88	No	88	88	88	No	88	88	88	88	88	88
88	88	88	88	88	88	88	88	88	88	88	88	88	88	88	88
88	Ye	88	88	88	No	88	88	88	No	88	88	88	88	88	88
88	Ye	88	88	88	No	88	88	88	No	88	88	88	88	88	88
88	88	88	88	88	88	88	88	88	88	88	88	88	88	88	88

88	Ye	88	88	88	No	88	88	88	No	88	88	88	88	88	88
88	Ye	88	Ye	88	No	88	No	88	No	88	88	88	88	88	88
88	Ye	88	Ye	88	No	88	No	88	No	88	88	88	88	88	88
88	Ye	88	88	88	No	88	88	88	No	88	88	88	88	88	88
88	Ye	88	88	88	Ye	88	88	88	No	88	88	88	88	88	88
88	Ye	88	88	88	No	88	88	88	No	88	88	88	88	88	88
88	Ye	88	88	88	No	88	88	88	No	88	88	88	88	88	88
88	Ye	88	88	88	No	88	88	88	No	88	88	88	88	88	88
88	Ye	88	88	88	No	88	88	88	No	88	88	88	88	88	88
88	Ye	88	88	88	No	88	88	88	No	88	88	88	88	88	88
88	88	88	88	88	88	88	88	88	88	88	88	88	88	88	88
88	Ye	88	88	88	No	88	88	88	No	88	88	88	88	88	88
88	Ye	88	88	88	No	88	88	88	No	88	88	88	88	88	88
88	Ye	88	88	88	No	88	88	88	No	88	88	88	88	88	88
88	Ye	88	88	88	No	88	88	88	No	88	88	88	88	88	88
88	88	88	88	88	88	88	88	88	88	88	88	88	88	88	88
88	Ye	88	88	88	No	88	88	88	No	88	88	88	88	88	88
88	Ye	88	88	88	No	88	88	88	No	88	88	88	88	88	88
88	Ye	88	88	88	No	88	88	88	No	88	88	88	88	88	88
88	Ye	88	88	88	No	88	88	88	No	88	88	88	88	88	88
88	Ye	88	88	88	No	88	88	88	No	88	88	88	88	88	88
88	Ye	88	88	88	No	88	88	88	No	88	88	88	88	88	88
88	Ye	88	88	88	No	88	88	88	No	88	88	88	88	88	88
88	Ye	88	88	88	No	88	88	88	No	88	88	88	88	88	88
88	88	88	88	88	88	88	88	88	88	88	88	88	88	88	88
88	Ye	88	88	88	No	88	88	88	No	88	88	88	88	88	88
88	Ye	88	88	88	No	88	88	88	No	88	88	88	88	88	88
88	Ye	88	88	88	No	88	88	88	No	88	88	88	88	88	88
88	88	88	88	88	88	88	88	88	88	88	88	88	88	88	88
88	Ye	88	Ye	88	No	88	No	88	No	88	88	88	88	88	88
88	Ye	88	88	88	Ye	88	88	88	No	88	88	88	88	88	88
88	Ye	88	88	88	No	88	88	88	No	88	88	88	88	88	88
88	Ye	88	88	88	No	88	88	88	No	88	88	88	88	88	88
88	Ye	88	88	88	No	88	88	88	No	88	88	88	88	88	88
88	Ye	88	88	88	No	88	88	88	No	88	88	88	88	88	88
88	Ye	88	88	88	No	88	88	88	No	88	88	88	88	88	88
88	Ye	88	88	88	No	88	88	88	No	88	88	88	88	88	88
88	Ye	88	88	88	No	88	88	88	No	88	88	88	88	88	88
88	Ye	88	88	88	No	88	88	88	No	88	88	88	88	88	88
88	Ye	88	88	88	Ye	88	88	88	No	88	88	88	88	88	88
88	Ye	88	88	88	No	88	88	88	No	88	88	88	88	88	88
88	Ye	88	88	88	Ye	88	88	88	Ye	88	88	88	No	88	88
88	Ye	88	88	88	No	88	88	88	No	88	88	88	88	88	88
88	88	88	88	88	88	88	88	88	88	88	88	88	88	88	88
88	Ye	88	88	88	No	88	88	88	No	88	88	88	88	88	88
88	Ye	88	88	88	Ye	88	88	88	No	88	88	88	88	88	88
88	Ye	88	88	88	No	88	88	88	No	88	88	88	88	88	88

88	Ye	88	88	88	No	88	88	88	No	88	88	88	88	88	88
88	88	88	88	88	88	88	88	88	88	88	88	88	88	88	88
88	Ye	88	88	88	No	88	88	88	No	88	88	88	88	88	88
88	Ye	88	88	88	No	88	88	88	No	88	88	88	88	88	88
88	Ye	88	88	88	No	88	88	88	No	88	88	88	88	88	88
88	Ye	88	88	88	No	88	88	88	No	88	88	88	88	88	88
88	Ye	88	88	88	No	88	88	88	No	88	88	88	88	88	88
88	Ye	88	88	88	No	88	88	88	No	88	88	88	88	88	88
88	Ye	88	88	88	No	88	88	88	No	88	88	88	88	88	88
88	Ye	88	88	88	No	88	88	88	No	88	88	88	88	88	88
88	Ye	88	88	88	No	88	88	88	No	88	88	88	88	88	88
88	Ye	88	88	88	No	88	88	88	No	88	88	88	88	88	88
88	Ye	88	88	88	No	88	88	88	No	88	88	88	88	88	88
88	Ye	88	88	88	No	88	88	88	No	88	88	88	88	88	88
88	No	88	88	88	No	88	88	88	No	88	88	88	88	88	88
88	Ye	88	88	88	No	88	88	88	No	88	88	88	88	88	88
88	Ye	88	88	88	No	88	88	88	No	88	88	88	88	88	88
88	Ye	88	88	88	No	88	88	88	No	88	88	88	88	88	88
88	Ye	88	88	88	Ye	88	88	88	No	88	88	88	88	88	88
88	Ye	88	88	88	No	88	88	88	No	88	88	88	88	88	88
88	Ye	88	88	88	No	88	88	88	No	88	88	88	88	88	88
88	Ye	88	88	88	No	88	88	88	No	88	88	88	88	88	88
88	Ye	88	88	88	Ye	88	88	88	No	88	88	88	88	88	88
88	Ye	88	88	88	No	88	88	88	No	88	88	88	88	88	88
88	Ye	88	88	88	No	88	88	88	No	88	88	88	88	88	88
88	88	88	88	88	88	88	88	88	88	88	88	88	88	88	88
88	Ye	88	88	88	No	88	88	88	No	88	88	88	88	88	88
88	Ye	88	88	88	No	88	88	88	No	88	88	88	88	88	88
88	Ye	88	88	88	No	88	88	88	No	88	88	88	88	88	88
88	Ye	88	88	88	No	88	88	88	No	88	88	88	88	88	88
88	Ye	88	88	88	No	88	88	88	No	88	88	88	88	88	88
88	Ye	88	88	88	No	88	88	88	No	88	88	88	88	88	88
88	Ye	88	88	88	No	88	88	88	No	88	88	88	88	88	88
88	Ye	88	88	88	No	88	88	88	No	88	88	88	88	88	88
88	Ye	88	88	88	No	88	88	88	No	88	88	88	88	88	88
88	88	88	88	88	88	88	88	88	88	88	88	88	88	88	88
88	Ye	88	88	88	No	88	88	88	No	88	88	88	88	88	88
88	Ye	88	88	88	Ye	88	88	88	No	88	88	88	88	88	88
88	Ye	88	88	88	No	88	88	88	No	88	88	88	88	88	88
88	Ye	88	88	88	No	88	88	88	No	88	88	88	88	88	88
88	Ye	88	88	88	No	88	88	88	No	88	88	88	88	88	88
88	Ye	88	88	88	No	88	88	88	No	88	88	88	88	88	88
88	Ye	88	88	88	No	88	88	88	No	88	88	88	88	88	88
88	Ye	88 Ye		88	No	88 No		88	No	88	88	88	88	88	88
88	Ye	88	88	88	No	88	88	88	No	88	88	88	88	88	88
88	Ye	88	88	88	No	88	88	88	No	88	88	88	88	88	88
88	Ye	88	88	88	No	88	88	88	No	88	88	88	88	88	88

88	Ye	88	88	88	No	88	88	88	No	88	88	88	88	88	88
88	Ye	88	88	88	Ye	88	88	88	No	88	88	88	88	88	88
88	Ye	88	88	88	No	88	88	88	No	88	88	88	88	88	88
88	Ye	88	88	88	No	88	88	88	No	88	88	88	88	88	88
88	Ye	88	88	88	No	88	88	88	No	88	88	88	88	88	88
88	Ye	88	88	88	No	88	88	88	No	88	88	88	88	88	88
88	Ye	88	88	88	No	88	88	88	No	88	88	88	88	88	88
88	Ye	88	88	88	No	88	88	88	No	88	88	88	88	88	88
88	Ye	88	88	88	No	88	88	88	No	88	88	88	88	88	88
88	88	88	88	88	88	88	88	88	88	88	88	88	88	88	88
88	Ye	88	88	88	No	88	88	88	No	88	88	88	88	88	88
88	Ye	88	Ye	88	No	88	No	88	No	88	88	88	88	88	88
88	Ye	88	88	88	No	88	88	88	No	88	88	88	88	88	88
88	Ye	88	88	88	No	88	88	88	No	88	88	88	88	88	88
88	Ye	88	88	88	No	88	88	88	No	88	88	88	88	88	88
88	Ye	88	88	88	No	88	88	88	No	88	88	88	88	88	88
88	Ye	88	88	88	No	88	88	88	No	88	88	88	88	88	88
88	88	88	88	88	88	88	88	88	88	88	88	88	88	88	88
88	Ye	88	88	88	No	88	88	88	No	88	88	88	88	88	88
88	Ye	88	88	88	No	88	88	88	No	88	88	88	88	88	88
88	Ye	88	88	88	No	88	88	88	No	88	88	88	88	88	88
88	Ye	88	88	88	No	88	88	88	No	88	88	88	88	88	88
88	Ye	88	88	88	No	88	88	88	No	88	88	88	88	88	88
88	Ye	88	88	88	No	88	88	88	No	88	88	88	88	88	88
88	Ye	88	88	88	No	88	88	88	No	88	88	88	88	88	88
88	88	88	88	88	88	88	88	88	88	88	88	88	88	88	88
88	Ye	88	88	88	No	88	88	88	No	88	88	88	88	88	88
88	Ye	88	88	88	Ye	88	88	88	No	88	88	88	88	88	88
88	Ye	88	88	88	Ye	88	88	88	No	88	88	88	88	88	88
88	88	88	88	88	88	88	88	88	88	88	88	88	88	88	88
88	Ye	88	88	88	No	88	88	88	No	88	88	88	88	88	88
88	Ye	88	88	88	No	88	88	88	No	88	88	88	88	88	88
88	Ye	88	88	88	No	88	88	88	No	88	88	88	88	88	88
88	Ye	88	88	88	No	88	88	88	No	88	88	88	88	88	88
88	Ye	88	Ye	88	No	88	No	88	No	88	88	88	88	88	88
88	Ye	88	Ye	88	No	88	No	88	No	88	88	88	88	88	88
88	Ye	88	88	88	No	88	88	88	No	88	88	88	88	88	88
88	Ye	88	88	88	No	88	88	88	No	88	88	88	88	88	88
88	Ye	88	88	88	No	88	88	88	No	88	88	88	88	88	88
88	Ye	88	88	88	No	88	88	88	No	88	88	88	88	88	88
88	Ye	88	Ye	88	No	88	No	88	Ye	88	Ye	88	88	88	88
88	Ye	88	Ye	88	No	88	No	88	No	88	88	88	88	88	88
88	88	88	Ye	88	88	88	No	88	88	88	No	88	88	88	88
88	Ye	88	88	88	No	88	88	88	No	88	88	88	88	88	88
88	Ye	88	88	88	No	88	88	88	No	88	88	88	88	88	88
88	Ye	88	88	88	No	88	88	88	No	88	88	88	88	88	88
88	Ye	88	88	88	No	88	88	88	No	88	88	88	88	88	88

88	Ye	88	88	88	Ye	88	88	88	No	88	88	88	88	88	88
88	Ye	88	88	88	No	88	88	88	No	88	88	88	88	88	88
88	Ye	88	88	88	No	88	88	88	No	88	88	88	88	88	88
88	Ye	88	88	88	No	88	88	88	No	88	88	88	88	88	88
88	Ye	88	88	88	No	88	88	88	No	88	88	88	88	88	88
88	88	88	88	88	88	88	88	88	88	88	88	88	88	88	88
88	Ye	88	88	88	No	88	88	88	No	88	88	88	88	88	88
88	Ye	88	88	88	Ye	88	88	88	No	88	88	88	88	88	88
88	Ye	88	88	88	No	88	88	88	No	88	88	88	88	88	88
88	Ye	88	88	88	No	88	88	88	No	88	88	88	88	88	88
88	Ye	88	88	88	No	88	88	88	No	88	88	88	88	88	88
88	Ye	88	88	88	No	88	88	88	No	88	88	88	88	88	88
88	88	88	88	88	88	88	88	88	88	88	88	88	88	88	88
88	Ye	88	88	88	No	88	88	88	No	88	88	88	88	88	88
88	Ye	88	88	88	No	88	88	88	No	88	88	88	88	88	88
88	Ye	88	88	88	No	88	88	88	No	88	88	88	88	88	88
88	Ye	88	88	88	No	88	88	88	No	88	88	88	88	88	88
88	Ye	88	88	88	No	88	88	88	No	88	88	88	88	88	88

q53i3	q54i	q55w	q55w1	q55w2	q55w3	q56d	q56d1	q56d2	q56d3	q57i	q57i1	q57i2	q57i3	q57anti
88	88	Mo	NA	NA	NA	N	8	8	8	Me	88	88	88	NA
88	88	Ot	NA	NA	NA	N	8	8	8	Me	88	88	88	NA
88	88	NA	NA	NA	NA	N	8	8	8	Me	88	88	88	NA
88	88	NA	NA	NA	NA	N	8	8	8	Me	88	88	88	NA
88	88	Fa	NA	NA	NA	N	8	8	8	Me	88	88	88	NA
88	88	Mo	NA	NA	NA	N	8	8	8	Me	88	88	88	NA
88	88	Fa	NA	Fa	NA	N	8	8	8	Fo	88	88	88	NA
88	88	Fa	NA	Fa	NA	Y	8	8	8	88	88	88	88	NA
88	88	Mo	NA	NA	NA	N	8	8	8	Me	88	88	88	NA
88	88	Mo	NA	NA	NA	N	8	8	8	Me	88	88	88	NA
88	88	NA	NA	NA	NA	Y	8	8	8	88	88	88	88	NA
88	88	Mo	NA	NA	NA	N	8	8	8	Me	88	88	88	NA
88	88	Fa	NA	Fa	NA	Y	8	8	8	88	88	88	88	NA
88	88	Mo	NA	Mo	NA	N	8	8	8	ot	88	88	88	NA
88	88	Fa	NA	NA	NA	N	8	8	8	Me	88	88	88	NA
88	88	NA	NA	NA	NA	N	8	8	8	Me	88	88	88	NA
88	88	NA	NA	NA	NA	N	8	8	8	Me	88	88	88	NA
88	88	NA	NA	NA	NA	N	8	8	8	Me	88	88	88	NA
88	88	NA	NA	NA	NA	Y	8	8	8	88	88	88	88	NA
88	88	Mo	NA	NA	NA	N	8	8	8	Me	88	88	88	NA
88	88	Mo	NA	NA	NA	N	8	8	8	Me	88	88	88	NA
88	88	NA	NA	NA	NA	Y	8	8	8	88	88	88	88	NA
88	88	Mo	NA	NA	NA	N	8	8	8	Me	88	88	88	NA
88	88	NA	NA	NA	NA	N	8	8	8	Me	88	88	88	NA
88	88	Mo	NA	NA	NA	N	8	8	8	Me	88	88	88	NA
88	88	Mo	NA	NA	NA	N	8	8	8	Me	88	88	88	NA
88	88	Mo	NA	NA	NA	N	8	8	8	Me	88	88	88	NA
88	88	NA	NA	NA	NA	N	8	8	8	No	88	88	88	NA
88	88	NA	NA	NA	NA	Y	8	8	8	88	88	88	88	NA
88	88	Mo	NA	NA	NA	N	8	8	8	Me	88	88	88	NA
88	88	Ot	NA	NA	NA	N	8	8	8	Me	88	88	88	NA
88	88	Fa	NA	NA	NA	N	8	8	8	Me	88	88	88	NA
88	88	Mo	NA	NA	NA	N	8	8	8	Me	88	88	88	NA
88	88	NA	NA	NA	NA	N	8	8	8	Me	88	88	88	NA
88	88	NA	NA	NA	NA	N	8	8	8	Me	88	Me	88	NA
88	88	NA	NA	NA	NA	Y	8	8	8	88	88	88	88	NA
88	88	NA	NA	NA	NA	N	8	8	8	Si	88	88	88	NA
88	88	NA	NA	NA	NA	Y	8	8	8	88	88	88	88	NA
88	88	Mo	NA	Mo	NA	N	8	8	8	Me	88	88	88	NA
88	88	Ot	NA	Ot	NA	N	8	8	8	Me	88	88	88	NA
88	88	Ot	NA	Ot	NA	N	8	8	8	Me	88	88	88	NA
88	88	NA	NA	NA	NA	N	8	8	8	Me	88	88	88	NA
88	88	Mo	NA	Ot	NA	N	8	8	8	ot	88	88	88	NA
88	88	Ot	NA	NA	NA	N	8	8	8	Me	88	88	88	NA
88	88	Mo	NA	NA	NA	N	8	8	8	Me	88	88	88	NA
88	88	Ot	NA	Ot	NA	Y	8	8	8	88	88	88	88	NA

88	88	Ot	NA	NA	NA	N		8		8	8	Me	88	88	88	NA
88	88	Mo	NA	NA	NA	N		8		8	8	Me	88	88	88	NA
88	88	NA	NA	NA	NA	N		8		8	8	Me	88	88	88	NA
88	88	Mo	NA	Mo	NA	Y		8		8	8	88	88	88	88	NA
88	88	Ot	NA	NA	NA	N		8		8	8	Me	88	88	88	NA
88	88	NA	NA	NA	NA	N		8		8	8	Me	88	88	88	NA
88	88	Mo	NA	NA	NA	N		8		8	8	Me	88	88	88	NA
88	88	Mo	NA	NA	NA	N		8		8	8	Me	88	88	88	NA
88	88	NA	NA	NA	NA	N		8		8	8	Me	88	88	88	NA
88	88	NA	NA	NA	NA	N		8		8	8	Me	88	88	88	NA
88	88	Ot	NA	NA	NA	N		8		8	8	Me	88	88	88	NA
88	88	Mo	NA	NA	NA	N		8		8	8	Me	88	88	88	NA
88	88	Mo	NA	NA	NA	N		8		8	8	Me	88	88	88	NA
88	88	Mo	NA	NA	NA	N		8		8	8	Me	88	88	88	NA
88	88	NA	NA	NA	NA	N		8		8	8	Me	88	88	88	NA
88	88	Ot	NA	NA	NA	N		8		8	8	Me	88	88	88	NA
88	88	NA	NA	NA	NA	N		8		8	8	Me	88	88	88	NA
88	88	Mo	NA	NA	NA	N		8		8	8	Me	88	88	88	NA
88	88	Mo	NA	NA	NA	N		8		8	8	Me	88	88	88	NA
88	88	Mo	NA	Mo	NA	N		8		8	8	Me	88	88	88	NA
88	88	Mo	NA	NA	NA	N		8		8	8	Me	88	88	88	NA
88	88	Mo	NA	NA	NA	N		8		8	8	Me	88	88	88	NA
88	88	Mo	NA	NA	NA	N		8		8	8	ot	88	88	88	NA
88	88	NA	NA	NA	NA	N		8		8	8	Me	88	88	88	NA
88	88	Mo	NA	NA	NA	N		8		8	8	Me	88	88	88	NA
88	88	NA	NA	NA	NA	N		8		8	8	Me	88	88	88	NA
88	88	Ot	NA	NA	NA	N		8		8	8	Me	88	88	88	NA
88	88	NA	NA	NA	NA	N		8		8	8	Me	88	88	88	NA
88	88	NA	NA	NA	NA	N		8		8	8	Me	88	88	88	NA
88	88	Mo	NA	NA	NA	N		8		8	8	Me	88	88	88	NA
88	88	Mo	NA	NA	NA	N		8		8	8	Me	88	88	88	NA
88	88	Mo	NA	NA	NA	N		8		8	8	Me	88	88	88	NA
88	88	NA	NA	NA	NA	N		8		8	8	Me	88	88	88	NA
88	88	NA	NA	NA	NA	N		8		8	8	Me	88	88	88	NA
88	88	NA	NA	NA	NA	N		8		8	8	Me	88	88	88	NA
88	88	Mo	NA	NA	NA	N		8		8	8	Me	88	88	88	NA
88	88	Mo	NA	NA	NA	N		8		8	8	Me	88	88	88	NA
88	88	Mo	NA	NA	NA	N		8		8	8	Me	88	88	88	NA
88	88	Mo	NA	NA	NA	N		8		8	8	Me	88	88	88	NA
88	88	Mo	NA	NA	NA	N		8		8	8	Me	88	88	88	NA
88	88	Mo	NA	Mo	NA	N		8		8	8	Me	88	88	88	NA
88	88	Mo	NA	NA	NA	N		8		8	8	Me	88	88	88	NA
88	88	NA	NA	NA	NA	N		8		8	8	88	88	88	88	NA
88	88	NA	NA	NA	NA		8	8		8	8	88	88	88	88	NA
88	88	NA	NA	NA	NA	N		8	N		8	Do	88	88	88	NA
88	88	Mo	NA	NA	NA	N		8		8	8	Me	88	88	88	NA
88	88	NA	NA	NA	NA	N		8		8	8	Me	88	88	88	NA

88	88	NA	NA	NA	NA	N	8	8	8	Me	88	88	88	NA
88	88	NA	NA	NA	NA	Y	8	8	8	88	88	88	88	NA
88	88	NA	NA	NA	NA	N	8	8	8	Me	88	88	88	NA
88	88	NA	NA	NA	NA	N	8	8	8	Si	88	88	88	NA
88	88	Mo	NA	NA	NA	N	8	8	8	Me	88	88	88	NA
88	88	Mo	NA	NA	NA	N	8	8	8	Me	88	88	88	NA
88	88	Mo	NA	NA	NA	N	8	8	8	Me	88	88	88	NA
88	88	Mo	NA	NA	NA	N	8	8	8	Me	88	88	88	NA
88	88	Mo	NA	NA	NA	N	8	8	8	Me	88	88	88	NA
88	88	Mo	NA	NA	NA	N	8	8	8	Me	88	88	88	NA
88	88	Mo	NA	NA	NA	N	8	8	8	Me	88	88	88	NA
88	88	Mo	NA	NA	NA	N	8	8	8	Me	88	88	88	NA
88	88	Mo	NA	NA	NA	N	8	8	8	Me	88	88	88	NA
88	88	Fa	NA	NA	NA	Y	8	8	8	88	88	88	88	NA
88	88	NA	NA	NA	NA	N	8	8	8	Me	88	88	88	NA
88	88	Mo	NA	NA	NA	N	8	8	8	Me	88	88	88	NA
88	88	NA	NA	NA	NA	N	8	8	8	Me	88	88	88	NA
88	88	NA	NA	NA	NA	N	8	8	8	ot	88	88	88	NA
88	88	Mo	NA	NA	NA	N	8	8	8	Me	88	88	88	NA
88	88	Ot	NA	NA	NA	N	8	8	8	Me	88	88	88	NA
88	88	Mo	NA	NA	NA	N	8	8	8	Me	88	88	88	NA
88	88	Mo	NA	NA	NA	N	8	8	8	Me	88	88	88	NA
88	88	Fa	NA	NA	NA	N	8	8	8	Me	88	88	88	NA
88	88	NA	NA	NA	NA	Y	8	8	8	88	88	88	88	NA
88	88	Mo	NA	NA	NA	N	8	8	8	Me	88	88	88	NA
88	88	Mo	NA	NA	NA	N	8	8	8	Me	88	88	88	NA
88	88	Mo	NA	NA	NA	N	8	8	8	Me	88	88	88	NA
88	88	NA	NA	NA	NA	N	8	8	8	Me	88	88	88	NA
88	88	NA	NA	NA	NA	N	8	8	8	Me	88	88	88	NA
88	88	Mo	NA	NA	NA	N	8	8	8	Me	88	88	88	NA
88	88	Mo	NA	Mo	NA	Y	8	8	8	88	88	88	88	NA
88	88	Ot	NA	NA	NA	N	8	8	8	Me	88	88	88	NA
88	88	NA	NA	NA	NA	Y	8	8	8	88	88	88	88	NA
88	88	Mo	NA	NA	NA	Y	8	8	8	88	88	88	88	NA
88	88	Mo	NA	NA	NA	N	8	8	8	Do	88	88	88	NA
88	88	Mo	NA	NA	NA	N	8	8	8	Me	88	88	88	NA
88	88	Mo	NA	NA	NA	N	8	8	8	Me	88	88	88	NA
88	88	Mo	NA	NA	NA	N	8	8	8	Me	88	88	88	NA
88	88	NA	NA	NA	NA	N	8	8	8	Me	88	88	88	NA
88	88	NA	NA	NA	NA	N	8	8	8	Me	88	88	88	NA
88	88	Ot	NA	NA	NA	N	8	8	8	Me	88	88	88	NA
88	88	NA	NA	NA	NA	N	8	8	8	Me	88	88	88	NA
88	88	NA	NA	NA	NA	N	8	8	8	Me	88	88	88	NA
88	88	NA	NA	NA	NA	N	8	8	8	88	88	88	88	NA
88	88	Mo	NA	NA	NA	N	8	8	8	Me	88	88	88	NA
88	88	NA	NA	NA	NA	N	8	8	8	Me	88	88	88	NA
88	88	NA	NA	NA	NA	N	8	8	8	ot	88	88	88	NA

88	88	NA	NA	NA	NA	N	8	8	8	ot	88	88	88	NA
88	88	Fa	NA	Fa	NA	N	8	8	8	ot	88	88	88	NA
88	88	Mo	NA	Mo	NA	N	8	8	8	Do	88	88	88	NA
88	88	NA	NA	NA	NA	Y	8	8	8	88	88	88	88	NA
88	88	Mo	NA	NA	NA	N	8	8	8	Me	88	88	88	NA
88	88	NA	NA	NA	NA	N	8	8	8	Me	88	88	88	NA
88	88	Mo	NA	NA	NA	N	8	8	8	Me	88	88	88	NA
88	88	Mo	NA	NA	NA	N	8	8	8	Me	88	88	88	NA
88	88	Mo	NA	NA	NA	N	8	8	8	Me	88	88	88	NA
88	88	Mo	NA	NA	NA	N	8	8	8	Do	88	88	88	NA
88	88	NA	NA	NA	NA	N	8	8	8	No	88	88	88	NA
88	88	Mo	NA	NA	NA	N	8	8	8	Me	88	88	88	NA
88	88	Mo	NA	NA	NA	N	8	8	8	Me	88	88	88	NA
88	88	Mo	NA	NA	NA	N	8	8	8	Me	88	88	88	NA
88	88	Mo	NA	NA	NA	N	8	8	8	Me	88	88	88	NA
88	88	NA	NA	NA	NA	Y	8	8	8	88	88	88	88	NA
88	88	Mo	NA	NA	NA	N	8	8	8	Me	88	88	88	NA
88	88	Mo	NA	NA	NA	N	8	8	8	Me	88	88	88	NA
88	88	Ot	NA	NA	NA	N	8	8	8	Me	88	88	88	NA
88	88	Mo	NA	NA	NA	N	8	8	8	Me	88	88	88	NA
88	88	Mo	NA	NA	NA	N	8	8	8	Me	88	88	88	NA
88	88	Mo	NA	NA	NA	Y	8	8	8	88	88	88	88	NA
88	88	Mo	NA	NA	NA	N	8	8	8	Me	88	88	88	NA
88	88	Mo	NA	NA	NA	N	8	8	8	Me	88	88	88	NA
88	88	NA	NA	NA	NA	N	8	8	8	Me	88	88	88	NA
88	88	Mo	NA	NA	NA	N	8	8	8	Me	88	88	88	NA
88	88	Mo	NA	NA	NA	N	8	8	8	Me	88	88	88	NA
88	88	Mo	NA	NA	NA	Y	8	8	8	88	88	88	88	NA
88	88	NA	NA	NA	NA	N	8	8	8	Me	88	88	88	NA
88	88	Mo	NA	Mo	NA	N	8	8	8	ot	88	88	88	NA
88	88	Mo	NA	NA	NA	N	8	8	8	Me	88	88	88	NA
88	88	Mo	NA	NA	NA	N	8	8	8	Me	88	88	88	NA
88	88	Mo	NA	NA	NA	N	8	8	8	Me	88	88	88	NA
88	88	Mo	NA	NA	NA	N	8	8	8	Me	88	88	88	NA
88	88	Mo	NA	NA	NA	N	8	8	8	Me	88	88	88	NA
88	88	NA	NA	NA	NA	N	8	8	8	Me	88	88	88	NA
88	88	Mo	NA	NA	NA	N	8	8	8	Me	88	88	88	NA
88	88	Mo	NA	NA	NA	N	8	8	8	Me	88	88	88	NA
88	88	Fa	NA	NA	NA	N	8	8	8	Me	88	88	88	NA
88	88	Mo	NA	NA	NA	N	8	8	8	Me	88	88	88	NA
88	88	Mo	NA	NA	NA	N	8	8	8	Me	88	88	88	NA
88	88	Mo	NA	NA	NA	N	8	8	8	Me	88	88	88	NA
88	88	NA	NA	NA	NA	N	8	8	8	Me	88	88	88	NA
88	88	NA	NA	NA	NA	N	8	8	8	ot	88	88	88	NA
88	88	Mo	NA	NA	NA	N	8	8	8	Me	88	88	88	NA
88	88	Mo	NA	NA	NA	N	8	8	8	Me	88	88	88	NA
88	88	Mo	NA	NA	NA	N	8	8	8	Me	88	88	88	NA

88	88	Mo	NA	NA	NA	N	8	8	8	Me	88	88	88	NA
88	88	NA	NA	NA	NA	N	8	8	8	Si	88	88	88	NA
88	88	Mo	NA	NA	NA	N	8	8	8	Me	88	88	88	NA
88	88	Mo	NA	NA	NA	N	8	8	8	Me	88	88	88	NA
88	88	Mo	NA	NA	NA	N	8	8	8	Me	88	88	88	NA
88	88	Ot	NA	NA	NA	N	8	8	8	Me	88	88	88	NA
88	88	Mo	NA	NA	NA	N	8	8	8	Me	88	88	88	NA
88	88	Mo	NA	NA	NA	N	8	8	8	Me	88	88	88	NA
88	88	Mo	NA	NA	NA	N	8	8	8	Me	88	88	88	NA
88	88	Mo	NA	NA	NA	N	8	8	8	Me	88	88	88	NA
88	88	Mo	NA	NA	NA	N	8	8	8	Me	88	88	88	NA
88	88	Mo	NA	NA	NA	N	8	8	8	Me	88	88	88	NA
88	88	Mo	NA	NA	NA	Y	8	8	8	88	88	88	88	NA
88	88	Mo	NA	NA	NA	N	8	8	8	Me	88	88	88	NA
88	88	NA	NA	NA	NA	N	8	8	8	Me	88	88	88	NA
88	88	Mo	NA	NA	NA	N	8	8	8	Me	88	88	88	NA
88	88	Fa	NA	NA	NA	N	8	8	8	Me	88	88	88	NA
88	88	Mo	NA	NA	NA	N	8	8	8	Me	88	88	88	NA
88	88	Mo	NA	NA	NA	N	8	8	8	Me	88	88	88	NA
88	88	NA	NA	NA	NA	N	8	8	8	Me	88	88	88	NA
88	88	NA	NA	NA	NA	N	8	8	8	Do	88	88	88	NA
88	88	Mo	NA	NA	NA	N	8	8	8	Me	88	88	88	NA
88	88	Mo	NA	NA	NA	N	8	8	8	Me	88	88	88	NA
88	88	Mo	NA	NA	NA	N	8	8	8	Me	88	88	88	NA
88	88	NA	NA	NA	NA	N	8	8	8	Me	88	88	88	NA
88	88	NA	NA	NA	NA	Y	8	8	8	88	88	88	88	NA
88	88	Mo	NA	NA	NA	N	8	8	8	Me	88	88	88	NA
88	88	Mo	NA	NA	NA	N	8	8	8	Me	88	88	88	NA
88	88	NA	NA	NA	NA	N	8	8	8	Me	88	88	88	NA
88	88	NA	NA	NA	NA	N	8	8	8	Me	88	88	88	NA
88	88	NA	NA	NA	NA	N	8	8	8	Me	88	88	88	NA
88	88	Mo	NA	NA	NA	N	8	8	8	Me	88	88	88	NA
88	88	NA	NA	NA	NA	N	8	8	8	Me	88	88	88	NA
88	88	NA	NA	NA	NA	N	8	8	8	Me	88	88	88	NA
88	88	NA	NA	NA	NA	N	8	8	8	Me	88	88	88	NA
88	88	NA	NA	NA	NA	N	8	8	8	Me	88	88	88	NA
88	88	Ot	NA	NA	NA	N	8	8	8	Me	88	88	88	NA
88	88	Mo	NA	NA	NA	Y	8	8	8	88	88	88	88	NA
88	88	Ot	NA	NA	NA	N	8	8	8	Me	88	88	88	NA
88	88	Mo	NA	NA	NA	N	8	8	8	Me	88	88	88	NA
88	88	Mo	NA	NA	NA	N	8	8	8	Me	88	88	88	NA
88	88	Ot	NA	NA	NA	N	8	8	8	Me	88	88	88	NA
88	88	Mo	NA	NA	NA	N	8	8	8	Me	88	88	88	NA
88	88	Mo	NA	Mo	NA	Y	8	8	8	88	88	88	88	NA
88	88	NA	NA	NA	NA	N	8	8	8	Me	88	88	88	NA
88	88	NA	NA	NA	NA	N	8	8	8	Me	88	88	88	NA
88	88	Mo	NA	NA	NA	N	8	8	8	Me	88	88	88	NA

88	88	Ot	NA	NA	NA	N	8	8	8	Me	88	88	88	NA
88	88	Mo	NA	NA	NA	N	8	8	8	Me	88	88	88	NA
88	88	Mo	NA	NA	NA	N	8	8	8	Me	88	88	88	NA
88	88	Mo	NA	NA	NA	N	8	8	8	Me	88	88	88	NA
88	88	Mo	NA	NA	NA	N	8	8	8	Me	88	88	88	NA
88	88	Mo	NA	NA	NA	N	8	8	8	Me	88	88	88	NA
88	88	Mo	NA	NA	NA	N	8	8	8	ot	88	88	88	NA
88	88	Mo	NA	NA	NA	N	8	8	8	Me	88	88	88	NA
88	88	Mo	NA	NA	NA	N	8	8	8	ot	88	88	88	NA
88	88	NA	NA	NA	NA	Y	8	8	8	88	88	88	88	NA
88	88	Fa	NA	NA	NA	N	8	8	8	Me	88	88	88	NA
88	88	Mo	NA	Mo	NA	Y	8	8	8	88	88	88	88	NA
88	88	Mo	NA	NA	NA	N	8	8	8	Me	88	88	88	NA
88	88	Mo	NA	NA	NA	N	8	8	8	Me	88	88	88	NA
88	88	Mo	NA	NA	NA	N	8	8	8	Me	88	88	88	NA
88	88	Mo	NA	NA	NA	N	8	8	8	Me	88	88	88	NA
88	88	Mo	NA	NA	NA	N	8	8	8	Me	88	88	88	NA
88	88	NA	NA	NA	NA	N	8	8	8	ot	88	88	88	NA
88	88	Mo	NA	NA	NA	N	8	8	8	Me	88	88	88	NA
88	88	Mo	NA	NA	NA	N	8	8	8	Me	88	88	88	NA
88	88	Fa	NA	NA	NA	N	8	8	8	Me	88	88	88	NA
88	88	Mo	NA	NA	NA	N	8	8	8	Me	88	88	88	NA
88	88	NA	NA	NA	NA	N	8	8	8	Me	88	88	88	NA
88	88	Mo	NA	NA	NA	N	8	8	8	Me	88	88	88	NA
88	88	Mo	NA	NA	NA	Y	8	8	8	88	88	88	88	NA
88	88	NA	NA	NA	NA	N	8	8	8	ot	88	88	88	NA
88	88	Mo	NA	NA	NA	N	8	8	8	Me	88	88	88	NA
88	88	Mo	NA	NA	NA	N	8	8	8	Me	88	88	88	NA
88	88	Mo	NA	NA	NA	N	8	8	8	ot	88	88	88	NA
88	88	NA	NA	NA	NA	N	8	8	8	88	88	88	88	NA
88	88	Mo	NA	NA	NA	N	8	8	8	Me	88	88	88	NA
88	88	Mo	NA	NA	NA	N	8	8	8	Me	88	88	88	NA
88	88	Mo	NA	NA	NA	Y	8	8	8	88	88	88	88	NA
88	88	Mo	NA	NA	NA	N	8	8	8	Me	88	88	88	NA
88	88	Mo	NA	Mo	NA	N	8	8	8	Me	88	88	88	NA
88	88	Mo	NA	Mo	NA	Y	8	8	8	88	88	88	88	NA
88	88	Mo	NA	NA	NA	Y	8	8	8	88	88	88	88	NA
88	88	Mo	NA	NA	NA	N	8	8	8	Me	88	88	88	NA
88	88	Ot	NA	NA	NA	N	8	8	8	Me	88	88	88	NA
88	88	Fa	NA	NA	NA	Y	8	8	8	88	88	88	88	NA
88	88	Mo	NA	Mo	NA	N	8	8	8	Me	88	88	88	NA
88	88	Fa	NA	Fa	NA	Y	8	8	8	88	88	88	88	NA
88	88	NA	NA	Ot	NA	Y	8	8	8	88	88	88	88	NA
88	88	Mo	NA	NA	NA	N	8	8	8	Me	88	88	88	NA
88	88	Ot	NA	NA	NA	N	8	8	8	Me	88	88	88	NA
88	88	Mo	NA	NA	NA	N	8	8	8	Me	88	88	88	NA
88	88	Ot	NA	NA	NA	N	8	8	8	Me	88	88	88	NA

88	88	Mo	NA	NA	NA	N	8	8	8	Me	88	88	88	NA
88	88	Ot	NA	NA	NA	N	8	8	8	Me	88	88	88	NA
88	88	Mo	NA	NA	NA	N	8	8	8	Me	88	88	88	NA
88	88	Mo	NA	NA	NA	N	8	8	8	Me	88	88	88	NA
88	88	Mo	NA	NA	NA	N	8	8	8	Me	88	88	88	NA
88	88	NA	NA	NA	NA	N	8	8	8	Do	88	88	88	NA
88	88	Mo	NA	NA	NA	N	8	8	8	Me	88	88	88	NA
88	88	Mo	NA	NA	NA	N	8	8	8	Me	88	88	88	NA
88	88	Mo	NA	NA	NA	N	8	8	8	Me	88	88	88	NA
88	88	Mo	NA	NA	NA	N	8	8	8	Me	88	88	88	NA
88	88	Mo	NA	NA	NA	N	8	8	8	ot	88	88	88	NA
88	88	Mo	NA	NA	NA	N	8	8	8	Me	88	88	88	NA
88	88	NA	NA	NA	NA	N	8	8	8	Do	88	88	88	NA
88	88	Mo	NA	NA	NA	N	8	8	8	Me	88	88	88	NA
88	88	Mo	NA	NA	NA	N	8	8	8	Me	88	88	88	NA
88	88	Ot	NA	NA	NA	N	8	8	8	Me	88	88	88	NA
88	88	Mo	NA	NA	NA	N	8	8	8	Me	88	88	88	NA
88	88	Mo	NA	NA	NA	N	8	8	8	Me	88	88	88	NA

q57anti1	q57anti2	q57anti3	q57other	q57other1	q57other2	q57other3	q58h	q58h1
NA	NA	NA	NA	NA	NA	NA	88	88
NA	NA	NA	NA	NA	NA	NA	88	88
NA	NA	NA	NA	NA	NA	NA	88	88
NA	NA	NA	NA	NA	NA	NA	88	88
NA	NA	NA	NA	NA	NA	NA	88	88
NA	NA	NA	NA	NA	NA	NA	88	88
NA	NA	NA	NA	NA	NA	NA	88	88
NA	NA	NA	NA	NA	NA	NA	1	88
NA	NA	NA	NA	NA	NA	NA	88	88
NA	NA	NA	NA	NA	NA	NA	88	88
NA	NA	NA	NA	NA	NA	NA	3	88
NA	NA	NA	NA	NA	NA	NA	88	88
NA	NA	NA	NA	NA	NA	NA	1	88
NA	NA	NA	YET TO EAT BEFORE	NA	NA	NA	88	88
NA	NA	NA	NA	NA	NA	NA	88	88
NA	NA	NA	NA	NA	NA	NA	88	88
NA	NA	NA	NA	NA	NA	NA	88	88
NA	NA	NA	NA	NA	NA	NA	88	88
NA	NA	NA	NA	NA	NA	NA	1	88
NA	NA	NA	NA	NA	NA	NA	88	88
NA	NA	NA	NA	NA	NA	NA	88	88
NA	NA	NA	NA	NA	NA	NA	1	88
NA	NA	NA	NA	NA	NA	NA	88	88
NA	NA	NA	NA	NA	NA	NA	88	88
NA	NA	NA	NA	NA	NA	NA	88	88
NA	NA	NA	NA	NA	NA	NA	88	88
NA	NA	NA	NA	NA	NA	NA	88	88
NA	NA	NA	NA	NA	NA	NA	88	88
NA	NA	NA	NA	NA	NA	NA	1	88
NA	NA	NA	NA	NA	NA	NA	88	88
NA	NA	NA	NA	NA	NA	NA	88	88
NA	NA	NA	NA	NA	NA	NA	88	88
NA	NA	NA	NA	NA	NA	NA	88	88
NA	NA	NA	NA	NA	NA	NA	88	88
NA	NA	NA	NA	NA	NA	NA	88	88
NA	NA	NA	NA	NA	NA	NA	1	88
NA	NA	NA	NA	NA	NA	NA	88	88
NA	NA	NA	NA	NA	NA	NA	1	88
NA	NA	NA	NA	NA	NA	NA	88	88
NA	NA	NA	NA	NA	NA	NA	88	88
NA	NA	NA	NA	NA	NA	NA	88	88
NA	NA	NA	NA	NA	NA	NA	88	88
NA	NA	NA	CHILD NOT YET EATING	NA	NA	NA	88	88
NA	NA	NA	NA	NA	NA	NA	88	88
NA	NA	NA	NA	NA	NA	NA	88	88
NA	NA	NA	NA	NA	NA	NA	1	88

NA	NA	NA	NA	NA	NA	NA	88	88
NA	NA	NA	NA	NA	NA	NA	88	88
NA	NA	NA	NA	NA	NA	NA	88	88
NA	NA	NA	NA	NA	NA	NA	2	88
NA	NA	NA	NA	NA	NA	NA	88	88
NA	NA	NA	NA	NA	NA	NA	88	88
NA	NA	NA	NA	NA	NA	NA	88	88
NA	NA	NA	NA	NA	NA	NA	88	88
NA	NA	NA	NA	NA	NA	NA	88	88
NA	NA	NA	NA	NA	NA	NA	88	88
NA	NA	NA	NA	NA	NA	NA	88	88
NA	NA	NA	NA	NA	NA	NA	88	88
NA	NA	NA	NA	NA	NA	NA	88	88
NA	NA	NA	NA	NA	NA	NA	88	88
NA	NA	NA	NA	NA	NA	NA	88	88
NA	NA	NA	NA	NA	NA	NA	88	88
NA	NA	NA	NA	NA	NA	NA	88	88
NA	NA	NA	NA	NA	NA	NA	88	88
NA	NA	NA	NA	NA	NA	NA	88	88
NA	NA	NA	NA	NA	NA	NA	88	88
NA	NA	NA	NA	NA	NA	NA	88	88
NA	NA	NA	NA	NA	NA	NA	88	88
NA	NA	NA	YET TO TAKE MEDICINE	NA	NA	NA	88	88
NA	NA	NA	NA	NA	NA	NA	88	88
NA	NA	NA	NA	NA	NA	NA	88	88
NA	NA	NA	NA	NA	NA	NA	88	88
NA	NA	NA	NA	NA	NA	NA	88	88
NA	NA	NA	NA	NA	NA	NA	88	88
NA	NA	NA	NA	NA	NA	NA	88	88
NA	NA	NA	NA	NA	NA	NA	88	88
NA	NA	NA	NA	NA	NA	NA	88	88
NA	NA	NA	NA	NA	NA	NA	88	88
NA	NA	NA	NA	NA	NA	NA	88	88
NA	NA	NA	NA	NA	NA	NA	88	88
NA	NA	NA	NA	NA	NA	NA	88	88
NA	NA	NA	NA	NA	NA	NA	88	88
NA	NA	NA	NA	NA	NA	NA	88	88
NA	NA	NA	NA	NA	NA	NA	88	88
NA	NA	NA	NA	NA	NA	NA	88	88
NA	NA	NA	NA	NA	NA	NA	88	88
NA	NA	NA	NA	NA	NA	NA	88	88
NA	NA	NA	NA	NA	NA	NA	88	88
NA	NA	NA	NA	NA	NA	NA	88	88
NA	NA	NA	NA	NA	NA	NA	88	88
NA	NA	NA	NA	NA	NA	NA	88	88
NA	NA	NA	NA	NA	NA	NA	88	88
NA	NA	NA	NA	NA	NA	NA	88	88

NA	NA	NA	NA	NA	NA	NA	88	88
NA	NA	NA	NA	NA	NA	NA	2	88
NA	NA	NA	NA	NA	NA	NA	88	88
NA	NA	NA	NA	NA	NA	NA	88	88
NA	NA	NA	NA	NA	NA	NA	88	88
NA	NA	NA	NA	NA	NA	NA	88	88
NA	NA	NA	NA	NA	NA	NA	88	88
NA	NA	NA	NA	NA	NA	NA	88	88
NA	NA	NA	NA	NA	NA	NA	88	88
NA	NA	NA	NA	NA	NA	NA	88	88
NA	NA	NA	NA	NA	NA	NA	88	88
NA	NA	NA	NA	NA	NA	NA	88	88
NA	NA	NA	NA	NA	NA	NA	88	88
NA	NA	NA	NA	NA	NA	NA	2	88
NA	NA	NA	NA	NA	NA	NA	88	88
NA	NA	NA	NA	NA	NA	NA	88	88
NA	NA	NA	NA	NA	NA	NA	88	88
NA	NA	NA	NOT YET EATE	NA	NA	NA	88	88
NA	NA	NA	NA	NA	NA	NA	88	88
NA	NA	NA	NA	NA	NA	NA	88	88
NA	NA	NA	NA	NA	NA	NA	88	88
NA	NA	NA	NA	NA	NA	NA	88	88
NA	NA	NA	NA	NA	NA	NA	88	88
NA	NA	NA	NA	NA	NA	NA	2	88
NA	NA	NA	NA	NA	NA	NA	88	88
NA	NA	NA	NA	NA	NA	NA	88	88
NA	NA	NA	NA	NA	NA	NA	88	88
NA	NA	NA	NA	NA	NA	NA	88	88
NA	NA	NA	NA	NA	NA	NA	88	88
NA	NA	NA	NA	NA	NA	NA	88	88
NA	NA	NA	NA	NA	NA	NA	1	88
NA	NA	NA	NA	NA	NA	NA	88	88
NA	NA	NA	NA	NA	NA	NA	2	88
NA	NA	NA	NA	NA	NA	NA	1	88
NA	NA	NA	NA	NA	NA	NA	88	88
NA	NA	NA	NA	NA	NA	NA	88	88
NA	NA	NA	NA	NA	NA	NA	88	88
NA	NA	NA	NA	NA	NA	NA	88	88
NA	NA	NA	NA	NA	NA	NA	88	88
NA	NA	NA	NA	NA	NA	NA	88	88
NA	NA	NA	NA	NA	NA	NA	88	88
NA	NA	NA	NA	NA	NA	NA	88	88
NA	NA	NA	NA	NA	NA	NA	88	88
NA	NA	NA	NA	NA	NA	NA	88	88
NA	NA	NA	NA	NA	NA	NA	88	88
NA	NA	NA	NA	NA	NA	NA	88	88
NA	NA	NA	YET TO TAKE MEDICINE	NA	NA	NA	88	88

NA	NA	NA	YET TO TAKE MEDICINE	NA	NA	NA	88	88
NA	NA	NA	YET TO TAKE MEDICINE	NA	NA	NA	88	88
NA	NA	NA	NA	NA	NA	NA	88	88
NA	NA	NA	NA	NA	NA	NA	3	88
NA	NA	NA	NA	NA	NA	NA	88	88
NA	NA	NA	NA	NA	NA	NA	88	88
NA	NA	NA	NA	NA	NA	NA	88	88
NA	NA	NA	NA	NA	NA	NA	88	88
NA	NA	NA	NA	NA	NA	NA	88	88
NA	NA	NA	NA	NA	NA	NA	88	88
NA	NA	NA	NA	NA	NA	NA	88	88
NA	NA	NA	NA	NA	NA	NA	88	88
NA	NA	NA	NA	NA	NA	NA	88	88
NA	NA	NA	NA	NA	NA	NA	88	88
NA	NA	NA	NA	NA	NA	NA	88	88
NA	NA	NA	NA	NA	NA	NA	2	88
NA	NA	NA	NA	NA	NA	NA	88	88
NA	NA	NA	NA	NA	NA	NA	88	88
NA	NA	NA	NA	NA	NA	NA	88	88
NA	NA	NA	NA	NA	NA	NA	88	88
NA	NA	NA	NA	NA	NA	NA	88	88
NA	NA	NA	NA	NA	NA	NA	2	88
NA	NA	NA	NA	NA	NA	NA	88	88
NA	NA	NA	NA	NA	NA	NA	88	88
NA	NA	NA	NA	NA	NA	NA	88	88
NA	NA	NA	NA	NA	NA	NA	88	88
NA	NA	NA	NA	NA	NA	NA	88	88
NA	NA	NA	NA	NA	NA	NA	1	88
NA	NA	NA	NA	NA	NA	NA	88	88
NA	NA	NA	YET TO GIVE MEDICINE	NA	NA	NA	88	88
NA	NA	NA	NA	NA	NA	NA	88	88
NA	NA	NA	NA	NA	NA	NA	88	88
NA	NA	NA	NA	NA	NA	NA	88	88
NA	NA	NA	NA	NA	NA	NA	88	88
NA	NA	NA	NA	NA	NA	NA	88	88
NA	NA	NA	NA	NA	NA	NA	88	88
NA	NA	NA	NA	NA	NA	NA	88	88
NA	NA	NA	NA	NA	NA	NA	88	88
NA	NA	NA	NA	NA	NA	NA	88	88
NA	NA	NA	NA	NA	NA	NA	88	88
NA	NA	NA	NA	NA	NA	NA	88	88
NA	NA	NA	NA	NA	NA	NA	88	88
NA	NA	NA	NA	NA	NA	NA	88	88
NA	NA	NA	CHILD REFEWS TO TAKE	NA	NA	NA	88	88
NA	NA	NA	NA	NA	NA	NA	88	88
NA	NA	NA	NA	NA	NA	NA	88	88
NA	NA	NA	NA	NA	NA	NA	88	88

NA	NA	NA	NA	NA	NA	NA	88	88
NA	NA	NA	NA	NA	NA	NA	88	88
NA	NA	NA	NA	NA	NA	NA	88	88
NA	NA	NA	NA	NA	NA	NA	88	88
NA	NA	NA	NA	NA	NA	NA	88	88
NA	NA	NA	NA	NA	NA	NA	88	88
NA	NA	NA	NA	NA	NA	NA	88	88
NA	NA	NA	NA	NA	NA	NA	88	88
NA	NA	NA	NA	NA	NA	NA	88	88
NA	NA	NA	NA	NA	NA	NA	88	88
NA	NA	NA	NA	NA	NA	NA	88	88
NA	NA	NA	NA	NA	NA	NA	88	88
NA	NA	NA	NA	NA	NA	NA	1	88
NA	NA	NA	NA	NA	NA	NA	88	88
NA	NA	NA	NA	NA	NA	NA	88	88
NA	NA	NA	NA	NA	NA	NA	88	88
NA	NA	NA	NA	NA	NA	NA	88	88
NA	NA	NA	NA	NA	NA	NA	88	88
NA	NA	NA	NA	NA	NA	NA	88	88
NA	NA	NA	NA	NA	NA	NA	88	88
NA	NA	NA	NA	NA	NA	NA	88	88
NA	NA	NA	NA	NA	NA	NA	88	88
NA	NA	NA	NA	NA	NA	NA	88	88
NA	NA	NA	NA	NA	NA	NA	88	88
NA	NA	NA	NA	NA	NA	NA	88	88
NA	NA	NA	NA	NA	NA	NA	2	88
NA	NA	NA	NA	NA	NA	NA	88	88
NA	NA	NA	NA	NA	NA	NA	88	88
NA	NA	NA	NA	NA	NA	NA	88	88
NA	NA	NA	NA	NA	NA	NA	88	88
NA	NA	NA	NA	NA	NA	NA	88	88
NA	NA	NA	NA	NA	NA	NA	88	88
NA	NA	NA	NA	NA	NA	NA	88	88
NA	NA	NA	NA	NA	NA	NA	88	88
NA	NA	NA	NA	NA	NA	NA	88	88
NA	NA	NA	NA	NA	NA	NA	88	88
NA	NA	NA	NA	NA	NA	NA	88	88
NA	NA	NA	NA	NA	NA	NA	2	88
NA	NA	NA	NA	NA	NA	NA	88	88
NA	NA	NA	NA	NA	NA	NA	88	88
NA	NA	NA	NA	NA	NA	NA	88	88
NA	NA	NA	NA	NA	NA	NA	88	88
NA	NA	NA	NA	NA	NA	NA	88	88
NA	NA	NA	NA	NA	NA	NA	1	88
NA	NA	NA	NA	NA	NA	NA	88	88
NA	NA	NA	NA	NA	NA	NA	88	88
NA	NA	NA	NA	NA	NA	NA	88	88

NA	NA	NA	NA	NA	NA	NA	88	88
NA	NA	NA	NA	NA	NA	NA	88	88
NA	NA	NA	NA	NA	NA	NA	88	88
NA	NA	NA	NA	NA	NA	NA	88	88
NA	NA	NA	NA	NA	NA	NA	88	88
NA	NA	NA	NA	NA	NA	NA	88	88
NA	NA	NA	WILL CONTINUE TREATM	NA	NA	NA	88	88
NA	NA	NA	NA	NA	NA	NA	88	88
NA	NA	NA	WILL CONTINUE TREATM	NA	NA	NA	88	88
NA	NA	NA	NA	NA	NA	NA	3	88
NA	NA	NA	NA	NA	NA	NA	88	88
NA	NA	NA	NA	NA	NA	NA	1	88
NA	NA	NA	NA	NA	NA	NA	88	88
NA	NA	NA	NA	NA	NA	NA	88	88
NA	NA	NA	NA	NA	NA	NA	88	88
NA	NA	NA	NA	NA	NA	NA	88	88
NA	NA	NA	NA	NA	NA	NA	88	88
NA	NA	NA	WILL CONTINUE TREATM	NA	NA	NA	88	88
NA	NA	NA	NA	NA	NA	NA	88	88
NA	NA	NA	NA	NA	NA	NA	88	88
NA	NA	NA	NA	NA	NA	NA	88	88
NA	NA	NA	NA	NA	NA	NA	88	88
NA	NA	NA	NA	NA	NA	NA	88	88
NA	NA	NA	NA	NA	NA	NA	88	88
NA	NA	NA	NA	NA	NA	NA	1	88
NA	NA	NA	WILL CONTINUE TREATM	NA	NA	NA	88	88
NA	NA	NA	NA	NA	NA	NA	88	88
NA	NA	NA	NA	NA	NA	NA	88	88
NA	NA	NA	YET TO GIVE MEDICINE	NA	NA	NA	88	88
NA	NA	NA	NA	NA	NA	NA	88	88
NA	NA	NA	NA	NA	NA	NA	88	88
NA	NA	NA	NA	NA	NA	NA	88	88
NA	NA	NA	NA	NA	NA	NA	2	88
NA	NA	NA	NA	NA	NA	NA	88	88
NA	NA	NA	NA	NA	NA	NA	88	88
NA	NA	NA	NA	NA	NA	NA	1	88
NA	NA	NA	NA	NA	NA	NA	2	88
NA	NA	NA	NA	NA	NA	NA	88	88
NA	NA	NA	NA	NA	NA	NA	88	88
NA	NA	NA	NA	NA	NA	NA	1	88
NA	NA	NA	NA	NA	NA	NA	88	88
NA	NA	NA	NA	NA	NA	NA	1	88
NA	NA	NA	NA	NA	NA	NA	2	88
NA	NA	NA	NA	NA	NA	NA	88	88
NA	NA	NA	NA	NA	NA	NA	88	88
NA	NA	NA	NA	NA	NA	NA	88	88
NA	NA	NA	NA	NA	NA	NA	88	88

NA	NA	NA	NA	NA	NA	NA	88	88
NA	NA	NA	NA	NA	NA	NA	88	88
NA	NA	NA	NA	NA	NA	NA	88	88
NA	NA	NA	NA	NA	NA	NA	88	88
NA	NA	NA	NA	NA	NA	NA	88	88
NA	NA	NA	NA	NA	NA	NA	88	88
NA	NA	NA	NA	NA	NA	NA	88	88
NA	NA	NA	NA	NA	NA	NA	88	88
NA	NA	NA	NA	NA	NA	NA	88	88
NA	NA	NA	NA	NA	NA	NA	88	88
NA	NA	NA	WILL CONTINUE TREATM	NA	NA	NA	88	88
NA	NA	NA	NA	NA	NA	NA	88	88
NA	NA	NA	NA	NA	NA	NA	88	88
NA	NA	NA	NA	NA	NA	NA	88	88
NA	NA	NA	NA	NA	NA	NA	88	88
NA	NA	NA	NA	NA	NA	NA	88	88
NA	NA	NA	NA	NA	NA	NA	88	88
NA	NA	NA	NA	NA	NA	NA	88	88

q58h2	q58h3	q59t	q59t1	q59t2	q59t3	q60t	q60t1	q60t2	q60t3	q61t	q61t1	q61t2	q61t3	q62v	q62v1
88	88	88	88	88	88	88	88	88	88	88	88	88	88	88	88
88	88	88	88	88	88	88	88	88	88	88	88	88	88	88	88
88	88	88	88	88	88	88	88	88	88	88	88	88	88	88	88
88	88	88	88	88	88	88	88	88	88	88	88	88	88	88	88
88	88	88	88	88	88	88	88	88	88	88	88	88	88	88	88
88	88	88	88	88	88	88	88	88	88	88	88	88	88	88	88
88	88	88	88	88	88	88	88	88	88	88	88	88	88	88	88
88	88	Mo	88	88	88	Ye	88	88	88	No	88	88	88	No	88
88	88	88	88	88	88	88	88	88	88	88	88	88	88	88	88
88	88	88	88	88	88	88	88	88	88	88	88	88	88	88	88
88	88	Ev	88	88	88	Ye	88	88	88	No	88	88	88	No	88
88	88	88	88	88	88	88	88	88	88	88	88	88	88	88	88
88	88	Mo	88	88	88	Ye	88	88	88	No	88	88	88	No	88
88	88	88	88	88	88	88	88	88	88	88	88	88	88	88	88
88	88	88	88	88	88	88	88	88	88	88	88	88	88	88	88
88	88	88	88	88	88	88	88	88	88	88	88	88	88	88	88
88	88	88	88	88	88	88	88	88	88	88	88	88	88	88	88
88	88	88	88	88	88	88	88	88	88	88	88	88	88	88	88
88	88	Mo	88	88	88	Ye	88	88	88	No	88	88	88	No	88
88	88	88	88	88	88	88	88	88	88	88	88	88	88	88	88
88	88	88	88	88	88	88	88	88	88	88	88	88	88	88	88
88	88	Mo	88	88	88	Ye	88	88	88	No	88	88	88	No	88
88	88	88	88	88	88	88	88	88	88	88	88	88	88	88	88
88	88	88	88	88	88	88	88	88	88	88	88	88	88	88	88
88	88	88	88	88	88	88	88	88	88	88	88	88	88	88	88
88	88	88	88	88	88	88	88	88	88	88	88	88	88	88	88
88	88	88	88	88	88	88	88	88	88	88	88	88	88	88	88
88	88	88	88	88	88	88	88	88	88	88	88	88	88	88	88
88	88	Mo	88	88	88	Ye	88	88	88	No	88	88	88	No	88
88	88	88	88	88	88	88	88	88	88	88	88	88	88	88	88
88	88	88	88	88	88	88	88	88	88	88	88	88	88	88	88
88	88	88	88	88	88	88	88	88	88	88	88	88	88	88	88
88	88	88	88	88	88	88	88	88	88	88	88	88	88	88	88
88	88	88	88	88	88	88	88	88	88	88	88	88	88	88	88
88	88	88	88	88	88	88	88	88	88	88	88	88	88	88	88
88	88	Mo	88	88	88	Ye	88	88	88	No	88	88	88	No	88
88	88	88	88	88	88	88	88	88	88	88	88	88	88	88	88
88	88	Mo	88	88	88	Ye	88	88	88	No	88	88	88	No	88
88	88	88	88	88	88	88	88	88	88	88	88	88	88	88	88
88	88	88	88	88	88	88	88	88	88	88	88	88	88	88	88
88	88	88	88	88	88	88	88	88	88	88	88	88	88	88	88
88	88	88	88	88	88	88	88	88	88	88	88	88	88	88	88
88	88	88	88	88	88	88	88	88	88	88	88	88	88	88	88
88	88	88	88	88	88	88	88	88	88	88	88	88	88	88	88
88	88	88	88	88	88	88	88	88	88	88	88	88	88	88	88
88	88	Mo	88	88	88	Ye	88	88	88	No	88	88	88	No	88

88	88	88	88	88	88	88	88	88	88	88	88	88	88	88	88
88	88	88	88	88	88	88	88	88	88	88	88	88	88	88	88
88	88	88	88	88	88	88	88	88	88	88	88	88	88	88	88
88	88	Mo	88	88	88	No	88	88	88	No	88	88	88	No	88
88	88	88	88	88	88	88	88	88	88	88	88	88	88	88	88
88	88	88	88	88	88	88	88	88	88	88	88	88	88	88	88
88	88	88	88	88	88	88	88	88	88	88	88	88	88	88	88
88	88	88	88	88	88	88	88	88	88	88	88	88	88	88	88
88	88	88	88	88	88	88	88	88	88	88	88	88	88	88	88
88	88	88	88	88	88	88	88	88	88	88	88	88	88	88	88
88	88	88	88	88	88	88	88	88	88	88	88	88	88	88	88
88	88	88	88	88	88	88	88	88	88	88	88	88	88	88	88
88	88	88	88	88	88	88	88	88	88	88	88	88	88	88	88
88	88	88	88	88	88	88	88	88	88	88	88	88	88	88	88
88	88	88	88	88	88	88	88	88	88	88	88	88	88	88	88
88	88	88	88	88	88	88	88	88	88	88	88	88	88	88	88
88	88	88	88	88	88	88	88	88	88	88	88	88	88	88	88
88	88	88	88	88	88	88	88	88	88	88	88	88	88	88	88
88	88	88	88	88	88	88	88	88	88	88	88	88	88	88	88
88	88	88	88	88	88	88	88	88	88	88	88	88	88	88	88
88	88	88	88	88	88	88	88	88	88	88	88	88	88	88	88
88	88	88	88	88	88	88	88	88	88	88	88	88	88	88	88
88	88	88	88	88	88	88	88	88	88	88	88	88	88	88	88
88	88	88	88	88	88	88	88	88	88	88	88	88	88	88	88
88	88	88	88	88	88	88	88	88	88	88	88	88	88	88	88
88	88	88	88	88	88	88	88	88	88	88	88	88	88	88	88
88	88	88	88	88	88	88	88	88	88	88	88	88	88	88	88
88	88	88	88	88	88	88	88	88	88	88	88	88	88	88	88
88	88	88	88	88	88	88	88	88	88	88	88	88	88	88	88
88	88	88	88	88	88	88	88	88	88	88	88	88	88	88	88
88	88	88	88	88	88	88	88	88	88	88	88	88	88	88	88
88	88	88	88	88	88	88	88	88	88	88	88	88	88	88	88
88	88	88	88	88	88	88	88	88	88	88	88	88	88	88	88
88	88	88	88	88	88	88	88	88	88	88	88	88	88	88	88
88	88	88	88	88	88	88	88	88	88	88	88	88	88	88	88
88	88	88	88	88	88	88	88	88	88	88	88	88	88	88	88
88	88	88	88	88	88	88	88	88	88	88	88	88	88	88	88
88	88	88	88	88	88	88	88	88	88	88	88	88	88	88	88
88	88	88	88	88	88	88	88	88	88	88	88	88	88	88	88
88	88	88	88	88	88	88	88	88	88	88	88	88	88	88	88
88	88	88	88	88	88	88	88	88	88	88	88	88	88	88	88
88	88	88	88	88	88	88	88	88	88	88	88	88	88	88	88
88	88	88	88	88	88	88	88	88	88	88	88	88	88	88	88
88	88	88	88	88	88	88	88	88	88	88	88	88	88	88	88
88	88	88	88	88	88	88	88	88	88	88	88	88	88	88	88
88	88	88	88	88	88	88	88	88	88	88	88	88	88	88	88
88	88	88	88	88	88	88	88	88	88	88	88	88	88	88	88

88	88	88	88	88	88	88	88	88	88	88	88	88	88	88	88
88	88	Mo	88	88	88	Ye	88	88	88	No	88	88	88	No	88
88	88	88	88	88	88	88	88	88	88	88	88	88	88	88	88
88	88	88	88	88	88	88	88	88	88	88	88	88	88	88	88
88	88	88	88	88	88	88	88	88	88	88	88	88	88	88	88
88	88	88	88	88	88	88	88	88	88	88	88	88	88	88	88
88	88	88	88	88	88	88	88	88	88	88	88	88	88	88	88
88	88	88	88	88	88	88	88	88	88	88	88	88	88	88	88
88	88	88	88	88	88	88	88	88	88	88	88	88	88	88	88
88	88	88	88	88	88	88	88	88	88	88	88	88	88	88	88
88	88	88	88	88	88	88	88	88	88	88	88	88	88	88	88
88	88	88	88	88	88	88	88	88	88	88	88	88	88	88	88
88	88	88	88	88	88	88	88	88	88	88	88	88	88	88	88
88	88	Mo	88	88	88	Ye	88	88	88	No	88	88	88	No	88
88	88	88	88	88	88	88	88	88	88	88	88	88	88	88	88
88	88	88	88	88	88	88	88	88	88	88	88	88	88	88	88
88	88	88	88	88	88	88	88	88	88	88	88	88	88	88	88
88	88	88	88	88	88	88	88	88	88	88	88	88	88	88	88
88	88	88	88	88	88	88	88	88	88	88	88	88	88	88	88
88	88	88	88	88	88	88	88	88	88	88	88	88	88	88	88
88	88	88	88	88	88	88	88	88	88	88	88	88	88	88	88
88	88	88	88	88	88	88	88	88	88	88	88	88	88	88	88
88	88	88	88	88	88	88	88	88	88	88	88	88	88	88	88
88	88	Mo	88	88	88	Ye	88	88	88	No	88	88	88	88	88
88	88	88	88	88	88	88	88	88	88	88	88	88	88	88	88
88	88	88	88	88	88	88	88	88	88	88	88	88	88	88	88
88	88	88	88	88	88	88	88	88	88	88	88	88	88	88	88
88	88	88	88	88	88	88	88	88	88	88	88	88	88	88	88
88	88	88	88	88	88	88	88	88	88	88	88	88	88	88	88
88	88	88	88	88	88	88	88	88	88	88	88	88	88	88	88
88	88	Mo	88	88	88	Ye	88	88	88	No	88	88	88	No	88
88	88	88	88	88	88	88	88	88	88	88	88	88	88	88	88
88	88	Mo	88	88	88	Ye	88	88	88	No	88	88	88	No	88
88	88	Mo	88	88	88	Ye	88	88	88	No	88	88	88	No	88
88	88	88	88	88	88	88	88	88	88	88	88	88	88	88	88
88	88	88	88	88	88	88	88	88	88	88	88	88	88	88	88
88	88	88	88	88	88	88	88	88	88	88	88	88	88	88	88
88	88	88	88	88	88	88	88	88	88	88	88	88	88	88	88
88	88	88	88	88	88	88	88	88	88	88	88	88	88	88	88
88	88	88	88	88	88	88	88	88	88	88	88	88	88	88	88
88	88	88	88	88	88	88	88	88	88	88	88	88	88	88	88
88	88	88	88	88	88	88	88	88	88	88	88	88	88	88	88
88	88	88	88	88	88	88	88	88	88	88	88	88	88	88	88
88	88	88	88	88	88	88	88	88	88	88	88	88	88	88	88
88	88	88	88	88	88	88	88	88	88	88	88	88	88	88	88
88	88	88	88	88	88	88	88	88	88	88	88	88	88	88	88
88	88	88	88	88	88	88	88	88	88	88	88	88	88	88	88

88	88	88	88	88	88	88	88	88	88	88	88	88	88	88	88
88	88	88	88	88	88	88	88	88	88	88	88	88	88	88	88
88	88	88	88	88	88	88	88	88	88	88	88	88	88	88	88
88	88	Mo	88	88	88	Ye	88	88	88	No	88	88	88	No	88
88	88	88	88	88	88	88	88	88	88	88	88	88	88	88	88
88	88	88	88	88	88	88	88	88	88	88	88	88	88	88	88
88	88	88	88	88	88	88	88	88	88	88	88	88	88	88	88
88	88	88	88	88	88	88	88	88	88	88	88	88	88	88	88
88	88	88	88	88	88	88	88	88	88	88	88	88	88	88	88
88	88	88	88	88	88	88	88	88	88	88	88	88	88	88	88
88	88	88	88	88	88	88	88	88	88	88	88	88	88	88	88
88	88	88	88	88	88	88	88	88	88	88	88	88	88	88	88
88	88	88	88	88	88	88	88	88	88	88	88	88	88	88	88
88	88	88	88	88	88	88	88	88	88	88	88	88	88	88	88
88	88	88	88	88	88	88	88	88	88	88	88	88	88	88	88
88	88	Mo	88	88	88	Ye	88	88	88	No	88	88	88	No	88
88	88	88	88	88	88	88	88	88	88	88	88	88	88	88	88
88	88	88	88	88	88	88	88	88	88	88	88	88	88	88	88
88	88	88	88	88	88	88	88	88	88	88	88	88	88	88	88
88	88	88	88	88	88	88	88	88	88	88	88	88	88	88	88
88	88	88	88	88	88	88	88	88	88	88	88	88	88	88	88
88	88	Mo	88	88	88	Ye	88	88	88	No	88	88	88	No	88
88	88	88	88	88	88	88	88	88	88	88	88	88	88	88	88
88	88	88	88	88	88	88	88	88	88	88	88	88	88	88	88
88	88	88	88	88	88	88	88	88	88	88	88	88	88	88	88
88	88	88	88	88	88	88	88	88	88	88	88	88	88	88	88
88	88	88	88	88	88	88	88	88	88	88	88	88	88	88	88
88	88	Mo	88	88	88	Ye	88	88	88	No	88	88	88	No	88
88	88	88	88	88	88	88	88	88	88	88	88	88	88	88	88
88	88	88	88	88	88	88	88	88	88	88	88	88	88	88	88
88	88	88	88	88	88	88	88	88	88	88	88	88	88	88	88
88	88	88	88	88	88	88	88	88	88	88	88	88	88	88	88
88	88	88	88	88	88	88	88	88	88	88	88	88	88	88	88
88	88	88	88	88	88	88	88	88	88	88	88	88	88	88	88
88	88	88	88	88	88	88	88	88	88	88	88	88	88	88	88
88	88	88	88	88	88	88	88	88	88	88	88	88	88	88	88
88	88	88	88	88	88	88	88	88	88	88	88	88	88	88	88
88	88	88	88	88	88	88	88	88	88	88	88	88	88	88	88
88	88	88	88	88	88	88	88	88	88	88	88	88	88	88	88
88	88	88	88	88	88	88	88	88	88	88	88	88	88	88	88
88	88	88	88	88	88	88	88	88	88	88	88	88	88	88	88
88	88	88	88	88	88	88	88	88	88	88	88	88	88	88	88
88	88	88	88	88	88	88	88	88	88	88	88	88	88	88	88
88	88	88	88	88	88	88	88	88	88	88	88	88	88	88	88
88	88	88	88	88	88	88	88	88	88	88	88	88	88	88	88
88	88	88	88	88	88	88	88	88	88	88	88	88	88	88	88
88	88	88	88	88	88	88	88	88	88	88	88	88	88	88	88

88	88	88	88	88	88	88	88	88	88	88	88	88	88	88	88
88	88	88	88	88	88	88	88	88	88	88	88	88	88	88	88
88	88	88	88	88	88	88	88	88	88	88	88	88	88	88	88
88	88	88	88	88	88	88	88	88	88	88	88	88	88	88	88
88	88	88	88	88	88	88	88	88	88	88	88	88	88	88	88
88	88	88	88	88	88	88	88	88	88	88	88	88	88	88	88
88	88	88	88	88	88	88	88	88	88	88	88	88	88	88	88
88	88	88	88	88	88	88	88	88	88	88	88	88	88	88	88
88	88	88	88	88	88	88	88	88	88	88	88	88	88	88	88
88	88	88	88	88	88	88	88	88	88	88	88	88	88	88	88
88	88	88	88	88	88	88	88	88	88	88	88	88	88	88	88
88	88	88	88	88	88	88	88	88	88	88	88	88	88	88	88
88	88	Mo	88	88	88	Ye	88	88	88	No	88	88	88	No	88
88	88	88	88	88	88	88	88	88	88	88	88	88	88	88	88
88	88	88	88	88	88	88	88	88	88	88	88	88	88	88	88
88	88	88	88	88	88	88	88	88	88	88	88	88	88	88	88
88	88	88	88	88	88	88	88	88	88	88	88	88	88	88	88
88	88	88	88	88	88	88	88	88	88	88	88	88	88	88	88
88	88	88	88	88	88	88	88	88	88	88	88	88	88	88	88
88	88	88	88	88	88	88	88	88	88	88	88	88	88	88	88
88	88	88	88	88	88	88	88	88	88	88	88	88	88	88	88
88	88	88	88	88	88	88	88	88	88	88	88	88	88	88	88
88	88	88	88	88	88	88	88	88	88	88	88	88	88	88	88
88	88	88	88	88	88	88	88	88	88	88	88	88	88	88	88
88	88	88	88	88	88	88	88	88	88	88	88	88	88	88	88
88	88	Mo	88	88	88	Ye	88	88	88	No	88	88	88	No	88
88	88	88	88	88	88	88	88	88	88	88	88	88	88	88	88
88	88	88	88	88	88	88	88	88	88	88	88	88	88	88	88
88	88	88	88	88	88	88	88	88	88	88	88	88	88	88	88
88	88	88	88	88	88	88	88	88	88	88	88	88	88	88	88
88	88	88	88	88	88	88	88	88	88	88	88	88	88	88	88
88	88	88	88	88	88	88	88	88	88	88	88	88	88	88	88
88	88	88	88	88	88	88	88	88	88	88	88	88	88	88	88
88	88	88	88	88	88	88	88	88	88	88	88	88	88	88	88
88	88	88	88	88	88	88	88	88	88	88	88	88	88	88	88
88	88	88	88	88	88	88	88	88	88	88	88	88	88	88	88
88	88	88	88	88	88	88	88	88	88	88	88	88	88	88	88
88	88	Mo	88	88	88	Ye	88	88	88	Ye	88	88	88	No	88
88	88	88	88	88	88	88	88	88	88	88	88	88	88	88	88
88	88	88	88	88	88	88	88	88	88	88	88	88	88	88	88
88	88	88	88	88	88	88	88	88	88	88	88	88	88	88	88
88	88	88	88	88	88	88	88	88	88	88	88	88	88	88	88
88	88	88	88	88	88	88	88	88	88	88	88	88	88	88	88
88	88	Mo	88	88	88	Ye	88	88	88	No	88	88	88	No	88
88	88	88	88	88	88	88	88	88	88	88	88	88	88	88	88
88	88	88	88	88	88	88	88	88	88	88	88	88	88	88	88
88	88	88	88	88	88	88	88	88	88	88	88	88	88	88	88

88	88	88	88	88	88	88	88	88	88	88	88	88	88	88	88
88	88	88	88	88	88	88	88	88	88	88	88	88	88	88	88
88	88	88	88	88	88	88	88	88	88	88	88	88	88	88	88
88	88	88	88	88	88	88	88	88	88	88	88	88	88	88	88
88	88	88	88	88	88	88	88	88	88	88	88	88	88	88	88
88	88	88	88	88	88	88	88	88	88	88	88	88	88	88	88
88	88	88	88	88	88	88	88	88	88	88	88	88	88	88	88
88	88	88	88	88	88	88	88	88	88	88	88	88	88	88	88
88	88	88	88	88	88	88	88	88	88	88	88	88	88	88	88
88	88	Mo	88	88	88	Ye	88	88	88	No	88	88	88	No	88
88	88	88	88	88	88	88	88	88	88	88	88	88	88	88	88
88	88	Mo	88	88	88	Ye	88	88	88	No	88	88	88	No	88
88	88	88	88	88	88	88	88	88	88	88	88	88	88	88	88
88	88	88	88	88	88	88	88	88	88	88	88	88	88	88	88
88	88	88	88	88	88	88	88	88	88	88	88	88	88	88	88
88	88	88	88	88	88	88	88	88	88	88	88	88	88	88	88
88	88	88	88	88	88	88	88	88	88	88	88	88	88	88	88
88	88	88	88	88	88	88	88	88	88	88	88	88	88	88	88
88	88	88	88	88	88	88	88	88	88	88	88	88	88	88	88
88	88	88	88	88	88	88	88	88	88	88	88	88	88	88	88
88	88	88	88	88	88	88	88	88	88	88	88	88	88	88	88
88	88	88	88	88	88	88	88	88	88	88	88	88	88	88	88
88	88	88	88	88	88	88	88	88	88	88	88	88	88	88	88
88	88	88	88	88	88	88	88	88	88	88	88	88	88	88	88
88	88	Mo	88	88	88	Ye	88	88	88	No	88	88	88	No	88
88	88	88	88	88	88	88	88	88	88	88	88	88	88	88	88
88	88	88	88	88	88	88	88	88	88	88	88	88	88	88	88
88	88	88	88	88	88	88	88	88	88	88	88	88	88	88	88
88	88	88	88	88	88	88	88	88	88	88	88	88	88	88	88
88	88	88	88	88	88	88	88	88	88	88	88	88	88	88	88
88	88	88	88	88	88	88	88	88	88	88	88	88	88	88	88
88	88	88	88	88	88	88	88	88	88	88	88	88	88	88	88
88	88	Mo	88	88	88	Ye	88	88	88	No	88	88	88	No	88
88	88	88	88	88	88	88	88	88	88	88	88	88	88	88	88
88	88	88	88	88	88	88	88	88	88	88	88	88	88	88	88
88	88	Mo	88	88	88	Ye	88	88	88	No	88	88	88	No	88
88	88	Mo	88	88	88	Ye	88	88	88	No	88	88	88	No	88
88	88	88	88	88	88	88	88	88	88	88	88	88	88	88	88
88	88	88	88	88	88	88	88	88	88	88	88	88	88	88	88
88	88	Mo	88	88	88	Ye	88	88	88	No	88	88	88	No	88
88	88	88	88	88	88	88	88	88	88	88	88	88	88	88	88
88	88	Mo	88	88	88	Ye	88	88	88	No	88	88	88	No	88
88	88	Mo	88	88	88	Ye	88	88	88	No	88	88	88	No	88
88	88	88	88	88	88	88	88	88	88	88	88	88	88	88	88
88	88	88	88	88	88	88	88	88	88	88	88	88	88	88	88
88	88	88	88	88	88	88	88	88	88	88	88	88	88	88	88
88	88	88	88	88	88	88	88	88	88	88	88	88	88	88	88

88	88	88	88	88	88	88	88	88	88	88	88	88	88	88	88
88	88	88	88	88	88	88	88	88	88	88	88	88	88	88	88
88	88	88	88	88	88	88	88	88	88	88	88	88	88	88	88
88	88	88	88	88	88	88	88	88	88	88	88	88	88	88	88
88	88	88	88	88	88	88	88	88	88	88	88	88	88	88	88
88	88	88	88	88	88	88	88	88	88	88	88	88	88	88	88
88	88	88	88	88	88	88	88	88	88	88	88	88	88	88	88
88	88	88	88	88	88	88	88	88	88	88	88	88	88	88	88
88	88	88	88	88	88	88	88	88	88	88	88	88	88	88	88
88	88	88	88	88	88	88	88	88	88	88	88	88	88	88	88
88	88	88	88	88	88	88	88	88	88	88	88	88	88	88	88
88	88	88	88	88	88	88	88	88	88	88	88	88	88	88	88
88	88	88	88	88	88	88	88	88	88	88	88	88	88	88	88
88	88	88	88	88	88	88	88	88	88	88	88	88	88	88	88
88	88	88	88	88	88	88	88	88	88	88	88	88	88	88	88
88	88	88	88	88	88	88	88	88	88	88	88	88	88	88	88
88	88	88	88	88	88	88	88	88	88	88	88	88	88	88	88
88	88	88	88	88	88	88	88	88	88	88	88	88	88	88	88

q62v2	q62v3	q63i	q63i1	q63i2	q63i3	q64i	q65w	q65w1	q65w2	q65w3	q66	q67	q67a	q68	q69	q70
88	88	88	88	88	88	88	NA	NA	NA	NA	Y	Pa	NA	Ye	6	Ye
88	88	88	88	88	88	88	Ot	NA	NA	NA	Y	Ot	NA	Ye	6	Ye
88	88	88	88	88	88	88	NA	NA	NA	NA	N		NA	Ye	9	Ye
88	88	88	88	88	88	88	NA	NA	NA	NA	N		NA	Ye	9	Ye
88	88	88	88	88	88	88	NA	NA	NA	NA	Y	Pa	NA	Ye	6	Ye
88	88	88	88	88	88	88	NA	NA	NA	NA	Y	Pa	NA	Ye	6	Ye
88	88	88	88	88	88	88	NA	NA	NA	NA	Y	Pa	NA	Ye	6	Ye
88	88	88	88	88	88	88	Fa	NA	NA	NA	Y	Pa	NA	Ye	6	Ye
88	88	88	88	88	88	88	NA	NA	NA	NA	Y	Pa	NA	Ye	6	Ye
88	88	88	88	88	88	88	NA	NA	NA	NA	Y	Pa	NA	Ye	6	Ye
88	88	88	88	88	88	88	NA	NA	NA	NA	N	99	NA	Ye	9	Ye
88	88	88	88	88	88	88	Mo	NA	NA	NA	Y	Pa	NA	Ye	6	Ye
88	88	88	88	88	88	88	Fa	NA	NA	NA	Y	Pa	NA	Ye	6	Ye
88	88	88	88	88	88	88	NA	NA	NA	NA	Y	Pa	NA	Ye	6	Ye
88	88	88	88	88	88	88	NA	NA	NA	NA	Y	Pa	NA	Ye	6	Ye
88	88	88	88	88	88	88	NA	NA	NA	NA	N		NA	Ye	9	Ye
88	88	88	88	88	88	88	NA	NA	NA	NA	Y	Pa	NA	Ye	9	Ye
88	88	88	88	88	88	88	NA	NA	NA	NA	N		NA	Ye	9	Ye
88	88	88	88	88	88	88	NA	NA	NA	NA	N		NA	Ye	9	Ye
88	88	88	88	88	88	88	NA	NA	NA	NA	Y	Pa	NA	Ye	6	Ye
88	88	88	88	88	88	88	NA	NA	NA	NA	Y	Pa	NA	Ye	6	Ye
88	88	88	88	88	88	88	Mo	NA	NA	NA	Y	Pa	NA	Ye	6	Ye
88	88	88	88	88	88	88	NA	NA	NA	NA	Y	Pa	NA	Ye	6	Ye
88	88	88	88	88	88	88	NA	NA	NA	NA	N		NA	Ye	9	Ye
88	88	88	88	88	88	88	NA	NA	NA	NA	Y	Pa	NA	Ye	9	Ye
88	88	88	88	88	88	88	NA	NA	NA	NA	Y	Pa	NA	Ye	6	Ye
88	88	88	88	88	88	88	NA	NA	NA	NA	Y	Pa	NA	Ye	3	Ye
88	88	88	88	88	88	88	NA	NA	NA	NA	Y	Pa	NA	Ye	6	Ye
88	88	88	88	88	88	88	NA	NA	NA	NA	Y	Pa	NA	Ye	9	Ye
88	88	88	88	88	88	88	NA	NA	NA	NA	Y	Pa	NA	Ye	3	Ye
88	88	88	88	88	88	88	NA	NA	NA	NA	Y	Ot	NA	Ye	6	Ye
88	88	88	88	88	88	88	NA	NA	NA	NA	Y	Pa	NA	Ye	6	Ye
88	88	88	88	88	88	88	NA	NA	NA	NA	Y	Pa	NA	Ye	3	Ye
88	88	88	88	88	88	88	NA	NA	NA	NA	N		NA	Ye	9	Ye
88	88	88	88	88	88	88	NA	NA	NA	NA	N		NA	Ye	9	Ye
88	88	88	88	88	88	88	NA	NA	NA	NA	N		NA	Ye	9	Ye
88	88	88	88	88	88	88	NA	NA	NA	NA	N		NA	Ye	9	Ye
88	88	88	88	88	88	88	Mo	NA	NA	NA	Y	Pa	NA	Ye	6	Ye
88	88	88	88	88	88	88	NA	NA	NA	NA	Y	Pa	NA	Ye	6	Ye
88	88	88	88	88	88	88	NA	NA	NA	NA	Y	Ot	NA	Ye	6	Ye
88	88	88	88	88	88	88	NA	NA	NA	NA	Y	Ot	NA	Ye	6	Ye
88	88	88	88	88	88	88	NA	NA	NA	NA	Y	Pa	NA	Ye	6	Ye
88	88	88	88	88	88	88	NA	NA	NA	NA	Y	Pa	NA	Ye	6	Ye
88	88	88	88	88	88	88	NA	NA	NA	NA	Y	Ot	NA	Ye	6	Ye
88	88	88	88	88	88	88	NA	NA	NA	NA	Y	Pa	NA	Ye	3	Ye
88	88	88	88	88	88	88	Ot	NA	NA	NA	Y	Ot	NA	Ye	6	Ye

88	88	88	88	88	88	88	NA	NA	NA	NA	Y	Ot	NA	Ye	6	Ye
88	88	88	88	88	88	88	NA	NA	NA	NA	Y	Pa	NA	Ye	3	Ye
88	88	88	88	88	88	88	NA	NA	NA	NA	N		NA	Ye	9	Ye
88	88	88	88	88	88	88	Mo	NA	NA	NA	Y	Pa	NA	Ye	6	Ye
88	88	88	88	88	88	88	NA	NA	NA	NA	Y	Pa	NA	Ye	6	Ye
88	88	88	88	88	88	88	NA	NA	NA	NA	Y	Pa	NA	Ye	9	Ye
88	88	88	88	88	88	88	NA	NA	NA	NA	Y	Pa	NA	Ye	6	Ye
88	88	88	88	88	88	88	NA	NA	NA	NA	Y	Pa	NA	Ye	6	Ye
88	88	88	88	88	88	88	NA	NA	NA	NA	N		NA	Ye	9	Ye
88	88	88	88	88	88	88	NA	NA	NA	NA	N		NA	Ye	9	Ye
88	88	88	88	88	88	88	NA	NA	NA	NA	Y	Ot	NA	Ye	6	Ye
88	88	88	88	88	88	88	NA	NA	NA	NA	Y	Pa	NA	Ye	6	Ye
88	88	88	88	88	88	88	NA	NA	NA	NA	Y	Ot	NA	Ye	6	Ye
88	88	88	88	88	88	88	NA	NA	NA	NA	Y	Pa	NA	Ye	6	Ye
88	88	88	88	88	88	88	NA	NA	NA	NA	N		NA	Ye	9	Ye
88	88	88	88	88	88	88	NA	NA	NA	NA	Y	Ot	NA	Ye	6	Ye
88	88	88	88	88	88	88	NA	NA	NA	NA	Y	Pa	NA	Ye	6	Ye
88	88	88	88	88	88	88	NA	NA	NA	NA	Y	Pa	NA	Ye	6	Ye
88	88	88	88	88	88	88	NA	NA	NA	NA	Y	Pa	NA	Ye	6	Ye
88	88	88	88	88	88	88	NA	NA	NA	NA	Y	Pa	NA	Ye	6	Ye
88	88	88	88	88	88	88	NA	NA	NA	NA	Y	Pa	NA	Ye	3	Ye
88	88	88	88	88	88	88	NA	NA	NA	NA	Y	Pa	NA	Ye	6	Ye
88	88	88	88	88	88	88	NA	NA	NA	NA	Y	Pa	NA	Ye	6	Ye
88	88	88	88	88	88	88	NA	NA	NA	NA	N		NA	Ye	9	Ye
88	88	88	88	88	88	88	NA	NA	NA	NA	Y	Pa	NA	Ye	6	Ye
88	88	88	88	88	88	88	NA	NA	NA	NA	N		NA	Ye	9	Ye
88	88	88	88	88	88	88	NA	NA	NA	NA	Y	Ot	NA	Ye	6	Ye
88	88	88	88	88	88	88	NA	NA	NA	NA	N		NA	Ye	9	Ye
88	88	88	88	88	88	88	NA	NA	NA	NA	Y	Pa	NA	Ye	6	Ye
88	88	88	88	88	88	88	NA	NA	NA	NA	Y	Pa	NA	Ye	6	Ye
88	88	88	88	88	88	88	NA	NA	NA	NA	Y	Pa	NA	Ye	6	Ye
88	88	88	88	88	88	88	NA	NA	NA	NA	Y	Pa	NA	Ye	6	Ye
88	88	88	88	88	88	88	NA	NA	NA	NA	N		NA	Ye	9	Ye
88	88	88	88	88	88	88	NA	NA	NA	NA	N		NA	Ye	9	Ye
88	88	88	88	88	88	88	NA	NA	NA	NA	N		NA	Ye	9	Ye
88	88	88	88	88	88	88	NA	NA	NA	NA	Y	Pa	NA	Ye	6	Ye
88	88	88	88	88	88	88	NA	NA	NA	NA	Y	Pa	NA	Ye	6	Ye
88	88	88	88	88	88	88	NA	NA	NA	NA	Y	Ot	NA	Ye	6	Ye
88	88	88	88	88	88	88	NA	NA	NA	NA	Y	Pa	NA	Ye	6	Ye
88	88	88	88	88	88	88	NA	NA	NA	NA	Y	Pa	NA	Ye	6	Ye
88	88	88	88	88	88	88	NA	NA	NA	NA	Y	Pa	NA	Ye	6	Ye
88	88	88	88	88	88	88	NA	NA	NA	NA	Y	Pa	NA	Ye	9	Ye
88	88	88	88	88	88	88	NA	NA	NA	NA	Y	Pa	NA	Ye	3	Ye
88	88	88	88	88	88	88	NA	NA	NA	NA	Y	Pa	NA	Ye	3	Ye
88	88	88	88	88	88	88	NA	NA	NA	NA	Y	Pa	NA	Ye	6	Ye
88	88	88	88	88	88	88	NA	NA	NA	NA			NA			
88	88	88	88	88	88	88	NA	NA	NA	NA	N		NA	Ye	9	Ye

88	88	88	88	88	88	88	NA	NA	NA	NA	N		NA	Ye	9	Ye
88	88	88	88	88	88	88	Ot	NA	NA	NA	Y	Ot	NA	Ye	6	Ye
88	88	88	88	88	88	88	NA	NA	NA	NA	N		NA	Ye	9	Ye
88	88	88	88	88	88	88	NA	NA	NA	NA	N		NA	Ye	9	Ye
88	88	88	88	88	88	88	NA	NA	NA	NA	Y	Pa	NA	Ye	6	Ye
88	88	88	88	88	88	88	NA	NA	NA	NA	Y	Pa	NA	Ye	6	Ye
88	88	88	88	88	88	88	NA	NA	NA	NA	Y	Pa	NA	Ye	6	Ye
88	88	88	88	88	88	88	NA	NA	NA	NA	Y	Pa	NA	Ye	6	Ye
88	88	88	88	88	88	88	NA	NA	NA	NA	Y	Pa	NA	Ye	6	Ye
88	88	88	88	88	88	88	NA	NA	NA	NA	Y	Pa	NA	Ye	3	Ye
88	88	88	88	88	88	88	NA	NA	NA	NA	Y	Pa	NA	Ye	6	Ye
88	88	88	88	88	88	88	NA	NA	NA	NA	Y	Pa	NA	Ye	6	Ye
88	88	88	88	88	88	88	NA	NA	NA	NA	Y	Pa	NA	Ye	6	Ye
88	88	88	88	88	88	88	Fa	NA	NA	NA	Y	Pa	NA	Ye	6	Ye
88	88	88	88	88	88	88	NA	NA	NA	NA	N		NA	Ye	9	Ye
88	88	88	88	88	88	88	NA	NA	NA	NA	Y	Pa	NA	Ye	6	Ye
88	88	88	88	88	88	88	NA	NA	NA	NA	N		NA	Ye	9	Ye
88	88	88	88	88	88	88	NA	NA	NA	NA	N		NA	Ye	9	Ye
88	88	88	88	88	88	88	NA	NA	NA	NA	Y	Pa	NA	Ye	6	Ye
88	88	88	88	88	88	88	NA	NA	NA	NA	Y	Ot	NA	Ye	6	Ye
88	88	88	88	88	88	88	NA	NA	NA	NA	Y	Pa	NA	Ye	6	Ye
88	88	88	88	88	88	88	NA	NA	NA	NA	Y	Pa	NA	Ye	6	Ye
88	88	88	88	88	88	88	NA	NA	NA	NA	Y	Pa	NA	Ye	6	Ye
88	88	88	88	88	88	88	Mo	NA	NA	NA	Y	Pa	NA	Ye	6	Ye
88	88	88	88	88	88	88	NA	NA	NA	NA	Y	Pa	NA	Ye	6	Ye
88	88	88	88	88	88	88	NA	NA	NA	NA	Y	Pa	NA	Ye	6	Ye
88	88	88	88	88	88	88	NA	NA	NA	NA	Y	Pa	NA	Ye	6	Ye
88	88	88	88	88	88	88	NA	NA	NA	NA	N		NA	Ye	9	Ye
88	88	88	88	88	88	88	NA	NA	NA	NA	N	Pa	NA	Ye	9	Ye
88	88	88	88	88	88	88	NA	NA	NA	NA	Y	Pa	NA	Ye	3	Ye
88	88	88	88	88	88	88	Mo	NA	NA	NA	Y	Pa	NA	Ye	6	Ye
88	88	88	88	88	88	88	NA	NA	NA	NA	Y	Ot	NA	Ye	6	Ye
88	88	88	88	88	88	88	Ot	NA	NA	NA	Y	Ot	NA	Ye	6	Ye
88	88	88	88	88	88	88	Mo	NA	NA	NA	Y	Pa	NA	Ye	6	Ye
88	88	88	88	88	88	88	NA	NA	NA	NA	Y	Pa	NA	Ye	6	Ye
88	88	88	88	88	88	88	NA	NA	NA	NA	Y	Pa	NA	Ye	6	Ye
88	88	88	88	88	88	88	NA	NA	NA	NA	Y	Pa	NA	Ye	6	Ye
88	88	88	88	88	88	88	NA	NA	NA	NA	Y	Pa	NA	Ye	6	Ye
88	88	88	88	88	88	88	NA	NA	NA	NA	N		NA	Ye	9	Ye
88	88	88	88	88	88	88	NA	NA	NA	NA	N		NA	Ye	9	Ye
88	88	88	88	88	88	88	NA	NA	NA	NA	Y	Ot	NA	Ye	6	Ye
88	88	88	88	88	88	88	NA	NA	NA	NA	N		NA	Ye	9	Ye
88	88	88	88	88	88	88	NA	NA	NA	NA	N		NA	Ye	9	Ye
88	88	88	88	88	88	88	NA	NA	NA	NA	Y	Pa	NA	Ye	6	Ye
88	88	88	88	88	88	88	NA	NA	NA	NA	Y	Pa	NA	Ye	6	Ye
88	88	88	88	88	88	88	NA	NA	NA	NA	Y	Ot	NA	Ye	9	Ye
88	88	88	88	88	88	88	NA	NA	NA	NA	Y	Pa	NA	Ye	9	Ye

88	88	88	88	88	88	88	NA	NA	NA	NA	Y	Pa	NA	Ye	9	Ye
88	88	88	88	88	88	88	NA	NA	NA	NA	Y		NA	Ye	6	Ye
88	88	88	88	88	88	88	NA	NA	NA	NA	Y	Pa	NA	Ye	6	Ye
88	88	88	88	88	88	88	NA	NA	NA	NA	N		NA	Ye	9	Ye
88	88	88	88	88	88	88	NA	NA	NA	NA	Y	Pa	NA	Ye	3	Ye
88	88	88	88	88	88	88	NA	NA	NA	NA	N		NA	Ye	9	Ye
88	88	88	88	88	88	88	NA	NA	NA	NA	Y	Pa	NA	Ye	6	Ye
88	88	88	88	88	88	88	NA	NA	NA	NA	Y	Pa	NA	Ye	6	Ye
88	88	88	88	88	88	88	NA	NA	NA	NA	Y	Pa	NA	Ye	6	Ye
88	88	88	88	88	88	88	NA	NA	NA	NA	Y	Pa	NA	Ye	6	Ye
88	88	88	88	88	88	88	NA	NA	NA	NA	Y	Pa	NA	Ye	3	Ye
88	88	88	88	88	88	88	NA	NA	NA	NA	Y	Pa	NA	Ye	6	Ye
88	88	88	88	88	88	88	NA	NA	NA	NA	Y	Pa	NA	Ye	6	Ye
88	88	88	88	88	88	88	NA	NA	NA	NA	Y	Pa	NA	Ye	6	Ye
88	88	88	88	88	88	88	NA	NA	NA	NA	Y	Pa	NA	Ye	6	Ye
88	88	88	88	88	88	88	Fa	NA	NA	NA	Y	Pa	NA	Ye	6	Ye
88	88	88	88	88	88	88	NA	NA	NA	NA	Y	Pa	NA	Ye	6	Ye
88	88	88	88	88	88	88	NA	NA	NA	NA	Y	Ot	NA	Ye	6	Ye
88	88	88	88	88	88	88	NA	NA	NA	NA	Y	Pa	NA	Ye	6	Ye
88	88	88	88	88	88	88	NA	NA	NA	NA	Y	Pa	NA	Ye	6	Ye
88	88	88	88	88	88	88	NA	NA	NA	NA	Y	Pa	NA	Ye	6	Ye
88	88	88	88	88	88	88	Mo	NA	NA	NA	Y	Pa	NA	Ye	6	Ye
88	88	88	88	88	88	88	NA	NA	NA	NA	Y	Pa	NA	Ye	6	Ye
88	88	88	88	88	88	88	NA	NA	NA	NA	Y	Pa	NA	Ye	6	Ye
88	88	88	88	88	88	88	NA	NA	NA	NA	Y	Ot	NA	Ye	6	Ye
88	88	88	88	88	88	88	NA	NA	NA	NA	Y	Pa	NA	Ye	6	Ye
88	88	88	88	88	88	88	NA	NA	NA	NA	Y	Pa	NA	Ye	3	Ye
88	88	88	88	88	88	88	Ot	NA	NA	NA	Y	Pa	NA	Ye	12	Ye
88	88	88	88	88	88	88	NA	NA	NA	NA	Y	Pa	NA	Ye	6	Ye
88	88	88	88	88	88	88	NA	NA	NA	NA	Y	Pa	NA	Ye	6	Ye
88	88	88	88	88	88	88	NA	NA	NA	NA	Y	Pa	NA	Ye	3	Ye
88	88	88	88	88	88	88	NA	NA	NA	NA	Y	Pa	NA	Ye	6	Ye
88	88	88	88	88	88	88	NA	NA	NA	NA	Y	Pa	NA	Ye	6	Ye
88	88	88	88	88	88	88	NA	NA	NA	NA	Y	Pa	NA	Ye	6	Ye
88	88	88	88	88	88	88	NA	NA	NA	NA	Y	Pa	NA	Ye	6	Ye
88	88	88	88	88	88	88	NA	NA	NA	NA	N		NA	Ye	9	Ye
88	88	88	88	88	88	88	NA	NA	NA	NA	Y	Pa	NA	Ye	6	Ye
88	88	88	88	88	88	88	NA	NA	NA	NA	Y	Pa	NA	Ye	3	Ye
88	88	88	88	88	88	88	NA	NA	NA	NA	Y	Pa	NA	Ye	6	Ye
88	88	88	88	88	88	88	NA	NA	NA	NA	Y	Pa	NA	Ye	3	Ye
88	88	88	88	88	88	88	NA	NA	NA	NA	Y	Pa	NA	Ye	6	Ye
88	88	88	88	88	88	88	NA	NA	NA	NA	Y	Pa	NA	Ye	3	Ye
88	88	88	88	88	88	88	NA	NA	NA	NA	N		NA	Ye	9	Ye
88	88	88	88	88	88	88	NA	NA	NA	NA	Y	Pa	NA	Ye	6	Ye
88	88	88	88	88	88	88	NA	NA	NA	NA	Y	Pa	NA	Ye	6	Ye
88	88	88	88	88	88	88	NA	NA	NA	NA	Y	Pa	NA	Ye	6	Ye
88	88	88	88	88	88	88	NA	NA	NA	NA	Y	Pa	NA	Ye	6	Ye

88	88	88	88	88	88	88	NA	NA	NA	NA	Y	Pa	NA	Ye	6	Ye
88	88	88	88	88	88	88	NA	NA	NA	NA	Y		NA	Ye	6	Ye
88	88	88	88	88	88	88	NA	NA	NA	NA	Y	Pa	NA	Ye	9	Ye
88	88	88	88	88	88	88	NA	NA	NA	NA	Y	Pa	NA	Ye	6	Ye
88	88	88	88	88	88	88	NA	NA	NA	NA	Y	Pa	NA	Ye	6	Ye
88	88	88	88	88	88	88	NA	NA	NA	NA	Y	Ot	NA	Ye	6	Ye
88	88	88	88	88	88	88	NA	NA	NA	NA	Y	Pa	NA	Ye	3	Ye
88	88	88	88	88	88	88	NA	NA	NA	NA	Y	Pa	NA	Ye	6	Ye
88	88	88	88	88	88	88	NA	NA	NA	NA	Y	Pa	NA	Ye	6	Ye
88	88	88	88	88	88	88	NA	NA	NA	NA	Y	Pa	NA	Ye	6	Ye
88	88	88	88	88	88	88	NA	NA	NA	NA	Y	Pa	NA	Ye	6	Ye
88	88	88	88	88	88	88	NA	NA	NA	NA	Y	Pa	NA	Ye	6	Ye
88	88	88	88	88	88	Af	Mo	NA	NA	NA	Y	Pa	NA	Ye	6	Ye
88	88	88	88	88	88	88	NA	NA	NA	NA	Y	Pa	NA	Ye	6	Ye
88	88	88	88	88	88	88	NA	NA	NA	NA	N		NA	Ye	9	Ye
88	88	88	88	88	88	88	NA	NA	NA	NA	Y	Pa	NA	Ye	6	Ye
88	88	88	88	88	88	88	NA	NA	NA	NA	Y	Pa	NA	Ye	6	Ye
88	88	88	88	88	88	88	NA	NA	NA	NA	Y	Pa	NA	Ye	6	Ye
88	88	88	88	88	88	88	NA	NA	NA	NA	Y	Pa	NA	Ye	3	Ye
88	88	88	88	88	88	88	NA	NA	NA	NA	N		NA	Ye	9	Ye
88	88	88	88	88	88	88	NA	NA	NA	NA	N		NA	Ye	9	Ye
88	88	88	88	88	88	88	NA	NA	NA	NA	Y	Pa	NA	Ye	6	Ye
88	88	88	88	88	88	88	NA	NA	NA	NA	Y	Pa	NA	Ye	3	Ye
88	88	88	88	88	88	88	NA	NA	NA	NA	Y	Pa	NA	Ye	6	Ye
88	88	88	88	88	88	88	NA	NA	NA	NA	N		NA	Ye	9	Ye
88	88	88	88	88	88	88	Mo	NA	NA	NA	Y	Pa	NA	Ye	6	Ye
88	88	88	88	88	88	88	NA	NA	NA	NA	Y	Pa	NA	Ye	6	Ye
88	88	88	88	88	88	88	NA	NA	NA	NA	Y	Pa	NA	Ye	6	Ye
88	88	88	88	88	88	88	NA	NA	NA	NA	Y	Ot	NA	Ye	9	Ye
88	88	88	88	88	88	88	NA	NA	NA	NA	N		NA	Ye	9	Ye
88	88	88	88	88	88	88	NA	NA	NA	NA	N		NA	Ye	9	Ye
88	88	88	88	88	88	88	NA	NA	NA	NA	Y	Pa	NA	Ye	6	Ye
88	88	88	88	88	88	88	NA	NA	NA	NA	N		NA	Ye	9	Ye
88	88	88	88	88	88	88	NA	NA	NA	NA	N		NA	Ye	9	Ye
88	88	88	88	88	88	88	NA	NA	NA	NA	N		NA	Ye	9	Ye
88	88	88	88	88	88	88	NA	NA	NA	NA	Y	Ot	NA	Ye	9	Ye
88	88	88	88	88	88	88	NA	NA	NA	NA	Y	Ot	NA	Ye	9	Ye
88	88	88	88	88	88	88	Mo	NA	NA	NA	Y	Pa	NA	Ye	6	Ye
88	88	88	88	88	88	88	NA	NA	NA	NA	Y	Ot	NA	Ye	6	Ye
88	88	88	88	88	88	88	NA	NA	NA	NA	Y	Pa	NA	Ye	6	Ye
88	88	88	88	88	88	88	NA	NA	NA	NA	Y	Pa	NA	Ye	6	Ye
88	88	88	88	88	88	88	NA	NA	NA	NA	Y	Ot	NA	Ye	6	Ye
88	88	88	88	88	88	88	NA	NA	NA	NA	Y	Pa	NA	Ye	6	Ye
88	88	88	88	88	88	88	Mo	NA	NA	NA	Y	Pa	NA	Ye	9	Ye
88	88	88	88	88	88	88	NA	NA	NA	NA	N		NA	Ye	9	Ye
88	88	88	88	88	88	88	NA	NA	NA	NA	N		NA	Ye	9	Ye
88	88	88	88	88	88	88	NA	NA	NA	NA	Y	Pa	NA	Ye	6	Ye

88	88	88	88	88	88	88	NA	NA	NA	NA	Y	Ot	NA	Ye	6	Ye
88	88	88	88	88	88	88	NA	NA	NA	NA	Y	Pa	NA	Ye	3	Ye
88	88	88	88	88	88	88	NA	NA	NA	NA	Y	Pa	NA	Ye	3	Ye
88	88	88	88	88	88	88	NA	NA	NA	NA	Y	Pa	NA	Ye	6	Ye
88	88	88	88	88	88	88	NA	NA	NA	NA	Y	Pa	NA	Ye	6	Ye
88	88	88	88	88	88	88	NA	NA	NA	NA	Y	Pa	NA	Ye	6	Ye
88	88	88	88	88	88	88	NA	NA	NA	NA	Y	Pa	NA	Ye	6	Ye
88	88	88	88	88	88	88	NA	NA	NA	NA	Y	Pa	NA	Ye	6	Ye
88	88	88	88	88	88	88	NA	NA	NA	NA	Y	Pa	NA	Ye	6	Ye
88	88	88	88	88	88	88	NA	NA	NA	NA	N		NA	Ye	9	Ye
88	88	88	88	88	88	88	NA	NA	NA	NA	Y	Pa	NA	Ye	6	Ye
88	88	88	88	88	88	88	Mo	NA	NA	NA	Y	Pa	NA	Ye	6	Ye
88	88	88	88	88	88	88	NA	NA	NA	NA	Y	Ot	NA	Ye	6	Ye
88	88	88	88	88	88	88	NA	NA	NA	NA	Y	Pa	NA	Ye	6	Ye
88	88	88	88	88	88	88	NA	NA	NA	NA	Y	Pa	NA	Ye	6	Ye
88	88	88	88	88	88	88	NA	NA	NA	NA	Y	Pa	NA	Ye	6	Ye
88	88	88	88	88	88	88	NA	NA	NA	NA	Y	Pa	NA	Ye	6	Ye
88	88	88	88	88	88	88	NA	NA	NA	NA	Y	Pa	NA	Ye	6	Ye
88	88	88	88	88	88	88	NA	NA	NA	NA	Y	Pa	NA	Ye	6	Ye
88	88	88	88	88	88	88	NA	NA	NA	NA	Y	Pa	NA	Ye	6	Ye
88	88	88	88	88	88	88	NA	NA	NA	NA	Y	Pa	NA	Ye	6	Ye
88	88	88	88	88	88	88	NA	NA	NA	NA	Y	Pa	NA	Ye	6	Ye
88	88	88	88	88	88	88	NA	NA	NA	NA	N		NA	Ye	9	Ye
88	88	88	88	88	88	88	NA	NA	NA	NA	Y	Pa	NA	Ye	6	Ye
88	88	88	88	88	88	88	Mo	NA	NA	NA	Y	Pa	NA	Ye	6	Ye
88	88	88	88	88	88	88	NA	NA	NA	NA	Y	Pa	NA	Ye	6	Ye
88	88	88	88	88	88	88	NA	NA	NA	NA	Y	Pa	NA	Ye	6	Ye
88	88	88	88	88	88	88	NA	NA	NA	NA	Y	Pa	NA	Ye	3	Ye
88	88	88	88	88	88	88	NA	NA	NA	NA	Y	Pa	NA	Ye	6	Ye
88	88	88	88	88	88	88	NA	NA	NA	NA	Y	Pa	NA	Ye	6	Ye
88	88	88	88	88	88	88	NA	NA	NA	NA	Y	Pa	NA	Ye	6	Ye
88	88	88	88	88	88	88	NA	NA	NA	NA	Y	Pa	NA	Ye	6	Ye
88	88	88	88	88	88	88	Mo	NA	NA	NA	Y	Pa	NA	Ye	6	Ye
88	88	88	88	88	88	88	NA	NA	NA	NA	Y	Pa	NA	Ye	6	Ye
88	88	88	88	88	88	88	NA	NA	NA	NA	Y	Pa	NA	Ye	6	Ye
88	88	88	88	88	88	88	Mo	NA	NA	NA			NA			
88	88	88	88	88	88	88	Mo	NA	NA	NA	Y	Pa	NA	Ye	6	Ye
88	88	88	88	88	88	88	NA	NA	NA	NA	Y	Pa	NA	Ye	6	Ye
88	88	88	88	88	88	88	NA	NA	NA	NA	Y	Ot	NA	Ye	6	Ye
88	88	88	88	88	88	88	Fa	NA	NA	NA	Y	Pa	NA	Ye	6	Ye
88	88	88	88	88	88	88	NA	NA	NA	NA	Y	Ot	NA	Ye	6	Ye
88	88	88	88	88	88	88	Ot	NA	NA	NA	Y	Ot	NA	Ye	6	Ye
88	88	88	88	88	88	88	Ot	NA	NA	NA	Y	Ot	NA	Ye	6	Ye
88	88	88	88	88	88	88	NA	NA	NA	NA	Y	Pa	NA	Ye	6	Ye
88	88	88	88	88	88	88	NA	NA	NA	NA	Y	Ot	NA	Ye	6	Ye
88	88	88	88	88	88	88	NA	NA	NA	NA	Y	Pa	NA	Ye	6	Ye
88	88	88	88	88	88	88	NA	NA	NA	NA	Y	Ot	NA	Ye	6	Ye

88	88	88	88	88	88	88	NA	NA	NA	NA	Y	Pa	NA	Ye	3	Ye
88	88	88	88	88	88	88	NA	NA	NA	NA	Y	Ot	NA	Ye	6	Ye
88	88	88	88	88	88	88	NA	NA	NA	NA	Y	Pa	NA	Ye	6	Ye
88	88	88	88	88	88	88	NA	NA	NA	NA	Y	Pa	NA	Ye	6	Ye
88	88	88	88	88	88	88	NA	NA	NA	NA	Y	Pa	NA	Ye	6	Ye
88	88	88	88	88	88	88	NA	NA	NA	NA	Y	Pa	NA	Ye	6	Ye
88	88	88	88	88	88	88	NA	NA	NA	NA	Y	Pa	NA	Ye	6	Ye
88	88	88	88	88	88	88	NA	NA	NA	NA	Y	Pa	NA	Ye	6	Ye
88	88	88	88	88	88	88	NA	NA	NA	NA	Y	Ot	NA	Ye	6	Ye
88	88	88	88	88	88	88	NA	NA	NA	NA	Y	Pa	NA	Ye	6	Ye
88	88	88	88	88	88	88	NA	NA	NA	NA	Y	Pa	NA	Ye	6	Ye
88	88	88	88	88	88	88	NA	NA	NA	NA	Y	Pa	NA	Ye	6	Ye
88	88	88	88	88	88	88	NA	NA	NA	NA	Y	Pa	NA	Ye	6	Ye
88	88	88	88	88	88	88	NA	NA	NA	NA	Y	Pa	NA	Ye	3	Ye
88	88	88	88	88	88	88	NA	NA	NA	NA	Y	Pa	NA	Ye	6	Ye
88	88	88	88	88	88	88	NA	NA	NA	NA	Y	Pa	NA	Ye	6	Ye
88	88	88	88	88	88	88	NA	NA	NA	NA	Y	Pa	NA	Ye	6	Ye
88	88	88	88	88	88	88	NA	NA	NA	NA	Y	Ot	NA	Ye	6	Ye

q71a	q71b	q71c	q71d	q71d1	q72	q73a	q73b	q73c	q74	q75	q75a
Y				NA	3			2	N		NA
Y				NA	3	2			Y	R	NA
Y				NA	3			3	N	R	NA
Y				NA	3	3			N		NA
Y		Y		NA	3	2			N		NA
		Y		NA	3	2			N		NA
Y				NA	3	2			N		NA
Y				NA	3			2	N		NA
		Y		NA	3	2			N		NA
Y		Y		NA	3	2			N		NA
Y				NA	3	3			N		NA
Y				NA	3	2			N		NA
Y				NA	3	2			N		NA
Y		Y		NA	3	1		1	N		NA
Y				NA	3	2			N		NA
Y				NA	3	3			N		NA
Y		Y		NA	3	3			N		NA
Y				NA	3	3			N		NA
			Y	DISPENCIN ASSISTANT	3	1	1	1	N		NA
Y				NA	3			2	N		NA
			Y	DISPENCIN ASSISTANT	3			2	N		NA
			Y	DISPENCIN ASSISTANT	3	1		1	N		NA
			Y	DISPENCYN ASSISTANT	3			2	N		NA
			Y	DISPENCINE ASSISTANT	3			3	N		NA
Y				NA	3			3	N		NA
Y				NA	3			2	N		NA
Y				NA	3	1			N		NA
Y				NA	3	2			N		NA
Y				NA	3	1			N		NA
Y				NA	3		3		N		NA
Y				NA	3			2	N		NA
Y				NA	3		2		N		NA
Y				NA	3			1	N		NA
		Y		NA	3	1	1	1	N		NA
		Y		NA	3	1	1	1	N		NA
		Y		NA	3	1	1	1	N		NA
		Y		NA	3	1	1	1	N		NA
		Y		NA	3	1		1	N		NA
		Y		NA	3	1		1	N		NA
		Y		NA	3	1		1	N		NA
		Y		NA	3	2			N		NA
		Y		NA	3	1		1	N		NA
		Y		NA	3	1		1	N		NA
Y				NA	3	2			Y	R	NA
Y				NA	3	1			N	R	NA
		Y		NA	3	1		1	N		NA

		Y		NA	3		2		N		NA
		Y		NA	3	1			N		NA
Y				NA	3	3			N		NA
Y				NA	3	1		1	N		NA
		Y		NA	3			2	N		NA
Y				NA	3	3			N	R	NA
Y				NA	3	2			Y	R	NA
Y				NA	3	2			Y	R	NA
Y				NA	3			3	N		NA
Y				NA	3	3			Y	R	NA
Y				NA	3	2			Y	R	NA
Y				NA	3	2			Y	R	NA
Y				NA	3			2	Y	R	NA
		Y		NA	3	2			N		NA
Y				NA	3			3	Y	R	NA
		Y		NA	3	2			N		NA
Y				NA	3			2	Y	R	NA
Y				NA	3			3	Y	R	NA
Y				NA	3	2			N		NA
		Y		NA	3	1		1	N		NA
		Y		NA	3			1	N		NA
Y				NA	3	2			N		NA
Y				NA	3	2			Y	R	NA
Y				NA	3			3	N		NA
Y				NA	3			2	N		NA
Y				NA	3			3	N		NA
		Y		NA	3	2			N		NA
Y				NA	3			3	Y	R	NA
Y				NA	3			2	Y	R	NA
		Y		NA	3	2			N		NA
		Y		NA	3			2	N		NA
Y				NA	3	2			N	R	NA
Y				NA	3	2			Y	R	NA
Y	N	N	N	NA	3		3		N		NA
Y	N	N	N	NA	3		3		N		NA
Y	N	N	N	NA	3		2		N		NA
Y	N	N	N	NA	3	2			N		NA
Y	N	N	N	NA	3			2	N		NA
Y	N	N	N	NA	3			2	N		NA
Y	N	N	N	NA	3	2			N		NA
Y	N	N	N	NA	3	1		1	N		NA
Y	N	N	N	NA	3	3			N		NA
Y	N	N	N	NA	3	1			Y	R	NA
Y	N	N	N	NA	3	1			Y	R	NA
Y	N	N	N	NA	3	2			Y	R	NA
				NA							NA
Y	N	N	N	NA	3	3			Y	R	NA

Y	Y	N	N	NA	3		3		N		NA
Y	N	N	N	NA	3	2			N		NA
Y	N	N	N	NA	3			3	N		NA
Y	N	N	N	NA	3	3			N		NA
Y	N	N	N	NA	3		2		N		NA
Y	N	N	N	NA	3			2	N		NA
Y	N	N	N	NA	3			2	N		NA
Y	N	N	N	NA	3			2	N		NA
Y	N	N	N	NA	3			2	N		NA
Y	N	N	N	NA	3			1	N		NA
Y	N	N	N	NA	3			2	N		NA
Y	N	N	N	NA	3		2		N		NA
Y	N	N	N	NA	3		2		N		NA
Y	N	N	N	NA	3	2			N		NA
Y	N	N	N	NA	3	3			N		NA
Y	N	N	N	NA	3	2			N		NA
Y	N	N	N	NA	3		3		N		NA
Y	N	N	N	NA	3		3		N		NA
Y	N	N	N	NA	3		2		N		NA
Y	N	N	N	NA	3	2			N		NA
Y	N	N	N	NA	3			2	N		NA
Y	N	N	N	NA	3			2	N		NA
Y	N	N	N	NA	3			2	N		NA
Y	N	N	N	NA	3			2	N		NA
Y	N	N	N	NA	3			2	N		NA
Y	N	N	N	NA	3			2	N		NA
Y	N	N	N	NA	3		2		N		NA
Y	N	N	N	NA	3	3			N		NA
Y	N	N	N	NA	3	3			N		NA
Y	N	N	N	NA	3		1		N		NA
Y	N	N	N	NA	3			2	Y	R	NA
Y	N	N	N	NA	3			2	N		NA
Y	N	N	N	NA	3		2		N		NA
Y	N	N	N	NA	3	2			Y	R	NA
Y	N	N	N	NA	3	2			N		NA
Y	N	N	N	NA	3	2			Y	R	NA
Y	N	N	N	NA	3	2			N		NA
Y	N	N	N	NA	3	2			Y	R	NA
Y	N	N	N	NA	3		3		N		NA
Y	N	N	N	NA	3		3		N		NA
Y	N	N	N	NA	3		2		N		NA
Y	N	N	N	NA	3			3	N		NA
Y	N	N	N	NA	3	3			N		NA
Y	N	N	N	NA	3	2			Y	R	NA
Y	N	N	N	NA	3	2			Y	R	NA
Y	N	N	N	NA	3	3			N		NA
Y	N	N	N	NA	3	3			N		NA

Y	N	N	N	NA	3	3			N		NA
Y	N	N	N	NA	3	2			N		NA
Y	N	N	N	NA	3	2			N		NA
Y	N	N	N	NA	3	3			N		NA
Y	N	N	N	NA	3	1			N		NA
Y	N	N	N	NA	3	3			N		NA
Y	N	N	N	NA	3	2			N		NA
Y	N	N	N	NA	3	2			N		NA
Y	N	N	N	NA	3			2	N		NA
Y	N	N	N	NA	3	2			N		NA
Y	N	N	N	NA	3	1			Y	R	NA
Y	N	N	N	NA	3			2	N		NA
Y	N	N	N	NA	3	2			N		NA
Y	N	N	N	NA	3	2			N		NA
Y	N	N	N	NA	3			2	N		NA
Y	N	N	N	NA	3			2	N		NA
Y	N	N	N	NA	3	2			N		NA
Y	N	N	N	NA	3	2			N		NA
Y	N	N	N	NA	2			2	N		NA
Y	N	N	N	NA	3			2	N		NA
Y	N	N	N	NA	3			2	N		NA
Y	N	N	N	NA	3	2			Y	R	NA
Y	N	N	N	NA	3	2			N		NA
Y	N	N	N	NA	3			2	N		NA
Y	N	N	N	NA	3	2			Y	R	NA
Y	N	N	N	NA	3	2			N		NA
Y	N	N	N	NA	3	1			N		NA
Y	N	N	N	NA	3		1		N		NA
Y	N	N	N	NA	3		2		N		NA
Y	N	N	N	NA	3	2			N		NA
Y	N	N	N	NA	3	1			N		NA
Y	N	N	N	NA	3			2	N		NA
Y	N	N	N	NA	3	2			N		NA
Y	N	N	N	NA	3	2			N		NA
Y	N	N	N	NA	3	2			N		NA
Y	N	N	N	NA	3		3		N		NA
Y	N	N	N	NA	3			2	N		NA
Y	N	N	N	NA	3		1		N		NA
Y	N	N	N	NA	3			2	N		NA
Y	N	N	N	NA	3	1			N		NA
Y	N	N	N	NA	3	2			N		NA
Y	N	N	N	NA	3	1			N		NA
Y	N	N	N	NA	3		3		N		NA
Y	N	N	N	NA	3		2		N		NA
Y	N	N	N	NA	3	2			N		NA
Y	N	N	N	NA	3	2			Y	R	NA
Y	N	N	N	NA	3			2	N		NA

Y	N	N	N	NA	3			2	N		NA
Y	N	N	N	NA	3	2			N		NA
Y	N	N	N	NA	3			3	Y	R	NA
Y	N	N	N	NA	3			2	N		NA
Y	N	N	N	NA	3			2	N		NA
Y	N	N	N	NA	3			2	N		NA
Y	N	N	N	NA	3	1			N		NA
Y	N	N	N	NA	3	2			N		NA
Y	N	N	N	NA	3		2		N		NA
Y	N	N	N	NA	3	2			Y	R	NA
Y	N	N	N	NA	3	2			Y	R	NA
Y	N	N	N	NA	3	2			N		NA
Y	N	N	N	NA	3	2			N		NA
Y	N	N	N	NA	3		2		N		NA
Y	N	N	N	NA	3			3	N		NA
Y	N	N	N	NA	3	2			N		NA
Y	N	N	N	NA	3	2			N		NA
Y	N	N	N	NA	3		2		N		NA
Y	N	N	N	NA	3	1			N		NA
Y	N	N	N	NA	3	3			N		NA
Y	N	N	N	NA	3			2	N		NA
Y	N	N	N	NA	3			2	N		NA
Y	N	N	N	NA	3	1			N		NA
Y	N	N	N	NA	3	2			Y	R	NA
Y	N	N	N	NA	3	3			Y	R	NA
Y	N	N	N	NA	3	2			Y	R	NA
Y	N	N	N	NA	3	2			Y	R	NA
Y	N	N	N	NA	3	2			Y	R	NA
Y	N	N	N	NA	3		3		N		NA
Y	N	N	N	NA	3	3			N		NA
Y	N	N	N	NA	3			3	N		NA
Y	N	N	N	NA	3			3	N		NA
Y	N	N	N	NA	3	3			N		NA
Y	N	N	N	NA	3	3			N		NA
Y	N	N	N	NA	3	3			N		NA
Y	N	N	N	NA	3	3			N		NA
Y	N	N	N	NA	3	3			Y	R	NA
Y	N	N	N	NA	3	2			N		NA
Y	N	N	N	NA	3	2			N		NA
Y	N	N	N	NA	3		2		N		NA
Y	N	N	N	NA	3		2		N		NA
Y	N	N	N	NA	3		2		N		NA
Y	N	N	N	NA	3		2		N		NA
Y	N	N	N	NA	3	3			N		NA
Y	N	N	N	NA	3	3			N		NA
Y	N	N	N	NA	3			3	N		NA
Y	N	N	N	NA	3			2	N		NA

Y	N	N	N	NA	3			2	N		NA
Y	N	N	N	NA	3	1			N		NA
Y	N	N	N	NA	3		1		N		NA
Y	N	N	N	NA	3	2			N		NA
Y	N	N	N	NA	3	2			N		NA
Y	N	N	N	NA	3			2	Y	R	NA
Y	N	N	N	NA	3			2	N		NA
Y	N	N	N	NA	3			2	N		NA
Y	N	N	N	NA	3			2	N		NA
Y	N	N	N	NA	3			3	N		NA
Y	N	N	N	NA	3	2			N		NA
Y	N	N	N	NA	3			2	N		NA
Y	N	N	N	NA	3			2	N		NA
Y	N	N	N	NA	3	2			N		NA
Y	N	N	N	NA	3	2			Y	R	NA
Y	N	N	N	NA	3	2			N		NA
Y	N	N	N	NA	3	2			N		NA
Y	N	N	N	NA	3	2			Y	R	NA
Y	N	N	N	NA	3	2			N		NA
Y	N	N	N	NA	3			2	N		NA
Y	N	N	N	NA	3	2			Y	R	NA
Y	N	N	N	NA	3	2			N		NA
Y	N	N	N	NA	3		3		N		NA
Y	N	N	N	NA	3		2		N		NA
Y	N	N	N	NA	3			2	N		NA
Y	N	N	N	NA	3	2			Y	R	NA
Y	N	N	N	NA	3			2	N		NA
Y	N	N	N	NA	3			1	Y	R	NA
Y	N	N	N	NA	3	2			N		NA
Y	N	N	N	NA	3	2			Y	R	NA
Y	N	N	N	NA	3			2	Y	R	NA
Y	N	N	N	NA	3	2			N		NA
Y	N	N	N	NA	3	2			N		NA
Y	N	N	N	NA	3	2			N		NA
Y	N	N	N	NA	3	1		1	N		NA
				NA							NA
Y	N	N	N	NA	3			2	N		NA
Y	N	N	N	NA	3			2	N		NA
Y	N	N	N	NA	3			2	Y	R	NA
Y	N	N	N	NA	3			2	Y	R	NA
Y	N	N	N	NA	3	2			N		NA
Y	N	N	N	NA	3	1		1	N		NA
Y	N	N	N	NA	3	1		1	N		NA
Y	N	N	N	NA	3		2		N		NA
Y	N	N	N	NA	3		2		N		NA
Y	N	N	N	NA	3		2		N		NA
Y	N	N	N	NA	3		2		N		NA

Y	N	N	N	NA	3		1		N		NA
Y	N	N	N	NA	3	2			N		NA
Y	N	N	N	NA	3		2		N		NA
Y	N	N	N	NA	3			2	Y	R	NA
Y	N	N	N	NA	3	2			Y	R	NA
Y	N	N	N	NA	3	2			Y	R	NA
Y	N	N	N	NA	3	2			Y	R	NA
Y	N	N	N	NA	3	2			Y	R	NA
Y	N	N	N	NA	3	2			Y	R	NA
Y	N	N	N	NA	3	2			Y	R	NA
Y	N	N	N	NA	3			2	N		NA
Y	N	N	N	NA	3	2			N		NA
Y	N	N	N	NA	3	2			N		NA
Y	N	N	N	NA	3	1			N		NA
Y	N	N	N	NA	3			2	N		NA
Y	N	N	N	NA	3	2			N		NA
Y	N	N	N	NA	3	2			N		NA
Y	N	N	N	NA	3			2	N		NA

q76a	q76b	q76c
PARACETAMOL	NESTRIM	
COUGH SYRUP	PARACETAMOL	CO-TRIMOZOLE
PARACETAMOL		
PARACETAMOL		
PARACETAMOL	NESTRIM	COUGH SYRUP (SAMALIN)
PARACETAMOL	PRAZIQUANTEL	
PARACETAMOL	ALUMINIUM HYDROXIDE	
PARACETAMOL	METRONIDAZOLE	WHITFIELD'S OINTMENT
NESTRIM	PARACETAMOL	SAMALIN
PARACETAMOL	ALUMINIUM HYDROXIDE	
PARACETAMOL		
PARACETAMOL SYRUP		
PARACETAMOL		
O.R.S	PARACETAMOL	
PARACETAMOL		
PARACETAMOL		
PARACETAMOL		
PARACETAMOL	CHLORPHENIRAMINE MALEATE	
FOLIC ACID	DICLOFEN	
CHLORPHENIRAMINE MALEATE	PARACETAMOL	
PARACETAMOL	MULTIVITE	
PARACETAMOL	MULTIVITAMIN	
PARACETAMOL	MULTIVITE	
PARACETAMOL	FOLIC ACID	
MULTIVITAMIN	PARACETAMOL	
ALUMINIUM HYDROXIDE	PARACETAMOL	
MULTIVITAMIN	PARACETAMOL	
PARACETAMOL	MULTIVITAMIN	
MULTIVITAMIN	PARACETAMOL	CLERMUC SYRUP
PARACETAMOL	MULTIVITE	
PARACETAMOL	MULTIVITE	ALUMINIUM HYDROXIDE
PARACETAMOL	MOLTIVITE	
PARACETAMOL	MULTIVITE	
IBUPROFEN	FOLIC ACID	
IBUPROFEN	AMOXYCILLIN	
DICLOFEN		
METRONIDAZOL	AMOXYCILLIN	PARACETAMOL
PARAMOL	MULTIVITAMIN	
PARACETAMOL	MULTIVITE	
MULTIVIT	PARACETAMOL	
PARACETAMOL	NESTRIM	
CLEMUCDS		
MUITIVITE	PARACETAMOL	
PARACETAMOL SYRUP	NOVAGYL SUSPENSION	
PARACETAMOL SYRUP	MULTIVITAMIN SYRUP	
PARACETAMOL	O.R.S	

PARACETAMOL	MULTIVITAMIN	KINAMOX
PARACETAMOL	KINAMOX	
PARACETAMOL	PROFEN	MULITIVITE
PARACETAMOL	MULTIVITE	
PARACETAMOL	MULTIVITE	AMOXYCILLIN
PARACETAMOL	MULTIVITAMIN TAB	
AMOXYCILLIN TAB	PARACETAMOL	
MULTIVITE TAB	PARACETAMOL	
PARACETAMOL	MULTIVITE	
NESTRIM	MUSCLE HEAT RUB	PARACETAMOL
FOLIC ACID	PARACETAMOL	
PARACETAMOL		
SYR CARBOCISTEINE	TAB PARACETAMOL	TAB CO-TRIMAZOLE
PARACETAMOL	MULTIVITE	
TAB EMGIPROFEN	MULTIVITAMIN TAB	
PRARCETAMOL	MULTIVITE	
MULTIVITAMIN SYRUP	COTRIZOLE SUSPENSION	PARACETAMOL SYRUP
PARACETAMOL SYRUP	SAMALIN COUGH SYRUP	INHIRFIELD'S OINTMENT
SYRUP PARACETAMOL	SAMALIN COUGH SYRUP	
PARACETAMOL	MULTIVITE	
PARACETAMOL	EXPECTOLYN	AMOXYCILLIN POWDER
MULTIVITAMIN SYRUP	PARACETAMOL SYRUP	
MULTIVITE TAB	FOLIC ACID	PARACETAMOL TAB
PARACETAMOL	MULTIVITE	ALIVERFENAC GEL
LINTUS	CO-TRIMAZOL	PARACETAMOL
PARACETAMOL	DICLOFENAC GEL	MULTIVITE
PARACETAMOL	RONFENAC GEL	
IBRUFEN TAB	COUGH SYRUP	
SAMALIN COUGH SYRUP	PARACETAMOL SYRUP	
PARACETAMOL	MULTIVITE	
PARACETAMOL	MULTIVITE	
TAB MULTIVITAMIN	TAB PARACETAMOL	
NESTRIM	PARACETAMOL	MUSCLE HEALTH BUB
BRUFEN	MULTIVITE	
ANATEN	MULTIVITE	
PARAMOL	MULTIVITE	
PARAMOL	MULTIVITE	
PARAMOL	MULTIVITE	
PARAMOL	MULTIVITE	
PARAMOL	MULTIVITE	
PARAMOL	MULTIVITE	
DEPTRIN	PARAMOL	MULTIVITE
METRONIDAZOLE SUSPENSION	PARACETAMOL SYRUP	
METRONIDAZOLE SUSPENSION	PARACETAMOL SYRUP	
	MULTIVITAMIN TAB	
AMOXYCILLIN SUSPENSION	PARACETAMOL SYRUP	
TOBUFEN TAB		

PARAMOL	MULTIVITE	
PARAMOL	MULTIVITAMIN SYRUP	
CHLORPHENIRAMIN	DEPRIN	PARAMOL
PARAMOL	MULTIVITE	
MULTIVITE	PARAMOL	
SAMALIN SYRUP	PARACETAMOL TAB	AMOXYCILLIN CAPSULES
SAMALIN SYRUP	EMGIPROFEN TAB	AMOXYCILLIN CAPSULES
SAMALIN SYRUP	AMOXYCILLIN CAPSULES	PARACETAMOL TAB
PARACETAMOL SYRUP	AMOXYCILLIN SUSPENSION	
METRONIDAZOLE SUSPENSION	PARACETAMOL SYRUP	
FOLIC ACID	PARACETAMOL TAB	MULTIVITAMIN TAB
EMGIPROFEN SYRUP	MULTIVITE SYRUP	
EMGIPROFEN	MULTIVITE	
BRUFEN	MULTIVITE	
PARAMOL	MULTIVITE	
MULTIVITE	ANFEN IBUPROFEN	
PARAMOL		
PARAMOL	MULTIVITE	
PARAMOL	AMOXICILLIN	
PARAMOL	MULTIVITE	
PARACETAMOL SYRUP	AMOXYCILLIN SUSPENSION	
METRONIDAZOLE SUSPENSION	HAEMOGLOBIN SYRUP	PARACETAMOL SYRUP
PARACETAMOL TAB		
METRONIDAZOLE SUSPENSION	PARACETAMOL SYRUP	
PARACETAMOL TAB	AMOXYCILLIN SUSPENSION	
PARACETAMOL TAB		
EMGIPROFEN	MULTIVITE	
MULTIVITE		
MULTIVITAMIN	IBRUPROFEN	
AMOXICILLIN POWDER	PARAMOL	
MULTIVITAMIN TAB	PARACETAMOL TAB	
PARAMOL	MULTIVITE	
PARAMOL	MULTIVITE	
SAMALIN SYRUP	AMOXYCILLIN SUSPENSION	
CO-TRIMOXAZOLE	PARACETAMOL SYRUP	
CO-TRIMOXAZOLE SUSPENSION	PARACETAMOL SYRUP	
PARACETAMOL SYRUP		
CO-TRIMOXAZOLE SUSPENSION	METRONIDAZOLE SUSPENSION	
PARAMOL		
PARAMOL	MULTIVITE	
DICLOFENAC		
TOBUFEN TAB	AMOXYCILLIN CAPSULE	
MULTIVITAMIN SYRUP	PARACETAMOL SYRUP	
SAMALIN COUGH SYRUP	AMOXYCILLIN SUSPENSION	PARACETAMOL SYRUP
TOBUFEN TAB	MULTIVITAMIN TAB	
DICLOFENAC TAB		

FOLIC ACID	TAB TOBUFEN	
FLUCLOXACILLIN SYRUP	PARACETAMOL SYRUP	
PARACETAMOL SYRUP	CLOTRIMAZOLE CREAM	
FOLIC ACID	TOBUFEN TAB	
METRONIDAZOLE SUSPENSION	CO-TRIMOXAZOLE SUSPENSION	PARACETAMOL SYRUP
MULTIVITE TABS	DICLOFINIC TABS	
MUCOTIN	MULTIVITE	PARAMOL
AMOXYCILLIN SUSPENSION	CO-TRIMOXAZOLE SUSPENSION	PARACETAMOL SYRUP
METRONIDAZOLE SUSPENSION	PARACETAMOL SYRUP	
LINCTUS COUGH SYRUP	AMOXYCILLIN SUSPENSION	PARACETAMOL SYRUP
AMOXYCILLIN SUSPENSION	LINCTUS COUGH SYRUP	PARACETAMOL SYRUP
ALUMINIUM HYDROXIDE TAB	PARACETAMOL TAB	
AMOXYCILLIN SUSPENSION	PARACETAMOL TAB	
PARAMOL SYRUP	MULTIVITE SYRUP	AMOXICILINE POWDER
PARAMOL SYRUP	MULTIVITE SYRUP	
MUCOTIN SYRUP	MULTIVITE SYRUP	PARAMOL SYRUP
PARAMOL SYRUP	MULTIVITE SYRUP	
PARAMOL	MULTIVITE	
MULTIVITE	PARAMOL	
PARAMOL	MULTIVITE	
PARAMOL	MULTIVITE	
MULTIVITAMIN TAB	PARACETAMOL	
PARACETAMOL TAB		
METRONIDAZOLE SUSPENSION		
MULTIVITAMIN TAB	PARACETAMOL TAM	
MULTIVITAMIN TAB		
MULTIVITAMIN SYRUP	PARACETAMOL SYRUP	
PARAMOL	MULTIVITE	
PARAMOL	DEPTRIN	
MULTIVITAMIN SYRUP	PARACETAMOL SYRUP	
MULTIVITAMIN SYRUP	PARACETAMOL SYRUP	
MULTIVITAMIN TAB	PARACETAMOL TAB	
PARAMOL	MULTIVITE	
PARACETAMOL SYRUP	AMOXICILLIN SUSPENSION	
MULTIVITE	PARAMOL	
MULTIVITE TABS	DICLOFEN	
AMOXICILLIN SUSPENSION		
PARAMOL SYRUP	METRONIDAZOL	
PARACETAMOL SYRUP	MULTIVITAMIN SYRUP	
PARACETAMOL SYRUP	ORS	
PARAMOL SYRUP	MULTIVITE SYRUP	
PARACETAMOL SYRUP	MULTIVITE SYRUP	
FOLIC ACID	PARAMOL	
PROFEN SYRUP	MULTIVITE SYRUP	
IBUPROFEN SUSPENSION	MULTIVITE TAB	
AMOXICILLIN SUSPENSION	MULTIVITAMIN SYRUP	PARACETAMOL SYRUP
METRONIDAZOLE TABLETS	PARACETAMOL SYRUP	

AMOXICILLIN SUSPENSION	PARACETAMOL SYRUP	
PARACETAMOL SYRUP		
MULTIVITAMIN TAB	DICLOFENAC TAB	FOLIC ACID
ANFEN IBUPROFEN	MULTIVITE	
PARAMOL	MULTIVITE	
PARAMOL SYRUP	MULTIVITE SYRUP	
MULTIVITE SYRUP	PARAMOL SYRUP	
PRUFEN	MULTIVITE	
PARAMOL	MULTIVITE	
FLUCLOXACILLIN ORAL SOLUTION	PARACETAMOL SYRUP	
HAEMOGLOBIN SYRUP	PARACETAMOL SYRUP	
PARACETAMOL SYRUP		
CO-TRIMOXAZOLE TAB	PARACETAMOL SYRUP	
PARAMOL	MULTIVITE	
PARAMOL	FOLIC ACID	
SAMALIN SYRUP		
ORS	PARACETAMOL SYRUP	
EMGIPROFEN	MULTIVITE SYRUP	
PARAMOL	MULTIVITAMIN SYRUP	
DICLOFENAC	MULTIVITE	
PARAMOL	MULTIVITAMIN	
PARAMOL	MULTIVITE	
PARAMOL	MULTIVITE	
TAB PARACETAMOL		
DICLOFENAC GEL		
PARACETAMOL TAB		
GENTAMICIN EYE DROPS	TAB PARACETAMOL	
TAB PARACETAMOL	FLUCOXALLIN SUSPENSION	
DICLOFEN	MULTIVITE	
PARAMOL	MULTIVITE	
PARAMOL	MULTIVITE	
PARAMOL	MULTIVITE	
DICLOFEN	MULTIVITE	
EMGIFLO CAPSULE	DICLOPAC TAB	TAB CETZIN
PARACETAMOL TAB		
TAB DICLOPAC		
FLUCLOXACILLIN CAPSULE		
FLUCLOXACILLIN SYRUP	METRONIDAZOLE SUSPENSION	TAB PARACETAMOL
ORS	TAB PARACETAMOL	
IBUPROFEN	MULTIVITE	
PARAMOL	MULTIVITE SYRUP	
MULTIVITE SYRUP	AMOXYCILLIN POWDER	
IBUPROFEN	MULTIVITE	
TAB MULTIVITAMIN	TAB PARACETAMOL	
MULTIVITAMIN TAB	PARACETAMOL TAB	
MULTIVITE	PARAMOL	
TAB PARACETAMOL	TAB MULTIVITAMIN	

TAB MULTIVITAMIN	TAB PARACETAMOL	
PARAMOL		
PARAMOL	CHLORPHENIRAMINE	
TAB PARACETAMOL	TAB MULTIVIMIN	
LINCTUS COUGH SYRUP	MULTIVITAMIN TAB	PARACETAMOL TAB
MULTIVITAMIN TAB		
SAMALIN SYRUP	MULTIVITAMIN	PARACETAMOL TAB ORS
FOLIC ACID	MULTIVITAMIN TAB	
MULTIVITAMIN TAB	FOLIC ACID	
PARAMOL	MULTIVITE	
MULTIVITAMIN TAB	PARACETAMOL TAB	
AMOXYCILLIN SUSPENSION	LINCTUS COUGH SYRUP	PARACETAMOL TAB
PARAMOL	MULTIVITE	
HYDROXIDE TAB	FLUXAKIN CAPSULES	PARACETAMOL TAB
FLUXAKIN CAPSULES	MULTIVITAMIN TAB	PARACETAMOL TAB
COTRIZOLE SUSPENSION	LINCTUS COUGH SYRUP	PARACETAMOL TAB
COTRIZOLE SUSPENSION	LINCTUS COUGH SYRUP	MULTIVITAMIN TAB & PARACETAMO
AMOXYCILLIN SUSPENSION	PARACETAMOL TAB	
METRONIDAZOLE SUSPENSION	CO-TRIMOXAZOLE SUSPENSION	
PARACETAMOL TAB	MULTIVITAMIN TAB	ALBENDAZOLE TAB
MULTIVITAMIN TAB	PARACETAMOL TAB	
AMOXYCILLIN CAPSULES	PARACETAMOL TAB	
TOBUFEN TABS	MULTIVITE TABS	
PARAMOL	MULTIVITE	
PARAMOL	DEPTRIN	
PARACETAMOL TAB	METRONIDAZOLE TAB	
AMOXYCILLIN SUSPENSION	PARACETAMOL TAB	
AMOXYCILLIN SUSPENSION	LINCTUS COUGH SYRUP	PARACETAMOL TAB
MULTIVITAMIN TABLETS	PARACETAMOL TABLETS	
PARACETAMOL TABLETS	LINCTUS COUGH SYRUP	
AMOXYCILLIN SUSPENSION	LINCTUS COUGH SYRUP	PARACETAMOL TAB
EMGIPROFEN		
MULTIVITE	PARAMOL	
PARAMOL	MULTIVITE	
EMGIPROFEN		
PARACETAMOL TAB	ALUMINIUM HYDROXIDE TAB	
DEPTRIN TAB	PARACETAMOL TAB	
SAMALIN SYRUP	DEPTRIN TAB	PARACETAMOL TAB
DEPTRIN TAB	PARACETAMOL TAB	
METRONIDAZOLE SUSPENSION	PARACETAMOL TAB	
DEPTRIN TAB	PARACETAMOL TAB	
PARAMOL	MULTIVITE	
PARAMOL	MULTIVITE	
CARBOZAP	PARAMOL	MULTIVITE
PARAMOL	MULTIVITE	
PARAMOL	MULTIVITE	
PARAMOL	MULTIVITE	

MUCOTIN SYRUP	PARAMOL SYRUP	
MULTIVITE	PARAMOL	
PARAMOL	MULTIVITE	
SAMALIN SYRUP	PARACETAMOL SYRUP	AMOXYCILLIN SUSPENSION
NEXCOFER BLOOD TONIC	AMOXYCILLIN SUSPENSION	LINCTUS SYRUP & PARACETAMOL S
COTRIZOLE SUSPENSION	PARACETAMOL SYRUP	
METRONIDAZOLE SUSPENSION	PARACETAMOL SYRUP	
COTRIZOLE SUSPENSION	HAEMOGLOBIN SYRUP	PARACETAMOL SYRUP
AMOXYCILLIN SUSPENSION	PARACETAMOL SYRUP	
AMOXYCILLIN SUSPENSION	LINCTUS SYRUP	PARACETAMOL SYRUP
FLUCLOXACILLIN SOLUTION	PARACETAMOL SYRUP	
HAEMOGLOBIN SYRUP	PARACETAMOL TAB	
PARACETAMOL TABLETS	METRONIDAZOLE TABLETS	
PARACETAMOL SYRUP		
PARACETAMOL SYRUP	MULTIVITAMIN TABLETS	
MULTIVITAMIN TAB	PARACETAMOL TAB	SAMALIN SYRUP
PARAMOL	MULTIVITE	
PARAMO TABS	MULTIVITE TABS	

q76d	q76e	q77	q78	q79	q80	q81h	q81n	q81d	q81a	q81m	q81di	q81ma	q81r	q81i	q81t	q81da	q81in
		N	I	Y	N	88	88	88	88	88	88	88	88	88	88	88	88
		N	I	Y	N	88	88	88	88	88	88	88	88	88	88	88	88
		N	I	Y	Y	88	88	88	Ye	88	88	88	88	88	88	88	88
		N	I	Y	N	88	88	88	88	88	88	88	88	88	88	88	88
		N	I	Y	N	88	88	88	88	88	88	88	88	88	88	88	88
		N	I	Y	N	88	88	88	88	88	88	88	88	88	88	88	88
		N	I	Y	N	88	88	88	88	88	88	88	88	88	88	88	88
		N	I	Y	N	88	88	88	88	88	88	88	88	88	88	88	88
		N	I	Y	N	88	88	88	88	88	88	88	88	88	88	88	88
		N	I	Y	Y	88	88	Ye	88	88	88	88	88	88	88	88	88
		N	I	Y	N	88	88	88	88	88	88	88	88	88	88	88	88
		N	I	Y	N	88	88	88	88	88	88	88	88	88	88	88	88
		N	I	Y	N	88	88	88	88	88	88	88	88	88	88	88	88
		N	I	Y	N	88	88	88	88	88	88	88	88	88	88	88	88
		N	I	Y	N	88	88	88	88	88	88	88	88	88	88	88	88
		N	I	Y	Y	88	88	Ye	88	88	88	88	88	88	88	88	88
		N	I	Y	N	88	88	88	88	88	88	88	88	88	88	88	88
		N	I	Y	Y	88	88	Ye	88	88	88	88	88	88	88	88	88
		N	I	Y	N	88	88	88	88	88	88	88	88	88	88	88	88
		N	I	Y	N	88	88	88	88	88	88	88	88	88	88	88	88
		N	I	Y	N	88	88	88	88	88	88	88	88	88	88	88	88
		N	I	Y	N	88	88	88	88	88	88	88	88	88	88	88	88
		N	I	Y	N	88	88	88	88	88	88	88	88	88	88	88	88
		N	I	Y	N	88	88	88	88	88	88	88	88	88	88	88	88
		N	I	N	N	88	88	88	88	88	88	88	88	88	88	88	88
		N	I	Y	N	88	88	88	88	88	88	88	88	88	88	88	88
		N	I	Y	Y	88	88	88	88	88	88	88	88	88	88	88	88
		N	I	Y	N	88	88	88	88	88	88	88	88	88	88	88	88
		N	I	Y	N	88	88	88	88	88	88	88	88	88	88	88	88
		N	I	Y	Y	88	88	88	88	88	88	88	88	88	88	88	88
		N	I	Y	N	88	88	88	88	88	88	88	88	88	88	88	88
		N	I	Y	N	88	88	88	88	88	88	88	88	88	88	88	88
		N	I	Y	N	88	88	88	88	88	88	88	88	88	88	88	88
		N	I	Y	N	88	88	88	88	88	88	88	88	88	88	88	88
		N	I	N	Y	88	88	88	88	88	88	88	88	88	88	88	88
		N	I	Y	Y	88	88	88	88	88	88	88	88	88	88	88	88
		N	I	Y	Y	88	88	88	Ye	88	88	88	88	88	88	88	88
		N	I	Y	N	88	88	88	88	88	88	88	88	88	88	88	88
		N	I	Y	N	88	88	88	88	88	88	88	88	88	88	88	88
		N	I	Y	N	88	88	88	88	88	88	88	88	88	88	88	88
		Y	I	Y	N	88	88	88	88	88	88	88	88	88	88	88	88
		N	I	Y	N	88	88	88	88	88	88	88	88	88	88	88	88
		N	I	Y	Y	88	88	88	88	88	88	88	88	88	88	88	88
		Y	I	Y	N	88	88	88	88	88	88	88	88	88	88	88	88
		Y	I	Y	N	88	88	88	88	88	88	88	88	88	88	88	88
		N	I	Y	N	88	88	88	88	88	88	88	88	88	88	88	88

N	I	Y	N	88	88	88	88	88	88	88	88	88	88	88	88
N	I	Y	N	88	88	88	88	88	88	88	88	88	88	88	88
N	I	Y	N	88	88	88	88	88	88	88	88	88	88	88	88
N	I	Y	N	88	88	88	88	88	88	88	88	88	88	88	88
N	I	Y	N	88	88	88	88	88	88	88	88	88	88	88	88
N	I	Y		88	Ye	Ye	88	88	88	88	88	88	88	88	88
N	I	Y	N	88	88	88	88	88	88	88	88	88	88	88	88
N	I	Y	N	88	88	88	88	88	88	88	88	88	88	88	88
N	I	Y	N	88	88	88	88	88	88	88	88	88	88	88	88
N	I	Y	N	88	88	88	88	88	88	88	88	88	88	88	88
N	I	Y	N	88	88	88	88	88	88	88	88	88	88	88	88
N	I	Y	N	88	88	88	88	88	88	88	88	88	88	88	88
N	I	Y	N	88	88	88	88	88	88	88	88	88	88	88	88
N	I	Y	N	88	88	88	88	88	88	88	88	88	88	88	88
N	I	Y	N	88	88	88	88	88	88	88	88	88	88	88	88
N	I	Y	N	88	88	88	88	88	88	88	88	88	88	88	88
Y	I	Y	N	88	88	88	88	88	88	88	88	88	88	88	88
N	I	Y	N	88	88	88	88	88	88	88	88	88	88	88	88
Y	I	Y	N	88	88	88	88	88	88	88	88	88	88	88	88
N	I	Y	N	88	88	88	88	88	88	88	88	88	88	88	88
Y	N	N	N	88	88	88	88	88	88	88	88	88	88	88	88
N	I	Y	N	88	88	88	88	88	88	88	88	88	88	88	88
N	I	Y	N	88	88	88	88	88	88	88	88	88	88	88	88
N	I	Y	N	88	88	88	88	88	88	88	88	88	88	88	88
N	I	Y	N	88	88	88	88	88	88	88	88	88	88	88	88
N	I	Y	N	88	88	88	88	88	88	88	88	88	88	88	88
N	I	Y	N	88	88	88	88	88	88	88	88	88	88	88	88
N	I	Y	N	88	88	88	88	88	88	88	88	88	88	88	88
N	I	Y	N	88	88	88	88	88	88	88	88	88	88	88	88
N	I	Y	N	88	88	88	88	88	88	88	88	88	88	88	88
N	I	Y	N	88	88	88	88	88	88	88	88	88	88	88	88
N	I	Y	N	88	88	88	88	88	88	88	88	88	88	88	88
N	I	Y	N	88	88	88	88	88	88	88	88	88	88	88	88
N	I	Y	N	88	88	88	88	88	88	88	88	88	88	88	88
N	I	Y	N	88	88	88	88	88	88	88	88	88	88	88	88
N	I	Y	N	88	88	88	88	88	88	88	88	88	88	88	88
N	I	Y	N	88	88	88	88	88	88	88	88	88	88	88	88
N	I	Y	Y	No	No	No	No	No	No	No	No	No	No	No	No
N	I	Y	N	88	88	88	88	88	88	88	88	88	88	88	88
N	I	Y	N	88	88	88	88	88	88	88	88	88	88	88	88
N	I	Y	N	88	88	88	88	88	88	88	88	88	88	88	88
N	I	Y	N	88	88	88	88	88	88	88	88	88	88	88	88
Y	I	Y	Y	No	No	No	No	No	No	No	No	Ye	No	No	No
Y	I	Y	Y	No	No	No	No	No	No	No	No	Ye	No	No	No
N	I	Y	N	88	88	88	88	88	88	88	88	88	88	88	88
N	I	Y	N	88	88	88	88	88	88	88	88	88	88	88	88
N	I		N	88	88	88	88	88	88	88	88	88	88	88	88

N	I	Y	N	88	88	88	88	88	88	88	88	88	88	88	88
N	I	Y	N	88	88	88	88	88	88	88	88	88	88	88	88
N	I	Y	N	88	88	88	88	88	88	88	88	88	88	88	88
N	I	Y	N	88	88	88	88	88	88	88	88	88	88	88	88
N	I	Y	N	88	88	88	88	88	88	88	88	88	88	88	88
N	I	Y	N	88	88	88	88	88	88	88	88	88	88	88	88
N	I	Y	N	88	88	88	88	88	88	88	88	88	88	88	88
N	I	Y	N	88	88	88	88	88	88	88	88	88	88	88	88
N	I	Y	N	88	88	88	88	88	88	88	88	88	88	88	88
N	I	Y	N	88	88	88	88	88	88	88	88	88	88	88	88
N	I	Y	N	88	88	88	88	88	88	88	88	88	88	88	88
N	I	Y	N	88	88	88	88	88	88	88	88	88	88	88	88
N	I	Y	N	88	88	88	88	88	88	88	88	88	88	88	88
N	I	Y	N	88	88	88	88	88	88	88	88	88	88	88	88
N	I	Y	N	88	88	88	88	88	88	88	88	88	88	88	88
N	I	Y	N	88	88	88	88	88	88	88	88	88	88	88	88
N	I	Y	N	88	88	88	88	88	88	88	88	88	88	88	88
N	I	Y	N	88	88	88	88	88	88	88	88	88	88	88	88
N	I	Y	N	88	88	88	88	88	88	88	88	88	88	88	88
N	I	Y	Y	No	No	No	No	No	No	No	No	No	No	No	No
N	I	Y	N	88	88	88	88	88	88	88	88	88	88	88	88
N	I	Y	N	88	88	88	88	88	88	88	88	88	88	88	88
N	I	Y	N	88	88	88	88	88	88	88	88	88	88	88	88
Y	I	Y	N	88	88	88	88	88	88	88	88	88	88	88	88
Y	I	Y	N	88	88	88	88	88	88	88	88	88	88	88	88
N	I	Y	N	88	88	88	88	88	88	88	88	88	88	88	88
N	I	Y	N	88	88	88	88	88	88	88	88	88	88	88	88
N	I	Y	N	88	88	88	88	88	88	88	88	88	88	88	88
N	I	Y	Y	No	No	No	No	No	No	No	No	No	No	No	No
N	I	Y	N	88	88	88	88	88	88	88	88	88	88	88	88
N	I	Y	N	88	88	88	88	88	88	88	88	88	88	88	88
N	I	Y	Y	No	No	No	No	No	No	No	No	Ye	No	No	No
N	I	Y	N	88	88	88	88	88	88	88	88	88	88	88	88
N	I	Y	N	88	88	88	88	88	88	88	88	88	88	88	88
N	I	Y	N	88	88	88	88	88	88	88	88	88	88	88	88
N	I	Y	N	88	88	88	88	88	88	88	88	88	88	88	88
N	I	Y	N	88	88	88	88	88	88	88	88	88	88	88	88
N	I	Y	N	88	88	88	88	88	88	88	88	88	88	88	88
Y	N	N	Y	Ye	No	No	Ye	No	No	No	No	No	No	No	No
N	I	Y	N	88	88	88	88	88	88	88	88	88	88	88	88
N	I	Y	N	88	88	88	88	88	88	88	88	88	88	88	88
N	I	Y	N	88	88	88	88	88	88	88	88	88	88	88	88
N	I	Y	Y	No	No	No	No	No	No	No	No	Ye	No	No	No
N	I	Y	N	88	88	88	88	88	88	88	88	88	88	88	88
N	I	Y	N	88	88	88	88	88	88	88	88	88	88	88	88
N	I	Y	N	88	88	88	88	88	88	88	88	88	88	88	88
N	I	Y	N	88	88	88	88	88	88	88	88	88	88	88	88

N	I	Y	N	88	88	88	88	88	88	88	88	88	88	88	88
N	I	Y	N	88	88	88	88	88	88	88	88	88	88	88	88
N	I	Y	N	88	88	88	88	88	88	88	88	88	88	88	88
N	I	Y	Y	No	No	No	No	No	No	No	No	Ye	No	No	No
N	I	Y	N	88	88	88	88	88	88	88	88	88	88	88	88
N	I	Y	Y	No	No	No	No	No	No	No	No	No	No	No	No
N	I	Y	N	88	88	88	88	88	88	88	88	88	88	88	88
N	I	Y	N	88	88	88	88	88	88	88	88	88	88	88	88
N	I	Y	N	88	88	88	88	88	88	88	88	88	88	88	88
Y	I		N	88	88	88	88	88	88	88	88	88	88	88	88
N	I	Y	N	88	88	88	88	88	88	88	88	88	88	88	88
N	I	Y	N	88	88	88	88	88	88	88	88	88	88	88	88
N	I	Y	N	88	88	88	88	88	88	88	88	88	88	88	88
N	I	Y	N	88	88	88	88	88	88	88	88	88	88	88	88
N	I	Y	N	88	88	88	88	88	88	88	88	88	88	88	88
N	I	Y	N	88	88	88	88	88	88	88	88	88	88	88	88
N	I	Y	N	88	88	88	88	88	88	88	88	88	88	88	88
N	I	Y	N	88	88	88	88	88	88	88	88	88	88	88	88
N	I	Y	N	88	88	88	88	88	88	88	88	88	88	88	88
N	I	Y	N	88	88	88	88	88	88	88	88	88	88	88	88
N	I	Y	N	88	88	88	88	88	88	88	88	88	88	88	88
N	I	Y	Y	No	No	Ye	No	No	No	No	No	No	No	No	No
N	I	Y	N	88	88	88	88	88	88	88	88	88	88	88	88
N	I	Y	N	88	88	88	88	88	88	88	88	88	88	88	88
N	I	Y	N	88	88	88	88	88	88	88	88	88	88	88	88
N	I	Y	N	88	88	88	88	88	88	88	88	88	88	88	88
N	I	Y	N	88	88	88	88	88	88	88	88	88	88	88	88
N	I	Y	N	88	88	88	88	88	88	88	88	88	88	88	88
N	I	Y	Y	No	No	No	No	No	No	No	No	No	No	No	No
Y	I	Y	N	88	88	88	88	88	88	88	88	88	88	88	88
N	I	Y	N	88	88	88	88	88	88	88	88	88	88	88	88
N	I	Y	N	88	88	88	88	88	88	88	88	88	88	88	88
N	I	Y	N	88	88	88	88	88	88	88	88	88	88	88	88
N	I	Y	N	88	88	88	88	88	88	88	88	88	88	88	88
N	I	Y	N	88	88	88	88	88	88	88	88	88	88	88	88
N	I	Y	Y	Ye	No	No	No	No	No	No	No	No	No	No	No
N	I	Y	N	88	88	88	88	88	88	88	88	88	88	88	88
N	I	Y	N	88	88	88	88	88	88	88	88	88	88	88	88
N	I	Y	N	88	88	88	88	88	88	88	88	88	88	88	88
N	I	Y	N	88	88	88	88	88	88	88	88	88	88	88	88
N	I	Y	N	88	88	88	88	88	88	88	88	88	88	88	88
N	I	Y	N	88	88	88	88	88	88	88	88	88	88	88	88
N	I	Y	N	88	88	88	88	88	88	88	88	88	88	88	88
N	I	Y	N	88	88	88	88	88	88	88	88	88	88	88	88
N	I	Y	N	88	88	88	88	88	88	88	88	88	88	88	88
N	I	Y	N	88	88	88	88	88	88	88	88	88	88	88	88
N	I	Y	N	88	88	88	88	88	88	88	88	88	88	88	88

N	I	Y	N	88	88	88	88	88	88	88	88	88	88	88	88
Y	N	N	Y	Ye	No	Ye	Ye	No	No	No	No	No	No	No	No
N	I	Y	N	88	88	88	88	88	88	88	88	88	88	88	88
N	I	Y	Y	No	No	No	No	No	No	No	No	No	No	Ye	No
N	I	Y	N	88	88	88	88	88	88	88	88	88	88	88	88
N	I	Y	Y	No	No	Ye	No	No	No	No	No	No	No	No	No
N	I	Y	Y	No	No	No	No	No	No	No	No	No	No	Ye	No
N	I	Y	N	88	88	88	88	88	88	88	88	88	88	88	88
N	I	Y	N	88	88	88	88	88	88	88	88	88	88	88	88
N	I	Y	N	88	88	88	88	88	88	88	88	88	88	88	88
N	I	Y	N	88	88	88	88	88	88	88	88	88	88	88	88
N	I	Y	N	88	88	88	88	88	88	88	88	88	88	88	88
N	I	Y	N	88	88	88	88	88	88	88	88	88	88	88	88
N	I	Y	N	88	88	88	88	88	88	88	88	88	88	88	88
N	I	Y	Y	No	No	No	No	No	No	No	No	No	No	No	No
N	I	Y	N	88	88	88	88	88	88	88	88	88	88	88	88
N	I	Y	N	88	88	88	88	88	88	88	88	88	88	88	88
N	I	Y	Y	Ye	No	No	No	No	No	No	No	No	No	No	No
N	I	Y	N	88	88	88	88	88	88	88	88	88	88	88	88
N	I	Y	N	88	88	88	88	88	88	88	88	88	88	88	88
N	I	Y	N	88	88	88	88	88	88	88	88	88	88	88	88
N	I	Y	N	88	88	88	88	88	88	88	88	88	88	88	88
N	I	Y	N	88	88	88	88	88	88	88	88	88	88	88	88
N	I	Y	N	88	88	88	88	88	88	88	88	88	88	88	88
N	I	Y	N	88	88	88	88	88	88	88	88	88	88	88	88
N	I	Y	N	88	88	88	88	88	88	88	88	88	88	88	88
N	I	Y	N	88	88	88	88	88	88	88	88	88	88	88	88
N	I	Y	N	88	88	88	88	88	88	88	88	88	88	88	88
N	I	Y	N	88	88	88	88	88	88	88	88	88	88	88	88
N	I	Y	N	88	88	88	88	88	88	88	88	88	88	88	88
N	I	Y	N	88	88	88	88	88	88	88	88	88	88	88	88
N	I	Y	N	88	88	88	88	88	88	88	88	88	88	88	88
N	I	Y	N	88	88	88	88	88	88	88	88	88	88	88	88
N	I	Y	N	88	88	88	88	88	88	88	88	88	88	88	88
N	I	Y	N	88	88	88	88	88	88	88	88	88	88	88	88
N	I	Y	N	88	88	88	88	88	88	88	88	88	88	88	88
N	I	Y	N	88	88	88	88	88	88	88	88	88	88	88	88
N	I	Y	N	88	88	88	88	88	88	88	88	88	88	88	88
N	I	Y	N	88	88	88	88	88	88	88	88	88	88	88	88
Y	I	Y	N	88	88	88	88	88	88	88	88	88	88	88	88
N	I	Y	N	88	88	88	88	88	88	88	88	88	88	88	88
N	I	Y	N	88	88	88	88	88	88	88	88	88	88	88	88
N	I	Y	N	88	88	88	88	88	88	88	88	88	88	88	88
N	I	Y	Y	No	No	No	Ye	No	No	No	No	No	No	No	No
N	I	Y	N	88	88	88	88	88	88	88	88	88	88	88	88
N	I	Y	N	88	88	88	88	88	88	88	88	88	88	88	88
N	I	Y	N	88	88	88	88	88	88	88	88	88	88	88	88

N	I	Y	N	88	88	88	88	88	88	88	88	88	88	88	88
N	I	Y	N	88	88	88	88	88	88	88	88	88	88	88	88
N	I	Y	N	88	88	88	88	88	88	88	88	88	88	88	88
N	I	Y	N	88	88	88	88	88	88	88	88	88	88	88	88
N	I	Y	N	88	88	88	88	88	88	88	88	88	88	88	88
N	I	Y	N	88	88	88	88	88	88	88	88	88	88	88	88
N	I	Y	N	88	88	88	88	88	88	88	88	88	88	88	88
N	I	Y	N	88	88	88	88	88	88	88	88	88	88	88	88
N	I	Y	N	88	88	88	88	88	88	88	88	88	88	88	88
N	I	Y	N	88	88	88	88	88	88	88	88	88	88	88	88
N	I	Y	N	88	88	88	88	88	88	88	88	88	88	88	88
N	I	Y	N	88	88	88	88	88	88	88	88	88	88	88	88
N	I	Y	N	88	88	88	88	88	88	88	88	88	88	88	88
N	I	Y	N	88	88	88	88	88	88	88	88	88	88	88	88
N	I	N	N	88	88	88	88	88	88	88	88	88	88	88	88
Y	I	Y	N	88	88	88	88	88	88	88	88	88	88	88	88
N	I	Y	N	88	88	88	88	88	88	88	88	88	88	88	88
N	I	Y	N	88	88	88	88	88	88	88	88	88	88	88	88
N	I	Y	N	88	88	88	88	88	88	88	88	88	88	88	88
N	I	Y	Y	No	No	Ye	No	No	No	No	No	No	No	No	No
N	I	Y	N	88	88	88	88	88	88	88	88	88	88	88	88
N	I	Y	Y	No	No	No	No	No	No	No	Ye	No	No	No	No
N	I	Y	N	88	88	88	88	88	88	88	88	88	88	88	88
N	I	Y	N	88	88	88	88	88	88	88	88	88	88	88	88
N	I	Y	N	88	88	88	88	88	88	88	88	88	88	88	88
N	I	Y	Y	No	No	Ye	No	No	No	No	No	No	No	No	No
N	I	Y	N	88	88	88	88	88	88	88	88	88	88	88	88
N	I	Y	N	88	88	88	88	88	88	88	88	88	88	88	88
N	I	Y	N	88	88	88	88	88	88	88	88	88	88	88	88
Y	N	N	N	88	88	88	88	88	88	88	88	88	88	88	88
N	I	Y	N	88	88	88	88	88	88	88	88	88	88	88	88
N	I	Y	N	88	88	88	88	88	88	88	88	88	88	88	88
N	I	Y	N	88	88	88	88	88	88	88	88	88	88	88	88
N	I	Y	N	88	88	88	88	88	88	88	88	88	88	88	88
N	I	Y	N	88	88	88	88	88	88	88	88	88	88	88	88
N	I	Y	N	88	88	88	88	88	88	88	88	88	88	88	88
N	I	Y	N	88	88	88	88	88	88	88	88	88	88	88	88
N	I	Y	N	88	88	88	88	88	88	88	88	88	88	88	88
N	I	Y	N	88	88	88	88	88	88	88	88	88	88	88	88
N	I	Y	N	88	88	88	88	88	88	88	88	88	88	88	88
Y	I	Y	N	88	88	88	88	88	88	88	88	88	88	88	88
N	I	Y	N	88	88	88	88	88	88	88	88	88	88	88	88
N	I	Y	N	88	88	88	88	88	88	88	88	88	88	88	88
N	I	Y	Y	No	No	No	No	No	No	No	No	No	No	No	No
N	I	Y	N	88	88	88	88	88	88	88	88	88	88	88	88
N	I	Y	N	88	88	88	88	88	88	88	88	88	88	88	88
N	I	Y	N	88	88	88	88	88	88	88	88	88	88	88	88

N	I	Y	N	88	88	88	88	88	88	88	88	88	88	88	88
N	I	Y	N	88	88	88	88	88	88	88	88	88	88	88	88
N	I	Y	N	88	88	88	88	88	88	88	88	88	88	88	88
Y	I	Y	N	88	88	88	88	88	88	88	88	88	88	88	88
N	I	Y	N	88	88	88	88	88	88	88	88	88	88	88	88
N	I	Y	N	88	88	88	88	88	88	88	88	88	88	88	88
N	I	Y	N	88	88	88	88	88	88	88	88	88	88	88	88
N	I	Y	N	88	88	88	88	88	88	88	88	88	88	88	88
N	I	Y	N	88	88	88	88	88	88	88	88	88	88	88	88
Y	I	Y	N	88	88	88	88	88	88	88	88	88	88	88	88
Y	I	Y	N	88	88	88	88	88	88	88	88	88	88	88	88
N	I	Y	N	88	88	88	88	88	88	88	88	88	88	88	88
Y	I	Y	N	88	88	88	88	88	88	88	88	88	88	88	88
N	I	Y	N	88	88	88	88	88	88	88	88	88	88	88	88
N	I	Y	N	88	88	88	88	88	88	88	88	88	88	88	88
Y	I	Y	N	88	88	88	88	88	88	88	88	88	88	88	88
N	I	Y	N	88	88	88	88	88	88	88	88	88	88	88	88
N	I	Y	Y	No	No	No	Ye	No	No	No	No	No	No	No	No

q81o	q81os	q82	q83ant1	q83ant1a
88	NA	2	1	DUO-COTECXIN
88	NA	2	1	DUO-COTECXIN
88	NA	2	1	DUO-COTECXIN
88	NA	2	1	DUO-COTECXIN
88	NA	2	1	DUO-COTECXIN
88	NA	2	1	DUO-COTECXIN
88	NA	2	1	DUO-COTECXIN
88	NA	2	1	
88	NA	2	1	DUO-COTECXIN
88	NA	2	1	DUO-COTECXIN
88	NA	2	1	DUO-COTECXIN
88	NA	2	1	
88	NA	2	1	DUO-COTECXIN
88	NA	2	1	DUO-COTECXIN
88	NA	2	1	DUO-COTECXIN
Ye	LOSS OF APPETCFE	2	1	DUO-COTECXIN
88	NA	2	1	
88	NA	2	1	DUO-COTECXIN
88	NA	2	1	DUO-COTECXIN
88	NA	2	1	DUO-COTEXCIN
88	NA	2	1	DUO-COTECXIN
88	NA	2	1	DUO-COTECXIN
88	NA	2	1	DUO-COTECXIN
88	NA	2	1	DUO-COTECXIN
88	NA	2	1	
88	NA	2	1	
Ye	FACE SWELLING	2	1	DUO-COTECXIN
88	NA	2	1	
88	NA	2	1	
Ye	PUFFY CHEEKS	2	1	DUO-COTECXIN
88	NA	2	1	DUO-COTECXIN
88	NA	2	1	DUO-COTECXIN
88	NA	2	1	DUO-COTECXIN
88	NA	2	1	DUO-COTECXIN
Ye	WEAKNESS	2	1	DUO-COTECXIN
Ye	WEAKNESS	2	1	DUO-COTECXIN
Ye	WEAKNESS	2	1	DUO-COTECXIN
88	NA	2	1	DUO-COTECXIN
88	NA	2	1	DUO-COTECXIN
88	NA	2	1	DUO-COTECXIN
88	NA	2	1	DUO-COTECXIN
88	NA	2	1	DUO-COTECXIN
Ye	WEAKNESS	2	1	DUO-COTECXIN DIHYDRTEMISININ PIPERAQUINE
88	NA	2	8	NA
88	NA	2	8	NA
88	NA	2	1	DUO-COTECXIN

88	NA	2	1	DUO-COTECXIN
88	NA	2	1	DUO-COTECXIN
88	NA	2	1	DUO-COTECXIN
88	NA	2	1	DUO-COTECXIN
88	NA	2	1	DUO-COTECXIN
Ye	BODY PAINS	2	8	NA
88	NA	2	8	NA
88	NA	2	1	DUO-COTECXIN
88	NA		1	
88	NA	2	1	
88	NA	2	8	NA
88	NA	2	1	DUO-COTECXIN
88	NA	2	8	NA
88	NA	2	1	DUO-COTECXIN
88	NA	2	1	DUO-COTECXIN
88	NA	2	1	DUO-COTECXIN
88	NA	2	8	NA
88	NA	2	8	NA
88	NA	2	1	DUO-COTECXIN
88	NA	2	1	DUO-COTECXIN
88	NA	2	1	DUO-COTECXIN
88	NA	2	8	NA
88	NA	2	1	DUO-COTECXIN
88	NA	2	1	DUO-COTECXIN
88	NA	2	1	DUO-COTECXIN
88	NA	2	1	DUO-COTECXIN
88	NA	2	1	DUO-COTECXIN
88	NA	2	1	DUO-COTECXIN
88	NA	2	1	DUO-COECXIN
88	NA	2	1	DUO-COTECXIN
88	NA	2	1	DUO-COTECXIN
88	NA	2	1	DUO-COTECXIN
88	NA	2	1	DUO-COTECXIN
88	NA	2	1	DUO-COTECXIN
88	NA	2	1	DUO-COTECXIN
88	NA	2	1	DUO-COTECXIN
88	NA	2	1	DUO-COTECXIN
Ye	WEAKNESS	2	1	DUO-COTECXIN
88	NA	2	1	DUO-COTECXIN
88	NA	2	1	DUO-COTECXIN
88	NA	2	1	DUO-COTECXIN
88	NA	2	1	DUO-COTECXIN
No		2	8	NA
No		2	8	NA
88	NA	2	8	NA
88	NA	2	1	DUO-COTECXIN
88	NA	2	1	DUO-COTECXIN

88	NA	2	1	DUO-COTECXIN
88	NA	2	1	DUO-COTECXIN
88	NA	2	1	DUO-COTECXIN
88	NA	2	1	DUO-COTECXIN
88	NA	2	1	DUO-COTECXIN
88	NA	2	1	DUO-COTECXIN
88	NA	2	1	DUO-COTECXIN
88	NA	2	1	DUO-COTECXIN
88	NA	2	1	DUO-COTECXIN
88	NA	2	1	DUO-COTECXIN
88	NA	2	1	DUO-COTECXIN
88	NA	2	1	DUO-COTECXIN
88	NA	2	1	DUO-COTECXIN
88	NA	2	1	DUO-COTECXIN
88	NA	2	1	DUO-COTECXIN
88	NA	2	1	DUO-COTECXIN
88	NA	2	1	DUO-COTECXIN
88	NA	2	1	DUO-COTECXIN
88	NA	2	1	DUO-COTECXIN
Ye	WEAKNESS	2	1	DIHYDROARTEMISININ PIPERAQUINE
88	NA	2	1	DUO-COTECXIN
88	NA	2	1	DUO-COTECXIN
88	NA	2	1	DUO-COTECXIN
88	NA	2	1	DUO-COTECXIN
88	NA	2	1	DUO-COTECXIN
88	NA	2	1	DUO-COTECXIN
88	NA	2	1	DUO-COTECXIN
88	NA	2	1	DUO-COTECXIN
Ye	WEAKNESS	2	1	DUO-COTECXIN
88	NA	2	1	DUO-COTECXIN
88	NA	2	1	DUO-COTECXIN
No		2	1	DUO-COTECXIN
88	NA	2	1	DUO-COTECXIN
88	NA	2	1	DUO-COTECXIN
88	NA	2	1	DUO-COTECXIN
88	NA	2	1	DUO-COTECXIN
88	NA	2	1	DUO-COTECXIN
88	NA	2	1	DUO-COTECXIN
Ye	WEAKNESS, BODY PAINS		1	DUO-COTECXIN
88	NA	2	1	DUO-COTECXIN
88	NA	2	1	DUO-COTECXIN
88	NA	2	1	DUO-COTECXIN
No		2	1	DUO-COTECXIN
88	NA	2	8	NA
88	NA	2	1	DUO-COTECXIN
88	NA	2	1	DUO-COTECXIN
88	NA	2	1	DUO-COTECXIN

88	NA	2	1	DUO-COTECXIN
88	NA	2	1	DUO-COTECXIN
88	NA	2	1	DUO-COTECXIN
No		2	1	DUO-COTECXIN
88	NA	2	8	NA
Ye	WEAKNESS	2	1	DUO-COTECXIN
88	NA	2	1	DUO-COTECXIN
88	NA	2	1	DUO-COTECXIN
88	NA	2	1	DUO-COTECXIN
88	NA	2	1	DUO-COTECXIN
88	NA	2	1	DUO-COTECXIN
88	NA	2	8	NA
88	NA	2	8	NA
88	NA	2	1	DUO-COTECXIN
88	NA	2	1	DUO-COTECXIN
88	NA	2	1	DUO-COTECXIN
88	NA	2	1	DUO-COTECXIN
88	NA	2	1	DUO-COTECXIN
88	NA	2	1	DUO-COTECXIN
88	NA	2	1	DUO-COTECXIN
88	NA	2	1	DUO-COTECXIN
Ye	LOSS OF APPETITE	2	8	NA
88	NA	2	8	NA
88	NA	2	8	NA
88	NA	2	1	DUO-COTECXIN
88	NA	2	1	DUO-COTECXIN
88	NA	2	1	DUO-COTECXIN
88	NA	2	1	DUO-COTECXIN
Ye	WEAKNESS	2	1	DUO-COTECXIN
88	NA	2	1	DUO-COTECXIN
88	NA	2	8	NA
88	NA	2	8	NA
88	NA	2	1	DUO-COTECXIN
88	NA	2	1	DUO-COTECXIN
88	NA	2	1	DUO-COTECXIN
No		2	1	DUO-COTECXIN
88	NA	2	1	DUO-COTECXIN
88	NA	2	1	DUO-COTECXIN
88	NA	2	1	DUO-COTECXIN
88	NA	2	1	DUO-COTECXIN
88	NA	2	1	DUO-COTECXIN
88	NA	2	1	DUO-COTECXIN
88	NA	2	1	DUO-COTECXIN
88	NA	2	1	DUO-COTECXIN
88	NA	2	8	NA
88	NA	2	8	NA
88	NA	2	8	NA

88	NA	2	1	DUO-COTECXIN
Ye	FEVER	2	1	DUO-COTECXIN
88	NA	2	1	DUO-COTECXIN
No	WEAKNESS	2	1	DUO-COTECXIN
88	NA	2	1	DUO-COTECXIN
Ye	WEAKNESS	2	1	DUO-COTECXIN
No	WEAKNESS	2	1	DIHYDROARTEMISININ PIPERAQUINE
88	NA	2	1	DUO-COTECXIN
88	NA	2	1	DUO-COTECXIN
88	NA	2	1	DUO-COTECXIN
88	NA	2	8	NA
88	NA	2	8	NA
88	NA	2	1	DUO-COTECXIN
88	NA	2	1	DUO-COTECXIN
Ye	WEAKNESS	2	1	DUO-COTECXIN
88	NA	2	1	DUO-COTECXIN
88	NA	2	1	DUO-COTECXIN
No		2	1	DUO-COTECXIN
88	NA	2	1	DUO-COTECXIN
88	NA	2	1	DUO-COTECXIN
88	NA	2	1	DUO-COTECXIN
88	NA	2	1	DUO-COTECXIN
88	NA	2	1	DUO-COTECXIN
88	NA	2	8	NA
88	NA	2	1	DUO-COTECXIN
88	NA	2	1	DUO-COTECXIN
88	NA	2	8	NA
88	NA	2	1	DUO-COTECXIN
88	NA	2	1	DUO-COTECXIN
88	NA	2	1	DUO-COTECXIN
88	NA	2	1	DUO-COTECXIN
88	NA	2	1	DUO-COTECXIN
88	NA	2	1	DUO-COTECXIN
88	NA	2	1	DUO-COTECXIN
88	NA	2	1	DUO-COTECXIN
88	NA	2	1	DUO-COTECXIN
88	NA	2	1	DUO-COTECXIN
88	NA	2	1	DUO-COTECXIN
88	NA	2	1	DUO-COTECXIN
88	NA	2	1	DUO-COTECXIN
88	NA	2	1	DUO-COTECXIN
88	NA	2	1	DUO-COTECXIN
88	NA	2	1	DUO-COTECXIN
No		2	1	DUO-COTECXIN
88	NA	2	1	DUO-COTECXIN
88	NA	2	1	DUO-COTECXIN
88	NA	2	1	DUO-COTECXIN

88	NA	2	1	DUO-COTECXIN
88	NA	2	1	DUO-COTECXIN
88	NA	2	1	DUO-COTECXIN
88	NA	2	1	DUO-COTECXIN
88	NA	2	8	NA
88	NA	2	1	DUO-COTECXIN
88	NA	2	1	DUO-COTECXIN
88	NA	2	8	NA
88	NA	2	1	DUO-COTECXIN
88	NA	2	1	DUO-COTECXIN
88	NA	2	1	DUO-COTECXIN
88	NA	2	8	NA
88	NA	2	1	DUO-COTECXIN
88	NA	2	8	NA
88	NA	2	8	NA
88	NA	2	1	DUO-COTECXIN
88	NA	2	8	NA
88	NA	2	1	DUO-COTECXIN
88	NA	2	1	DUO-COTECXIN
No		2	1	DUO-COTECXIN
88		2	8	NA
No		2	1	DUO-COTECXIN
88	NA	2	1	DUO-COTECXIN
88	NA	2	1	DUO-COTECXIN
88	NA	2	1	DUO-COTECXIN
No		2	1	DUO-COTECXIN
88	NA	2	8	NA
88	NA	2	1	DUO-COTECXIN
88	NA	2	1	DUO-COTECXIN
88	NA	2	8	NA
88	NA	2	8	NA
88	NA	2	1	DUO-COTECXIN
88	NA	2	1	DUO-COTECXIN
88	NA	2	1	DUO-COTECXIN
88	NA	2	1	DUO-COTECXIN
88	NA	2	1	DUO-COTECXIN
88	NA	2	1	DUO-COTECXIN
88	NA	2	1	DUO-COTECXIN
88	NA	2	1	DUO-COTECXIN
88	NA	2	1	DUO-COTECXIN
88	NA	2	1	DUO-COTECXIN
88	NA	2	1	DUO-COTECXIN
88	NA	2	1	DUO-COTECXIN
Ye	WEAKNESS	2	1	DUO-COTECXIN
88	NA	2	1	DUO-COTECXIN
88	NA	2	1	DUO-COTECXIN
88	NA	2	1	DUO-COTECXIN

88	NA	2	1	DUO-COTECXIN
88	NA	2	1	DUO-COTECXIN
88	NA	2	1	DUO-COTECXIN
88	NA	2	1	DUO-COTECXIN
88	NA	2	1	DUO-COTECXIN
88	NA	2	1	DUO-COTECXIN
88	NA	2	1	DUO-COTECXIN
88	NA	2	1	DUO-COTECXIN
88	NA	2	1	DUO-COTECXIN
88	NA	2	1	DUO-COTECXIN
88	NA	2	1	DUO-COTECXIN
88	NA	2	8	NA
88	NA	2	1	DUO-COTECXIN
88	NA	2	1	DUO-COTECXIN
88	NA	2	1	DUO-COTECXIN
88	NA	2	8	NA
88	NA	2	1	DUO-COTECXIN
No		2	1	DUO-COTECXIN

q83ant1b	q83ant2	q83ant2a	q83ant2b	q84	q85	q86	q86a	q87	q88	q88a
DIHYDROARTEMISININ PIPERAQUINE	8	NA	NA	0	88			Y	N	NA
DIHYDROARTEMISININ PIPERAQUINE	8	NA	NA	0	88			Y	N	NA
DIHYDROARTEMISIN PIPERAQUINE	8	NA	NA	0	88			N		NA
DIHYDROARTEMISININ PIPERAQUINE	8	NA	NA	0	88			Y	N	NA
DIHYDROARTEMISININ PIPERAQUINE	8	NA	NA	0				Y	N	NA
DIHDROARTEMISININ PIPERAQUINE	8	NA	NA	0				Y	N	NA
DIHDROARTEMISININ PIPERAQUINE	8	NA	NA	1		C	NA	Y	N	NA
	8	NA	NA	0				Y	N	NA
DIHYDROARTEMISININ PIPERAQUINE	8			0				Y	N	NA
DIHYDROARTEMISININ PIPERAQUINE	8	NA	NA	0				Y	P	NA
DIHYDROARTEMISININ/PIPERAQUINE	8			0				Y	N	NA
	8	NA	NA	0	88			Y	N	NA
DIHYDRARTEMISININ-PIPERAQUINE	8	NA	NA	0	88			Y	N	NA
DIHYDRARTEMISININ PIPERAQUINE	8	NA	NA	1		C	NA	Y	N	NA
DIHYDRARTEMISININ PIPERAQUINE	8			0	88			Y	N	NA
DIHYDROARTEMISININ PIPERAQUINE	8	NA	NA	0				Y	P	NA
	8	NA	NA	0				Y	N	NA
DIHYDROARTEMISININM PIPERAQUINE	8	NA	NA	0				Y	P	NA
DIHYDROARTEMISININ/PIPERAQUINE	8	NA	NA	0	88			Y	P	NA
DIHYDROARTEMISININ-PIPERAQUINE	8	NA	NA	0	88			Y	N	NA
DIHYDROARTEMISININ PIPERAQUINE	8	NA	NA	0	88			Y	P	NA
(DIHYDROARTEMISININ PIPERAQUINE)	8	NA	NA	2	88	C	NA	N		NA
DIHYDROARTEMISININ PIPERAQUINE	8	NA	NA	0	88			Y	P	NA
DIHYDROARTEMISININ/PIPERAQUINE	8	NA	NA	0				Y	P	NA
	8	NA	NA	0	88			Y	N	NA
	8	NA	NA	0	88			Y	N	NA
DIHYDROARTEMISININ PIPERAQUINE	8	NA	NA	88	88			Y	P	NA
	8	NA	NA	4		C	NA	Y	N	NA
	8	NA	NA	6	88	C	NA	Y	P	NA
DIHYDROARTEMISININ PIPERAQUINE	8	NA	NA	0				Y	P	NA
DIHYDROARTEMISININ PIPERAQUINE	8	NA	NA	0				Y	P	NA
(DIHYDROATEMISININ PIPERAQUINE)	8	NA	NA	0				Y	P	NA
(DIHYDROARTEMISININ PIPERAQUINE)	8	NA	NA	0	88			Y	P	NA
(DIHYDROARTEMISININ PIPERAQUINE)	8	NA	NA	0				Y	P	NA
(DIHYDROATEMISININ PIPERAQUINE)	8	NA	NA	0				Y	P	NA
(DIHYDROARTEMISININ PIPERAQUINE)	8	NA	NA	0				Y	P	NA
(DIHYDROABTEMISININ PIPERAQUINE)	8	NA	NA	6		C	NA	Y	P	NA
(DIHYDROARTEMISININ PIPERAQUINE)	8	NA	NA	2		C	NA	Y	P	NA
(DIHYDROABTEMISININ PIPERAQUINE)	8	NA	NA	0				Y	P	NA
(DIHYDROARTEMISININ PIPERAQUIN	8	NA	NA	0	88			Y	P	NA
DIHYDROARTEMISININ PIPERAQUINE	8	NA	NA	0				Y	N	NA
DIHYDROARTEMISININ PIPERAQUINE	8	NA	NA	0				Y	P	NA
	8	NA	NA	2	88	C	NA	Y	P	NA
NA	8	NA	NA	88	88			Y	G	NA
NA	8	NA	NA	88	88			Y	G	NA
DIHYDROARTEMISININ PIPERAQUINE	8	NA	NA	0	88			Y	P	NA

DIHYDROARTEMISININ PIPERAQUINE	8	NA	NA	0				Y	P	NA
DIHYDROARTEMISININ PIPERAQUINE	8	NA	NA	0				Y	P	NA
(DIHYDROARTEMISININ PIPERAQUINE)	8	NA	NA	0				Y	P	NA
DIHYDROARTEMISININ PIPERAQUINE	8	NA	NA	0	88			Y	P	NA
DIHYDROARTEMISININ PIPERAQUINE	8	NA	NA	0	88			Y	P	NA
NA	8	NA	NA	88	88			N		NA
NA	8	NA	NA	88	88			Y	G	NA
DIHYDROARTEMISININ PIPERAQUINE	0			0	88			Y	G	NA
	8	NA	NA	0	88			Y	P	NA
	8			0	88			Y	G	NA
NA	8	NA	NA	88	88			Y	G	NA
DIHYDROARTEMISININ PIPERAQUINE	8	NA	NA	0	88			Y	N	NA
NA	8	NA	NA	88	88			Y	N	NA
DIHYDRARTEMISININ PIPREAQUINE	8	NA	NA	0				Y	P	NA
DIHYDROARTEMISININ PIPERAQUINE	8	NA	NA	0	88			Y	G	NA
DIHYDROARTEMISININ PIPERAQUINE	8	NA	NA	0				Y	P	NA
NA	8	NA	NA	88	88			Y	N	NA
NA	8	NA	NA	88	88			Y	G	NA
DIHYDROARTEMISININ PIPERAQUINE	8	NA	NA	0	88			Y	N	NA
DIHYDROARTEMISININ PIPERAQUINE	8	NA	NA	0	88			Y	P	NA
DIHYDROARTEMISININ PIPERAQUINE	8	NA	NA	0				N		NA
NA	8	NA	NA	88	88			Y	N	NA
DIHYDROARTEMISININ PIPERAQUINE	8	NA	NA	2		C	NA	Y	N	NA
DIHYDROARTEMISININ PIPERAQUINE	8	NA	NA	0	88			Y	P	NA
DIHYDROARTEMISIN PIPERAQUINE	8	NA	NA	0	88			Y	P	NA
DIHYDROARTEMISIN PIPERAQUINE	8	NA	NA	0	88			Y	P	NA
DIHYDROARTEMISIN PIPERAQUINE	8	NA	NA	0				Y	P	NA
DIHYDROARTEMISIN PIPERAQUINE	8	NA	NA	0	88			Y	N	NA
DIHYDROARTEMISIN PIPERAQUINE	8	NA	NA	0				Y	N	NA
DIHYDROARTEMISIN PIPERAQUINE	8	NA	NA	0				Y	P	NA
DIHYDROARTEMISIN PIPERAQUINE	8	NA	NA	0				Y	P	NA
DIHYDROARTEMISIN PIPERAQUINE	8	NA	NA	0	88			Y	G	NA
DIHYDROARTEMISIN PIPERAQUINE	8	NA	NA	0				Y	G	NA
DIHYDROARTEMISININ PIPERAQUINE	8	NA	NA	0	88			Y	P	NA
DIHYDROARTEMISININ PIPERAQUINE	8	NA	NA	0	88			Y	P	NA
DIHYDROARTEMISININ PIPERAQUINE	8	NA	NA	0	88			Y	P	NA
DIHYDROARTEMISININ PIPERAQUINE	8	NA	NA	0	88			Y	P	NA
DIHYDROARTEMISININ PIPERAQUINE	8	NA	NA	0	88			Y	P	NA
DIHYDROARTEMISININ PIPERAQUINE	8	NA	NA	0	88			Y	P	NA
DIHYDROARTEMISININ PIPERAQUINE	8	NA	NA	0	88			Y	P	NA
DIHYDROARTEMISININ PIPERAQUINE	8	NA	NA	0	88			Y	P	NA
DIHYDROARTEMISININ PIPERAQUINE	8	NA	NA	0	88			Y	P	NA
NA	8	NA	NA	88	88			Y	P	NA
NA	8	NA	NA	88	88			Y	P	NA
NA	8	NA	NA	88	88			Y	G	NA
DIHYDROARTEMISININ PIPERAQUINE	8	NA	NA	0	88			Y	N	NA
DIHYDROARTEMISININ PIPERAQUINE	8	NA	NA	0	88			Y	N	NA

DIHYDROARTEMISININ PIPERAQUINE	8	NA	NA	0	88			Y	N	NA
DIHYDROARTEMISININ PIPERAQUINE	8	NA	NA	0	88			Y	P	NA
DIHYDROARTEMISININ PIPERAQUINE	8	NA	NA	0	88			Y	P	NA
DIHYDROARTEMISININ PIPERAQUINE	8	NA	NA	9	88	C	NA	Y	P	NA
DIHYDROARTEMISININ PIPERAQUINE	8	NA	NA	0	88			Y	P	NA
DIHYDROARTEMISININ PIPERAQUINE	8	NA	NA	0	88			Y	N	NA
DIHYDROARTEMISININ	8	NA	NA	0	88			Y	G	NA
DIHYDROARTEMISININ PIPERAQUINE	8	NA	NA	0	88			Y	G	NA
DIHYDROARTEMISININ PIPERAQUINE	8	NA	NA	0	88			Y	N	NA
DIHYDROARTEMISININ PIPERAQUINE	8	NA	NA	0	88			Y	G	NA
DIHYDROARTEMISININ PIPERAQUINE	8	NA	NA	0	88			Y	G	NA
DIHYDROARTEMISININ PIPERAQUINE	8	NA	NA	0	88			Y	P	NA
DIHYDROARTEMISININ PIPERAQUINE	8	NA	NA	0	88			Y	P	NA
DIHYDROARTEMISININ PIPERAQUINE	8	NA	NA	0	88			Y	P	NA
DIHYDROARTEMISININ PIPERAQUINE	8	NA	NA	0	88			Y	P	NA
DIHYDROARTEMISININ PIPERAQUINE	8	NA	NA	0	88			Y	P	NA
DIHYDROARTEMISININ PIPERAQUINE	8	NA	NA	0	88			Y	P	NA
DIHYDROARTEMISININ PIPERAQUINE	8	NA	NA	3	88	C	NA	Y	N	NA
DIHYDROARTEMISININ PIPERAQUINE	8	NA	NA	0	88			Y	P	NA
DUO-COTECXIN	8	NA	NA	0	88			Y	P	NA
DIHYDROARTEMISININ PIPERAQUINE	8	NA	NA	0	88			Y	N	NA
DIHYDROARTEMISININ PIPERAQUINE	8	NA	NA	0	88			Y	N	NA
DIHYDROARTEMISININ PIPERAQUINE	8	NA	NA	0	88			Y	N	NA
DIHYDROARTEMISININ PIPERAQUINE	8	NA	NA	0	88			Y	N	NA
DIHYDROARTEMISININ PIPERAQUINE	8	NA	NA	0	88			Y	N	NA
DIHYDROARTEMISININ PIPERAQUINE	8	NA	NA	0	88			Y	N	NA
DIHYDROARTEMISININ PIPERAQUINE	8	NA	NA	0	88			Y	P	NA
DIHYDROARTEMISININ PIPERAQUINE	8	NA	NA	0	88			Y	N	NA
DIHYDROARTEMISININ PIPERAQUINE	8	NA	NA	0	88			Y	P	NA
DIHYDROARTEMISININ PIPERAQUINE	8	NA	NA	0	88			Y	P	NA
DIHYDROARTEMISININ PIPERAQUINE	8	NA	NA	0	88			Y	N	NA
DIHYDROARTEMISININ PIPERAQUINE	8	NA	NA	0	88			Y	P	NA
DIHYDROARTEMISININ PIPERAQUINE	8	NA	NA	0	88			Y	P	NA
DIHYDROARTEMISININ PIPERAQUINE	8	NA	NA	2	88	C	NA	Y	P	NA
DIHYDROARTEMISININ PIPERAQUINE	8	NA	NA	2	88	C	NA	Y	f	NA
DIHYDROARTEMISININ PIPERAQUINE	8	NA	NA	0	88			Y	N	NA
DIHYDROARTEMISININ PIPERAQUINE	8	NA	NA	0	88			Y	N	NA
DIHYDROARTEMISININ PIPERAQUINE	8	NA	NA	0	88			Y	N	NA
DIHYDROARTEMISININ PIPERAQUINE	8	NA	NA	0	88		NA	N		NA
DIHYDROARTEMISININ PIPERAQUINE	8	NA	NA	0	88			Y	P	NA
DIHYDROARTEMISININ PIPERAQUINE	8	NA	NA	0	88			Y	P	NA
DIHYDROARTEMISININ PIPERAQUINE	8	NA	NA	0	88			Y	P	NA
DIHYDROARTEMISININ PIPERAQUINE	8	NA	NA	0	88			Y	P	NA
NA	8	NA	NA	88	88			Y	N	NA
DIHYDROARTEMISININ PIPERAQUINE	8	NA	NA	0	88			Y	N	NA
DIHYDROARTEMISININ PIPERAQUINE	8	NA	NA	0	88			Y	N	NA
DIHYDROARTEMISININ PIPERAQUINE	8	NA	NA	3	88	C	NA	Y	f	NA

DIHYDROARTEMISININ PIPERAQUINE	8	NA	NA	1	88	C	NA	Y	N	NA
DIHYDROARTEMISININ PIPERAQUINE	8	NA	NA	2	88	C	NA	Y	N	NA
DIHYDROARTEMISININ PIPERAQUINE	8	NA	NA	2	88	C	NA	Y	N	NA
DIHYDROARTEMISININ PIPERAQUINE	8	NA	NA	0	88			Y	P	NA
NA	8	NA	NA	88	88			Y	N	NA
DIHYDROARTEMISININ PIPERAQUINE	8	NA	NA	0	88			Y	P	NA
DIHYDROARTEMISININ PIPERAQUINE	8	NA	NA	0	88			Y	P	NA
DIHYDROARTEMISININ PIPERAQUINE	8	NA	NA	0	88			Y	N	NA
DIHYDROARTEMISININ PIPERAQUINE	8	NA	NA	0	88			Y	N	NA
DIHYDROARTEMISININ PIPERAQUINE	8	NA	NA	3	88	C	NA	Y	N	NA
DIHYDROARTEMISININ PIPERAQUINE	8	NA	NA	2	88	C	NA	Y	N	NA
NA	8	NA	NA	88	88			Y	N	NA
NA	8	NA	NA	88	88			Y	N	NA
DIHYDROARTEMISININ PIPERAQUINE	8	NA	NA	0	88			Y	P	NA
DIHYDROARTEMISININ PIPERAQUINE	8	NA	NA	0	88			Y	P	NA
DIHYDROARTEMISININ PIPERAQUINE	8	NA	NA	0	88			Y	P	NA
DIHYDROARTEMISININ PIPERAQUINE	8	NA	NA	0	88			Y	P	NA
DIHYDROARTEMISININ PIPERAQUINE	8	NA	NA	0	88			Y	P	NA
DIHYDROARTEMISININ PIPERAQUINE	8	NA	NA	0	88			Y	P	NA
DIHYDROARTEMISININ PIPERAQUINE	8	NA	NA	0	88			Y	P	NA
DIHYDROARTEMISININ PIPERAQUINE	8	NA	NA	0	88			Y	P	NA
NA	8	NA	NA	88	88			N		NA
NA	8	NA	NA	88	88			Y	N	NA
NA	8	NA	NA	88	88			Y	N	NA
DIHYDROARTEMISININ PIPERAQUINE	8	NA	NA	0	88			Y	N	NA
DIHYDROARTEMISININ PIPERAQUINE	8	NA	NA	0	88			Y	N	NA
DIHYDROARTEMISININ PIPERAQUINE	8	NA	NA	0	88			Y	N	NA
DIHYDROARTEMISININ PIPERAQUINE	8	NA	NA	8	88	D		Y	P	NA
DIHYDROARTEMISININ PIPERAQUINE	8	NA	NA	2	88	C	NA	Y	f	NA
DIHYDROARTEMISININ PIPERAQUINE	8	NA	NA	1	88	C	NA	Y	f	NA
NA	8	NA	NA	88	88			Y	N	NA
NA	8	NA	NA	88	88			Y	N	NA
DIHYDROARTEMISININ PIPERAQUINE	8	NA	NA	0	88			Y	P	NA
DIHYDROARTEMISININ PIPERAQUINE	8	NA	NA	0	88			Y	N	NA
DIHYDROARTEMISININ PIPERAQUINE	8	NA	NA	0	88			Y	N	NA
DIHYDROARTEMISININ PIPERAQUINE	8	NA	NA	0	88			Y	P	NA
DIHYDROARTEMISININ PIPERAQUINE	8	NA	NA	0	88			Y	N	NA
DIHYDROARTEMISININ PIPERAQUINE	8	NA	NA	0	88			Y	P	NA
DIHYDROARTEMISININ PIPERAQUINE	8	NA	NA	0	88			Y	N	NA
DIHYDROARTEMISININ PIPERAQUINE	8	NA	NA	0	88			Y	N	NA
DIHYDROARTEMISININ PIPERAQUINE	8	NA	NA	0	88			Y	P	NA
DIHYDROARTEMISININ PIPERAQUINE	8	NA	NA	0	88			Y	N	NA
DIHYDROARTEMISININ PIPERAQUINE	8	NA	NA	0	88			Y	P	NA
DIHYDROARTEMISININ PIPERAQUINE	8	NA	NA	2	88	S		N		NA
NA	8	NA	NA	88	88			Y	N	NA
NA	8	NA	NA	88	88			Y	N	NA
NA	8	NA	NA	88	88			Y	N	NA

DIHYDROARTEMISININ PIPERAQUINE	8	NA	NA	0	88			Y	N	NA
DIHYDROARTEMISININ PIPERAQUINE	8	NA	NA	3	88	C		N		NA
DIHYDROARTEMISININ PIPERAQUINE	8	NA	NA	0	88			Y	N	NA
DIHYDROARTEMISININ PIPERAQUINE	8	NA	NA	0	88			Y	P	NA
DIHYDROARTEMISININ PIPERAQUINE	8	NA	NA	0	88			Y	P	NA
DIHYDROARTEMISININ PIPERAQUINE	8	NA	NA	0	88			Y	P	NA
	8	NA	NA	0	88			Y	P	NA
DIHYDROARTEMISININ PIPERAQUINE	8	NA	NA	0	88			Y	P	NA
DIHYDROARTEMISININ PIPERAQUINE	8	NA	NA	0	88			Y	P	NA
DIHYDROARTEMISININ PIPERAQUINE	8	NA	NA	0	88			Y	N	NA
NA	8	NA	NA	88	88			Y	N	NA
NA	8	NA	NA	88	88			Y	N	NA
DIHYDROARTEMISININ PIPERAQUINE	8	NA	NA	2		C	NA	Y	f	NA
DIHYDROARTEMISININ PIPERAQUINE	8	NA	NA	0	88			Y	P	NA
DIHYDROARTEMISININ PIPERAQUINE	8	NA	NA	0	88			Y	P	NA
DIHYDROARTEMISININ PIPERAQUINE	8	NA	NA	0	88			Y	N	NA
DIHYDROARTEMISININ PIPERAQUINE	8	NA	NA	0	88			Y	N	NA
DIHYDROARTEMISININ PIPERAQUINE	8	NA	NA	0	88			Y	P	NA
DIHYDROARTEMISININ PIPERAQUINE	8	NA	NA	0	88			Y	P	NA
DIHYDROARTEMISININ PIPERAQUINE	8	NA	NA	0	88			Y	P	NA
DIHYDROARTEMISININ PIPERAQUINE	8	NA	NA	3	88	D		Y	P	NA
DIHYDROARTEMISININ PIPERAQUINE	8	NA	NA	0	88			Y	P	NA
DIHYDROARTEMISININ PIPERAQUINE	8	NA	NA	0	88			Y	P	NA
NA	8	NA	NA	88	88			Y	N	NA
DIHYDROARTEMISININ PIPERAQUINE	8	NA	NA	0	88			Y	N	NA
DIHYDROARTEMISININ PIPERAQUINE	8	NA	NA	0	88			Y	N	NA
NA	8	NA	NA	88	88			Y	N	NA
DIHYDROARTEMISININ PIPERAQUINE	8	NA	NA	0	88			Y	N	NA
DIHYDROARTEMISININ PIPERAQUINE	8	NA	NA	2	88	C	NA	Y	P	NA
DIHYDROARTEMISININ PIPERAQUINE	8	NA	NA	0	88			Y	P	NA
DIHYDROARTEMISININ PIPERAQUINE	8	NA	NA	0	88			Y	P	NA
DIHYDROARTEMISININ PIPERAQUINE	8	NA	NA	0	88			Y	P	NA
DIHYDROARTEMISININ PIPERAQUINE	8	NA	NA	0	88			Y	P	NA
DIHYDROARTEMISININ PIPERAQUINE	8	NA	NA	0	88			Y	N	NA
DIHYDROARTEMISININ PIPERAQUINE	8	NA	NA	0	88			Y	N	NA
DIHYDROARTEMISININ PIPERAQUINE	8	NA	NA	3	88	C	NA	Y	P	NA
DIHYDROARTEMISININ PIPERAQUINE	8	NA	NA	0	88			Y	N	NA
DIHYDROARTEMISININ PIPERAQUINE	8	NA	NA	0	88			Y	N	NA
DIHYDROARTEMISININ PIPERAQUINE	8	NA	NA	0	88			Y	N	NA
DIHYDROARTEMISININ PIPERAQUINE	8	NA	NA	0	88			Y	N	NA
DIHYDROARTEMISININ PIPERAQUINE	8	NA	NA	0	88			Y	P	NA
DIHYDROARTEMISININ PIPERAQUINE	8	NA	NA	0	88			Y	P	NA
DIHYDROARTEMISININ PIPERAQUINE	8	NA	NA	0	88			Y	P	NA
DIHYDROARTEMISININ PIPERAQUINE	8	NA	NA	2	88	D		Y	P	NA
DIHYDROARTEMISININ PIPERAQUINE	8	NA	NA	0	88			Y	N	NA
DIHYDROARTEMISININ PIPERAQUINE	8	NA	NA	0	88			Y	P	NA
DIHYDROARTEMISININ PIPERAQUINE	8	NA	NA	0	88			Y	N	NA

DIHYDROARTEMISININ PIPERAQUINE	8	NA	NA	0	88			Y	N	NA
DIHYDROARTEMISININ PIPERAQUINE	8	NA	NA	0	88			Y	P	NA
DIHYDROARTEMISININ PIPERAQUINE	8	NA	NA	0	88			Y	P	NA
DIHYDROARTEMISININ PIPERAQUINE	8	NA	NA	0	88			Y	N	NA
NA	8	NA	NA	88	88			Y	N	NA
DIHYDROARTEMISININ PIPERAQUINE	8	NA	NA	0	88			Y	N	NA
DIHYDROARTEMISININ PIPERAQUINE	8	NA	NA	2	88	C	NA	Y	P	NA
NA	8	NA	NA	88	88			Y	N	NA
DIHYDROARTEMISININ PIPERAQUINE	8	NA	NA	2		C	NA	Y	P	NA
DIHYDROARTEMISININ PIPERAQUINE	8	NA	NA	0	88			Y	P	NA
DIHYDROARTEMISININ PIPERAQUINE	8	NA	NA	0	88			Y	N	NA
NA	8	NA	NA	88	88			Y	N	NA
DIHYDROARTEMISININ PIPERAQUINE	8	NA	NA	0	88			Y	P	NA
NA	8	NA	NA	88	88			Y	N	NA
NA	8	NA	NA	88	88			Y	N	NA
	8	NA	NA	0	88			Y	N	NA
	8	NA	NA	88	88			Y	N	NA
DIHYDROARTEMISININ PIPERAQUINE	8	NA	NA	2	88	C	NA	Y	P	NA
DIHYDROARTEMISININ PIPERAQUINE	8	NA	NA	0	88			Y	N	NA
DIHYDROARTEMISININ PIPERAQUINE	8	NA	NA	0	88			Y	P	NA
NA	8	NA	NA	88	88			Y	N	NA
DIHYDROARTEMISININ PIPERAQUINE	8	NA	NA	0	88			Y	N	NA
DIHYDROARTEMISININ PIPERAQUINE	8	NA	NA	0	88			Y	P	NA
DIHYDROARTEMISININ PIPERAQUINE	8	NA	NA	0	88			Y	P	NA
DIHYDROARTEMISININ PIPERAQUINE	8	NA	NA	0	88			Y	P	NA
DIHYDROARTEMISININ PIPERAQUINE	8	NA	NA	2		C	NA	Y	P	NA
NA	8	NA	NA	88	88			Y	N	NA
DIHYDROARTEMISININ PIPERAQUINE	8	NA	NA	0	88			Y	N	NA
DIHYDROARTEMISININ PIPERAQUINE	8	NA	NA	2	88	C	NA	Y	P	NA
NA	8	NA	NA	88	88			N		NA
NA	8	NA	NA	88	88			Y	N	NA
DIHYDROARTEMISININ PIPERAQUINE	8	NA	NA	0	88			Y	P	NA
DIHYDROARTEMISININ PIPERAQUINE	8	NA	NA	0	88			Y	P	NA
DIHYDROARTEMISININ PIPERAQUINE	8	NA	NA	0	88			Y	P	NA
DIHYDROARTEMISININ PIPERAQUINE	8	NA	NA	0	88			Y	P	NA
DIHYDROARTEMISININ PIPERAQUINE	8	NA	NA	0	88			Y	N	NA
DIHYDROARTEMISININ PIPERAQUINE	8	NA	NA	2	88	C	NA	Y	N	NA
DIHYDROARTEMISININ PIPERAQUINE	8	NA	NA	0	88			Y	N	NA
DIHYDROARTEMISININ PIPERAQUINE	8	NA	NA	0	88			Y	N	NA
DIHYDROARTEMISININ PIPERAQUINE	8	NA	NA	0	88			Y	N	NA
	8	NA	NA	0	88			Y	N	NA
DIHYDROARTEMISININ PIPERAQUINE	8	NA	NA	0	88			Y	P	NA
DIHYDROARTEMISININ PIPERAQUINE	8	NA	NA	0	88			Y	P	NA
DIHYDROARTEMISININ PIPERAQUINE	8	NA	NA	0	88			Y	P	NA
DIHYDROARTEMISININ PIPERAQUINE	8	NA	NA	0	88			Y	P	NA
DIHYDROARTEMISININ PIPERAQUINE	8	NA	NA	0	88			Y	P	NA
DIHYDROARTEMISININ PIPERAQUINE	8	NA	NA	0	88			Y	P	NA

DIHYDROARTEMISININ PIPERAQUINE	8	NA	NA	0	88			Y	P	NA
DIHYDROARTEMISININ PIPERAQUINE	8	NA	NA	0	88			Y	P	NA
DIHYDROARTEMISININ PIPERAQUINE	8	NA	NA	0	88			Y	P	NA
DIHYDROARTEMISININ PIPERAQUINE	8	NA	NA	0	88			Y	N	NA
DIHYDROARTEMISININ PIPERAQUINE	8	NA	NA	0	88			Y	N	NA
DIHYDROARTEMISININ PIPERAQUINE	8	NA	NA	2	88	C	NA	Y	f	NA
DIHYDROARTEMISININ PIPERAQUINE	8	NA	NA	0	88			Y	N	NA
DIHYDROARTEMISININ PIPERAQUINE	8	NA	NA	0	88			Y	N	NA
DIHYDROARTEMISININ PIPERAQUINE	8	NA	NA	0	88			Y	N	NA
DIHYDROARTEMISININ PIPERAQUINE	8	NA	NA	0	88			Y	N	NA
DIHYDROARTEMISININ PIPERAQUINE	8	NA	NA	1	88	C	NA	Y	G	NA
NA	8	NA	NA	88	88			Y	N	NA
DIHYDROARTEMISININ PIPERAQUINE	8	NA	NA	2	88	C	NA	Y	P	NA
DIHYDROARTEMISININ PIPERAQUINE	8	NA	NA	0	88			Y	N	NA
DIHYDROARTEMISININ PIPERAQUINE	8	NA	NA	0	88			Y	N	NA
NA	8	NA	NA	88	88			Y	N	NA
DIHYDROARTEMISININ PIPERAQUINE	8	NA	NA	0	88			Y	P	NA
DIHYDROARTEMISININ PIPERAQUINE	8	NA	NA	0	88			Y	P	NA

q89	q89a	inttime	inttime1	sdate	rdt	asexpf	sexpf
	NA	7	50	20-Oct-14	P	P	N
	NA	9	20	20-Oct-14	P	N	N
s	NA	7	11	20-Oct-14	P	P	N
	NA	8	30	20-Oct-14	P	N	N
	NA	10	23	20-Oct-14	P	N	N
	NA	8	15	21-Oct-14	P	N	N
	NA	8	38	21-Oct-14	P	N	N
	NA	10	10	21-Oct-14	P	N	N
	NA	7	50	21-Oct-14	P	N	N
	NA	7	9	21-Oct-14	P	N	N
	NA	7	53	28-Oct-14	P	N	N
	NA	9	25	28-Oct-14	P	P	N
	NA	8	44	28-Oct-14	P	P	N
	NA	8	25	3-Nov-14	P	P	N
	NA	7	5	3-Nov-14	P	P	N
	NA	8	2	3-Nov-14	P	N	N
	NA	8	55	3-Nov-14	P	N	N
	NA	7	38	4-Nov-14	P	P	N
	NA	7	55	4-Nov-14	P	N	N
	NA	8	10	4-Nov-14	P	P	N
	NA	8	26	4-Nov-14	P	P	N
I	IS GOOD BUT NEED TO TRY ANOTHER DRUG	6	52	4-Nov-14	P	P	N
	NA	8	57	4-Nov-14	P	N	N
	NA	9	15	4-Nov-14	P	P	N
	NA	8	2	10-Nov-14	P	P	N
	NA	8	15	10-Nov-14	P	P	N
	NA	8	50	10-Nov-14	P	P	N
	NA	7	22	10-Nov-14	P	P	N
B	NA	6	59	10-Nov-14	P	P	N
	NA	9	14	11-Dec-14	P	N	N
	NA	8	30	11-Nov-14	P	P	N
	NA	7	23	11-Nov-14	P	P	N
	NA	8	37	11-Nov-14	P	P	N
	NA	7	26	17-Nov-14	P	P	N
	NA	8	5	17-Nov-14	P	N	N
	NA	9	31	17-Nov-14	P	N	N
	NA	8	40	17-Nov-14	P	N	N
	NA	7	55	24-Nov-14	P	N	N
	NA	7	30	24-Nov-14	P	N	N
	NA	7	16	28-Nov-14	P	P	N
	NA	7	41	2-Dec-14	P	N	N
	NA	8	21	8-Dec-14	P	P	N
I	NA	9	40	9-Dec-14	P	N	N
	NA	7	25	15-Dec-14	P	P	N
	NA	11	40	16-Dec-14	P	P	N
	NA	8	48	17-Dec-14	P	P	N

	NA	6	44	18-Dec-14	P	N	N
	NA	7	15	18-Dec-14	P	N	N
	NA	7	46	22-Dec-14	P	P	N
	NA	8	15	22-Dec-14	P	N	P
	NA	7	10	22-Dec-14	P	N	N
t	NA	9	10	22-Dec-14	P	N	N
	NA	8	2	22-Dec-14	P	P	N
	NA	8	15	22-Dec-14	P	P	N
	NA	10	12	23-Dec-14	P	P	N
	NA	7	57	23-Dec-14	P	N	N
	NA	8	17	23-Dec-14	P	N	N
	NA	8	8	29-Dec-14		P	N
	NA	8	54	29-Dec-14	P	N	N
	NA	9	0	30-Dec-14	P	N	N
	NA	9	25	30-Dec-14	P	N	N
	NA	9	30	30-Dec-14	P	N	N
	NA	9	47	5-Jan-15	P	N	N
	NA	9	16	5-Jan-15	P	N	N
	NA	8	21	5-Jan-15	P	N	N
	NA	8	25	6-Jan-15	P	P	N
l	NA			6-Jan-15	P	N	N
	NA	8	55	6-Jan-15	P	P	N
	NA	9	21	12-Jan-15	P	P	N
	NA	9	10	12-Jan-15	P	P	N
	NA	9	25	12-Jan-15	P	P	N
	NA			20-Jan-15	P	N	N
	NA	8	35	26-Jan-15	P	P	N
	NA	7	5	26-Jan-15	P	P	N
	NA	7	28	26-Jan-15	P	P	N
	NA	10	56	2-Feb-15	P		
	NA	8	45	2-Feb-15	P		
	NA	7	24	20-Jan-15	P	P	N
	NA	11	13	16-Dec-14	P	P	N
	NA	6	42	14-Sep-15	P	P	N
	NA	6	21	14-Sep-15	P	P	N
	NA	7	8	14-Sep-15	P	P	N
	NA	8	41	14-Sep-15	P	N	N
	NA	7	50	8-Sep-15	P	P	N
	NA	6	45	8-Sep-15	P	P	N
	NA	8	15	8-Sep-15	P	P	N
	NA	7	30	8-Sep-15	P	P	P
	NA	7	11	8-Sep-15	P	P	N
	NA	8	50	8-Sep-15	P	N	N
	NA	9	0	8-Sep-15	P	N	N
	NA	6	50	8-Sep-15	P	P	N
	NA	7	41	8-Sep-15	P	N	N
	NA	7	29	8-Sep-15	P	N	N

	NA	8	1	7-Sep-15	P	P	N
	NA	7	42	7-Sep-15	P	P	N
	NA	9	27	7-Sep-15	P	N	N
	NA	10	38	7-Sep-15	P	N	N
	NA	9	35	3-Nov-15	P	P	N
	NA	7	15	3-Nov-15	P	N	N
	NA	7	58	3-Nov-15	P	N	N
	NA	8	16	3-Nov-15	P	P	N
	NA	6	45	3-Nov-15	P	P	N
	NA	7	40	3-Nov-15	P	P	N
	NA	7	27	3-Nov-15	P	P	N
	NA	6	15	3-Nov-15	P	P	N
	NA	10	17	3-Nov-15	P	P	N
	NA	9	7	3-Nov-15	P	P	N
	NA	8	34	3-Nov-15	P	N	N
	NA	7	30	3-Nov-15	P	P	N
	NA	6	42	3-Nov-15	P	P	N
	NA	11	17	2-Nov-15	P	P	N
	NA	8	53	2-Nov-15	P	P	N
	NA	9	35	2-Nov-15	P	P	N
	NA	7	53	2-Nov-15	P	P	N
	NA	6	45	2-Nov-15	P	P	N
	NA	7	30	2-Nov-15	P	P	N
	NA	8	17	2-Nov-15	P	P	N
	NA	8	43	2-Nov-15	P	P	N
	NA	7	16	2-Nov-15	P	P	N
	NA	6	33	2-Nov-15	P	P	N
	NA	7	25	2-Nov-15	P	P	N
	NA	7	3	2-Nov-15	P	P	N
	NA	8	42	27-Oct-15	P	P	N
	NA	8	7	27-Oct-15	P	P	N
	NA	8	53	7-Sep-15	P	P	N
	NA	7	18	7-Sep-15	P	P	N
	NA	8	35	7-Sep-15	P	N	N
	NA	6	55	7-Sep-15	P	P	N
	NA	7	55	7-Sep-15	P	P	N
	NA	8	15	7-Sep-15	P	P	N
	NA	7	21	7-Sep-15	P	P	N
I	NA	6	41	1-Sep-15	P	P	N
	NA	8	43	1-Sep-15	P	P	N
	NA	7	8	1-Sep-15	P	P	N
	NA	9	39	1-Sep-15	P	P	N
	NA	7	36	1-Sep-15	P	P	N
	NA	7	0	1-Sep-15	P	P	N
	NA	8	13	1-Sep-15	P	P	N
	NA	6	39	1-Sep-15	P	P	N
	NA	8	12	31-Aug-13	P	P	N

	NA	7	44	31-Aug-15	P	P	N
	NA	7	12	31-Aug-15	P	P	N
	NA	6	39	31-Aug-15	P	N	N
	NA	8	30	31-Aug-15	P	P	N
	NA	7	54	1-Sep-15	P	N	N
	NA	7	16	20-Oct-15	P	P	N
	NA	8	24	19-Oct-15	P	P	N
	NA	7	16	19-Oct-15	P	P	N
	NA	9	5	19-Oct-15	P	P	P
	NA	8	23	19-Oct-15	P	P	N
	NA	8	42	19-Oct-15	P	N	N
	NA	6	52	19-Oct-15	P	P	N
	NA	6	37	19-Oct-15	P	P	N
	NA	7	50	19-Oct-15	P	P	N
	NA	2	39	19-Oct-15	P	P	N
	NA	7	17	19-Oct-15	P	P	N
	NA	6	47	19-Oct-15	P	P	N
	NA	10	19	13-Oct-15	P	P	N
	NA			13-Oct-15	P	P	N
	NA	1	20	13-Oct-15	P	P	N
	NA	1	38	13-Oct-15	P	P	N
l	NA	7	15	13-Oct-15	P	P	N
	NA	7	36	13-Oct-15	P	P	N
	NA	8	15	13-Oct-15	P	P	N
	NA	6	55	13-Oct-15	P	P	N
	NA	8	40	13-Oct-15	P	N	N
	NA	7	53	12-Oct-15	P	P	N
	NA	6	56	12-Oct-15	P	P	N
	NA	7	20	12-Oct-15	P	N	N
	NA	8	22	12-Oct-15	P	P	N
	NA	7	20	12-Oct-15	P	P	P
	NA	7	38	12-Oct-15	P	P	N
	NA	7	44	12-Oct-15	P	P	N
	NA	7	3	12-Oct-15	P	P	N
	NA	6	39	12-Oct-15	P	P	N
	NA	6	34	28-Sep-15	P	P	N
	NA	7	43	22-Sep-15	P	N	N
	NA	6	48	22-Sep-15	P	P	N
	NA	6	28	22-Sep-15	P	N	N
	NA	6	49	22-Sep-15	P	P	N
	NA	7	15	22-Sep-15	P	P	N
	NA	8	27	22-Sep-15	P	P	N
	NA	8	25	22-Sep-15	P	P	N
s	BECAUSE CHILD DONT WANT TO TAKE TABS	7	45	22-Sep-15	P	P	N
	NA	7	20	22-Sep-15	P	P	N
	NA	8	57	15-Sep-15	P	N	N
	NA	7	53	15-Sep-15	P	P	N

	NA	7	41	15-Sep-15	P	N	N
s	NA	8	42	15-Sep-15	P	P	N
	NA	8	21	15-Sep-15	P	P	N
	NA	10	13	15-Sep-15	P	N	N
	NA	8	43	15-Sep-15	P	P	N
	NA	7	15	15-Sep-15	P	N	N
	NA	9	40	15-Sep-15	P	N	N
	NA	9	26	15-Sep-15	P	P	P
	NA	7	51	15-Sep-15	P	P	N
	NA	6	55	14-Sep-15	P	P	N
	NA	11	50	14-Sep-15	P	P	N
	NA	7	32	14-Sep-15	P	P	N
	NA	7	19	14-Sep-15	P	P	N
	NA	7	43	14-Sep-15	P	P	N
	NA	6	32	22-Sep-15	P	P	N
	NA	8	48	31-Aug-15	P	P	N
	NA	8	39	31-Aug-15	P	P	N
	NA	8	57	31-Aug-15	P	P	N
	NA	8	5	31-Aug-15	P	P	N
	NA	8	22	31-Aug-15	P	P	N
	NA	8	45	25-Aug-15	P	P	N
	NA	7	15	25-Aug-15	P	P	N
	NA	9	13	25-Aug-15	P	P	N
	NA	7	28	25-Aug-15	P	N	N
	NA	8	25	25-Aug-15	P	N	N
	NA	7	47	25-Aug-15	P	P	N
	NA	7	6	25-Aug-15	P	P	N
	NA	10	48	25-Aug-15	P	P	N
	NA	7	53	25-Aug-15	P	P	N
	NA	9	45	25-Aug-15	P	P	N
	NA	8	28	24-Aug-15	P	N	N
	NA	9	38	24-Aug-15	P	N	P
	NA	8	55	24-Aug-15	P	P	N
	NA	7	38	24-Aug-15	P	P	N
	NA	9	10	24-Aug-15	P	P	N
	NA	7	5	24-Aug-15	P	P	N
	NA	8	33	24-Aug-15	P	P	N
	NA	10	3	24-Aug-15	P	N	N
	NA	8	7	24-Aug-15	P	P	
	NA	7	13	24-Aug-15	P	P	N
	NA	8	46	24-Aug-15	P	P	N
	NA	8	2	24-Aug-15	P	N	N
	NA	7	32	24-Aug-15	P	P	N
	NA	8	55	18-Aug-15	P	P	N
	NA	9	25	18-Aug-15	P	P	N
	NA	7	11	17-Aug-15	P	P	N
	NA	8	36	17-Aug-15	P	N	N

	NA	7	44	17-Aug-15	P	P	N
	NA	8	6	17-Aug-15	P	N	N
	NA	7	54	17-Aug-15	P	P	N
	NA	7	8	17-Aug-15	P	P	N
	NA	7	35	6-Oct-15	P	N	N
	NA	8	38	6-Oct-15	P	P	N
s	NA	6	48	6-Oct-15	P	P	N
	NA	7	50	6-Oct-15	P	P	N
	NA	8	9	6-Oct-15	P	P	N
	NA	12	45	5-Oct-15	P	P	N
	NA	6	25	6-Oct-15	P	P	N
	NA	7	4	5-Oct-15	P	P	N
	NA	7	22	5-Oct-15	P	P	N
	NA	8	4	5-Oct-15	P	P	N
	NA	8	20	5-Oct-15	P	N	N
	NA	8	53	5-Oct-15	P	P	N
	NA	7	25	5-Oct-15	P	P	N
	NA	8	0	29-Sep-15	P	P	N
	NA	6	13	29-Sep-15	P	N	N
	NA	7	5	29-Sep-15	P	P	N
	NA	7	18	29-Sep-15	P	P	N
	NA	7	37	29-Sep-15	P	P	N
	NA	7	22	29-Sep-15	P	P	N
	NA	8	0	29-Sep-15	P	P	N
	NA	6	43	29-Sep-15	P	N	N
s	NA	6	52	29-Sep-15	P	P	P
	NA	7	27	28-Sep-15	P	P	N
	NA	7	9	28-Sep-15	P	N	N
	NA	7	43	28-Sep-15	P	N	N
l	NA	8	25	28-Sep-15	P	N	N
	NA	6	45	28-Sep-15	P	P	N
	NA	8	14	28-Sep-15	P	P	N
	NA	7	39	28-Sep-15	P	P	N
	NA	6	59	28-Sep-15	P	P	N
	NA	8	50	28-Sep-15	P	P	N
	NA	6	42	27-Oct-15	P	N	N
	NA	8	44	27-Oct-15	P	P	N
	NA	6	36	27-Oct-15	P	P	N
	NA	7	20	27-Oct-15	P	P	N
	NA	9	5	27-Oct-15	P	P	N
	NA	7	13	27-Oct-15	P	P	N
	NA	6	26	27-Oct-15	P	P	N
	NA	12	25	27-Oct-15	P	P	N
	NA	7	21	27-Oct-15	P	P	N
	NA	1	43	26-Oct-15	P	P	N
	NA	8	10	26-Oct-15	P	P	N
	NA	7	14	26-Oct-15	P	N	N

NA	7	36	26-Oct-15	P	N	N
NA	6	42	26-Oct-15	P	P	N
NA	9	5	26-Oct-15	P	P	N
NA	7	20	26-Oct-15	P	P	N
NA	9	0	26-Oct-15	P	N	N
NA	8	12	26-Oct-15	P	P	N
NA	7	35	26-Oct-15	P	P	N
NA	6	59	26-Oct-15	P	P	N
NA	6	30	26-Oct-15	P	P	N
NA	8	28	26-Oct-15	P	P	N
NA	9	23	20-Oct-15	P	P	N
NA	6	52	20-Oct-15	P	P	N
NA	7	13	20-Oct-10	P	P	N
NA	6	17	20-Oct-15	P	P	N
NA	7	41	20-Oct-15	P	P	N
NA	8	26	20-Oct-15	P	N	N
NA	10	22	20-Oct-15	P	P	N
NA	7	23	20-Oct-15	P	N	N

asexpm	sexpm	asexoval	sexoval	ascount0	sexcount0	q2d28	q18d28	q19ad28	q20d28	q21ad28
N	N	N	N	9	0	17-Nov-14	1	NA	20-Oct-14	7
N	N	N	N	0	0	17-Nov-14	1	NA	20-Oct-14	11
N	N	N	N	20	0	17-Nov-14	1	NA	20-Oct-14	7
N	N	N	N	0	0	17-Nov-14	1	NA	20-Oct-14	8
N	N	N	N	0	0	17-Nov-14	1	NA	20-Oct-14	10
N	N	N	N	0	0	18-Nov-14	1	NA	21-Oct-14	7
N	N	N	N	0	0	18-Nov-14	1	NA	21-Oct-14	8
N	N	N	N	0	0	24-Oct-14	1	NA	21-Oct-14	9
N	N	N	N	0	0	24-Oct-14	1	NA	21-Oct-14	7
N	N	N	N	0	0	18-Nov-14	1	NA	21-Oct-14	6
N	N	N	N	0	0	25-Nov-14	1	NA	28-Oct-14	3
N	N	N	N	347	0	25-Nov-14	1	NA	28-Oct-14	3
N	N	N	N	753	0	25-Nov-14	1	NA	28-Oct-14	2
N	N	N	N	1836	0	1-Dec-14	1	NA	3-Nov-14	6
N	N	N	N	1378	0	1-Dec-14	1	NA	3-Nov-14	7
N	N	N	N	0	0	1-Dec-14	3	NA	3-Nov-14	
N	N	N	N	0	0	1-Dec-14	1	NA	3-Nov-14	6
N	N	N	N	7	0	2-Dec-14	1	NA	4-Nov-14	6
N	N	N	N	0	0	2-Dec-14	1	NA	4-Nov-14	6
N	N	N	N	694	0	2-Dec-14	1	NA	4-Nov-14	7
N	N	N	N	24	0	2-Dec-14	1	NA	4-Nov-14	6
N	N	N	N	579	0	2-Dec-14	1	NA	4-Nov-14	8
N	N	N	N	0	0	2-Dec-14	1	NA	4-Nov-14	7
N	N	N	N	10	0	2-Dec-14	1	NA	4-Nov-14	7
N	N	N	N	478	0	8-Dec-14	1	NA	10-Nov-14	6
N	N	N	N	11	0	8-Dec-14	1	NA	10-Nov-14	7
N	N	N	N	223	0	3-Dec-14	1	NA	10-Nov-14	8
N	N	N	N	482	0	8-Dec-14	1	NA	10-Nov-14	6
N	N	N	N	40	0	8-Dec-14	1	NA	10-Nov-14	6
N	N	N	N	0	0	9-Dec-14	1	NA	11-Nov-14	2
N	N	N	N	7	0	9-Dec-14	1	NA	11-Nov-14	6
N	N	N	N	463	0	9-Dec-14	1	NA	11-Nov-14	7
N	N	N	N	1275	0	9-Dec-14	1	NA	11-Nov-14	7
N	N	N	N	10	0	15-Dec-14	1	NA	17-Nov-14	2
N	N	N	N	0	0	15-Dec-14	1	NA	17-Nov-14	2
N	N	N	N	0	0	15-Dec-14	1	NA	17-Nov-14	7
N	N	N	N	0	0	15-Dec-14	1	NA	17-Nov-14	7
N	N	N	N	0	0	22-Dec-14	1	NA	24-Nov-14	7
N	N	N	N	0	0	22-Dec-14	1	NA	24-Nov-14	8
N	N	N	N	894	0	26-Dec-14	1	NA	28-Nov-14	7
N	N	N	N	0	0	30-Dec-14	1	NA	2-Dec-14	8
N	N	N	N	398	0	5-Jan-15	1	NA	8-Dec-14	8
N	N	N	N	0	0	6-Jan-15	1	NA	9-Dec-14	9
N	N	N	N	285	0	12-Jan-15	1	NA	15-Dec-14	9
N	N	N	N	647	0	13-Jan-15	1	NA	16-Dec-14	9
N	N	N	N	159	0	14-Jan-15	1	NA	17-Dec-14	7

N	N	N	N	0	0	15-Jan-15	1	NA	18-Dec-14	2
N	N	N	N	0	0	15-Jan-15	1	NA	18-Dec-14	7
N	N	N	N	12	0	19-Jan-15	1	NA	22-Dec-14	10
N	N	N	N	0	5	19-Jan-15	1	NA	22-Dec-14	8
N	N	N	N	0	0	19-Jan-15	1	NA	22-Dec-14	7
N	N	N	N	0	0	19-Jan-15	1	NA	22-Dec-14	9
N	N	N	N	27	0	19-Jan-15	1	NA	22-Dec-14	8
N	N	N	N	496	0	19-Jan-15	1	NA	22-Dec-14	8
N	N	N	N	574	0	20-Jan-15	3	NA	23-Dec-14	
N	N	N	N	0	0	20-Jan-15	1	NA	23-Dec-14	11
N	N	N	N	256	0	20-Jan-15	1	NA	23-Dec-14	8
N	N	N	N	274	0	26-Jan-15	1	NA	29-Dec-14	8
N	N	N	N	0	0	26-Jan-15	1	NA	29-Dec-14	9
N	N	N	N	0	0	27-Jan-15	1	NA	30-Jan-14	3
N	N	N	N	0	0	27-Jan-15	1	NA	30-Dec-14	1
N	N	N	N	0	0	27-Jan-15	1	NA	30-Dec-14	9
N	N	N	N	0	0	2-Feb-15	1	NA	5-Jan-15	9
N	N	N	N	0	0	2-Feb-15	1	NA	5-Jan-15	8
N	N	N	N	0	0	2-Feb-15	1	NA	5-Jan-15	7
N	N	N	N	419	0	3-Feb-15	1	NA	6-Jan-15	8
N	N	N	N	0	0	3-Feb-15	1	NA	6-Jan-15	9
N	N	N	N	2368	0	3-Feb-15	1	NA	6-Jan-15	7
N	N	N	N	1283		9-Feb-15	1	NA	12-Jan-15	8
N	N	N	N	6		9-Feb-15	1	NA	12-Jan-15	6
N	N	N	N	8	0	15-Jan-15	1	NA	12-Jan-15	6
N	N	N	N	0	0	17-Feb-15	1	NA	20-Jan-15	9
N	N	N	N	31	0	23-Feb-15	1	NA	26-Feb-15	7
N	N	N	N	97	0	23-Feb-15	1	NA	26-Jan-15	3
N	N	N	N	15		23-Feb-15	1	NA	26-Jan-15	3
						2-Mar-15	1	NA	2-Feb-15	8
						2-Mar-15	1	NA	2-Feb-15	7
N	N	N	N	623		17-Feb-15	1	NA	20-Jan-15	10
N	N	N	N	1	0	13-Jan-15	1	NA	16-Dec-14	8
N	N	N	N	422		12-Oct-15	1	NA	14-Sep-15	1
N	N	N	N	1		12-Oct-15	1	NA	14-Sep-15	8
N	N	N	N	1295		12-Oct-15	1	NA	14-Sep-15	6
N	N	N	N	0	0	12-Oct-15	1	NA	14-Sep-15	7
N	N	N	N	3685		6-Oct-15	1	NA	8-Sep-15	7
N	N	N	N	1		6-Oct-15	1	NA	8-Sep-15	8
N	N	N	N	527		6-Oct-15	1	NA	8-Sep-15	7
N	N	N	N	2	7	6-Oct-15	1	NA	8-Sep-15	6
N	N	N	N	1		6-Oct-15	1	NA	8-Sep-15	6
N	N	N	N			6-Oct-15	1	NA	8-Sep-15	8
N	N	N	N			11-Sep-15	1	NA	8-Sep-15	9
N	N	N	N	665		6-Oct-15	1	NA	8-Sep-15	6
N	N	N	N			6-Oct-15	1	NA	8-Sep-15	8
N	N	N	N			6-Oct-15	3	NA	8-Sep-15	

N	N	N	N	50		5-Oct-15	1	NA	7-Sep-15	5
N	N	N	N	2563		10-Sep-15	1	NA	7-Sep-15	7
N	N	N	N			5-Oct-15	1	NA	7-Sep-15	6
N	N	N	N	0	0	5-Oct-15	1	NA		
	N	N	N	794		1-Dec-15	1	NA	3-Nov-15	11
	N		N	1		1-Dec-15	1	NA	3-Nov-15	6
N	N	N	N			1-Dec-15	1	NA	3-Nov-15	7
N	N	N	N	794		1-Dec-15	1	NA	3-Nov-15	7
	N		N	1		1-Dec-15	1	NA	3-Nov-15	6
N	N	N	N	1		1-Dec-15	1	NA	3-Nov-15	7
N	N	N	N	1253		1-Dec-15	1	NA	3-Nov-15	7
	N		N	482		1-Dec-15	1	NA	3-Nov-15	7
	N		N	395		6-Nov-15	1	NA	3-Nov-15	7
N	N	N	N	15		1-Dec-15	1	NA	3-Nov-15	8
N	N	N	N			1-Dec-15	1	NA	3-Nov-15	6
	N		N	1135		1-Dec-15	1	NA	3-Nov-15	6
	N		N	1		1-Dec-15	1	NA	3-Nov-15	2
	N		N	539		30-Nov-15	1	NA	2-Nov-15	10
	N		N	12		30-Nov-15	1	NA	2-Nov-15	7
	N		N	684		30-Nov-15	1	NA	2-Nov-15	6
	N		N	1283		30-Nov-15	1	NA	2-Nov-15	7
	N		N	295		30-Nov-15	1	NA	2-Nov-15	6
	N		N	3586		30-Nov-15	1	NA	2-Nov-15	7
	N		N	643		30-Nov-15	1	NA	2-Nov-15	8
	N		N	794		30-Nov-15	1	NA	2-Nov-15	6
	N		N	896		30-Nov-15	1	NA	2-Nov-15	7
	N		N	1248		30-Nov-15	1	NA	2-Nov-15	2
	N		N	369		30-Nov-15	1	NA	2-Nov-15	8
N	N	N	N	1		30-Nov-15	1	NA	2-Nov-15	8
N	N	N	N	3		24-Nov-15	1	NA	27-Oct-15	9
N	N	N	N	12		24-Nov-15	1	NA	27-Oct-15	7
N	N	N	N	3875		5-Oct-15	1	NA	7-Sep-15	6
N	N	N	N	13		5-Oct-15	1	NA	7-Sep-15	7
N	N	N	N			5-Oct-15	1	NA	7-Sep-15	8
N	N	N	N	1387		5-Oct-15	1	NA	7-Sep-15	6
N	N	N	N	1		5-Oct-15	1	NA	7-Sep-15	7
N	N	N	N	269		5-Oct-15	1	NA	7-Sep-15	8
N	N	N	N	2988		5-Oct-15	1	NA	7-Sep-15	12
N	N	N	N	237		29-Sep-15	1	NA	1-Sep-15	6
N	N	N	N	12		29-Sep-15	1	NA	1-Sep-15	6
N	N	N	N	822		4-Sep-15	1	NA	1-Sep-15	7
N	N	N	N	8		29-Sep-15	1	NA	1-Sep-15	12
N	N	N	N	9		29-Sep-15	1	NA	1-Sep-15	7
N	N	N	N	768		29-Sep-15	1	NA	1-Sep-15	6
N	N	N	N	1		29-Sep-15	1	NA	1-Sep-15	8
N	N	N	N	35		29-Sep-15	1	NA	1-Sep-15	6
N	N	N	N	1		28-Sep-15	1	NA	31-Aug-15	8

N	N	N	N	1397		28-Sep-15	1	NA	31-Aug-15	7
N	N	N	N	5		3-Sep-15	1	NA	31-Aug-15	6
N	N	N	N			28-Sep-15	1	NA	31-Aug-15	6
N	N	N	N	895		28-Sep-15	1	NA	31-Aug-15	8
N	N	N	N			29-Sep-15	1	NA	1-Sep-15	7
N	N	N	N	148		17-Nov-15	1	NA	20-Oct-15	6
N	N	N	N	2275		22-Oct-15	1	NA	19-Oct-15	8
N	N	N	N	473		16-Nov-15	1	NA	19-Oct-15	7
N	N	N	N	2198		16-Nov-15	1	NA	19-Oct-15	8
N	N	N	N	893		16-Nov-15	1	NA	19-Oct-15	7
N	N	N	N	0		16-Nov-15	1	NA	19-Oct-15	7
N	N	N	N	1243		16-Nov-15	1	NA	19-Oct-15	6
N	N	N	N	1197		16-Nov-15	1	NA	19-Oct-15	6
N	N	N	N	1375		16-Nov-15	1	NA	19-Oct-15	6
N	N	N	N	2264		16-Nov-15	1	NA	19-Oct-15	8
N	N	N	N	1298		16-Oct-15	1	NA	19-Oct-15	7
N	N	N	N	541		16-Oct-15	1	NA	19-Oct-15	6
N	N	N	N	219		16-Oct-15	1	NA	13-Oct-15	7
N	N	N	N	1655		10-Nov-15	1	NA	13-Oct-15	9
N	N	N	N	902		10-Nov-15	1	NA	13-Oct-15	6
N	N	N	N	967		16-Oct-15	1	NA	13-Oct-15	5
N	N	N	N	227		10-Nov-15	1	NA	13-Oct-15	10
N	N	N	N	442		10-Nov-15	1	NA	13-Oct-15	7
N	N	N	N	1275		10-Nov-15	1	NA	13-Oct-15	7
N	N	N	N	729		10-Nov-15	1	NA	13-Oct-15	6
N	N	N	N			10-Nov-15	1	NA	13-Oct-15	7
N	N	N	N	552		9-Nov-15	1	NA	12-Oct-15	6
N	N	N	N	510		9-Nov-15	1	NA	12-Oct-15	10
N	N	N	N			9-Nov-15	1	NA	12-Oct-15	9
N	N	N	N	3116		9-Nov-15	1	NA	12-Oct-15	7
N	N	N	N	1	4	9-Nov-15	1	NA	12-Oct-15	7
N	N	N	N	2967		9-Nov-15	1	NA	12-Oct-15	6
N	N	N	N	97		9-Nov-15	1	NA	12-Oct-15	9
N	N	N	N	1421		9-Nov-15	1	NA	12-Oct-15	6
N	N	N	N	736		9-Nov-15	1	NA	12-Oct-15	4
N	N	N	N	1005		26-Oct-15	1	NA	28-Sep-15	7
N	N	N	N	0	0	20-Oct-15	1	NA	22-Sep-15	6
N	N	N	N	1614		20-Oct-15	1	NA	22-Sep-15	7
N	N	N	N			20-Oct-15	1	NA	22-Sep-15	6
N	N	N	N	1581		20-Oct-15	1	NA	22-Sep-15	7
N	N	N	N	2912		20-Oct-15	1	NA	22-Sep-15	8
N	N	N	N	1206		25-Sep-15	1	NA	22-Sep-15	7
N	N	N	N	13		10-Oct-15	1	NA	22-Sep-15	7
N	N	N	N	1974		20-Oct-15	1	NA	22-Sep-15	6
N	N	N	N	1433		20-Oct-15	1	NA	22-Sep-15	6
N	N	N	N			13-Oct-15	1	NA	15-Sep-15	7
N	N	N	N	1358	0	13-Oct-15	1	NA	15-Sep-15	6

N	N	N	N			13-Oct-15	1	NA	15-Sep-15	6
N	N	N	N	1296		13-Oct-15	1	NA	15-Sep-15	7
N	N	N	N	1296		13-Oct-15	1	NA	15-Sep-15	6
N	N	N	N	0	1	13-Oct-15	1	NA	15-Sep-15	6
N	N	N	N	1365		13-Oct-15	1	NA	15-Sep-15	6
N	N	N	N			13-Oct-15	1	NA	15-Sep-15	3
N	N	N	N	0		13-Oct-15	1	NA	15-Sep-15	7
N	N	N	N	2	1	13-Oct-15	1	NA	15-Sep-15	7
N	N	N	N	1472		13-Oct-15	1	NA	15-Sep-15	7
N	N	N	N	1379		12-Oct-15	1	NA	14-Sep-15	6
N	N	N	N	247		12-Oct-15	1	NA	14-Sep-15	7
N	N	N	N	78		12-Oct-15	1	NA	14-Sep-15	7
N	N	N	N	836	0	12-Oct-15	1	NA	14-Sep-15	7
N	N	N	N	2		12-Oct-15	1	NA	14-Sep-15	6
N	N	N	N	1		20-Oct-15	1	NA	22-Sep-15	11
N	N	N	N	25		28-Sep-15	1	NA	31-Aug-15	7
N	N	N	N	1238		28-Sep-15	1	NA	31-Aug-15	8
N	N	N	N	299		28-Sep-15	1	NA	31-Aug-15	7
N	N	N	N	926		28-Sep-15	1	NA	31-Aug-15	7
N	N	N	N	15		28-Sep-15	1	NA	31-Aug-15	10
N	N	N	N	127		22-Sep-15	1	NA	25-Aug-15	8
N	N	N	N	1215		22-Sep-15	1	NA	25-Aug-15	6
N	N	N	N	26		22-Sep-15	1	NA	25-Aug-15	7
N	N	N	N			22-Sep-15	1	NA	25-Aug-15	7
N	N	N	N			22-Sep-15	1	NA	25-Aug-15	8
N	N	N	N	368		22-Sep-15	1	NA	25-Aug-15	7
N	N	N	N	35		22-Sep-15	1	NA	25-Aug-15	8
N	N	N	N	35		22-Sep-15	1	NA	25-Aug-15	6
N	N	N	N	770		22-Sep-15	1	NA	25-Aug-15	7
N	N	N	N	679		22-Sep-15	1	NA	25-Aug-15	6
N	N	N	N			21-Sep-15	1	NA	24-Aug-15	11
N	N	N	N		4	21-Sep-15	1	NA	24-Aug-15	10
N	N	N	N	426		21-Sep-15	1	NA	24-Aug-15	8
N	N	N	N	2587		21-Sep-15	1	NA	24-Aug-15	8
N	N	N	N	1		21-Sep-15	1	NA	24-Aug-15	8
N	N	N	N	1		21-Sep-15	1	NA	24-Aug-15	8
N	N	N	N	476		21-Sep-15	1	NA	24-Aug-15	9
N	N	N	N			21-Sep-15	1	NA	24-Aug-15	11
N	N	N	N	1		21-Sep-15	1	NA	24-Aug-15	9
N	N	N	N	2479		21-Sep-15	1	NA	24-Aug-15	7
N	N	N	N	1298		21-Sep-15	1	NA	24-Aug-15	8
N	N	N	N			21-Sep-15	1	NA	24-Aug-15	9
N	N	N	N	476		21-Sep-15	1	NA	24-Aug-15	8
N	N	N	N	739		15-Sep-15	1	NA	18-Aug-15	7
N	N	N	N	189		15-Sep-15	3	NA	18-Aug-15	
	N		N	285		14-Sep-15	1	NA	17-Aug-15	8
N	N	N	N			14-Sep-15	1	NA	17-Aug-15	9

N	N	N	N	1		14-Sep-15	1	NA	17-Aug-15	1
N	N	N	N			14-Sep-15	1	NA	17-Aug-15	1
N	N	N	N	846		14-Sep-15	1	NA	17-Aug-15	9
N	N	N	N	1643		14-Sep-15	1	NA	17-Aug-15	11
N	N	N	N	0		3-Nov-15	1	NA	6-Oct-15	7
N	N	N	N	46		3-Nov-15	1	NA	6-Oct-15	7
N	N	N	N	1629		3-Nov-15	1	NA	6-Oct-15	6
N	N	N	N	1597		3-Nov-15	1	NA	6-Oct-15	1
N	N	N	N	524		3-Nov-15	1	NA	6-Oct-15	8
N	N	N	N	5		2-Nov-15	1	NA	5-Oct-15	1
N	N	N	N	606		3-Nov-15	1	NA	6-Oct-15	6
N	N	N	N	1989		2-Nov-15	1	NA	5-Oct-15	7
N	N	N	N	429		2-Nov-15	1	NA	5-Oct-15	7
N	N	N	N	285		2-Nov-15	1	NA	5-Oct-15	7
N	N	N	N			2-Nov-15	1	NA	5-Oct-15	7
N	N	N	N	255		2-Nov-15	1	NA	5-Oct-15	8
N	N	N	N	2379		2-Nov-15	1	NA	5-Oct-15	7
N	N	N	N	357		27-Oct-15	1	NA	29-Sep-15	8
N	N	N	N			27-Oct-15	1	NA	29-Sep-15	6
N	N	N	N			27-Oct-15	1	NA	29-Sep-15	7
N	N	N	N	1735		27-Oct-15	1	NA	29-Sep-15	7
N	N	N	N	2211		27-Oct-15	1	NA	29-Sep-15	6
N	N	N	N	5		27-Oct-15	1	NA	29-Sep-15	6
N	N	N	N	2		27-Oct-15	1	NA	29-Sep-15	7
N	N	N	N			27-Oct-15	1	NA	29-Sep-15	6
N	N	N	N	1	1	27-Oct-15	1	NA	29-Sep-15	7
N	N	N	N	317		26-Oct-15	1	NA	28-Oct-15	7
N	N	N	N	0	0	26-Oct-15	1	NA	28-Sep-15	7
N	N	N	N	0		26-Oct-15	1	NA	28-Sep-15	7
N	N	N	N	0	0	26-Oct-15	1	NA	28-Sep-15	8
N	N	N	N	2715		26-Oct-15	1	NA	28-Sep-15	6
N	N	N	N	126		26-Oct-15	1	NA	28-Sep-15	2
N	N	N	N	64		26-Oct-15	1	NA	28-Sep-15	3
N	N	N	N	1163		26-Oct-15	1	NA	28-Sep-15	6
N	N	N	N	124		26-Oct-15	1	NA	28-Sep-15	8
N	N	N	N	0		24-Nov-15	1	NA	27-Oct-15	6
N	N	N	N	693		24-Nov-15	1	NA	27-Oct-15	8
N	N	N	N	1		24-Nov-15	1	NA	27-Oct-15	6
N	N	N	N	195		24-Nov-15	1	NA	27-Oct-15	7
N	N	N	N	837		24-Nov-15	1	NA	27-Oct-15	7
N	N	N	N	2984		24-Nov-15	1	NA	27-Oct-15	8
N	N	N	N	15		24-Nov-15	1	NA	27-Oct-15	8
N	N	N	N	296		30-Oct-15	1	NA	27-Oct-15	7
N	N	N	N	1175		24-Nov-15	1	NA	27-Oct-15	6
N	N	N	N	526		23-Nov-15	1	NA	26-Oct-15	4
N	N	N	N	974		23-Nov-15	1	NA	26-Oct-15	6
N	N	N	N	0	0	23-Nov-15	1	NA	26-Oct-15	7

N	N	N	N	0	0	23-Nov-15	1	NA	26-Nov-15	7
N	N	N	N	2793		23-Nov-15	1	NA	26-Oct-15	6
N	N	N	N	1895		23-Nov-15	1	NA	26-Oct-15	8
N	N	N	N	1268		23-Nov-15	1	NA	26-Oct-15	7
N	N	N	N	0	0	23-Nov-15	1	NA	26-Oct-15	9
N	N	N	N	736		23-Nov-15	1	NA	26-Oct-15	7
N	N	N	N	967		23-Nov-15	1	NA	26-Oct-15	8
N	N	N	N	1287		23-Nov-15	1	NA	26-Oct-15	7
N	N	N	N	2		23-Nov-15	1	NA	26-Oct-15	6
N	N	N	N	946		23-Nov-15	1	NA	26-Oct-15	8
N	N	N	N	1		17-Nov-15	1	NA	20-Oct-15	9
N	N	N	N	163		17-Nov-15	1	NA	20-Oct-15	6
N	N	N	N	975		17-Nov-15	1	NA	20-Oct-15	7
N	N	N	N	2		17-Nov-15	1	NA	20-Oct-15	9
N	N	N	N	1		17-Nov-15	1	NA	20-Oct-15	7
N	N	N	N			17-Nov-15	1	NA	20-Oct-15	8
N	N	N	N	1248		17-Oct-15	1	NA	20-Oct-15	7
N	N	N	N			17-Nov-15	1	NA	20-Oct-15	8

q21bd28	q22d28	q23d28	q24d28	q25d28	q26d28	q27d28	q28d28	q29d28	q30d28	q31d28	q32d28
32	2	3	1	2	88	17-Nov-14	1	2	2	1	2
8	2	3	1	2	88	17-Nov-14	2				
10	2	3	1	2	88	17-Nov-14	2				
30	2	3	1	2	88	17-Nov-14	1	2	2	1	2
0	2	3	1	2	88	27-Nov-14	2				
40	2	3	1	2	88	18-Nov-14	2				
10	2	3	1	2	88	18-Nov-14	2				
17	2	3	1	2	88	18-Nov-14	2				
20	2	3	1	2	88	18-Nov-14	2				
50	2	3	1	2	88	18-Nov-14	2				
47	2	3	2	2	88	25-Nov-14	2				
8	2	3	1	2	88	25-Nov-14	1	2	2	1	2
45	2	3	1	2	88	25-Nov-14	2				
30	2	3	1	2	88	1-Dec-14	2				
37	2	3	1	2	88	1-Dec-14	2				
55	2	3	1	2	88	1-Dec-14	2				
36	2	3	1	2	88	2-Dec-14	2				
30	2	3	1	2	88	2-Dec-14	2				
14	2	3	1	2	88	2-Dec-14	2				
44	2	3	1	2	88	2-Dec-14	2				
18	2	3	1	2	88	2-Dec-14	1	2	2	1	
43	2	3	1	2	88	2-Feb-14	2				
30	2	3	1	2	88	2-Feb-14	2				
50	2	3	1	2	88	8-Dec-14	2				
15	2	3	1	2	88	8-Dec-14	2				
54	2	3	1	2	88	8-Dec-14	2				
25	2	3	1	2	88	8-Dec-14	2				
8	2	3	1	2	88	8-Dec-14	2				
30	2	3	1	2	88	9-Dec-14	2				
40	2	3	1	2	88	9-Dec-14	2				
0	2	3	1	2	88	9-Dec-14	1	2	2	1	
33	2	3	1	2	88	9-Dec-14	2				
41	2	3	1	2	88	15-Dec-14	2				
15	2	3	1	2	88						
40	2	3	1	2	88	15-Dec-14	2				
15	2	3	1	2	88	15-Dec-14	2				
50	2	3	1	2	88	22-Dec-14	1	2	2	1	
20	2	3	1	2	88	22-Dec-14	2				
10	2	3	1	2	88	26-Dec-14	2				
30	2	3	1	2	88	30-Dec-14	2				
35	2	3	1	2	88	5-Dec-14	1	2	2	1	
40	2	3	1	2	88	6-Jan-15	2				
11	2	3	1	2	88	12-Jan-15	2				
4	1	3	1	2	88	13-Jan-15	1	2	1		
16	2	3	1	2	88	14-Jan-15	2				

49	2	3	1	2	88	15-Jan-15	2				
30	2	3	1	2	88	15-Jan-15	2				
20	2	3	1	2	88	19-Jan-15	2				
33	2	3	1	2	88	19-Jan-15	2				
50	2	3	1	2	88	19-Jan-15	2				
30	2	3	1	2	88	19-Jan-15	2				
58	2	3	1	2	88	19-Jan-15	2				
33	2	3	1	2	88	19-Jan-15	1	2	2	1	2
35	2	3	1	2	88	20-Jan-15	2				
40	2	3	1	2	88	20-Jan-15	2				
40	2	3	1	2	88	26-Jan-15	1	2	2	1	2
10	2	3	1	2	88	26-Jan-15	2				
15	2	3	1	2	88	27-Jan-15	2				
34	2	3	1	2	88	27-Jan-15	2				
15	2	3	1	2	88	27-Jan-15	2				
10	2	3	1	2	88	2-Feb-15	2				
40	2	3	1	2	88	2-Feb-15	2				
40	2	3	1	2	88	2-Feb-15	2				
3	2	3	1	2	88	3-Feb-15	2				
16	2	3	1	2	88	3-Feb-15	2				
40	2	3	1	2	88	3-Feb-15	1	2	2	1	2
49	2	3	1	2	88	9-Feb-15	2				
48	1	3	1	2	88	9-Feb-15	2				
40	2	3	1	2	88	9-Feb-15	2				
35	2	3	1	2	88	17-Feb-15	2				
10	2	3	1	2	88	23-Feb-15	2				
30	2	3	1	2	88	23-Feb-15	2				
2	2	3	1	2	88	23-Feb-15	2				
41	2	3	1	2	88	2-Mar-15	2				
30	2	3	1	2	88	2-Mar-15	2				
55	2	3	1	2	88	17-Feb-15	2				
40	2	3	1	2	88	13-Jan-15	2				
20	2	3	1	2	88	12-Oct-15	2				
5	2	3	1	2	88	12-Oct-15	1		2	1	
10	2	3	1	2	88	12-Oct-15	1	2	2	1	
10	2	3	1	2	88	12-Oct-15	2				
0	2	3	1	2	88	6-Oct-15	1	2	2	1	
3	2	3	1	2	88	6-Oct-15	2				
26	2	3	1	1	2	6-Oct-15	1	2	2	1	
37	2	3	1	2	88	6-Oct-15	2				
15	2	3	1	2	88	6-Oct-15	2				
50	2	3	1	2	88	6-Oct-15	2				
0	2	3	1	2	88	6-Oct-15	2				
45	2	3	1	2	88	6-Oct-15	1	2	2	1	2
12	2	3	1	2	88	6-Oct-15	2				

20	2	3	1	2	88	5-Oct-15	2				
29	2	3	1	2	88	5-Oct-15	1	2	2	1	
37	2	3	1	2	88	5-Oct-15	2				
	2	3	1	2	88	5-Oct-15	2				
12	2	3	1	2	88	1-Dec-15	2				
44	2	3	1	2	88	1-Dec-15	2				
25	2	3	1	2	88	1-Dec-15	2				
40	2	3	1	2	88	1-Dec-15	2				
18	2	3	1	2	88	1-Dec-15	2				
0	2	3	1	2	88	1-Dec-15	2				
8	2	3	1	2	88	1-Dec-15	2				
10	2	3	1	2	88	1-Dec-15	2				
40	2	3	1	2	88	1-Dec-15	2				
50	2	3	1	2	88	1-Dec-15	2				
31	2	3	1	2	88	1-Dec-15	2				
39	2	3	1	2	88	1-Dec-15	2				
10	2	3	1	2	88	1-Dec-15	2				
15	2	3	1	2	88	30-Nov-15	2				
20	2	3	1	2	88	30-Nov-15	2				
20	2	3	1	2	88	30-Nov-15	2				
45	2	3	1	2	88	30-Nov-15	2				
18	2	3	1	2	88	30-Nov-15	2				
25	2	3	1	2	88	30-Nov-15	2				
15	2	3	1	2	88	30-Nov-15	2				
50	2	3	1	2	88	30-Nov-15	2				
9	2	3	1	2	88	30-Nov-15	2				
10	2	3	1	2	88	30-Nov-15	2				
30	2	3	1	2	88	30-Nov-15	2				
47	2	3	1	2	88	30-Nov-15	2				
30	2	3	1	2	88	24-Nov-15	2				
2	2	3	1	2	88	24-Nov-15					
15	2	3	1	2	88	5-Oct-15	2				
10	2	3	1	2	88	5-Oct-15	2				
25	2	3	1	2	88	5-Oct-15	2				
40	2	3	1	2	88	5-Oct-15	1	2	2	1	2
37	2	3	1	2	88	5-Oct-15	2				
44	1	3	1	2	88	5-Oct-15	1	2	1		
55	2	3	1	2	88	5-Oct-15	1	2	2	1	2
30	2	3	1	2	88	29-Sep-15	2				
50	2	3	1	2	88	29-Sep-15	2				
10	2	3	1	2	88	29-Sep-15	1	2	2	1	
20	2	3	1	2	88	29-Sep-15	2				
21	2	3	1	2	88	29-Sep-15	2				
44	2	3	1	2	88	29-Sep-15	1	2	2	1	2
4	2	3	1	2	88	29-Sep-15	1	2	2	1	2
21	2	3	1	2	88	29-Sep-15	1	2	2	1	2
17	2	3	1	2	88	28-Sep-15	2				

9	2	3	1	2	88	28-Sep-15	1	2	2	1	2
40	2	3	1	2	88	28-Sep-15	1	2	2	1	2
15	1	3	1	2	88	28-Sep-15	2				
0	2	3	1	2	88	28-Sep-15	2				
42	2	3	1	2	88	29-Sep-15	2				
15	2	3	1	2	88	17-Nov-15	2				
15	2	3	1	1		16-Nov-15	1	2	2	1	
0	1	2	1	2	88	16-Nov-15	1	2	2	1	
2	2	3	1	2	88	16-Nov-15	1	2	2	1	
21	2	3	1	2	88	16-Nov-15	1	2	2	1	
41	2	3	1	2	88	16-Nov-15	2				
39	2	3	1	2	88	16-Nov-15	1	2	2	1	
20	2	3	1	2	88	16-Nov-15	1	2	2	1	
37	2	3	1	2	88	16-Nov-15	1	2	2	1	
32	2	3	1	2	88	16-Nov-15	1	2	2	1	
21	2	3	1	2	88	16-Nov-15	1	2	2	1	
20	2	3	1	2	88	16-Nov-15	1	2	2	1	
5	2	3	1	2	88	10-Nov-15	2				
10	2	3	1	2	88	10-Nov-15	1	2	2	1	
20	2	3	1	2	88	10-Nov-15	1	2	2	1	
10	2	3	1	2	88	10-Nov-15	1	2	2	1	
3	2	3	1	2	88	10-Nov-15	1	2	2	1	
10	2	3	1	2	88	10-Nov-15	2				
31	2	3	1	2	88	10-Nov-15	1	2	2	1	
46	2	3	1	2	88	10-Nov-15	1	2	2	1	2
56	2	3	1	2	88	10-Nov-15					
20	2	3	1	2	88	9-Nov-15	1	2	2	1	2
35	2	3	1	2	88	9-Nov-15	1	2	2	1	
10	2	3	1	2	88	9-Nov-15	1	2	2	1	
20	2	3	1	2	88	9-Nov-15	1	2	2	1	2
0	2	3	1	2	88	9-Nov-15	1	2	2	1	2
33	2	3	1	2	88	9-Nov-15	2				
50	2	3	1	2	88	9-Nov-15	2				
50	2	3	1	2	88	9-Nov-15	1	2	2	1	1
30	2	3	1	2	88	9-Nov-15	1	2	2	1	
40	2	3	1	2	88	26-Oct-15	1	2	2	1	
30	2	3	1	2	88	20-Oct-15	2				
40	2	3	1	2	88	20-Oct-15	1	2	2	1	
47	2	3	1	2	88	20-Oct-15	2				
18	2	3	1	2	2	20-Oct-15	1	2	2	1	2
23	2	3	1	2	88	20-Oct-15	1	2	2	1	
48	2	3	1	2	88	20-Oct-15	1	2	2	1	2
15	2	3	1	2	88	20-Oct-15	2				
45	2	3	1	2	88	20-Oct-15	1	2	2	1	
8	2	3	1	2	88	20-Oct-15	2				
13	2	3	1	2	88	13-Oct-15	2				
20	2	3	1	2	88	13-Oct-15	2				

34	2	3	1	2	88	13-Oct-15	2			
50	2	3	1	2	88	13-Oct-15	1	2	2	1	2
48	2	3	1	2	88	13-Oct-15	1	2	2	1	2
10	2	3	1	2	88	13-Oct-15	2				
10	2	3	1	2	88	13-Oct-15	1	2	2	2	2
10	2	3	1	2	88	13-Oct-15	2				
17	2	3	1	2	88	13-Oct-15	1	2	2	1	
20	2	3	1	2	88	13-Oct-15	2				
15	2	3	1	2	88	13-Oct-15	1	2	2	1	
44	2	3	1	2	88	12-Oct-15	2				
58	2	3	1	2	88	12-Oct-15	1	2	2	1	2
31	2	3	1	2	88	12-Oct-15	2				
4	2	3	1	2	88	12-Oct-15	1	2	2	1	2
30	2	3	1	2	88	12-Oct-15	2				
20	2	3	1	2	88	20-Oct-15	2				
35	2	3	1	2	88	28-Sep-15	1	2	1	1	2
40	2	3	1	2	88	28-Sep-15	1	2	2	1	2
35	2	3	1	2	88	28-Sep-15	2				
12	2	3	1	2	88	28-Sep-15	1	2	2	2	2
50	2	3	1	2	88	28-Sep-15	2				
20	2	3	1	2	88	22-Sep-15	2				
27	2	3	1	2	88	22-Sep-15	2				
20	2	3	1	2	88	22-Sep-15	1	2	2	1	
15	1	3	1	2	88	22-Sep-15	2				
35	2	3	1	2	88	22-Sep-15	2				
39	2	3	1	2	88	22-Sep-15	2				
0	2	3	1	2	88	22-Sep-15	2				
20	2	3	1	2	88	22-Sep-15	1	2	2	1	2
38	2	3	1	2	88	22-Sep-15	1	2	2	1	
5	2	3	1	2	88	22-Sep-15	2				
20	2	3	1	2	88	21-Sep-15	2				
11	2	3	1	2	88	21-Sep-15	2				
10	2	3	1	2	88	21-Sep-15	2				
24	2	3	1	2	88	21-Sep-15	1	2	2	1	2
50	2	3	1	2	88	21-Sep-15	2				
7	2	3	1	2	88	21-Sep-15	2				
40	2	3	1	2	88	21-Sep-15	2				
0	2	3	1	2	88	21-Sep-15	2				
8	2	3	1	2	88	21-Sep-15	1	2	2	1	2
36	2	3	1	2	88	21-Sep-15	1	2	2	1	
35	2	3	1	2	88	21-Sep-15	1	2	2	1	
15	2	3	1	2	88	21-Sep-15	2				
50	2	3	1	2	88	21-Sep-15	2				
25	2	3	1	2	88	15-Sep-15	2				
53	2	3	1	2	88	14-Sep-15	2				
45	2	3	1	2	88	14-Sep-15	1	2	2	1	

35	2	3	1	2	88	14-Sep-15	2				
20	2	3	1	2	88	14-Sep-15	1	2	2	1	
10	2	3	1	2	88	14-Sep-14	1	2	2	1	
0	2	3	1	2	88	14-Sep-15	2				
52	2	3	1	2	88	3-Nov-15	1	2	2	1	2
28	2	3	1	2	88	3-Nov-15	1	2	2	1	2
37	2	3	1	2	88	3-Nov-15	1	2	2	1	2
55	2	3	1	2	88	3-Nov-15	1	2	2	1	2
35	2	3	1	2	88	3-Nov-15	1	2	2	1	2
5	2	3	1	2	88	2-Nov-15	2				
22	2	3	1	2	88	3-Nov-15	2				
35	2	3	1	2	88	2-Nov-15	1	2	2	1	2
20	2	3	1	2	88	2-Nov-15	2				
0	2	3	1	2	88	2-Nov-15	2				
17	2	3	1	2	88	2-Nov-15	2				
46	2	3	1	2	88	2-Nov-15	1	2	2	1	2
52	2	3	1	2	88	2-Nov-15	1	2	2	1	2
17	2	3	1	2	88	27-Oct-15	1	2	2	1	2
15	2	3	1	2	88	27-Oct-15	1	2	2	1	2
50	2	3	1	2	88	27-Oct-15	1	2	2	1	2
30	2	3	1	2	88	27-Oct-15	2				
50	2	3	1	2	88	27-Oct-15	2				
20	2	3	1	2	88	27-Oct-15	2				
23	2	3	1	2	88	27-Oct-15	2				
37	2	3	1	2	88	27-Oct-15	2				
10	2	3	1	2	88	27-Oct-15	1	2	2	1	2
55	2	3	1	2	88	26-Oct-15	2				
4	2	3	1	2	88	26-Oct-15	2				
40	2	3	1	2	88	26-Oct-15	2				
20	2	3	1	2	88	26-Oct-15	2				
36	2	3	1	2	88	26-Oct-15	2				
35	2	3	1	2	88	26-Oct-15	1	2	2	1	
10	2	3	1	2	88	26-Oct-15	1	2	2	1	
30	2	3	1	2	88	26-Oct-15	2				
1	2	3	1	2	88	26-Oct-15	1	2	2	1	
46	2	3	1	2	88	24-Nov-15					
0	2	3	1	2	88	24-Nov-15					
27	2	3	1	2	88	24-Nov-15					
18	2	3	1	2	88	24-Nov-15					
40	2	3	1	2	88	24-Nov-15					
50	2	3	1	2	88	24-Nov-15					
10	2	3	1	2	88	24-Nov-15	2				
10	2	3	1	2	88	24-Nov-15	2				
30	2	3	1	2	88	24-Nov-15	1	2	2	1	
33	2	3	1	2	88	24-Nov-15	2				
32	2	3	1	2	88	23-Nov-15	1	2	2	1	
39	2	3	1	2	88	23-Nov-15	1	2	2	1	

15	2	3	1	2	88	23-Nov-15	1	2	2	1
10	2	3	1	2	88	23-Nov-15	1	2	2	1
22	2	3	1	2	88	23-Nov-15	1	2	2	1
26	2	3	1	2	88	23-Nov-15	1	2	2	1
40	2	3	1	2	88	23-Nov-15	1	2	2	1
3	2	3	1	2	88	23-Nov-15	1	2	2	1
20	2	3	1	2	88	23-Nov-15	1	2	2	1
44	2	3	1	2	88	23-Nov-15	1	2	2	1
26	2	3	1	2	88	23-Nov-15	1	2	2	1
45	2	3	1	2	88	23-Nov-15	1	2	2	1
0	2	3	1	2	88	17-Nov-15	1	2	2	1
49	2	3	1	2	88	17-Nov-15	2			
9	2	3	1	2	88	17-Nov-15	2			
57	2	3	1	2	88	17-Nov-15	1	2	2	1
37	2	3	1	2	88	17-Nov-15	2			
18	2	3	1	2	88	17-Nov-15	1	2	2	1
15	2	3	1	2	88	17-Nov-15	1	2	2	1
59	2	3	1	2	88	17-Oct-15	2			

q33d28	q34hhd28	q34mmd28	q36d28	q37d28	asexpfd28	gmcypfd28	asexpmd28	gmcyxpmd28
	7	43	17-Nov-14		2	2	2	2
	11	17	17-Nov-14		2	2	2	2
	7	22	17-Nov-14		2	2	2	2
	8	40	17-Nov-14		2	2	2	2
	10	10	17-Nov-14		2	2	2	2
	7	50	18-Nov-14		2	2	2	2
	8	22	18-Nov-14		2	2	2	2
	9	25	18-Nov-14		2	2	2	2
	7	30	18-Nov-14		2	2	2	2
	6	58	18-Nov-14	18-Nov-14	2	2	2	2
	3	55	25-Nov-14		2	2	2	2
2	3	18	25-Nov-14		2	2	2	2
	2	54	25-Nov-14		2	2	2	2
	6	41	1-Dec-14		2	2	2	2
	7	44	1-Dec-14		2	2	2	2
	7	7	1-Dec-14		2	2	2	2
	6	44	2-Dec-14		2	2	2	2
	6	41	2-Dec-14		2	2	2	2
	7	23	2-Dec-14		2	2	2	2
	6	53	2-Dec-14		2	2	2	2
	8	30	2-Dec-14		2	2	2	2
	7	53	2-Dec-14		2	2	2	2
	7	43	2-Dec-14		2	2	2	2
	7	2	8-Dec-14		2	2	2	2
	7	25	8-Dec-14		2	2	2	2
	9	10	8-Dec-14		2	2	2	2
	6	37	8-Dec-14		2	2	2	2
	6	18	8-Dec-14		2	2	2	2
	2	41	9-Dec-14		2	2	2	2
	6	50	9-Dec-14		2	2	2	2
	7	9	9-Dec-14		2	2	2	2
	7	43	9-Dec-14		2	2	2	2
	2	52	15-Dec-14		2	2	2	2
			15-Dec-14		2	2	2	2
	7	51	15-Dec-14		2	2	2	2
	7	26	15-Dec-14		2	2	2	2
	8	3	22-Dec-14		2	2	2	2
	8	33	2-Dec-14		2	2	2	2
	7	20	29-Dec-14		2	2	2	2
	8	42	20-Dec-14		2	2	2	2
	8	45	5-Jan-15		2	2	2	2
	9	51	6-Jan-15		2	2	2	2
	9	20	12-Jan-15					
	9	12	13-Jan-15		2	2	2	2
	7	30	14-Jan-15		2	2	2	2

3	6					
9	41	15-Jan-15	2	2	2	2
10	33	19-Jan-15	2	2	2	2
8	42	19-Oct-15	2	2	2	2
8	0	19-Jan-15	2	2	2	2
9	38		2	2	2	2
9	5	19-Jan-15	2	2	2	2
8	41	19-Jan-15	2	2	2	2
11	42	20-Jan-15	2	2	2	2
8	49		2	2	2	2
8	48					
9	18					
3	29					
1	42					
9	29					
9	19					
8	47					
7	48					
8	13					
9	26					
7	51					
8	54	9-Feb-15	2	2	2	2
6	55	9-Feb-15	2	2	2	2
6	46	9-Feb-15	2	2	2	2
9	45	17-Feb-15	2	2	2	2
7	23	23-Feb-15	2	2	2	2
3	41	23-Feb-15	2	2	2	2
3	10	23-Feb-15	2	2	2	2
8	52					
7	43					
11	10	17-Feb-15	2	2	2	2
8	50	13-Jan-15	2	2	2	2
1	31	12-Oct-15	2	2	2	2
8	15	12-Oct-15	2	2	2	2
6	21	12-Oct-15	2	2	2	2
7	21	12-Oct-15	2	2	2	2
7	13	6-Oct-15	2	2	2	2
8	16	6-Oct-15	2	2	2	2
7	37	6-Oct-15	2	2	2	2
6	47	6-Oct-15	2	2	2	2
6	30	6-Oct-15	2	2	2	2
8	59	6-Oct-15	2	2	2	2
9	11	6-Oct-15	2	2	2	2
6	53	6-Oct-15	2	2	2	2
8	20	6-Oct-15	2	2	2	2

		5-Oct-15	2	2	2	2
7	43	5-Oct-15	2	2	2	2
6	51	5-Oct-15	2	2	2	2
		5-Oct-15	2	2	2	2
11	20	1-Dec-15	2	2	2	2
6	51	1-Dec-15	2	2	2	2
7	32	1-Dec-15	2	2	2	2
7	46	1-Dec-15	2	2	2	2
6	24	1-Dec-15	2	2	2	2
7	6	1-Dec-15	2	2	2	2
7	16	1-Dec-15	2	2	2	2
7	18	1-Dec-15	2	2	2	2
7	48	1-Dec-15	2	2	2	2
8	58	1-Dec-15	2	2	2	2
6	38	1-Dec-15	2	2	2	2
6	46	1-Dec-15	2	2	2	2
2	20	1-Dec-15	2	2	2	2
10	24	30-Nov-15	2	2	2	2
7	26	30-Nov-15	2	2	2	2
6	27	30-Nov-15	2	2	2	2
7	51	30-Nov-15	2	2	2	2
6	24	30-Nov-15	2	2	2	2
7	30	30-Nov-15	2	2	2	2
8	22	30-Nov-15	2	2	2	2
6	55	30-Nov-15	2	2	2	2
7	15	30-Nov-15	2	2	2	2
2	19	30-Nov-15	2	2	2	2
8	41	30-Nov-15	2	2	2	2
8	56	30-Nov-15	2	2	2	2
9	41	24-Nov-15	2	2	2	2
7	7	24-Nov-15	2	2	2	2
6	25	5-Oct-15	2	2	2	2
7	21	5-Oct-15	2	2	2	2
8	35	5-Oct-15	2	2	2	2
6	46	5-Oct-15	2	2	2	2
7	45	5-Oct-15	2	2	2	2
8	55	5-Oct-15	2	2	2	2
1	3	5-Oct-15	2	2	2	2
6	42	29-Sep-15	2	2	2	2
6	59	29-Sep-15	2	2	2	2
7	23	29-Sep-15	2	2	2	2
12	35	29-Sep-15	2	2	2	2
7	29	29-Sep-15	2	2	2	2
6	55	29-Sep-15	2	2	2	2
8	13	29-Sep-15	2	2	2	2
6	29	29-Sep-15	2	2	2	2
8	28	28-Sep-15	2	2	2	2

7	19	28-Sep-15	2	2	2	2
6	50	28-Sep-15	2	2	2	2
6	23	28-Sep-15	2	2	2	2
8	14	28-Sep-15	2	2	2	2
7	52	29-Sep-15	2	2	2	2
6	29	17-Nov-15	2	2	2	2
8	29	16-Nov-15	2	2	2	2
7	8	16-Nov-15	2	2	2	2
8	14	16-Nov-15	2	2	2	2
7	30	16-Nov-15	2	2	2	2
7	50	16-Nov-15	2	2	2	2
6	47	16-Nov-15	2	2	2	2
6	30	16-Nov-15	2	2	2	2
6	50	16-Nov-15	2	2	2	2
8	43	16-Nov-15	2	2	2	2
7	33	16-Nov-15	2	2	2	2
6	29	10-Nov-15	2	2	2	2
7	18	10-Nov-15	2	2	2	2
9	20	10-Nov-15	2	2	2	2
6	33	10-Nov-15	2	2	2	2
5	23	10-Nov-15	2	2	2	2
10	10	10-Nov-15	1	2	2	2
7	18	10-Nov-15	2	2	2	2
7	42	10-Nov-15	1	2	2	2
6	55	10-Nov-15	2	2	2	2
8	4	10-Nov-15	2	2	2	2
6	28	9-Nov-15	2	2	2	2
10	47	9-Nov-15	2	2	2	2
9	20	9-Nov-15	2	2	2	2
7	27	9-Nov-15	2	2	2	2
7	7	9-Nov-15	2	2	2	2
6	42	9-Nov-15	2	2	2	2
10	2	9-Nov-15	2	2	2	2
6	56	9-Nov-15	2	2	2	2
4	39	9-Nov-15	2	2	2	2
7	50	26-Oct-15	2	2	2	2
6	38	20-Oct-15	2	2	2	2
7	51	20-Oct-15	2	2	2	2
6	55	20-Oct-15	2	2	2	2
7	26	20-Oct-15	2	2	2	2
8	34	20-Oct-15	2	2	2	2
8	0	20-Oct-15	2	2	2	2
7	26	20-Oct-15	2	2	2	2
6	55	20-Oct-15	2	2	2	2
6	18	20-Oct-15	2	2	2	2
7	22	13-Oct-15	2	2	2	2
6	31	13-Oct-15	2	2	2	2

	6	46	13-Oct-15	2	2	2	2
	8	5	13-Oct-15	2	2	2	2
	6	57	13-Oct-15	2	2	2	2
	6	20	13-Oct-15	2	2	2	2
1	6	21	13-Oct-15	2	2	2	2
	3	23	13-Oct-15	2	2	2	2
	7	28	13-Oct-15	2	2	2	2
	7	29	13-Oct-15	2	2	2	2
	7	24	13-Oct-15	2	2	2	2
	6	53	12-Oct-15	2	2	2	2
	8	8	12-Oct-15	2	2	2	2
	7	43	12-Oct-15	2	2	2	2
	7	14	12-Oct-15	2	2	2	2
	6	39	12-Oct-15	2	2	2	2
	11	33	20-Oct-15	2	2	2	2
	7	47	28-Sep-15	2	2	2	2
	8	48	28-Sep-15	2	2	2	2
	7	47	28-Sep-15	2	2	2	2
1	7	22	28-Sep-15	2	2	2	2
	11	2	28-Sep-15	2	2	2	2
	8	33	22-Sep-15	2	2	2	2
	6	42	22-Sep-15	2	2	2	2
	7	32	22-Sep-15	2	2	2	2
	7	27	22-Sep-15	2	2	2	2
	8	45	22-Sep-15	2	2	2	2
	7	48	22-Sep-15	2	2	2	2
	8	9	22-Sep-15	2	2	2	2
	6	32	22-Sep-15	2	2	2	2
	7	50	22-Sep-15	2	2	2	2
	6	15	22-Sep-15	2	2	2	2
	11	33	21-Sep-15	2	2	2	2
	10	23	21-Sep-15	2	2	2	2
	8	20	21-Sep-15	2	2	2	2
	8	36	21-Sep-15	2	2	2	2
	9	0	21-Sep-15	2	2	2	2
	8	15	21-Sep-15	2	2	2	2
	9	50	21-Sep-15	2	2	2	2
	11	12	21-Sep-15	2	2	2	2
	9	17	21-Sep-15	2	2	2	2
	7	47	21-Sep-15	2	2	2	2
	8	46	21-Sep-15	2	2	2	2
	9	26	21-Sep-15	2	2	2	2
	8	59	21-Sep-15	2	2	2	2
	7	32	15-Sep-15	2	2	2	2
			15-Sep-15	2	2	2	2
	9	8	14-Sep-15	2	2	2	2
	9	51	14-Sep-15	2	2	2	2

1	43	14-Sep-15	2	2	2	2
1	35	14-Sep-15	2	2	2	2
9	23	14-Sep-15	2	2	2	2
11	8	14-Sep-15	2	2	2	2
7	58	3-Nov-15	2	2	2	2
8	34	3-Nov-15	2	2	2	2
6	45	3-Nov-15	2	2	2	2
2	0	3-Nov-15	2	2	2	2
8	40	3-Nov-15	2	2	2	2
1	15	2-Nov-15	2	2	2	2
6	30	3-Nov-15	2	2	2	2
7	40	2-Nov-15	2	2	2	2
7	31	2-Nov-15	2	2	2	2
7	8	2-Nov-15	2	2	2	2
7	26	2-Nov-15	2	2	2	2
8	52	2-Nov-15	2	2	2	2
7	58	2-Nov-15	2	2	2	2
8	25	27-Oct-15	2	2	2	2
6	22	27-Oct-15	2	2	2	2
7	59	27-Oct-15	2	2	2	2
7	38	27-Oct-15	2	2	2	2
6	58	27-Oct-15	2	2	2	2
6	31	27-Oct-15	2	2	2	2
7	35	27-Oct-15	2	2	2	2
6	50	27-Oct-15	2	2	2	2
7	20	27-Oct-15	2	2	2	2
8	4	26-Oct-15	2	2	2	2
7	15	26-Oct-15	2	2	2	2
7	52	26-Oct-15	2	2	2	2
8	32	26-Oct-15	2	2	2	2
6	45	26-Oct-15	2	2	2	2
2	48	26-Oct-15	2	2	2	2
3	21	26-Oct-15	2	2	2	2
6	43	26-Oct-15	2	2	2	2
8	13	26-Oct-15	2	2	2	2
6	52	24-Nov-15	2	2	2	2
8	5	24-Nov-15	2	2	2	2
6	35	24-Nov-15	2	2	2	2
7	25	24-Nov-15	2	2	2	2
7	46	24-Nov-15	2	2	2	2
8	56	24-Nov-15	2	2	2	2
8	21	24-Nov-15	2	2	2	2
7	24	24-Nov-15	2	2	2	2
6	43	24-Nov-15	2	2	2	2
4	41	23-Nov-15	2	2	2	2
6	43	23-Nov-15	2	2	2	2
7	53	23-Nov-15	2	2	2	2

7	29	23-Nov-15	2	2	2	2
6	17	23-Nov-15	2	2	2	2
8	35	23-Nov-15	2	2	2	2
7	39	23-Nov-15	2	2	2	2
9	57	23-Nov-15	2	2	2	2
7	13	23-Nov-15	2	2	2	2
8	33	23-Nov-15	2	2	2	2
7	57	23-Nov-15	2	2	2	2
6	39	23-Nov-15	2	2	2	2
8	58	23-Nov-15	2	2	2	2
9	12	17-Nov-15	2	2	2	2
6	58	17-Nov-15	2	2	2	2
7	20	17-Nov-15	2	2	2	2
10	10	17-Nov-15	2	2	2	2
7	49	17-Nov-15	2	2	2	2
8	28	17-Nov-15	2	2	2	2
7	27	17-Nov-15	2	2	2	2
9	13	17-Nov-15	2	2	2	2

asexpod28	gmcyxpod28	ascontd28	gmcycountd28	ascount1d28	gmcycount1d28	ascont28_uld28
2	2	0	0	0	0	0
2	2	0	0	0	0	0
2	2	0	0	0	0	0
2	2	0	0	0	0	0
2	2	0	0	0	0	0
2	2	0	0	0	0	0
2	2	0	0	0	0	0
2	2	0	0	0	0	0
2	2	0	0	0	0	0
2	2	0	0	0	0	0
2	2	0	0	0	0	0
2	2	0	0	0	0	0
2	2	0	0			0
2	2	0	0	0	0	0
2	2	0	0			0
2	2	0	0	0	0	0
2	2	0	0	0	0	0
2	2	0	0	0	0	0
2	2	0	0	0	0	0
2	2	0	0	0	0	0
2	2	0	0	0	0	0
2	2	0	0	0	0	0
2	2	0	0	0	0	0
2	2	0	0	0	0	0
2	2	0	0	0	0	0
2	2	0	0	0	0	0
2	2	0	0	0	0	0
2	2	0	0			0
2	2	0	0			0
2	2	0	0			0
2	2	0	0			0
2	2	0	0			0
2	2	0	0			0
2	2	0	0	0	0	0
2	2	0	0	0	0	0
2	2	0	0	0	0	0
2	2	0	0			0
2	2	0	0	0	0	0
2	2	0	0			0
2	2	0	0			0
2	2	0	0			0
2	2	0	0	0	0	0
2	2	0	0			0
2	2	0	0			0

2	2	0	0			0
2	2	0	0			0
2	2					
2	2	0	0			0
2	2	0	0			0
2	2	0	0			0
2	2	0	0			0
2	2	0	0			0
2	2	0	0			0
2	2			0	0	
2	2			0	0	
2	2			0	0	
2	2			0	0	
2	2			0	0	
2	2			0	0	
2	2			0	0	
2	2			0	0	
2	2	0	0			0
2	2					
2	2					
2	2					
2	2					
2	2			0	0	
2	2			0	0	
2	2			0	0	
2	2			0	0	
2	2			0	0	
2	2			0	0	
2	2			0	0	
2	2	0	0	0	0	0
2	2			0	0	

2	2	0	0
2	2	0	0
2	2	0	0
2	2	0	0
2	2		
2	2		
2	2		
2	2		
2	2		
2	2		
2	2		
2	2		
2	2		
2	2		
2	2		
2	2		
2	2		
2	2		
2	2		
2	2		
2	2		
2	2		
2	2		
2	2		
2	2		
2	2		
2	2		
2	2		
2	2		
2	2		
2	2		
2	2	0	0
2	2	0	0
2	2	0	0
2	2	0	0
2	2	0	0
2	2	0	0
2	2	0	0
2	2	0	0
2	2		
2	2		
2	2		
2	2		
2	2		
2	2		
2	2		
2	2		

2	2		
2	2		
2	2		
2	2		
2	2		
2	2		
2	2		
2	2	2579	103160
2	2		
2	2		
2	2		
2	2		
2	2		
2	2		
2	2		
2	2		
2	2		
2	2		
2	2		
2	2		
2	2		
2	2	15	600
2	2		
2	2	15	600
2	2		
2	2		
2	2		
2	2		
2	2		
2	2		
2	2		
2	2		
2	2		
2	2		
2	2		
2	2		
2	2		
2	2		
2	2		
2	2	1	40
2	2		
2	2		
2	2		
2	2		
2	2		
2	2		
2	2		

2	2				
2	2				
2	2				
2	2				
2	2				
2	2				
2	2				
2	2				
2	2				
2	2				
2	2				
2	2				
2	2	151			6040
2	2				
2	2				
2	2				
2	2				
2	2				
2	2				
2	2				
2	2				
2	2				
2	2				
2	2				
2	2				
2	2				
2	2				
2	2				
2	2				
2	2				
2	2				
2	2				
2	2				
2	2				
2	2				
	2		0	0	
2	2				
2	2				
2	2				
2	2				
2	2				
2	2				
2	2				
2	2				
2	2				
2	2				
2	2				

2	2		
2	2		
2	2		
2	2		
2	2		
2	2		
2	2		
2	2		
2	2		
2	2		
2	2		
2	2		
2	2		
2	2		
2	2		
2	2	853	34120
2	2		
2	2		
2	2		
2	2		
2	2		
2	2		
2	2		
2	2		
2	2		
2	2		
2	2		
2	2		
2	2		
2	2		
2	2		
2	2		
2	2		
2	2		
2	2		
2	2		
2	2		
2	2		
2	2		
2	2		
2	2		
2	2		
2	2		
2	2		
2	2		
2	2		
2	2		

2	2
2	2
2	2
2	2
2	2
2	2
2	2
2	2
2	2
2	2
2	2
2	2
2	2
2	2
2	2
2	2
2	2
2	2
